# Integrating recommendations for transgender and gender non-conforming perinatal care in the NHS: A qualitative exploration of healthcare professionals’ views

**DOI:** 10.1371/journal.pgph.0005684

**Published:** 2026-01-07

**Authors:** Kathleen Miriam Brown, Alison Swartz

**Affiliations:** 1 Department of Health, City St George’s, University of London, London, England; 2 Faculty of Health, Science, Social Care and Education, Kingston University, London, England; 3 Division of Social and Behavioural Sciences, University of Cape Town, Cape Town, South Africa; PLOS: Public Library of Science, UNITED STATES OF AMERICA

## Abstract

Transgender and gender non-conforming (TGNC) people are utilising National Health Service (NHS) perinatal care services with increasing frequency. Despite this, many healthcare institutions do not have policy, education, or resources in place to provide appropriate care. Research on the experience of TGNC pregnancy service users is limited within an NHS context. This study employed a qualitative approach to explore NHS midwives’ views of the acceptability and feasibility of current recommendations for providing care for TGNC people in pregnancy. We conducted 10 semi-structured interviews with midwives that lasted 25–80 minutes. These were audio recorded, and transcribed verbatim. Transcripts were read, reread and coded by author 1, in discussion with author 2. Analysis was informed by queer theory. Initial codes were then collapsed into themes using thematic analysis. Participants shared their philosophies of care provision and practical integration ideas to assist the implementation of care recommendations in their care settings. Findings were grouped into three main themes: (1) external barriers, (2) safety, and (3) eagerness to learn. Each of these key themes contained overlapping sub-themes. Whilst not a key theme, internal barriers was additionally explored. The research demonstrates a desire of participants to provide physically and psychologically safe individualised care. Participants acknowledged their role within a wider multi-disciplinary team (MDT) in addition to their role within a healthcare institution. Participants felt additional education was required for appropriate care for TGNC service users. The research demonstrates the emotional processes of midwives working within the NHS caring for a minority patient group. Greater support from institutions is required to provide gender-affirmative care. Policy is needed on a national and institutional level to support midwives to provide safe care. Further research may also strengthen existing recommendations and therefore support both midwives and TGNC service users.

## Introduction

In the last decade, transgender and gender non-conforming (TGNC) people have become increasingly visible within wider society [1]. It is therefore more likely for TGNC individuals to access and disclose their identity when presenting to healthcare services. Gender reassignment is a protected characteristic under the Equality Act [2], regardless of whether a person has undergone, is in the process of undergoing, or is planning to undergo transition. It is therefore illegal to discriminate against a person on the grounds of gender reassignment, and these people cannot be treated less favourably than others. Within the National Health Service (NHS) Constitution [3], all service users are entitled to appropriate care that meets their needs and reflects their preferences. Service users have the right not to be unlawfully discriminated against on the grounds of protected characteristics.

Despite this, most healthcare providers lack general knowledge about transgender health [4]. TGNC people accessing maternity care has also become more frequent in recent years, with a dearth of evidence and guidance for healthcare providers [5]. TGNC people accessing perinatal care are a vulnerable patient group, with added stressors to their perinatal experience of fear of discriminatory treatment [6]. TGNC people are a minority demographic for which most NHS services currently have no policy or care pathways to support in pregnancy, on either a local or national level.

The impact of this is demonstrated within the Improving Trans Experience of Maternity Services (ITEMS) report that outlines the health inequalities of TGNC people in comparison to those of cisgender service user [7]. With most research in this rapidly evolving field focusing on US healthcare systems, the ITEMS report is invaluable in comparing experiences of service users within the NHS. The ITEMS report found 28% of trans and non-binary respondents felt they were not treated with dignity and respect during labour and birth, compared to only 2% of pregnant people (assumed to be cisgender) surveyed in the Maternity Services Survey [8]. Some limitations of the ITEMS report are acknowledged, in particular the variations in care provision within the 30-year period in which ITEMS respondents accessed perinatal care. However, in an area of such limited research and in particular limited research within an NHS setting, this report is invaluable to demonstrate blatant inequitable health provision and directly contradicts care recommendations made by the Nursing and Midwifery Council [9].

There is a growing body of literature outlining TGNC peoples’ experiences of reproductive healthcare with fairly consistent findings of how this experience can be improved. Somewhat neglected within current research is the needs of healthcare providers (HCPs) in terms of educational development, guiding policy, and resources to enable positive experiences and outcomes for TGNC service users. The current evidence demonstrates both poorer outcomes and experiences for TGNC service users, highlighting the need for HCPs to be provided the means to provide safe and effective care. Pfeffer et al. [10] found HCPs commonly gave outdated guidance, inaccurate information, or cast judgement on TGNC pregnant people. Pfeffer et al. [10] do recognise however that HCPs do not typically receive targeted training surrounding TGNC pregnancies. In order to improve care provision, the needs of HCPs must be met, and structural change within healthcare institutions must support this. Individual HCPs can work to ensure their care provision is non-judgemental and person-centred, in line with professional recommendations [9,11]. However, without the necessary education, policies, and resources both physical and educational, NHS pregnancy services cannot provide consistent safe and effective care for TGNC pregnant people.

Within a UK setting, pregnancy care for healthy pregnant people is led by midwives. Pregnant people with medical complexities or pre-existing health conditions have care led by an obstetrician, and additionally see a midwife for antenatal checks, during their birth, and for postnatal checks. The authors aimed to understand the perspectives of midwives providing care for TGNC service users within the NHS. This was done by conducting semi-structured interviews with midwives working within the NHS. Care recommendations for TGNC collated from a literature review were discussed with midwives to understand the feasibility of these within NHS settings, in addition to the needs of midwives to provide care. This aimed to bridge the gap between the needs of TGNC service users and midwives to create opportunities for safe, effective, and gender-affirmative perinatal care within the NHS. In addition to this, the research provided deep emotional insight into the concerns and priorities of midwives, which can be used to frame training, policy, and to ensure recommendations are appropriate for care providers. Some findings may be transferable to other high income country healthcare systems, though would require collaboration with local stakeholders to ensure appropriate integration into existing systems.

## Methods

This study used qualitative research design to explore midwives’ views and experiences of providing care for TGNC patients in the context of the NHS. The study utilised in-depth semi-structured interviews with individual midwives to generate data. Gender within the context of this research relies on the NHS definition, referring to “our internal sense of who we are and how we see and describe ourselves” [12]. However, within a context of gender and feminist theory, gender can also be understood as cultural norms and expectations between men and women including social, physical, economic, political, and ideological imbalance [13]. Whilst within healthcare, sex is viewed as “biological” and relating to genes, reproductive organs, and hormones [12], Butler [14] argues sex can only be understood through gender, as “gender is the cultural significance the sexed body assumes”, with its significance “codetermined through various acts and their cultural perception” [15]. Therefore, whilst viewed in a simplified form within the research, the understanding of gender applied draws on queer, gender, and feminist theory to convey its cultural significance. Gender affirming care is understood as defined by WHO [16]: “any single or combination of a number of social, psychological, behavioural or medical (including hormonal treatment or surgery) interventions designed to support and affirm an individual’s gender identity”. These definitions and guiding frameworks are incorporated throughout the research in addition to guidance and ethical considerations for undertaking TGNC health research [17].

The overarching lens for data interpretation was queer theory. Within healthcare, “queer theory offers theoretical tools to disrupt biomedical norms and challenges biomedical normativity… Queer theory’s emphasis on normativity serves the political aim to subvert marginalisation and bring about radical social and material change” [18]. Whilst this research acknowledges the patriarchal framework on which “modern” healthcare systems are based, and the cis- and hetero-normativity this entails, queer theory is used to challenge “the normative social ordering of identities and subjectivities” [19, page 5], and analysis of the power relations between midwives and service users.

### Recruitment and sampling

Author 1 is a midwife who currently works in the NHS. The study was initially advertised using a poster through social and professional networks using a snowball recruitment approach. Professional colleagues were also encouraged to share the study though their social and professional networks. Recruitment commenced 05/01/2024 and closed 01/03/2024 as hypothetical data saturation was reached.

**Fig 1:**
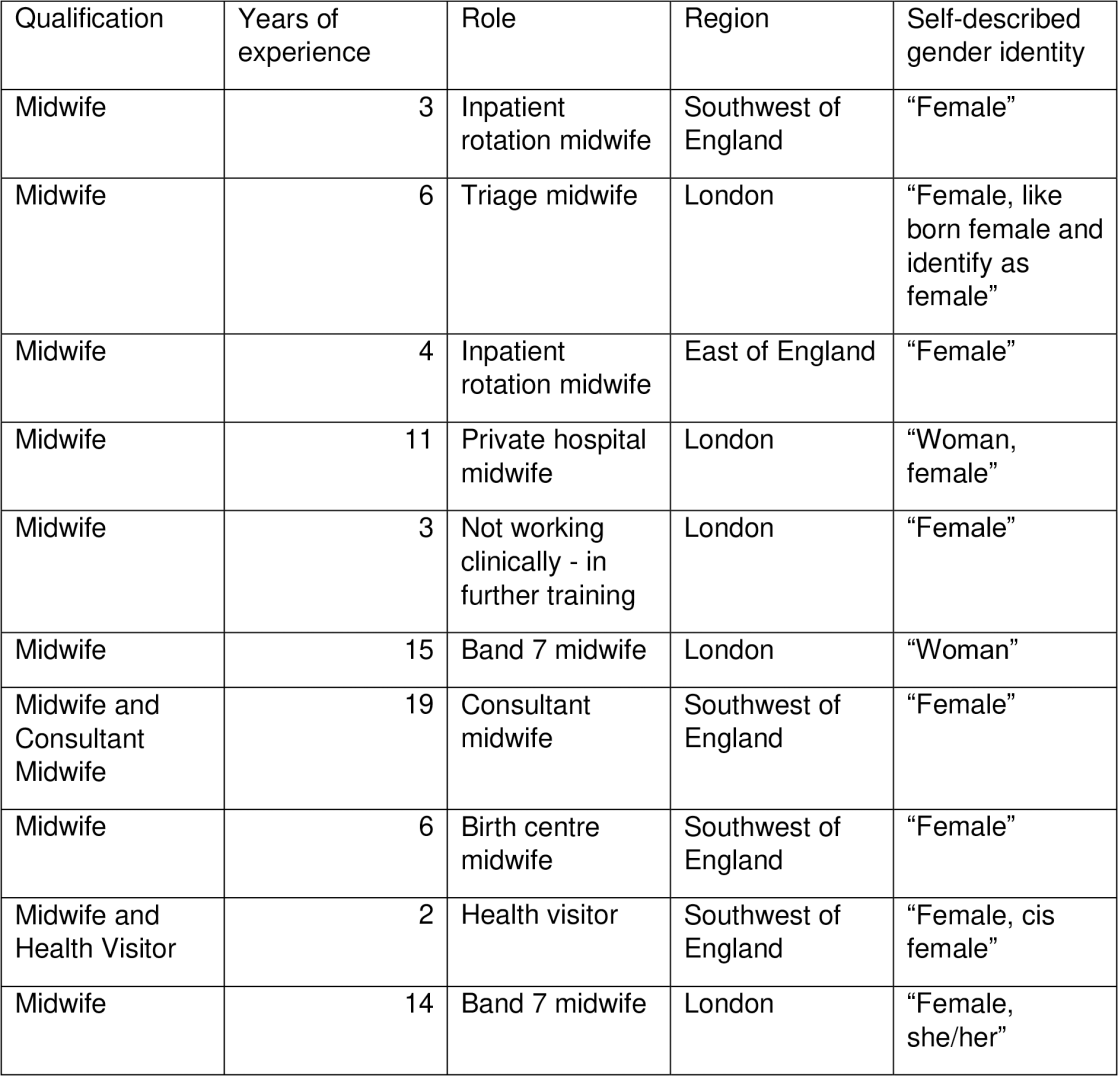
Table showing demographic details of interview participants.

The study was limited to midwives working in the UK within the NHS. The final study sample included nine midwives, and one health visitor who also held a professional qualification as a midwife. All participants identified as a “woman” or “female”; with several clarifying they identified as cisgender women. Professional qualifications, years of experience, area or working, and gender identity are explored in Fig 1.

### Data collection methods

Participant information sheets were sent via email prior to interviews with St. George’s Research Ethics Committee consent forms. Consent forms were completed by participants prior to interview. Verbal consent was obtained at the start of each interview and recorded within the transcript. Interviews were informed by a semi-structured interview guide that sought to explore the current evidence base of recommendations for TGNC pregnancy care provision and the feasibility of their integration within NHS care settings. Interviews took between 25 and 80 minutes. The semi-structured interview guide was developed based on a comprehensive literature review of evidence-based recommendations for TGNC care provision.

The literature review was completed in January 2024. A search strategy was designed to select appropriate literature. The search explored the following databases: NCBI PubMed, BioMed Central, Ovid, Cochrane Library, and Hunter Library. Keywords and Boolean terms used included transgender, pregnancy, recommendations, experience, male, birth, gender non-conforming, gender identity, midwifery, obstetrics, transgender AND pregnancy, transgender AND pregnancy AND recommendations. Search terms were used in various combinations. Inclusion and exclusion criteria and rationale for these was developed. The search was limited to literature written in English to ensure correct interpretation of literature. The search was not limited by year of publication, however no relevant articles published prior to 2014 were identified. Articles were excluded due to lack of relevance, such as focussing on contraceptive care, preconception care, pregnancy loss, lactation, non-pregnancy specific care, or not including recommendation for HCPs. Two articles were excluded for repetition. In total, 12 research articles or reports were identified. Full text was accessed for all articles. Four of these articles were excluded for not being specifically relevant to the niche of the topic or for not conducting original research. This left the eight papers that were critically appraised, found to be of high academic standard, and included in the literature review.

An overview of each paper was summarised (S1 Appendix), and all recommendations for care providers were documented (S2 Appendix). Recommendations were categorised by themes: education, language and literature, antenatal care, labour care, postnatal care, documentation and electronic medical records (EMR), environment, and institutional organisation. The recommendations which appeared most frequently were used to create the interview guide (S3 Appendix) for the semi-structured interviews. These recommendations were: training as a part of continued professional development on cultural competency and sexuality and gender diversity, asking service users their pronouns, asking service users their preferred terms for their anatomy and bodily function, gender inclusive EMR systems (allowing sex to differ from gender, allowing male patients admission to labour ward), documentation of correct pronouns, and gender inclusive posters and decoration.

The interviews followed a narrative approach [20] where honesty, sympathy, and respect were paramount to the process. Hypothetical data saturation was set at 10 participants for both feasibility and acceptability within the range proposed by Hennink and Kaiser [21]. Whilst conducting interviews, thematic saturation appeared to be reached by the eighth interview, however a further two were completed to ensure saturation was achieved.

Recommendations were made for midwives, healthcare institutions, policy makers, and for further research. The responsibilities of institutions and organisational bodies are emphasised in lieu of that of individual midwives.

### Data analysis

Findings of the interviews were analysed thematically and coded using NVivo using thematic analysis and queer theory. Commonly occurring assertions, experiences, and beliefs created the key themes which were treated as categories for the findings and discussion of the study. Queer theory was utilised to extract meaning from codes “through sociohistorical, contemporary, and self-reflexive lenses” [22]. The aim therefore was to understand participants’ responses within a wider context of a cis- and hetero-normative patriarchal society, and by extension a cis- and hetero-normative patriarchal healthcare system.

To ensure confidentiality and anonymity for participants, recordings of the interviews were stored securely on the university network behind a password protected account. Identifying details were removed from the transcripts.

The findings do not aim to add to the “great deal of ‘midwives should’ writing” [23] by creating prescriptive lists of solutions unachievable within an understaffed, underfunded NHS perinatal care setting. The interpretations of the data were informed by the authors positionality. Both authors identify as queer, with the first author working as a healthcare provider and thus as an “in-out-sider” [24] as both a queer person, and a healthcare practitioner.

**Fig 2:**
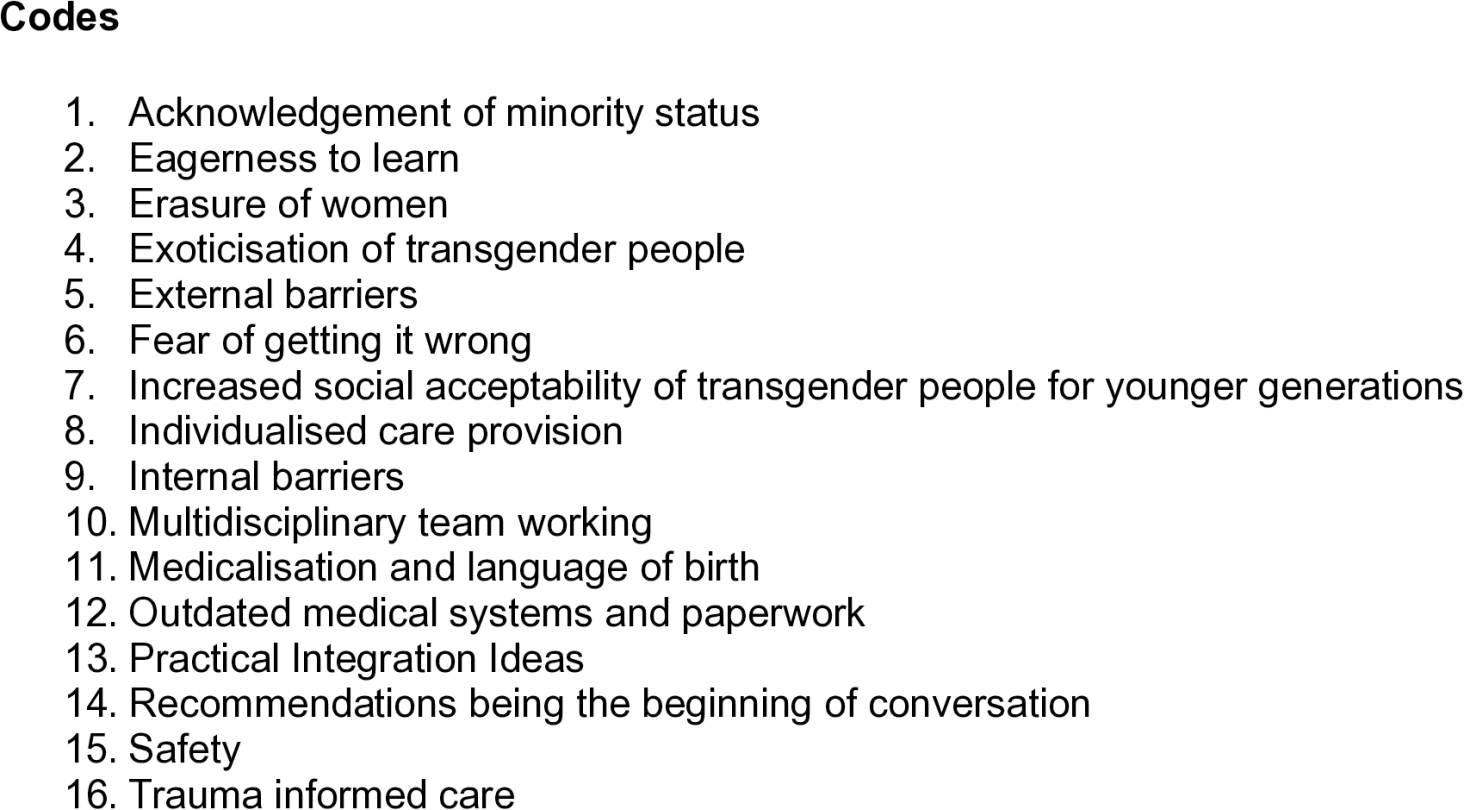
List of codes generated from participant interviews.

Sixteen codes were generated from transcript analysis (Fig 2). Some elements of interviews aligned with multiple themes, however each theme was notable and justified separate analysis. Codes with the most references are viewed as the key themes. The themes are distinct, but overlap between other codes, referenced from this point forward as sub-themes, which will be explored within each key themes.

The code “trauma informed care” was only mentioned by two participants. Despite this, the code remained included due to its importance as a framework for care provision [25,26]. This is included with the acknowledgement that trauma informed care was not explicitly explored by all midwives.

The code “practical integration ideas” was explored separately as these did not contain the same depth of emotional exploration from interview participants. Practical integration ideas encompassed four categories: pronouns and language, gender inclusion, education for staff, and education for TGNC service users (S4 Appendix). Excluding practical integration ideas, 14 distinct codes for analysis were generated from interviews.

Ethical Approval was granted by St. George’s University of London under REC reference 2023.0242.

## Results

Across all interviews was a yearning to provide safe and sensitive care for all service users. Whilst degrees of knowledge and experience with TGNC service users varied dramatically, all participants highlighted their desire to ensure that all service users had a comfortable experience of care and birth. Much of what was discussed during the interviews also linked back to participants’ fears, experiences, and eagerness to improve their care provision.

Before moving to an exploration of more detailed themes, it is important to note that the concept of equitable care varied between midwives. Some minimised TGNC identities by underscoring that care would be “the same as anyone else” (participant 9), whilst others recognised the specific needs of TGNC service users, including medications, surgeries, and mental health. One participant explicitly pointed to the need for “not just equal but equitable care” (participant 10), stressing the need for understanding and appropriateness of all care provided. Another stressed the need for “relationship building, creating a trusting relationship where someone feels able to access care” (participant 8).

**Fig 3:**
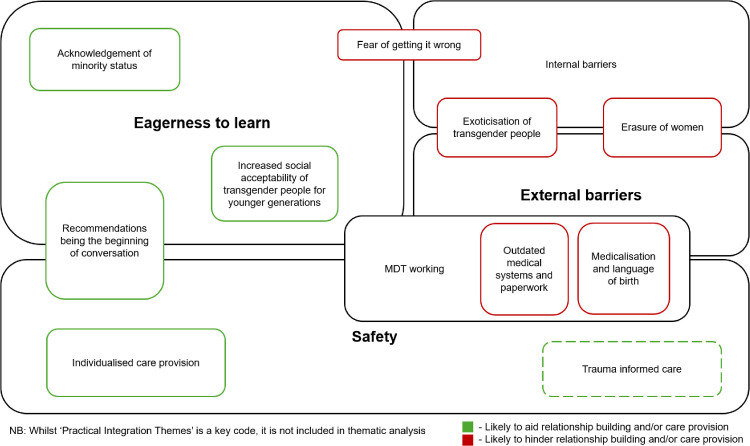
Diagram showing key themes and overlapping sub-themes from participant interviews.

With the framework of queer theory, the following themes will be explored; (1) external barriers to care provision, (2) physical and emotional safety of care provision, and (3) eagerness to learn how to provide care. Although not referenced as frequently as these themes, internal barriers will be discussed. Internal barriers was chosen due to the depth and reflective nature of the responses during interviews. Practical integration ideas are discussed to help frame recommendations for practice and highlight the solutions posed by midwives to assist them to provide gender affirming care. As stakeholders within their institutions, it is important to centre their ideas and priorities in tandem with priorities of TGNC service users to ensure recommendations for care provision are both appropriate and feasible. There was also overlap between some of the key thematic sub-sections, illustrated in Fig 3.

In the sections that follow we explore some of the key themes in more detail.

### External and institutional barriers

*“What’s the need and where does that come on the massive list of priorities that we’ve got and is that the most appropriate way to spend our money and time and effort or whatever it is?”*

*Participant 6*

Prior to discussion of the findings of the literature review, participants were asked about their backgrounds, experience, and philosophies of care. Following this, participants were asked to imagine there were no constraints on their practice, and to describe how this would change their philosophy and care provision. A common limitation to care provision was time. The viewing of philosophy of care as constant rather than situational was consistent across all participants, and significant in demonstrating their commitment to their care provision.

*“I don’t think it would be different, but I think I’d have more time to be able to treat people the way that I would want them to be treated… And so, I’d say, although my philosophy would probably be similar. I would be able to do it better.”*

*Participant 1*

Pressures of time, staffing, and funding are well-documented in NHS services, and in particular perinatal care services [27–29]. These provide notable context as to why care needs of vulnerable patient groups may not be appropriately prioritised. In particular this is an important consideration as due to their minority status within healthcare settings, TGNC service users may be affected by these constraints on care in addition to more specific shortcomings in care provision. There is therefore a compounded burden on TGNC service users: to advocate for themselves as birthing people in addition to advocating for themselves as TGNC birthing people.

External barriers overlapped with many themes perceived by participants as negatively impacting care provision and relationship building in addition to institutional barriers. Namely, outdated medical systems, and medicalisation of birth. External barriers additionally overlapped with internal barriers, and in particular exoticisation or othering of TGNC service users and fears around erasure of women. Lack of training and experience was commonly cited as affecting participants’ confidence in providing care for TGNC service users. Multidisciplinary team (MDT) working was discussed in both positive and negative aspects by participants. Ineffective MDT working was discussed in relation to external barriers to care provision, whilst effective MDT working was discussed in relation to ensuring service user safety.

An external limiting factor for midwives in providing appropriate care for TGNC was the EMR systems in place. There is no standard EMR system for perinatal care services across NHS settings, and a variety of systems are utilised. Despite this, participants who reported using different EMR systems reported similar limitations. These included: inability to record gender separately to sex assigned at birth, inability to add a pregnancy record to service users recorded as male, and inability to register admittance of a male service user to “female” inpatient wards, such as an antenatal ward. This led to a variety of non-standardised “solutions” which varied significantly between institutions. Solutions included changing service users’ sex to female, adding a separate record with different record numbers which was connected to their medical records, or utilising only handwritten notes. None of these solutions were deemed satisfactory or feasible by participants, who reported these EMR limitations were often not investigated in advance of appointments or admittance of TGNC to inpatient areas. They reported EMR limitations as taking clinical staff away from providing care as they attempted to find ad-hoc solutions in addition to being “embarrassing” and a “clinical risk” (participant 5). Limitations of tools and technologies can be understood to impact safety and ability of midwives to provide care through a human factors framework [30]. Participants did not feel there was a clear solution to these challenges however, they did highlight the role of digital midwives and the administrators of EMR systems to develop solutions.

*“We created the same name with a different MRN number, connected to the male record was a female record with the pregnancy added to it. So, we added any documentation for the pregnancy on female record, but he actually had a different MRN which was his male record that was used, you know, if he had to go into hospital for anything else, that would be his record. So, I can just see that this is gonna cause problems in the future as well.”*

*Participant 5*

Whilst there was a deep appreciation for the role of the MDT expressed by the participants, the MDT was seen as a potential area where miscommunications could occur. In particular, emergency situations where members of the MDT attending had been unaware of the service user’s history and used incorrect pronouns for them (participant 7). Participants grappled with ensuring that all members of the MDT were appropriately informed and creating an unnecessary and unwanted focus on a service user’s gender. In addition, participants could not clearly identify who the appropriate care provider to refer to would be when caring for TGNC service users. Participants reported examples of care being led by teams aimed to care for those with complex social histories or mental health diagnoses, and examples of care being led by complex medicines teams, both of which were appropriate for the case in question but recognised as not appropriate for all TGNC service users. Additionally, participants often felt they did not have the resources or knowledge to address specific concerns or considerations of TGNC service users, such as how having a caesarean birth would affect phalloplasty in the future (participant 5) and additional precautions in the cases of testosterone use at conception or prior to pregnancy (participant 9). This aligns strongly with areas where TGNC service users have attempted to seek further information from midwives and found insufficient knowledge, highlighting the importance of providers “differentiat[ing] between ‘I don’t know’ and ‘science doesn’t know’.” [31]. Therefore, lack of clear pathways for TGNC service users led to inequities with service provision, with appropriate care provision depending on the knowledge of individual midwives, unsupported by institutional policies or training. Participants highlighted many perceived aspects of caring for TGNC service users as outside their sphere of competency. Whilst understandable for individual practitioners, this is unacceptable for care institutions as a whole, who have a responsibility to provide comprehensive and equitable care to all, as outlined within the NHS Constitution [3].

*“Making sure that I am giving the care that they are expecting and also kind of making sure that as a team, we are providing the care that they are expecting because I’m very much aware that I am one member of a big team.”*

*Participant 9*

*“It’s just in terms of making people more likely to access care and making sure that staff are providing the same quality of care to everybody because as much as we would like to say that everybody would provide the same level of care, you know that staff in some situations find when they’re uncomfortable or not confident, they’re less likely to.”*

*Participant 1*

Labour and birth were highlighted as a time where inappropriate language and pronouns may be used, due to being at higher likelihood of exposure to multiple midwives and a higher chance of needing wider MDT involvement in comparison to the antenatal and postnatal period. Labour and birth were also viewed by participants as a time where it may not be appropriate to ask questions about preferred language, and where utilising preferred language in lieu of “medical” language may cause miscommunication between HCPs. Therefore, the importance of birth planning and continuity of carer were highlighted by some participants to minimise disruptions in birthing environments and allow midwives to advocate for TGNC service users. Participants were aware of these barriers however did not always envision clear solutions to overcoming them, especially when they seemed outside of their control. Participants were aware of their own sphere of practice and the individual impacts they could achieve, and acknowledged the importance of these. However, external barriers were seen as affecting relationship building, service user experience, and in some cases, service users’ safety.

*“Pelvic opening might not necessarily be seen as a medical term for it. It is more of a preferred term, same as breast and you have breast tissue, not chest tissue, but obviously so I think probably like in terms of medical things to clarify what you’re talking about, it would be hard to integrate that.”*

*Participant 4*

### Safety

Safety was viewed by participants as multifaceted, with importance placed on service users’ physical safety in tandem with their psychological safety. Participants recognised their role in contributing to a multitude of safeties, however felt that their institutions focused more on physical safety than psychological safety. Participants argued however that there was a strong link between types of safety. In particular, participants voiced concerns that if TGNC did not feel supported and psychologically safe within healthcare institutions, they may choose to limit access to them or not access care entirely. Participants felt therefore that without psychological safety, positive relationships could not be built, and care provision would be affected, which in turn would affect physical safety. This is in line with the findings of the ITEMS report [7], in which 30% of respondents reported they did not access NHS or private healthcare support during their pregnancies. Only 20% of respondents within the ITEMS survey “reported feeling comfortable accessing maternity services” (page 30). Conceptualising psychological safety as affecting physical safety additionally aligns with research within broader perinatal care services. From Better Births: “Women told us how important it was for them to know and form a relationship with the professionals caring for them… It was felt that this could provide better support for women, and enable midwives to better meet their needs, identify problems and provide a safer service” [32]. Therefore, whilst evidence applying prioritisation of relationship building to TGNC perinatal care is limited, with continuity of carer recommended in only two of the papers within the literature review, we can infer importance as with any person accessing perinatal care.

*“I think it’s just first impressions, relationship building, creating a trusting relationship where someone feels able to access care again. This is quite major when it comes to whether they’re likely to receive antenatal care, if they attend hospital in labour, or, you know, access support if they think something’s not going right. So, I think, yeah, first impressions count, so if we don’t meet their needs at the beginning, then we could possibly… They’re not going to access care at all.”*

*Participant 8*

In parallel to relationship building, individualised care provision was highlighted by participants as important to ensure physical and psychological safety. Many methods of facilitating individualised care provision were proposed by participants, as outlined within the practical integration section. Individualised care included using TGNC service users’ preferred language, discussing mode of birth and mode of feeding, and listening to the needs and priorities of the service users. Additionally, to ensure psychological safety, the importance of appropriate dissemination of this information between HCPs was highlighted, in particular with members of the MDT and at handovers. This was linked to planning and dissemination ahead of birth and ahead of HCPs interacting with TGNC service users. Individualised care was felt by participants to rely on communication between the service user and midwife in addition to communication between individual midwives and the wider MDT.

*“It could be quite confronting to go into labour and birth and have to, you know, talk about body parts, or maybe didn’t want to talk about and be asked a lot of personal questions. It’s probably quite a big thing, so I’d definitely say that planning aspect would be quite important.”*

*Participant 3*

Although only framed as such by two participants, the concept of safety is a key principle of trauma informed care, which “aims to promote feelings of psychological safety, choice, and control” [26, page 13]. Whilst this term was not commonly presented as a utilised framework, many aspects of a trauma informed care framework were used in participants’ responses. Further research into trauma informed care provision for TGNC perinatal service users could provide evidence into its acceptability.

### Eagerness to learn

All participants expressed a desire, and in some cases a perceived need, for additional training for midwives focused on TGNC healthcare and perinatal care provision. Training was perceived as feasible by most participants, however one participant highlighted “the massive impact to our service provision and the cost” of training (participant 6). This is a valid critique somewhat overlooked within the research. Within an NHS context, the Core Competency Framework [33] outlines the training institutions should provide perinatal care staff. Therefore, in order to provide additional training or training not covered by this framework, institutions may incur additional expenses in funding the training itself, paying midwives to attend the training, and paying other midwives to work clinically whilst midwives attend training. Therefore, to fund appropriate training, policy must view TGNC service users as a priority.

*“So, as a simple like suggestion, recommendation from a piece of evidence, yeah, it totally is like your goal, number one, that you just write down. We’ll provide training, lovely. Everyone says it like a tick box thing, done. Like the realities are really hard in terms of who delivers the training, what does the training… what does the training mean? What’s going to be backed up behind the training? What do we need to support that to make it a real part of our organisational culture and knowledge base?”*

*Participant 6*

Midwives’ attitude of normalising or othering TGNC service users fluctuated throughout their interviews, seemingly by how confident they felt with the care provision described. This awareness of personal limitations in terms of knowledge and care provision also featured prominently in responses. Awareness of one’s own competency, role within the MDT and when further resources and training are important factors in ensuring the safety of care provision [9]. Therefore, whilst this personal awareness and reflection on practice demonstrates consideration on the part of midwives interviewed, it also demonstrates the need for training, policies, and support to care for TGNC service users to ensure safe care provision.

Different methods of training were discussed, including in-person and e-learning packages. Participants reported increased value of in-person training led by “skilled facilitators” (participant 10). Participants did not feel that facilitators necessarily needed to have a clinical background, but instead would be able to foster healthy discussion, educate with room for discomfort, and address misconceptions. Examples of appropriate facilitators for this training were those with experience of TGNC healthcare, people who identified as TGNC, or TGNC midwives. Facilitators would ideally by “experts by experience” (participant 10). Participants felt that education on “the basics” (participant 8) including language and pronouns would be most helpful, alongside case studies.

Participants also stressed the importance of any training being within the MDT, which aligns with recommendations from Better Births [32]. Notably, MDT training programmes such as PROMPT report improvements in physical safety, reducing hypoxic brain injury and maternal death [34]. Further research would be needed to demonstrate the impact of MDT training on psychological safety. Recognising the importance of MDT training denotes the participants’ awareness of themselves within an MDT and healthcare institution. Training and education were viewed by participants as a starting point from which other positive changes to care for TGNC service users could flourish. Framing the importance of training in tandem with MDT training allowed participants to envision a difference in organisational culture and norms. Viewed through a more pessimistic lens, this could also be interpreted as participants seeing a strong need for education for their colleagues and perhaps witnessing or being involved in inappropriate care provision for TGNC service users.

### Internal barriers

Although participants had a detailed understanding of external barriers affecting care provision and could answer confidently and coherently, participants were more hesitant when discussing perceived internal barriers. This was explained by participants as internal barriers being more difficult to untangle, or not something they had thought about before. One outlier to this response was a participant who spoke in detail about the importance of recognising “unconscious bias”. This phrase allowed the participant to use an introspective analysis of their own experiences, however this phrase was not used by any other participants.

Some participants referenced their age as a limiting factor for providing care for TGNC clients. This was also referenced by midwives who did not self-identify as being “older” but saw “older” midwives as more likely to struggle with providing inclusive care. A potential consideration for viewing of gender variation and inclusion as more acceptable for younger generations and therefore younger midwives is the framing of gender inclusive practice as (1) exclusionary for older midwives and (2) a passive process which does not require active participation from the HCP or their institution. This grouping by generation could be harmful to older persons who have worked to be advocates and allies, and harmful for younger persons who may not be viewed as requiring training on gender inclusion. This framing also excludes older persons who are TGNC. Therefore, whilst an interesting theme across interviews, this perspective requires critique to ensure it does not influence policy makers to assume improved care will occur spontaneously over time without input or training. In addition to this, whilst a midwife’s personal experience may inform their practice, this cannot be used as a rationale not to provide appropriate care. It is standard for practice to evolve with time and in line with evidence and therefore care for TGNC service users must align with this.

*“I think it’s something that’s very unfamiliar to me because I haven’t grown up with this”.*

*Participant 2*

*“I would possibly say older generations have a more negative view.”*

*Participant 3*

Internal barriers were often spoken about in relation to external barriers, or participants referenced internal barriers they perceived in other staff members or the general population. An unexpected finding of this was, even when not referenced by name, participants held a strong awareness of trans-exclusionary ideologies present within feminist and birthing education and advocacy spheres. Although participants separated themselves from this ideology, several participants referenced their fear over the removal of the word “woman” from perinatal care and spaces. The topic created malaise within participants as they grappled between a perceived erasure of women, evoking the phantasm of “gender ideology” described by Butler [35], in conjunction with the importance of ensuring inclusion. Although not expressed as such by all participants, this can be understood as participants’ valuing their own gender identity and wanting to ensure this is included, respected, and represented. This is of course an important consideration as the majority of service users seeking perinatal care do identify as cisgender women and are equally entitled to affirming and inclusive care provision. Participants interviewed were able to recognise the importance of these inclusive languages in tandem.

*“Thinking about conversations I’ve heard, I think, if I’m being really honest, I think trans people are one of the few groups of people that people still think is socially acceptable to ridicule.”*

*Participant 8*

*“I’ve only heard amongst midwives; I’ve only really heard… A few sort of overheard conversations. Almost. And with some midwives feeling kind of disgruntled about. Changing language generally, but particularly that the fear that the word woman’s going to get taken away.”*

*Participant 10*

However, participants also referenced the opinions and fears of other midwives they knew which were more critical of TGNC service users, and reported midwives vocalised these concerns within care settings. The fears and opinions expressed by participants when a discussing phantasmic erasure of women is insightful into a wider political climate of conceptions of “gender”. The abjection of TGNC people allows an individual choosing to become a biological parent to become a wider political and cultural issue subject to scrutiny. Additionally, fears surrounding erasure of women invoke semblance to replacement theory, discussed by Butler [35]. This is most certainly an area requiring further research and discussion which cannot be adequately discussed within this research. However, it is mentioned to highlight participants’ awareness of the pathologisation of individual service users as representations of a perceived agenda. This directly contradicts many of the priorities reported by participants, in particular ensuring care is individualised and person-centred. Whilst every individual is entitled to their own opinions, the importance of providing fair and non-discriminatory care is enshrined within the NMC [9] and GMC [36]. Therefore, this is concerning when considering the midwives they reference are likely providing care for TGNC service users and are not behaving in line with their professions guiding principles.

*“It feels like a really difficult push pull for me on this one, because then the other part of me goes I do understand the whole thing around not getting to the point where we have… like we’ve… we’re afraid or we don’t… We don’t, we don’t talk about women. And I have that. I do... I understand that and I relate to that as a woman, and where I don’t even think we have… And this again is the same thing though, because we don’t even have what we need and want as women in society or in our lives.*

*And then so I’m acknowledging that as true. And then I’m also acknowledging the fact that then that therefore means that people who identify as male but can have babies and do want to have babies and things and have to do that process to become parents, they’re going to be feeling that on a whole other level.*

*So, it’s like I can hold all of these truths as reality, and it gets really muddied as to what the best way forward is then.*

*But I’m very aware of… I’m very aware of the need to listen and to value and to respect everyone’s experience as an individual and for that on both sides, not to diminish the other or the others specific issues or terminology or wording, so I would like to get to a place where it can all be held as OK.”*

*Participant 6*

## Discussion

This study adds to growing body of literature of TGNC pregnancy care by analysing the attitudes and beliefs of midwives providing care within the NHS. Many of the findings of this study are congruent with other literature within the field, whilst also exploring novel findings and providing an in-depth analysis of midwives’ experiences.

Recognition of TGNC service users as a marginalised group and an awareness of transphobic attitudes was similarly found by Pezaro et al. [37]. Their study was similarly conducted in the UK, and their findings in tandem with this research provide an in-depth analysis of the attitudes of care providers. The main themes found within Pezaro et al.’s qualitative analysis included “challenges of providing care for childbearing trans and nonbinary people” and “need for education”. Within the theme of “reported experiences of providing care for childbearing trans and nonbinary people”, subthemes included “witnessing transphobia among colleagues” and “apprehension about providing care”. These themes align with our findings and the experiences shared by our interview participants, with “apprehension about providing care” categorised within our research as “fear of getting it wrong”. Perazo et al.’s “challenges of providing care for childbearing transgender and nonbinary people” aligned with our categorisation of “internal barriers” and “external barriers”. Miller et al.’s [38] study similarly acknowledges “structural” and “social” barriers, which are frames within our research as “external barriers”.

Lack of specific education on TGNC pregnancy is highlighted as a key theme in much of the literature [37–39] and aligns with the experiences shared by participants of our study. Education and training are framed within the literature as desired by health care workers, and key to facilitating appropriate care provision. In particular, methods of knowledge acquisition between Pezaro et al.’s [37] study and this research align regarding the needs of healthcare providers. In both studies, participants wished to be educated by TGNC people. Participants within Pezaro et al.’s study wished for sharing of best practice and open discussion on inclusivity. This aligned with the desires of participants of this study for education to be led by a “skilled facilitator” with expert knowledge. Studies with findings discussing need for specific education have been conducted in the UK [37], New Zealand [38] and the US [39]. The importance of specific education is also discussed in Parker et al.’s [40] article focusing on New Zealand and Canada. Whilst it is important to acknowledge the structural differences in care systems, it is likely that findings of these studies are transferable in part to other Western countries and contexts and together contribute to a global picture of pregnancy care provision and challenges for TGNC service users.

Kukara’s [39] research within a US context acknowledges the “erasure of [TGNC] patients from perinatal care systems”, aligning with our categorisation of “outdated medical systems and paperwork” and “medicalisation of language and birth”. This paper advocates for “the need for systematic change” and promotes a midwifery care model to facilitate individualised care provision. Although this finding must be acknowledged with the US system of care provision which differs significantly from the NHS system, our research also acknowledges “individualised care provision” as a positive theme of interviews, and a factor likely to aid relationship building between care providers and service users. In a UK context, midwifery care can be framed as having the “opportunity and duty to uphold reproductive justice” [41, page 1]. This framing of midwifery was explored in part by interview participants within their self-described philosophies of care. Whilst the framework was not referred to with this terminology, participants described a philosophy which often centred around providing individualised care and upholding autonomy, acknowledging the limitations of this within the NHS institution. They therefore recognised structural and systematic issues impacting care provision and acknowledged the additional impact of this on marginalised groups. This aligns with the reproductive justice framework [42].

Debate surrounding the use of sexed language in comparison to inclusive language is well documented. Proponents of the use of sexed language argue this ensures consistent communication with less opportunities for miscommunication both for staff [43] and service users with low health literacy or language barriers [44]. Proponents of the use of inclusive language argue this will aid relationship building and promote engagement with care services [45]. Midwives interviewed within this study discussed aspects of this debate and their responses mirrored arguments for both sexed and inclusive language. Awareness of a climate of anti-trans rhetoric within the UK [46] was demonstrated within participants’ exploration of “internal barriers”. It is worth consideration that sexed language and conditional inclusion create a “subtle but significant hierarchy” [47], which does not align with concepts of inclusive and equitable care philosophies or with principles of reproductive justice. The use of inclusive language by contrast has the potential to ensure individualised care on an institutional level.

Novel findings of this research are primarily the discussion of challenges and recommendations within an NHS care context. Our decision to utilise semi-structured interviews provided a stronger emotional insight to the participant’s experiences, attitudes, and beliefs. Our framing of themes and challenges as overlapping and interconnected provides a more nuanced understanding of the climate of pregnancy care within the NHS. This paper builds on previous research through utilisation of queer theory as a framework to situate the findings within the wider context of a cisnormative and heteronormative healthcare system and society.

### Implications for practice

Many of the responses and recommendations made by participants provide further clarity on how findings of the literature review can be appropriately integrated into an NHS setting. Several ideas aligned with the literature review findings despite not being mentioned by the researcher at interview. These include non-gendered options for cot cards, appropriate handovers, and antenatal classes and information aimed at TGNC service users. Although some suggestions made may be problematic and may require further clarification or discussion, such as pronouns above beds creating a potential stigma or disclosing private information to others on a ward. These integration ideas would benefit from review and consultation with key stakeholders including TGNC service users to ensure acceptability. Participants ideas for practical integration could also be considered in other high income country healthcare settings with review and consultation with local healthcare workers and stakeholders.

Participants recommended appropriate referral systems and pathways. These were mentioned in the literature review in relation to mental health following birth, but not in relation to perinatal care as a whole. The importance of policies and pathways was also highlighted by participants within research by Pezaro et al. [37]. This may be due to the positionality of the participants of the research within the literature review in relation to the participants of this research and Pezaro et al.’s research. Service users may be less likely to have an awareness of hospital guiding policy in comparison to midwives who use these documents to frame their practice. Alternatively, this could be viewed as a pathologisation of TGNC service users by participants due to the assumption their care will need specialist input or oversight unlike that of a cisgender service user.

Overall, responses and practical integration ideas made by participants of this research were appropriate and highlighted areas of additional resources, knowledge, and guiding policy for participants. Perceptions of feasibility differed between recommendations, with many areas of change perceived as outside their control as midwives. This included changes to ward signage, colour schemes, ward names, and service user information (e.g., patient leaflets). Ideas based on discussions with service users such as asking for correct pronouns and preferred language was seen as more feasible than broader changes to institutional structure. This may also reflect the priority vocalised by participants to ensure their care provision is individualised. Eight out of the 10 participants suggested asking for pronouns at the “booking appointment”, which is usually the first point of contact with an HCP during a service users’ pregnancy. One participant reported that asking a person’s pronouns at booking was a standard practice within their institution. Asking a person’s pronouns was viewed by participants as something which may cause offense to a minority but ultimately was no more invasive than other questions routinely asked. It is interesting that participants tended to externalise discussion of pronouns. Several mentioned the use of pronouns in email signatures becoming more frequent, however no staff recommended implementing pronouns on staff name badges or introducing themselves with their pronouns. Perhaps this can be understood as an assumption their pronouns are known due to external presentations of gender identity. As use of pronoun badges for staff is recommended in the ITEMS report [7], it would be useful to ask for HCP feedback on this recommendation specifically.

*“And we’re asking them demographic questions, and I know this is an identity question as well. But we’re it’s, you know, you’re asking them “do you… do you go by other titles?” You know there’s a whole range of questions that are completely valid and. And yeah, I think that’s fine.”*

*Participant 10*

When discussing recommendations and their integration, participants had an awareness of their ability as an individual within a larger institution and placed value on their role to affect individual encounters and experiences. Additionally, there was an awareness of the time, resources, and funding required for larger or more institution-wide changes to occur. Participants tended to externalise recommendations as information or resources they could be provided with to better facilitate gender affirming care. Practical integration ideas were in general limited to horizontal interventions rather than vertical interventions. Horizontal integrations have shown in some cases to incur lower costs for institutions in addition to increasing efficiency [48]. Vertical integration “involves patient pathways to treat named medical conditions that transcend organisational boundaries” [49]. Therefore, vertical integration and creation of policies to support TGNC perinatal care service users could create a greater shift in institutional norms with greater support and resources between organisations. Further research involving policy makers may provide further integration ideas which extend the realms of individual patient interactions.

## Recommendations

The participants were aware of their roles as individuals within a larger institution and aimed to work to provide safe and individualised care to all service users. However, participants also expressed fears they did not have adequate tools, knowledge, and resources to provide appropriate care to TGNC service users. Whilst the role of individual’s seeking knowledge as a part of continued professional development is important and incorporated into many healthcare registration requirements within the UK [50], the lack of institutional support is likely to create inequities in knowledge and therefore inequities in service provision. These inequities may affect both psychological and physical safety [51]. Increased support for midwives will therefore aim to improve care provision for TGNC service users and is essential to improve outcomes and reduce distress.

**Fig 4:**
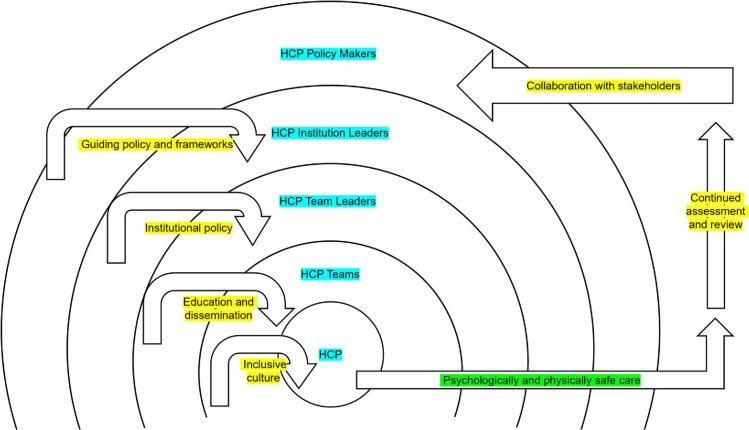
Outcomes framework demonstrating theory of change.

The lack of tools and resources can be conceptualised through a human factors framework which demonstrates the potential impact on service user safety when the correct resources are not provided to HCPs [52]. Hence, whilst primarily horizontal integration ideas were proposed by participants, vertical integration ideas should be considered by institutions and policy makers to improve support for care provision for TGNC service users within perinatal care settings. Therefore, the following Outcomes Framework is proposed in Fig 4 [53]. The long-term goals for care provision are service user’s psychological and physical safety. This is dependent on the conditions of individual HCPs, their wider team, institution, and national policy makers. Instead of a linear framework, components and outcomes must be considered in tandem and within the context of their role within the healthcare system. This additionally mimics the human factors framework in which lessons are learnt, and evidence is synthesised which allows improvements to be made continually.

**Fig 5:**
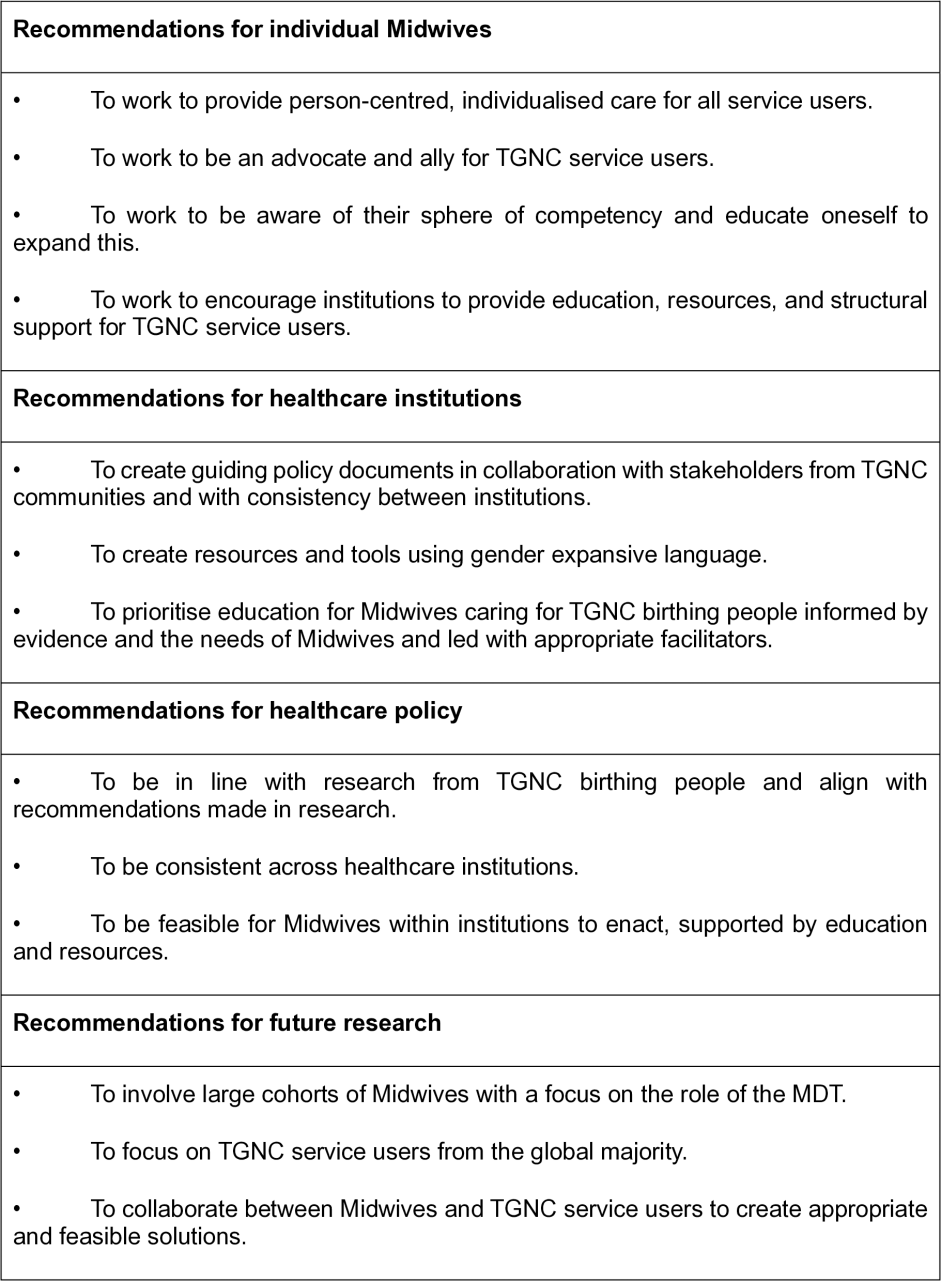
Recommendations for stakeholders.

The ultimate goal of care provision, in the eyes of the interview participants, is psychologically and physically safe care. This additionally represents goals of wider healthcare institutions and the NHS as a whole as outlined by the NHS Patient Safety Strategy [54], which aims to “continuously improve patient safety” by building on “two foundations: a patient safety culture and a patient safety system” (page 4). These aims are supported by the development of insight, involvement, and improvement. This is represented in the Outcome Framework within continued assessment and review in addition to collaboration with stakeholders. Safety is the responsibility of institutions and clinicians, as demonstrated as an output across all sectors of the Outcomes Framework. Additionally, assessment and review may come from all levels as represented with the arrow travelling from individual HCPs and escalating to institution leaders or policy makers. Stakeholders within the context of TGNC pregnancy service users may include TGNC service users, researchers, HCPs with specialised knowledge, and a wider team of institutional leaders. Recommendations for different stakeholders within healthcare roles are demonstrated in Fig 5.

Within the Outcome Framework care has been taken to demonstrate shared responsibility across healthcare systems, rather than placing onus on the individual HCP. The conditions are broad to allow them to be shaped by the needs of the system and service user, in addition to being guided by evolving evidence. It is additionally acknowledged that safe care as a goal is broad and may differ significantly between service users. Therefore, individualised care provision is required to ensure care is appropriate. Individualised care provision is supported by the prior conditions within the Outcome Framework, in particular an inclusive culture which prioritises the needs of service users. Individualised care provision is additionally supported by communication which centres the service user and avoids assumptions, which can be considered as an aspect of an inclusive culture [55]. Safe care must additionally by guided by existing and broader frameworks and evidence in addition to registrant requirements. TGNC service users must be entitled to safe and appropriate care in line with biomedical ethics and standards.

Whilst some practical integration ideas from participants may require additional screening for use in UK and other countries’ healthcare settings, the Outcome Framework is transferable across care settings. This has been achieved by providing an overarching recommendation to facilitate appropriate care, whilst ensuring policy and practice is guided by the needs of stakeholders. Therefore, the Outcome Framework could be considered in high income healthcare settings which operate in a similar model to the NHS.

## Limitations

The authors acknowledge several limitations including representation from different institutions and lack of inclusion of perspectives of TGNC service users. The ethical approval for this research included interviewing experts in TGNC pregnancy care and TGNC people who had utilised NHS pregnancy care services. These interviews were planned to follow the first round of midwife interviews to screen the recommendations made by midwives for acceptability. The authors did not anticipate the depth of emotional exploration from the midwife participants. Due to the majority of findings consisting of this, it was not viewed as appropriate by the authors to screen the emotional perspectives of the midwife participants for acceptability. Therefore, the decision was made not to conduct the second round of interviews with experts in TGNC pregnancy care and TGNC people who had utilised NHS pregnancy care services.

The research was conducted within the UK with a specific interest in NHS care settings and therefore may not be transferable in full to other healthcare settings or countries.

Responses at interview may additionally be biased by the framing of interview questions and social desirability bias. As seen in the interview guide (S3 Appendix), participants were informed the questions were framed by the findings of a literature review. Therefore, may have felt more compelled to agree with the findings posed due to the framing effect [56]. This is worth consideration when applying any recommendations to clinical practice and policy, and arguably a limitation of the study design. Responses may have also differed if more recommendations from the literature review were presented to and discussed with participants during interview.

## Conclusion

Within a relatively short period of time, literature surrounding TGNC pregnancy has increased to a degree that there is a growing evidence base for care recommendations. This is essential in ensuring appropriate service for TGNC service users. However, literature addressing feasibility and integration into current care services is limited. This qualitative analysis of the experience and perspectives of midwives providing pregnancy care adds to this growing field. The findings of this study demonstrate the desires of midwives to provide safe and appropriate care for all service users, inhibited by a lack of education, support, and guiding policy. Wider institutional factors such as resources and tools impacted participants physical ability to provide care, whilst wider societal factors such as anti-gender ideology movements were acknowledged as framing the thought processes of some participants. Therefore, to ensure TGNC pregnancy service users receive equitable care, greater consideration is required for the training of midwives which must be framed by evidence-based policy and consultation with stakeholders. This requires action from individual midwives and HCPs, institutions, and national policy makers, and must be seen as an ongoing process of learning and improvement rather than a static “tick-box” exercise. Responsibility lies with all involved in pregnancy care, rather than onus of education and safe care provision lying directly with midwives without wider institutional and national support. Midwives must additionally be receptive in acknowledging elements of practice and care which may require adapting for TGNC service users, in addition to acknowledging potential prejudices they hold. Healthcare institutions hold a responsibility to all service users, ensuring service users are respected, safe, and their dignity upheld. This applies equally to TGNC service users, who must be viewed as individuals rather than representatives of a wider perceived agenda. Commitment and action are required across multiple levels of healthcare systems to ensure these ideals are upheld and TGNC service users receive the care they are entitled to. This must consult midwives to ensure feasibility of action within existing systems and frameworks.

## Supporting information

S1 Appendix Table summarising research included in the literature review. (DOCX)

S2 Appendix Table demonstrating recommendations made by research included in the literature review. (DOCX)

S3 Appendix Interview guide. (DOCX)

S4 Appendix Interview participant practical integration ideas. (DOCX)

S1 File Interview transcripts. (DOCX)
