# Supplementary material for: Integrating recommendations for transgender and gender non-conforming perinatal care in the NHS: A qualitative exploration of healthcare professionals’ views
Source: PLOS Glob Public Health. 2026 Jan 7;6(1):e0005684. doi: 10.1371/journal.pgph.0005684 (PMC12788185; doi:10.1371/journal.pgph.0005684)
Supplement: S1 Appendix — (DOCX) [file pgph.0005684.s001.docx]

| Title | Authors | Date | Country | Aim | Stated Method | Description of Sample | Main Findings | Recommendations |
| --- | --- | --- | --- | --- | --- | --- | --- | --- |
| Providing gender affirming and inclusive care to transgender men experiencing pregnancy | Hillary Chu, Lee Kirby, Ashley Booth, Meredith Klepper, Athena D.F. Sherman, Kelly M. Bower, Erin M. Wright | 2022 | United States | To evaluate the impact of training on nurses’ knowledge, skills, and attitudes when caring for pregnant transgender men. | Self-administered surveys prior to and following training. | 55 nurses completed the pre-test survey. 23 nurses completed the post-test survey. | Training improved nurse's self-reported knowledge and skills to provide gender affirming care. | Unit-based training for care providers. |
| Creating change with families: Reflections and recommendations for the care of gender diverse and LGBTQIA+ individuals and their families throughout pregnancy and birth | Matilda Copeland, Julie Tucker, Annette Briley | 2023 | Australia | To understand transgender and non-binary people’s experience of perinatal care, and how care can be made more inclusive. | Case study reports. | 2 transgender and non-binary people who received perinatal care. | Service users appreciated continuity of carer and documentation of their pronouns. They found the perinatal care environment alienating. | Training for care providers. Gender neutral language. Gender neutral pregnancy resources. |
| Undergoing pregnancy and childbirth as trans masculine in Sweden: experiencing and dealing with structural discrimination, gender norms and microaggressions in antenatal care, delivery, and gender clinics | Felicitas Falck, Louise Frisén, Cecilia Dhejne, Gabriela Armuand | 2020 | Sweden | To understand how transgender people experience perinatal care encounters. | Face to face interviews and thematic content analysis. | 12 trans masculine individuals who had accessed perinatal care. | Care providers viewed pregnancy and a masculine gender identity as incompatible. Service users had to advocate for themselves to receive appropriate care. Quality of care was inconsistent. | Gender clinics should support service users during pregnancy. Perinatal care providers should ensure they use appropriate language and discuss birth preferences. Training for care providers to support chest feeding. |
| From erasure to opportunity: a qualitative study of the experiences of transgender men around pregnancy and recommendations for providers’ | Alexis Hoffkling, Juno Obedin-Maliver, Jae Sevelius | 2017 | United States | To understand the needs of transgender men who have given birth. | Interviews conducted online using video and/or audio. | 10 transgender men; interviews recorded, transcribed, and systematically coded | Participants had a diverse range of experiences. Structural barriers disempowered transgender service users. Affirming care improved experience. | Improved clinical visibility for transgender service users. Information on reproduction for transgender people: pre-transition, pre-conception, antenatal, and postpartum periods. |
| Perinatal considerations for care of transgender and nonbinary people: a narrative review | Megan McCracken, Gene DeHaan, and Juno Obedin-Maliver | 2022 | United States | To provide guidance on including all genders in obstetric care. | Narrative review. | Articles published on PubMed found using key terms | Gendered language excludes transgender service users. The care environment can be gendered and increase dysphoria. | Individualised care provision. Mental health support in the postnatal period. |
| Providing Inclusive Midwifery Care for 2SLGBTQQIA+ People: Supporting Inclusion in Ontario’s Midwifery Education Program | Melanie Murdock | 2023 | Canada | To define inclusive midwifery care and explore the experiences of midwives and their training in inclusive care provision. | Semi-structured interviews. | 11 midwives | Midwives were aware of the importance of appropriate language, environment, documents, websites, and individualised care provision. | Exposure to transgender service-users as a part of midwifery training. Improved inclusivity training for students taught as standard. |
| Experiences of transgender men in seeking gynaecological and reproductive health care: a qualitative systematic review’ | Julia D. Sbragia, Beth Vottero | 2020 | United States | To evaluate the experience of transgender people accessing perinatal care. | Systematic review. | 26 studies comprising 874 participants and yielding 86 findings | Perinatal care caters to cis women. Transgender men adapt behaviour due to discrimination. | Individualised care provision. Improved cultural competency education for care providers and students. Inclusive environment, documents, and electronic records. |
| Providing Patient-Centered Perinatal Care for Transgender Men and Gender-Diverse Individuals’ | Monica Hahn, Neal Sheran, Shannon Weber, Deborah Cohan, Juno Obedin-Maliver | 2019 | United States | To create an inclusive, compassionate, and equitable health care experience | Case report. | 20-year-old transgender man who accessed perinatal care | Gender affirming care requires system and personal changes. | Staff training. Gender appropriate language and documentation. |
