# Supplementary material for: Integrating recommendations for transgender and gender non-conforming perinatal care in the NHS: A qualitative exploration of healthcare professionals’ views
Source: PLOS Glob Public Health. 2026 Jan 7;6(1):e0005684. doi: 10.1371/journal.pgph.0005684 (PMC12788185; doi:10.1371/journal.pgph.0005684)
Supplement: S2 Appendix — (DOCX) [file pgph.0005684.s002.docx]

|  | | Copeland *et al.* 2023 | Sbragia and Vottero 2020 | Hoffkling *et al.* 2017 | McCracken *et al.* 2022 | Chu *et al.* 2022 | Murdock 2023 | Hahn *et al.* 2019 | Falck *et al.* 2020 |
| --- | --- | --- | --- | --- | --- | --- | --- | --- | --- |
| Education | Training during education on transgender care provision |  | ✔ |  |  |  | ✔ |  |  |
|  | Training as a part of continued professional development on transgender care provision |  | ✔ |  |  | ✔ | ✔ |  |  |
|  | Training during education on cultural competency and sexuality and gender diversity | ✔ | ✔ | ✔ |  |  | ✔ |  |  |
|  | Training as a part of continued professional development on cultural competency and sexuality and gender diversity | ✔ | ✔ | ✔ |  | ✔ | ✔ | ✔ |  |
|  | Gender-inclusive practice as a core competency for students |  |  |  |  |  | ✔ |  |  |
|  | Group discussions and reflections as part of education |  |  |  |  | ✔ |  |  |  |
| Language and Literature | Ask service users their pronouns | ✔ |  | ✔ | ✔ | ✔ | ✔ | ✔ | ✔ |
|  | Ask service users their preferred terms for their anatomy and bodily function |  |  | ✔ | ✔ | ✔ | ✔ |  | ✔ |
|  | Gender inclusive language on forms and documents |  | ✔ | ✔ |  | ✔ | ✔ |  |  |
|  | Gender inclusive service names and signage |  | ✔ | ✔ |  |  |  | ✔ |  |
|  | Gender neutral cot cards | ✔ |  |  |  |  |  |  |  |
|  | Gender inclusive service user information (e.g. pamphlets) | ✔ |  | ✔ |  | ✔ | ✔ |  |  |
| Antenatal Care | Discuss infant feeding goals and options |  |  |  | ✔ |  | ✔ | ✔ | ✔ |
|  | Gender inclusive birth preparation and parent education |  |  |  |  |  | ✔ | ✔ |  |
|  | Antenatal discussion of birth preferences and mode of birth | ✔ |  |  | ✔ |  |  |  | ✔ |
|  | Tours of birthing areas |  |  |  |  |  |  | ✔ |  |
| Labour Care | Neutral apparel for service users |  | ✔ |  | ✔ |  |  |  |  |
|  | Early analgesia in labour to reduce dysphoria if desired |  |  |  | ✔ |  |  |  |  |
| Postnatal Care | Appropriate referrals or support to recommence testosterone if desired |  |  | ✔ | ✔ |  |  | ✔ |  |
|  | Monitoring for deterioration in mental wellbeing and postnatal depression |  |  | ✔ | ✔ |  |  | ✔ |  |
| Documentation and EMR | Gender inclusive EMR systems (allow sex to differ from gender, allow male patients admission to labour ward) | ✔ | ✔ | ✔ |  | ✔ | ✔ |  | ✔ |
|  | Documentation of correct pronouns | ✔ |  | ✔ | ✔ |  | ✔ | ✔ |  |
| Environment | Gender inclusive bathroom facilities |  |  | ✔ |  |  |  | ✔ |  |
|  | Single rooms for hospital admissions |  |  |  | ✔ |  |  |  |  |
|  | Gender inclusive posters and decoration |  | ✔ | ✔ |  | ✔ | ✔ | ✔ |  |
| Institutional Organisation | Acceptance of gender-diverse pregnancies / "Zero tolerance" for transphobia |  | ✔ | ✔ |  | ✔ | ✔ |  |  |
|  | Communication between care providers and services including handovers | ✔ |  | ✔ | ✔ |  |  | ✔ |  |
|  | Continuity of Carer | ✔ |  |  |  |  |  |  | ✔ |
|  | Care provision by an LGBTQ+ care provider |  |  |  | ✔ |  |  |  |  |
|  | Co-operation between care providers and transgender support groups and services |  |  |  |  |  | ✔ | ✔ | ✔ |
