# Supplementary material for: Integrating recommendations for transgender and gender non-conforming perinatal care in the NHS: A qualitative exploration of healthcare professionals’ views
Source: PLOS Glob Public Health. 2026 Jan 7;6(1):e0005684. doi: 10.1371/journal.pgph.0005684 (PMC12788185; doi:10.1371/journal.pgph.0005684)
Supplement: S3 Appendix — (DOCX) [file pgph.0005684.s003.docx]

**Study Title:** Understanding and overcoming the barriers to safe and effective care for transgender and gender non-conforming NHS users: A qualitative exploration of healthcare professionals’ views.

**Consent**

The aim of this study is to explore healthcare professionals’ experiences, knowledge, and attitudes to care provision for transgender and gender non-conforming pregnant people.

Have you read the information sheet?

Do you have any questions?

Are you happy for me to record audio and visual? You can turn your camera off for audio only.

**Demographic Questions**

What is your professional role?

In which region do you work?

How many years have you been [your current role]?

What is your gender identity?

**Background Questions**

What experience have you had working with transgender and gender non-conforming pregnant people?

What do you feel are the priorities as a healthcare professional when caring for transgender pregnant people?

What support do healthcare professionals have to support this patient group?

Can you describe a time you feel good care has been provided for this patient group?

Can you describe a time you feel care has been inadequate?

**Definition Questions**

Could you describe your philosophy of care, or your care priorities as [your current role]?

In an ideal world, without any constrictions on your practice or work environment, would your philosophy of care be different?

Could you describe your organisation’s philosophy of care or care priorities?

What tools, resources and information are available to you to help you provide care?

In an ideal world, what tools, resources, or information would you like to have accessible to you?

How would you define safe care?

In an ideal world, without any constrictions on your practice or work environment, would you define safe care differently?

**Literature Review Questions**

I’m going to present you with some recommendations for transgender perinatal care, in no particular order, and ask you some questions about them.

**Recommendation 1**: Training as a part of continued professional development on cultural competency and sexuality and gender diversity.

1) What do you think of this recommendation?

2) How feasible is this care provision?

3) What tools, resources, or information would you need to provide this care?

4) How much does this align with current practice?

5) How could this care recommendation impact safety?

**Recommendation 2**: Ask service users their pronouns.

1) What do you think of this recommendation?

2) How feasible is this care provision?

3) What tools, resources, or information would you need to provide this care?

4) How much does this align with current practice?

5) How could this care recommendation impact safety?

**Recommendation 3**: Ask service users their preferred terms for their anatomy and bodily function.

1) What do you think of this recommendation?

2) How feasible is this care provision?

3) What tools, resources, or information would you need to provide this care?

4) How much does this align with current practice?

5) How could this care recommendation impact safety?

**Recommendation 4**: Gender inclusive EMR systems (allow sex to differ from gender, allow male patients admission to labour ward).

1) What do you think of this recommendation?

2) How feasible is this care provision?

3) What tools, resources, or information would you need to provide this care?

4) How much does this align with current practice?

5) How could this care recommendation impact safety?

**Recommendation 5**: Documentation of correct pronouns.

1) What do you think of this recommendation?

2) How feasible is this care provision?

3) What tools, resources, or information would you need to provide this care?

4) How much does this align with current practice?

5) How could this care recommendation impact safety?

**Recommendation 6**: Gender inclusive posters and decoration.

1) What do you think of this recommendation?

2) How feasible is this care provision?

3) What tools, resources, or information would you need to provide this care?

4) How much does this align with current practice?

5) How could this care recommendation impact safety?

**Concluding Questions**

Did any of these recommendations surprise you?

Do you see any of these recommendations as being more important than the others?

Was there anything you expected to be included that we haven’t spoken about?
