# Supplementary material for: Integrating recommendations for transgender and gender non-conforming perinatal care in the NHS: A qualitative exploration of healthcare professionals’ views
Source: PLOS Glob Public Health. 2026 Jan 7;6(1):e0005684. doi: 10.1371/journal.pgph.0005684 (PMC12788185; doi:10.1371/journal.pgph.0005684)
Supplement: S4 Appendix — (DOCX) [file pgph.0005684.s004.docx]

| **Pronouns and Language** |
| --- |
| - Signs for doors or above beds stating name and pronouns - Pronouns on hospital wristbands alongside demographic details - Discussion of pronouns at booking or mandatory field for pronouns on booking forms - Incorporating pronouns into handovers between staff - Pronouns on demographics banners on EMR systems - Sticker on handheld notes stating pronouns - Discuss language preferences as a part of birth preference discussions |
| **Education for staff** |
| - Key terminology "pocketbook"/posters/crib sheet for staff - Clear staff leads and referral pathways - Training to include personal accounts from TGNC pregnancy service users |
| **Education for service users** |
| - Antenatal classes and information aimed at TGNC service users |
| **Gender inclusion** |
| - Non-gendered options for baby hats and cot cards - Ensuring gender expansive language (e.g. women and birthing people) |
