# Supplementary material for: Integrating recommendations for transgender and gender non-conforming perinatal care in the NHS: A qualitative exploration of healthcare professionals’ views
Source: PLOS Glob Public Health. 2026 Jan 7;6(1):e0005684. doi: 10.1371/journal.pgph.0005684 (PMC12788185; doi:10.1371/journal.pgph.0005684)
Supplement: S1 File — (DOCX) [file pgph.0005684.s005.docx]

Interview Transcripts

# Participant 1 Transcript

42m 43s

Interviewer 0:03
Thank you so much, [participant’s name].

Interviewer started transcription

Interviewer 0:05
This study is exploring healthcare professionals experiences, knowledge and attitudes towards care provision for transgender and gender nonconforming people.
Have you been given an opportunity to read the information sheet?

Participant 1 0:16
Yes, I have. Yep.

Interviewer 0:17
Amazing.
And do you have any questions at this time?

Participant 1 0:21
No.

Interviewer 0:22
OK.
And are you happy for me to record?

Participant 1 0:24
Yes.

Interviewer 0:25
Great.
Thank you.
So I'm just gonna start with some demographic questions.
What's your professional role?

Participant 1 0:32
Midwife, triage midwife.

Interviewer 0:35
And what region do you work in?

Participant 1 0:38
As in like where? In [location].

Interviewer 0:40
Yeah.
Yeah, that's fine.
And how many years have you been a midwife?

Participant 1 0:46
Six years now.

Interviewer 0:48
And how would you describe your gender identity?

Participant 1 1:01
As in, as in, I'm like female, like born female and identify as female, yeah.

Interviewer 1:08
Yeah.
Thank you.
And in your professional role, have you had an experience working with transgender or gender non conforming pregnant people?

Participant 1 1:17
No, I haven't actually.

Interviewer 1:20
And have you known about other people who have provided this care?

Participant 1 1:26
Yeah.

Interviewer 1:27
Yeah.
And it's OK, because obviously, we're all gonna have different experiences and it's so it's OK if you if you don't feel that you can answer this one.
But do you feel there's any key priorities for healthcare professionals who are gonna be working with transgender pregnant people?

Participant 1 1:45
And I don't think so.
I think we just don't have enough experience to really know very much at the moment.

Interviewer 1:54
And what do you think could support midwives or other health care professionals to gain experience?

Participant 1 2:03
I think obviously exposure and kind of going out of our way to look into what we can do to help and also research into it as well.

Interviewer 2:17
And do you feel there's much support for midwives who are providing this care?

Participant 1 2:27
It's hard to say because obviously I've never provided the care, so I don't know what is around, but I don't think so.

Interviewer 2:35
OK.
And so it wouldn't be like that there's anything clearly signposted if someone was to present?

Participant 1 2:39
No.

Interviewer 2:48
And so just talking a bit about your care as a midwife before we go on to the literature.

And again, these seem a bit vague, so it's OK.
It's as much or as little you know you're comfortable giving.
Could you describe your personal philosophy of care or your current experience and your care priorities in your current role?

Participant 1 3:02
So as in like what I do basically?

Interviewer 3:06
Yeah.

Participant 1 3:07
OK, so obviously I work in triage, so there's a lot of flow through triage. Well, mostly work in triage. I’m also obviously birth centre and sometimes labour care and labour ward.

But there's a lot of people that come through triage and so personally I really try to make it as individual as I can when I see people because I know that it can feel a lot like in and out and like, you know, people don't really care, you're seeing as a bit of a number rather than anything like that, and so I try and treat people as individuals.
I look at their like personal history and see how I can make it easier for them, and when I do meet them and then take into account obviously what they're feeling as well as the physical things that they are coming in with as well and then find out where they need to go, who they need to see and everything like that.

Interviewer 4:04
Thank you.
And in an ideal world, if there were no constraints or there were not the current constraints on your practice, do you think your philosophy of care would be any different?

Participant 1 4:18
I don't think it would.
I don't think it would be different, but I think I'd have more time to be able to treat people the way that I would want them to be treated.
And yeah, so a lot, of especially like in triage and things like that you very much like in and out, you see people and you have to say, you know, “go sit at reception, wait possibly four hours to be seen by people. I'm not gonna communicate with you during that four hours because I'm gonna be so busy seeing everybody else that I can't even update you on what's happening.”
And so I’d say, although my philosophy would probably be similar. I would be able to do it better.

Interviewer 4:57
Because you'd have more time available?

Participant 1 4:59
Because I'd have more time, yeah.

Interviewer 5:00
Yeah.
OK.
Thank you.
And how would you define safe care?

Participant 1 5:11
Safe care, I'd say… Basically, doing everything that you need to do for what somebody is presenting with, I know this is very triage based.
Sorry, I know it's rather than anything else but…

Interviewer 5:27
That's OK.

Participant 1 5:31
Basically, making sure you've got all of the physical things there to be able to assess somebody properly being able to spend the time talking with them, get a full history.
Get like a physical history.
Being able to examine people, being able to have all the equipment that you need in order to do your job. Basically, and then having like amount of stuff that you need and like the medical professionals that you need, easy to be able to reach.

Interviewer 6:03
Umm so about equipment, personnel, and communication?

Participant 1 6:07
Yes.

Interviewer 6:08
Yeah.
And in an ideal world, so if we didn't have those constructions that we talked about earlier, would you define safe care any differently?
So if you had more time?

Participant 1 6:21
Umm, no, I'd say safe care is just what you ideally would be giving.
And no, I I'd say it's still about the same, but you would just have more time and more people around to be able to actually do it rather than say that that's what you want to do but not be able to do it.

Interviewer 6:40
Yeah, completely fair enough.
And what you spoke about the people and tools. What tools and resources and information are available to you to help you provide this care and this safe care?

Participant 1 6:55
So as in so, you've got your physical stuff.
Obviously, all of the equipment that you need, but we rely in triage quite heavily on the guidelines as well, so being able to access those on the Intranet is really important and having like the midwife in charge and things like that available to be able to contact if you have any questions or you need any support providing care for anyone.

Interviewer 7:21
OK, thank you.
Umm, so I'm going to talk about some recommendations for pregnancy care for trans people.
They're in no particular order of importance, but they all came up frequently in a literature review, and I'm just gonna ask you some questions about what you think of them.
And it's anything that springs to mind, really.

Participant 1 7:39
Yep.

Interviewer 7:39
So one of the recommendations was training as a part of continued professional development on cultural competency and sexuality, including gender diversity.
What do you think of this as a recommendation for healthcare providers?

Participant 1 7:53
I think it's good if they can provide training to basically open people's minds to what care we should be providing.

Interviewer 8:05
And do you think it would be feasible to have this training?

Participant 1 8:09
Yeah, I think so.

Interviewer 8:12
And who do you think would be the best people person to provide training of this sort of nature?

Participant 1 8:21
So I think the PDM, practice development midwives, should maybe look into like organizing a little… like include it in our study days basically and just find a member of staff that is passionate and willing to teach it and to show people how we should be caring for people.

Interviewer 8:49
So someone who has experience in a healthcare role?

Participant 1 8:52
Yes, well, actually, yeah, I guess. Yeah.
Yes in a healthcare role, but it would also be good if we made it like a study day, to have people who actually experience it in day-to-day life as well, being able to tell us their side of things and how we can help them if they need to access the care.

Interviewer 9:17
So having people who are transgender and be able to come and speak about their experiences?

Participant 1 9:19
Yeah.
I think that would be that would be good, yeah.

Interviewer 9:24
Yeah. OK, great. Thank you.
Is this something that's currently being done at all in practice?

Participant 1 9:32
No, not that I know of, no.

Interviewer 9:34
And what tools or resources do you think would be needed to initiate this training?
So we've already spoken about who the speaker would be.
Is there anything else that staff would need to facilitate the training?

Participant 1 9:51
Well, obviously you'd need the time and I guess.
I'm sure that they'd probably need, like, the funding and stuff like that to be able to pay people to do it and to pay us to come in to learn about it.
But… That's, I don't know, I think so.
I think that's it.

Interviewer 10:11
No, that's fine.
And then finally on this one, do you think that having additional training would contribute to safe care provision?

Participant 1 10:21
Yeah, I think it.
I think it would actually, yeah, because I think there's a lot of… Well, I know that there is a lot of like judgment and bias and things like that around it and so if we if people are like trained and know, it's hard to explain…
But basically I think there's a lot of avoidance in healthcare, and if you don't know how to talk to somebody or what to say to people or language to use, then you tend to avoid. And so then you worry, are you providing the same care to these people that you that and you're providing to everybody? And it should be the same across the board and if you're avoiding, then you're less likely to go in and do your checks, and you know, have conversations with people that you might be having with everybody.
So I'd say it would help provide safe care, yeah.

Interviewer 11:12
And this the judgment and bias you mentioned, is that within a healthcare setting you've experienced or general life?

Participant 1 11:20
No, I've not experienced…
Umm, not specifically to trans people, no, but you do see it even you know when I had it experience, I don't if I'm allowed to talk about, I'm not gonna mention names or anything obviously.
But like when I cared for a surrogacy lady, and she obviously then the intended parents were there and a lot of people didn't know how to broach the subject and how to have the conversations.
And so you do notice that people like, “oh, should I go in? Should I say this? Should I say that?”
And I know that it's not exactly the same, but it probably would be similar if people were caring, if you're caring for a trans person and so, yeah, I'd say I see it in situations like that, but I've not seen it specific to trans.

Interviewer 12:11
Umm, no, fair enough and it sounds like that avoidance also could be a fear of getting it wrong?

Participant 1 12:17
Yes, agreed. Yep.

Interviewer 12:19
Yeah.
OK, any more thoughts about training and professional and continued professional development?

Participant 1 12:30
I don't think so, no.

Interviewer 12:33
Great.
And so if that's OK, I'll move on to another recommendation.
And this one is asking service users their pronouns.
What do you think of that as a care recommendation?

Participant 1 12:46
I think it would be good if you did it like a booking appointment and so there should be like a… I have thought about this, there should be like a… you know on the like the green sheet, the front, the front page, it should be like has asked and then like specify what the pronouns are, just so that we know that we are getting it right when we're talking to people.

Interviewer 13:08
So having something documented as well for everyone else to see, and also having a standard time of when it's when it's discussed?

Participant 1 13:10
Yeah, I think so.
I think it should be included because it's important to know that we're not misgendering people.

Interviewer 13:24
Umm.
And do you think it would be feasible for healthcare workers to ask about people's pronouns?

Participant 1 13:32
Yes, I think so.

Interviewer 13:34
Umm.
And would any additional tools or resources or information be needed for healthcare professionals to ask these questions?

Participant 1 13:43
Umm, I just.
I don't think so.
I think it's quite an easy question to ask and then I think the only tool that you would need is maybe changing up the notes slightly, just that there's like a little bit that you fill in to say that you've asked the question.

Interviewer 13:59
And so you feel that the majority of staff will have the background knowledge needed to ask this question?

Participant 1 14:06
You'd hope so.

Interviewer 14:07
Yeah.

Participant 1 14:09
Maybe, maybe not in some cases, but I think it's a very easy question to ask.
And if you don't, you're not able to ask the question then I don't know…
It's tricky.

Interviewer 14:20
Can you think of anything that would make people more or less likely to be able to ask questions such as what someone's pronouns are?

Participant 1 14:30
Well the only thing that's means to mind is obviously language barriers and things like that, that then you should be doing appointments with like interpreters and things like that anyway.
So I think you should, it's just a very easy, simple quick question to ask and I think everybody should be able to ask it.

Interviewer 14:52
Thank you and is asking pronouns something that's currently being done within practice that you've seen.

Participant 1 14:59
Not that I know.
I've no not that I've seen.

Interviewer 15:01
OK.
And do you feel that asking pronouns would contribute or make any difference in terms of the safety of care provision?

Participant 1 15:11
The question itself, of asking?

Interviewer 15:13
Yeah.
Yeah, asking pronouns.

Participant 1 15:16
No, I don't think so, no.

Interviewer 15:17
Yeah.
OK, that's fine.
And so going on from asking about pronouns, any other comments about that one before I move on?

Participant 1 15:27
No, I don't think so.

Interviewer 15:28
OK.
One of the other recommendations was asking service users their preferred terms for their anatomy and bodily function.
So some examples of this would be, rather than breastfeeding, some people might prefer the term chest feeding and instead of words such as vaginal birth or vagina, they might prefer pelvic birth or frontal hole for their own vagina.
So using slightly different language, what do you think of this as a recommendation?

Participant 1 15:57
I think in general I think we should be personalizing care anyway.
And so I think if we are asking pronouns and what makes people comfortable, then we should know what language we should be using with people.
And so if we are and asking, you know, pronouns, do you do you prefer like this language or what would you prefer us to ask? If we're personalizing the care anyway we should, we should know that. If that does that make sense?
Am I making sense? Like if you, if you're asking, do you identify as female, male, then we should be able to then tailor our language to what they want to, you know, be identified as.

Interviewer 16:48
Umm.
So when do you feel would be an appropriate time to ask these questions or clarify language that's preferred?

Participant 1 16:57
As early as possible, I would say because if you're going you, you know, we see people throughout their whole pregnancy.
And so if we're using the wrong language, so we need to know what kind of language to use in the beginning so that as we go throughout the pregnancy, we're using the appropriate language.

Interviewer 17:17
Umm, so where do you feel would be a good place for this to be documented?

Participant 1 17:25
There's nowhere specific in the notes to document, but ideally you would when asking the pronouns at booking, at the earliest possible appointment, then you could put it in the booking, but I do also know I don't work in clinic so I don't know…
Bookings are already a really long appointment and so is throwing more information at people and asking more questions gonna make it harder?
I don't know, but it should be documented, probably around the booking information or somewhere like that.

Interviewer 18:02
And how feasible do you think it would be to ask about preferred terms for anatomy?

Participant 1 18:08
I think it's. I think it would be easy to ask, yeah.

Interviewer 18:12
And do you feel that healthcare providers would need any additional tools, resources or information to ask these questions?

Participant 1 18:20
I think.
Not to ask the question, but it would be good for healthcare professionals to kind of know what language is more appropriate.
So, like almost like information on saying like chest feeding and things like that, like a almost a list of alternatives that we could use.
Does that make sense?
Yeah, I think it does.

Interviewer 18:50
So like a crib sheet?

Participant 1 18:51
Yeah, just to be like, ohh, we could call it this instead of this.
And how about we use this terminology instead of this terminology?
Because yeah, I think that could be helpful, but everybody should really know the basics.

Interviewer 19:05
Umm.
And you said before that ideally we should be individualizing all care. How much does, and I know it's different if you haven't had much experience caring for trans people, how much do you feel that it that aligns with what's currently going on in practice? And have you heard these preferred terms for anatomy in practice at all?

Participant 1 19:30
I've heard them discussed, but I've never heard them at, like, used with somebody because obviously I've never had the experience caring for a trans person and so I've definitely heard them discussed and, you know, seen, you know, the like, people talk about them, but it's never been used in in front of me that I know of.

Interviewer 19:52
And what people usually say in these discussions or what's the tone of them?

Participant 1 19:58
So it varies.
I think initially when there was discussion over changing the term like for example breastfeeding to chest feeding in general, a lot of people were like, no, we shouldn't change it.
And but again, I have, the people that I transfer myself with, they're very much like, but we should be providing individual care anyway so if somebody's identifying as female, then breastfeeding most likely is not going to be an offensive term.
However, if you know you're caring for a trans individual, then you know to tailor your language anyway, and so we should be providing that care, in an ideal world, and I'd hope that a lot of my colleagues do try to provide individualized care.
But I do know that there's people out there that that definitely find it…
I've heard people say that it's offensive and that it takes away from, you know, women having babies and things like that. And so you know that there's those kind of opinions out there as well. But so far, I've not really encountered any really strong opinions against it.

Interviewer 21:13
And are these discussions at work or online or outside of the work environment?

Participant 1 21:21
Although, I was specifically talking about at work, but then there are, like the more kind of strong minded things that I've been hearing I've seen online as well.

Interviewer 21:33
OK.
Is that in healthcare settings like healthcare groups online or general?

Participant 1 21:37
Not really, no, just general, just general.

Interviewer 21:39
OK.
Thank you and how… do you feel that asking preferred terms for anatomy and bodily function would make any difference to the safety of care provision?

Participant 1 21:57
Thinking about it, I'd say…
It probably would in regards to I.
I wonder if the individual would be more comfortable talking to us if we are respecting their terminology and things like that.
And so I think possibly they'd be more likely to access care I'd think, but I'm not 100% sure.

Interviewer 22:25
So it could contribute towards their psychological safety?

Participant 1 22:30
Yeah.
And physically as well, because if they are having, you know, physical symptoms of things, but they're nervous about contacting because they're not sure, you know, how we would react to different terminology or like, you know, how to express themselves and tell us what's wrong then obviously, physically things can go wrong as well and so.

I'd say by us in encouraging the use generally of people knowing what terminology people are comfortable with, then it should help them access care, basically, and then we could provide safer care.

Interviewer 23:09
So improve engagement and potentially relationship building as well?

Participant 1 23:16
Yeah, definitely, yeah.

Interviewer 23:18
Yeah.
OK. And any other thoughts about preferred terms for anatomy?

Participant 1 23:24
No, I don't think so, no.

Interviewer 23:27
OK, so moving on, this one is kind of something we've already spoken about quite a bit, one of the other very frequent recommendations was documenting correct pronouns.
So we've kind of covered this when asking about pronouns, which is fantastic, but just to reiterate, what do you think of this as a recommendation, to document pronouns?

Participant 1 23:44
Umm.
I think it's a good idea to document pronouns because you don't want to misgender people.
And if you're, I know in triage because we see so many people, to be able to have it at a glance and be able to, like, find a page and be like, OK, this person identifies as this and this, then it would definitely be easier.

Interviewer 24:10
And I think we've mainly said it already, but how feasible do you feel this would be?

Participant 1 24:16
Yeah, I think it would be easy to do, yeah.

Interviewer 24:19
OK. And do you feel that documentation of pronouns would contribute towards the safety of care provision?

Participant 1 24:34
I'd say… Well, I don't know because I hope that you would be safely providing care regardless of pronouns, but I guess the same as before as long as you, because if you've got the information there then people will be more likely to want to engage in services if you're gendering them correctly.

Interviewer 25:03
OK.
And any other thoughts about that one?

Participant 1 25:08
Yeah, I don't think so.

Interviewer 25:10
OK.
So kind of as a follow on from all of that and another recommendation was to improve the gender inclusivity of electronic medical record systems.
So some examples of this is most electronic medical systems don't allow sex to differ from gender, but they also don't allow male patients to have a pregnancy or to be admitted to the labour ward.
And so the recommendation would be to improve the gender inclusivity of the medical record systems.

What do you think of this?

Participant 1 25:46
I think it needs to be done if it's, if it's not already.
I didn't know that obviously, cause I've never cared for anybody that's trans, but I think that it needs to be done, definitely.

Interviewer 25:56
Umm.
And again, this is more of an IT side of things question, but how feasible would you feel it would be for these systems to be made more inclusive?

Participant 1 26:06
Well, I think currently we're obviously going through a massive update anyway in the online systems and so it should really have been included in that.
I think… I don't know much about the IT side of things, but I think surely it should be fairly simple to be able to allow you know, male gender to be admitted onto the maternity ward and us to add babies onto their accounts and things like that.
Surely it wouldn't be that difficult to change that, but and regardless of how difficult it is, I think it needs to happen.

Interviewer 26:40
Umm and.
How much do you feel or do you feel that improving the gender inclusivity of the record systems would contribute towards safe care provision?

Participant 1 26:53
I think I think it's essential to be able to provide safe care by and be able to document like effectively, because then you're providing your safe care, aren't you?
If you're able to kind of access people's online like [electronic medical record system name], basically, if you're able to access it and you're able to do everything you need to do and document everything that's important. So even in like discharge from maternity services, if you can't do that on somebody who's male, then how will we able to effectively hand over care and give all of the information on things that you need to?
So I think it, I think it's important.

Interviewer 27:40
Absolutely.
And who do you think or how do you think would be in charge of these sorts of improvements?

Participant 1 27:52
Probably it probably needs to be discussed with, you know, like the digital midwives that they've got at the moment and then they could escalate to the people that are in charge of the actual [electronic medical record system name] database and whatever they it should be escalated to them.

Interviewer 28:10
And would any additional tools, resources or information be needed in your opinion to update these?

Participant 1 28:20
Not that I know of, but I don't know.
I'm not sure on the IT side of things, but yeah, I don't think so.

Interviewer 28:28
It's fine.
And then any more thoughts about that?

Participant 1 28:35
No, I'm just surprised by it.
I didn't know that, so yeah.

Interviewer 28:38
Umm, so this one in terms of safety was one that that you felt and please correct me if I'm wrong contributed more to physical safety?

Participant 1 28:49
Yeah, I'd say, well, both, definitely, but definitely physical as well, because if you're not, if you're not able to hand over care and give the vital information it can actually definitely cause problems down the line.

Interviewer 29:06
Yeah. OK. So I'll move on to the last recommendation which was having gender inclusive posters and environments and in hospital settings.
So this would include educational posters and also making wards less female centered.
So for example, some units only have female toilets, so rather than like impatient toilets, it will say women's only toilets and the decoration and educational posters may be tailored towards cis women who are pregnant.
And what do you think about changing some posters decorations to be more gender inclusive?

Participant 1 29:54
I think so. I think it would… It would be a good idea and I'd say, obviously it comes a lot down to like funding and stuff like that for the physical changes, I think our do our like impatient toilets, they… don't they just say… I don't know what they say, I've never looked actually.

Interviewer 30:16
They say women's only bathroom.

Participant 1 30:17
Ours do? Hmm, interesting.
Yeah, I think things like that should be changed because there's no, there's absolutely no harm in actually changing it, but it can be harmful if you don't change it.
So I'd say definitely it benefits everybody to change it.
And posters definitely as well.
I think that would be easier to do because it's not quite so like physical changing and like things like the inbuilt and like doors and things like that.
But like posters should be fairly easy and they should be available.
And yeah, I'd say, yeah, it needs to be changed.

Interviewer 30:57
And would you have any ideas about like the content of the posters?

Participant 1 31:02
So I'd say it needs to be the same kind of content that we've got already, because if that's stuff that's like tailored just to women, it's just saying that women need to know that is not right.
So if you know if you identify as a trans, as like a male then.
You still need to know this information as well if you're having a baby, and so it's the same kind of information, but it would be, you know, it's good also to have maybe information on… Like posters of like accessing care and stuff like that on there as well.
Also thinking about it, like antenatal classes and stuff like that extremely female and like orientated and always talk about like the women and all of the posters and leaflets and everything that I've ever seen are all based on, like women's birthing experience and stuff like that and so I think that needs that will need to be changed as well.

Interviewer 32:12
Umm so making sure that the any posters are educational and but also promoting access to available services?

Participant 1 32:13
Hmm.
Yes.

Interviewer 32:22
Yeah. OK.

Participant 1 32:22
Yeah, you're good at summarizing.

Interviewer 32:25
Thank you.
And anything else that you think would be important to include in any gender inclusive posters or information?

Participant 1 32:40
No, I would say… No, just the fact that there is support and things like that.
But other than that, it should… the way that I feel about it is that they should be treated the same as we treat anybody and so the information that we're giving people is the same.

You need to like make sure that everybody is aware of the same information, but in a respectful way.

Interviewer 33:10
And so the posters we've spoken about so far are all aimed at service users.
Anything that you would think would be helpful for healthcare professionals?

Participant 1 33:22
Like we talked about earlier with like the language kind of information and stuff like that, it would, it would be good if there was like a… You know, sometimes you get like posters with like a QR code that can take you to like maybe an information sheet or like a website that can give people that aren't confident providing the care the support or like ways that they can improve their practice and ways that they can provide safe care to people.
It would be good to have something like that maybe?

Interviewer 33:59
So more focused on care provision?

Participant 1 34:02
Yeah.

Interviewer 34:03
Yeah.
OK.
And sorry, I'm just thinking back on what you said. Umm.
Yes so support for staff as well As for service users.
Sorry, yeah.

Participant 1 34:16
Yeah.
No, don't worry.

Interviewer 34:19
OK.
And how feasible do you think it would be to make posters such as this for staff and for service users?

Participant 1 34:28
I think it is feasible, I think.
Surely there's some already available? You'd hope. That maybe we could bring in to our trust.

Interviewer 34:41
So the only trust in the UK with uh specific policy for transgender pregnancy is [trust name], so there is one trust where this is available and they also do have gender inclusion midwives.
So absolutely there is expertise that could be borrowed from them.

Participant 1 35:01
But that is very sad that there's only one trust that does that.
That's not good.

Interviewer 35:06
Do you think any additional tools, resources or information would be needed to create posters and create a more gender inclusive environment?

Participant 1 35:24
Well, you just mentioned about [trust name] having like specialist midwives.

Interviewer 35:31
Yeah, gender inclusion midwives.

Participant 1 35:33
Yeah, I feel like that would be a good thing to implement in all trust really.
And because then, if we've got, if you've got somebody that you know that you can email or go to or ask questions, then I think that that could improve, it could improve like patients experiences and staff confidence as well.
And so not even just in the case where trans people, but anybody. Really.

Yeah, we should think about introducing that.

Interviewer 36:05
So having a specific midwives who don't necessarily provide all the care but can be a support service for other staff and people?

Participant 1 36:15
Exactly, yeah.

Interviewer 36:19
And do you feel that improved general inclusion in environments or in terms of posters would make any difference to the safety of care provision?

Participant 1 36:36
I think again, it's just in terms of making people more likely to access care and making sure that staff are providing the same quality of care to everybody because as much as we would like to say that everybody would provide the same level of care, you know that staff in some situations find when they're uncomfortable or not confident, they're less likely to. And we know that they're less likely to provide the same quality of care that they would for other people and so.
And other than like, I think that's the main thing, yeah.

Interviewer 37:13
Umm.
And can you think of any specific barriers for these specific staff?
So any barriers for these staff that is holding them back from seeking this education or experience?

Participant 1 37:29
No, I think a lot of the time it's just their own… Beliefs and stubbornness and stuff like that, that sometimes get in the way and maybe lack of resources as well.
So some people obviously are much more likely to want to better their practice, you'd hope, and so lack of… not knowing where to look for resources or not… Not knowing like who to go to for advice of the things like that, then that can be a barrier.

Interviewer 38:00
And is there a type of person that you feel is more likely to have this beliefs and stubbornness?

Participant 1 38:10
Umm from what I've seen it's usually and I don't wanna be like judgy with things like this, because I know everybody is completely different.
But you tend to see it slightly more with like… I don't want to judge people just specifically off of like, age and things but you tend to see it more.

Interviewer 39:40
I can't hear you anymore.

Participant 1 39:57
You hear me now?

Interviewer 39:58
Yes, sorry about that.

Participant 1 40:00
Perfect.

Interviewer 40:01
I'm not sure what happened and so I think I got the gist of that last one.

Participant 1 40:02
That's OK.

Interviewer 40:06
Umm.
And let me just stop and check.
OK. So any other thoughts about gender inclusive posters or decoration?

Participant 1 40:19
No, not really. I think we should definitely have them.

Interviewer 40:23
Umm and OK, so that is all the recommendations that came up most frequently. Do any of those surprise you?

Participant 1 40:34
I think it surprised me that [trust name] is the only trust that that's got like guidance and stuff like that on it and it also really surprised me that you've can't like add male patients onto like labour ward and things like that.
I didn't know any of that. And that you can't like add babies and things like that to the [electronic medical record system name] as well.
I think that's not good, and I'm surprised by that.

Interviewer 41:05
And is there anything that hasn't come up in the literature that you would have thought was an important priority for care provision for this patient group?

Participant 1 41:21
I don't… I don't know that I can think of.
I don't think I'm sure there is, but I not.
I can think of off the top of my head.

Interviewer 41:28
That's OK.
And then, is there anything of the six that you see as being more important than the others?
And I can list them again if you need.
Or anything that stood out?

Participant 1 41:44
I think the physical safety of not being… like the whole [electronic medical record system name] thing again and not being able to add like information on to a male profile is it's like it's stood out to me a little bit as that could be very dangerous.
But it's all really important in general, everything being more inclusive and having you know, resources and being able to treat people how they should be treated, it's all important.

Interviewer 42:17
Thank you, [participant’s name] and any other comments, concerns anything at all?

Participant 1 42:31
No, but I'm glad that you're doing this because it's clearly needed.

Interviewer 42:34
Thank you.
And so if it's OK with your end the recording now.

Participant 1 42:40
Yep.

Interviewer 42:41
OK.

Interviewer stopped transcription

# Participant 2 Transcript

26m 51s

Interviewer 0:03
OK, so that should have started now.

Interviewer started transcription

Interviewer 0:06
So this study is exploring healthcare professionals’ experiences, knowledge and attitudes towards care provision for transgender and gender nonconforming pregnant people.

Participant 2 0:17
Right.

Interviewer 0:17
So firstly, have you had a chance to read the information sheet, and do you have any questions about it?

Participant 2 0:24
If I'm honest, I haven't had a chance.

Interviewer 0:31
Do you want me to run you through it now, or would you rather or?

Participant 2 0:34
And yeah, you can run through it briefly.

Interviewer 0:35
OK, so is the study of organised by St. George's ethical approval through St. George's Hospital as part of my master's dissertation.
And I'm interviewing midwives regarding what the literature says we should be doing for transgender and gender non-conforming pregnant people.
Anything that we speak about, I will then.
Make a transcript of and then delete the original recording.
So your face and name won't be in the study itself and I will then, once I've interviewed lots of midwives and healthcare professionals, I’ll also interview some what we've called “key informants”.
So either people who are trans and have used NHS services or midwifery managers, anything like that, which means that they would have a specific insight towards if care recommendations are appropriate or if care provision is feasible.

Participant 2 1:32
OK, perfect.

Interviewer 1:33
And are you happy for me to be recording you?

Participant 2 1:38
Yes.

Interviewer 1:38
Yes.
OK, amazing.
So what is your professional role?

Participant 2 1:45
I'm a midwife at [hospital name].

Interviewer 1:47
So that's where you work, and how long have you been in this role?

Participant 2 1:58
11 years but not 11 years. Not at this hospital, but in the same position I was at [hospital trust] initially, and then just being here at this hospital for nearly three years.

Interviewer 2:05.
Amazing and.
And what's your gender identity?

Participant 2 2:24
Umm.. woman, female.

Interviewer 2:33
And have you had any experience caring for transgender pregnant people?

Participant 2 2:38
No.

Interviewer 2:39
No.
And do you feel like there's any specific priorities as a healthcare professional when you're caring for transgender pregnant people?

Participant 2 2:49
I think it's the same that would apply to caring for anybody in that you have to be empathetic to their individual beliefs and position and tailor the kid to their specific needs and just be sensitive and kind.

Interviewer 2:50
Thank you.
And do you feel that healthcare professionals have support caring for this patient group and separately, need support caring for this patient group?

Participant 2 3:25
I think there's a lack of information and exposure as to the specific and individual needs.

Interviewer 3:26
And have there been, although you haven't provided care specifically, have there been any transgender patients in your hospital or where you've been working?

Participant 2 3:46
Yes.

Interviewer 3:47
It's OK if you can't, but can you describe to any time there's been good care or inadequate care for them?

Participant 2 3:56
I can't really because it wasn't something that I was involved with, but I know the intention would have been umm to obviously be particularly sensitive to the needs of those people because.
They would have.

They would have fallen out outside of what people are used to.

Interviewer 4:19
OK, so from the literature review that I've done there, we've chosen 6 recommendations based on the frequency of their appearance within the literature.
So I'm going to present the recommendation to you and we can discuss it a bit and we can just talk about how feasible it is for healthcare professionals to do and what you think of it as a recommendation.

Participant 2 4:44
OK.

Interviewer 4:46
So there's no particular order.
One of the recommendations was training as a part of continued professional development on cultural competency and sexual and gender diversity.

Participant 2 4:59
Mm-hmm.

Interviewer 4:59
What would you think of this?

Participant 2 5:01
Well, I think it's already been incorporated into the training and as part of you know, kind of embracing different people and showing a level of tolerance and acceptance.
I don't think it's specifically dealt with, within our training, but it's definitely been added to existing training.

Participant 2 5:33
I think that it could be specifically identified with umm, particular needs highlighted because they are very niche and are not comparable with the needs specific, you know which are broader, I suppose, when you're looking at other minority groupings.

Interviewer 6:02
And do you think it would be, how feasible do you feel it would be to have this training?

Participant 2 6:08
Yeah, I think any training that enhances.
Cared for a patient is useful and necessary.

Interviewer 6:14
And what tools, resources or information would organisations need to provide this training?

Participant 2 6:31
Well, I think you know the…
What comes out of research with regards to where there are shortfalls, according to interviews with that particular group and then also what tools? Just input from the particular groups concerned.

Interviewer 6:35
How would increased training contribute to safe care provision, or would it contribute to safe care provision?

Participant 2 7:09

Well, it always would, you know, because if you're training people, you are improving competency and enhancing the ability to care for particular patients.

So it would definitely improve care.

Interviewer 7:23
Any other thoughts about that recommendation in general?

Participant 2 7:35
I think that it should be universally incorporated into training and specifically identify the needs of particular, of these particular patients.

Interviewer 7:35
Thank you.
So one of the other recommendations from the literature was asking pregnant people and service users their pronouns.
What would you think of that as a part of your practice?

Participant 2 8:05
It's something that I personally and just because of being from a different generation find it quite difficult to adjust to.
But I think as it becomes more usable in, in normal society, secular society, I think it is something that will be expected in terms of people wanting to be addressed in a specific way.
So I think that healthcare, just like any other group, would have to conform with that.

Interviewer 8:48
And I think you've touched on this a little bit with that answer.
How feasible do you think it would be for, for you and other midwives or health care professionals to be asking service users their pronouns?

Participant 2 9:00
I think for younger people it just comes naturally and it's something that they find it easy to incorporate within vocabulary, etcetera.
I think it would require a bit more effort from people who are not used to it and older people, but I think that if your primary goal is to care for people in the way that they need to be cared for, then you have to make that effort.

Interviewer 9:14
And.
What tools, resources or information do you feel would be needed to ask service users about their pronouns?

Participant 2 9:45
Well, it could be incorporated into booking forms.

You know, into booklets, etcetera.
So and healthcare providers could be encouraged to look at that as part of any other basic information that they would before taking on the care of that patient. It could be also incorporated into handovers and into SBARs et cetera.

Interviewer 10:15
And is this done at all already where you work?
Is there any documentation of pronouns?

Participant 2 10:19
No.

Interviewer 10:21
No.
OK.
And do you feel that this would this recommendation make any impact on the safety of care provision?

Participant 2 10:33
Umm I think in terms of encouraging a relationship between the care provider and the patient, yes, you know because that.

A good relationship between the two we know and enhances care and improves outcome.

So if people feel respected, then yes, that definitely would improve care.

Interviewer 10:53
OK.
Thank you.
And then continuing on from that recommendation, one of the other things that came up frequently was asking pregnant people their preferred terms for their anatomy and bodily function.
So example of this could be an individual preferring the word chest feeding rather than breastfeeding.
And what would you think of this in as a recommendation?

Participant 2 11:26
Well, I think it all goes back to the same thing.

I think it's something that's very unfamiliar to me because I haven't grown up with this, but I think that it goes back to the same basis on which one would care for anybody and it's looking at the needs and the requirements of that specific patient and trying to be accommodating and tolerant.

Interviewer 11:45
Do you feel like it would be feasible to ask service users their preferred terms for their anatomy and bodily functions?

Participant 2 12:09
I think it would.

I don't know how appropriate it would be to do so verbally when taking on the care, but I think it also could be incorporated into the booking paperwork and highlighted if it's something that differs from what people usually say or use.

I mean, we often we often get, you know, people coming with and I'm sure you've seen it yourself, you know, a specific requirements in terms of how they want to birth and certain phrases used or not used, et cetera.

So along those same lines.

Interviewer 12:49
What would you say like those phrases usually are, that we're a bit more familiar with?

Participant 2 12:59
So surges as opposed to contractions, etcetera.

Interviewer 13:00
OK, perfect.
So do you feel that asking service users their preferred times for anatomy would contribute or makes any difference in safe care provision?

Participant 2 13:20
Well, just in that it improves the relationship between care provider and patient.

Interviewer 13:20
OK.
Umm.
And then any other comments about those, those two kind of together, so asking about pronouns and asking about preferred terms for anatomy?

Participant 2 13:37
And no, not really.
Just that, umm, I think the best place to start is in the booking documentation.

Interviewer 13:39
Umm.
So kind of alludes to importance of having continuity.
Potentially?

Participant 2 13:54
Yeah.

And I think that those specific groups, because they’re a minority at the moment would be probably put under specific team with a specific consultant midwife.

Interviewer 13:56
No, that's a really interesting point as well.
So then yes, I'm here with specialized knowledge in that area. Potentially.

Participant 2 14:20
Yeah, definitely.
And with the team that could provide continuity.

Interviewer 14:23
And do you have continuity usually where you work?

Participant 2 14:31
The continuity? Yeah, it quite is quite good with midwifery-led patients.

And then with the consultant patients, then they have continuity with the consultant.

And even if it's just the first time, they may meet the midwives and midwifery-led patients have a team of midwives who've cared for them antenatally and they work on labour ward as well.
For those specific patients.

Interviewer 15:06
OK.
Umm, so then the 4th recommendation in this list, not in any order is to make the electronic medical record systems more gender inclusive.
So two examples of this is allowing someone’s sex to differ from their gender and some electronic medical record systems don't allow male patients to be admitted to female areas such as the labour ward.
And yeah.

Participant 2 15:38
Yeah, ours doesn't either.

Interviewer 15:39
And what do you think in general of this recommendation so more inclusive electronic medical record systems?

Participant 2 15:50
I think you'd have to get people to understand it before you got the computers to understand it.
You know, I think it's quite complicated because the computers are basically taught by people.
So yeah, when once people are used to using it and understand it, then I suppose it's easier to incorporate it into because once the errors within the computer documentation, that's a nightmare.
So once you know to expect people to incorporate these things before they themselves really understand or appreciate, it would be difficult.

Interviewer 16:21
And so again, that kind of leads into it.
But how feasible do you think it would be to improve the system in this way?

Participant 2 16:40
And I think it would be, but not within in the immediate future, I think.
People's appreciation and understanding would have to be improved first.

Interviewer 16:48
What tools and resources and information would we need prior to or in order to do this and to improve the computer systems?

Participant 2 17:05
Well, IT specialist I suppose.
People who would have an input into the programming.

Interviewer 17:08
And do you feel that changing the electronic medical record systems in this way would contribute towards safe care provision?

Participant 2 17:24
Umm it's not something that the patients actually even see.
So I think it would have a negligible impact on the patient care.

Interviewer 17:29
Umm.
And then kind of tying some of the things together.
So one of the recommendations obviously being asking service users about pronouns and then gender inclusive electronic medical record systems. One of the other recommendations that came up frequently in the literature was documentation of the correct pronouns for people, and what would you think of this as part of your practice?

Participant 2 17:58
Umm.
Yes.
So that could all come with part of the training and as people kind of become used to this process and as the number of service users from this particular group increases, then I think that yes that would be incorporated into documentation and it should be.

Interviewer 18:02
How feasible do you feel is at the moment for that to be included?

Participant 2 18:33
No.
Well, I think it's completely I.
I think once people are educated and used to it, then it's feasible because you know you free text, the documentation and you can do it with the correct pronouns or the incorrect pronouns.
You know, just as easy, you just need to be educated and willing.

Interviewer 18:49
And do you feel that documentation of correct pronouns would impact the safety of care provision?

Participant 2 19:08
It would improve, umm you know, they don't really see the documentation, so it wouldn't really have a direct impact on the patient, but it would perhaps impact how service providers are thinking about particular patients.

And therefore, you know you can't switch from one thing to the other, so your documentation would reflect the way that you caring.

Interviewer 19:32
Any other thoughts or comments about that one? So documentation of correct pronouns.

Participant 2 19:45
No, it just that it would be consistent with how you are caring for the patient.

Interviewer 19:45
And then the last recommendation that came up was gender inclusive posters and decorations in outpatient and inpatient environments in maternity, what do you think of this?

Participant 2 20:07
So I think it's something that will become more and more.

Prevalent as.

Hospitals in general start to feel, you know, that the wider population is more comfortable with it.
I think you wouldn't want to.
I mean, it's difficult because on the one hand, you want to make those patients feel included, but on the other hand?
Other people are not tolerant of it.
That doesn't mean that you shouldn't encourage tolerance.
You 100% should.

But it's something that needs to be done along with education and.
And.
General tolerance within the wider community.
Patient community I think as healthcare providers, you obligated to be tolerant and caring for everybody.
As far as the patient community is concerned, that is a much longer-term process.

Interviewer 21:20

So do you feel that changing posters and decorations would be a feasible form of care, provision or recommendation?

Participant 2 21:36
I think it would be, but you know, it sounds so strange saying this, but it's the same as this religion.
Unfortunately, sometimes outward kind of.
Reflections of what you believe can be also antagonistic, so it's… which shouldn't be the case.
Everybody should be able to, you know, say what they believe and who they are openly without that antagonizing anybody.

But that isn't the case unfortunately, so I think it needs to be worked on by specific groups whose sole aim is to try and make incorporation and tolerance part of the system.

Interviewer 22:08
Is there a focus in your hospital on making sure posters and decorations are gender inclusive?

Participant 2 22:37
No.

Interviewer 22:42
I think we've again to kind of touched on this in terms of antagonism potentially, how do you feel that gender inclusive posters and decorations would it contribute to provision of safe care?

Participant 2 22:58
I don't know if it impacts the type of care, not really.
I mean, it may be.

Through osmosis, kind of enters people, psyche and ability to be more, more tolerant and accepting.
But I think it wouldn't impact the safety of care.

Interviewer 23:15
Any other thoughts about gender inclusion in the posters and decoration?

Participant 2 23:27
Well, I think it's acts to subliminally enhance the tolerance level of people you know by just seeing the images you know around and being able to appreciate them and accept them.

Interviewer 23:47
And so I've spoken about 6 recommendations with you, which was training, asking about pronouns, asking about preferred terms, gender inclusive systems, documentation of pronouns, and gender inclusive posts and decorations.

Is there any of those that you see as being more important than the others?

Participant 2 24:12
Training I think would be the most important.

Interviewer 24:12
And are there any of those that the surprise you that you wouldn't have thought of as being something that's often recommended?

Participant 2 24:24
I suppose the posters.

Interviewer 24:25
And any reason for that? Or have we covered it already?

Participant 2 24:36
I just don't I just wouldn't have thought that that would be a priority for the specific group.
But you know, on reflection it probably would be so, but initially it wouldn't be something that I would have thought of as a priority.

Interviewer 24:54
OK.
Thank you.
And is there anything that hasn't been included in those recommendations that you would have seen as a priority in terms of care provision for this patient group?

Participant 2 25:08
You know, not having cared for people from this group, I don't really understand what the individual and specific needs are.
Because I think that the people would present very, you know, differently in terms of where they are on the transition process.

And so I think that one would have to have that exposure or at least that education to know what else needs to be changed within the setting.

Interviewer 25:50
OK, so for you, if I if I'm understanding right.
So the priority would be training specifically on what, how care would differ for the patient group?

Participant 2 25:57
Yeah.
Yes, exactly. Yeah.

Interviewer 26:01
Yes, exactly.
OK.
Do you feel that?
Training on sexual and gender diversity in general is helpful.
Or do you feel like it's more helpful if it's focused specifically on care needs?

Participant 2 26:20
No, I think it has to be general and then made more specific.

Interviewer 26:20
Thanks and any other thoughts or comments about any of these questions or any of anything we've spoken about?

Participant 2 26:34
No, nothing really.
Just an interesting area to, you know, to kind of think about.

Interviewer 26:42
OK.
Well, thank you so much.
So if it's OK with you, I'll stop the recording now in.

Participant 2 26:48
That's OK.

Interviewer 26:50
One second.

Interviewer stopped transcription

# Participant 3 Transcript

1h 20m 41s

Interviewer0:07
Have you had a chance to read the information sheet?

Participant 3 0:09
Yes, I have.

Interviewer0:11
Amazing.
And do you have any questions?

Participant 3 0:14
Not at this moment.

Interviewer0:14
Are you happy for me to record audio and visual?

Participant 3 0:19
Yes.

Interviewer0:19
Yes.
And just to remind, you can turn your camera off if you would like.
So just to start off, I'm gonna go through some demographic questions.
What is your professional role?

Participant 3 0:31
So my background is in midwifery and I'm currently at health visitor.

Interviewer0:32
All right.
Amazing.
How long were you working as a midwife for?

Participant 3 0:40
I was working as a midwife for..
Qualifying in 2020, I think it was two years qualified.

Interviewer0:49
OK.
And how long have you been health visitor?

Participant 3 0:53
Three weeks.

Interviewer0:54
Amazing congratulations.

Participant 3 0:56
Thank you.

Interviewer0:57
And what region do you work with in the UK?

Participant 3 1:00
[Location]

Interviewer1:01
[Location]
And how would you describe your gender identity?

Participant 3 1:07
Female.

Interviewer1:08
Female, thank you.

Participant 3 1:10
Cis female.

Interviewer1:12
Yeah.

Participant 3 1:12
If that's how you say it?

Interviewer1:12
Thank you.
Yes, right, and so just some questions about your experiences as a health worker before we get on to the bulk of it and have you ever had any experience working with transgender or gender non conforming pregnant people?

Participant 3 1:29
Yes, in a way.
It's very relatively limited and it's almost ... not through a third party, but like working as a midwife would be someone on the ward.
Umm that maybe I wasn't looking after personally, but they were there.
Things like that, but yeah, relatively limited because I haven't been working that long.

Interviewer1:51
I know of course, but I mean, any experiences is fantastic.
Do you feel that it was like a bit more notable than if there was someone transgender who was an impatient?

Participant 3 2:02
Yes, the experience when there was someone on the ward.
Umm it yeah.
It was the "talk of the town", I guess you'd say. At least it felt like that at the time.
This was a few years ago now.
Umm but yeah, it was like it almost was like a talking point.

Interviewer2:22
And were those talking points usually more positive and more negative which you say?

Participant 3 2:29
I wouldn't...
I wouldn't have said either in particular, umm, I guess people were kind of putting in their two pence about it and maybe some people felt more strongly than others, but I wouldn't have said it was positive or negative.
I guess there was...
There were people being negative in that, you know.
"Ohh, that's ridiculous."
You know, it's just "identify as gender you are", I guess was kind of their opinion.
Whereas others may be a little bit more, umm, modern.

Interviewer3:03
Umm so so I guess with all things that emit a range of views, do you feel like it was potentially a specific type of person that would perhaps be more positive or more likely to be negative?

Participant 3 3:17
I don't think so.
I think it I think it's really personal.
I don't.
I would have said there was a certain type of person, no.

Interviewer3:26
OK.
That's really good to hear.
And do you think that a lot of the?
Or how do you feel that many of the negative opinions or positive opinions were based in personal experience or more based in evidence, or just things that seemed unrelated to experiences that they've actually had?

Participant 3 3:49
I'd say people, I don't think people are going with any evidence.
I think they're just going with their feeling and I think a lot of the time it's not necessarily they've even thought through themselves.

Interviewer4:02
Hmm.

Participant 3 4:02
It's just they're either.
I just.
I don't know, it might be...
In my experience.
I'd say that people either just latch on to others opinions, or yeah, just have a view and I think that's right.
Umm.

Interviewer4:17
Right. OK.

Participant 3 4:19
Is it possible to go back to a question?

Interviewer4:21
Absolutely, of course.

Participant 3 4:23
Umm, because I was just thinking when you're saying a certain type of people.
I mean, not necessarily with.
That specific experience.
But I would possibly say older generations have a more negative view.

Interviewer4:41
Umm OK.

Participant 3 4:42
Umm yeah, I just.
I don't know.
I just find that generally.

Interviewer4:48
No, that's absolutely fine.
And as I said, this is all about your experiences.
So there's whatever you found is.
Is absolutely fine and do you?
Do you feel you said it wasn't particularly for that experience?
Do you think that it's within healthcare workers as well?
Older generations might have a more negative opinion or outside of the work environment.

Participant 3 5:07
Both I would say.

Interviewer5:10
OK.

Participant 3 5:13
Yeah, I've got a colleague in my current job health visiting.
Who is our oldest member of staff and she's kind of known for being a bit cranky.
And I would peg her as someone as being less inclusive.
Obviously that's then me judging her, but umm just comments you here around the office, isn't it?
Umm, it's interesting.

Interviewer5:41
Umm.
And how does it affect your team environment if when these sorts of comments are being made?

Participant 3 5:49
I think they can make it a bit awkward.
Uh, because you always gonna get conflicting opinions on it and think you can feel quite strongly both ends of the spectrum.
Some people aren't always willing to agree on that because I think people do feel quite strongly in one way or the other.

Interviewer6:13
Right.
And do you think there's affects the care provision that's being provided?

Participant 3 6:21
I would say so, yes, I think it's gonna vary.
Like whoever's looking after you, it's gonna vary as to who you have, isn't it?
And what their opinion is, because... Although in healthcare you are taught to you know, not oppress others with your opinions.
If you were to feel strongly, then you probably are going to without meaning to maybe.

Interviewer6:47
Hmm.
And what would you describe as the priorities when working with transgender people?
And it's absolutely fine if you would say that you're unsure.

Participant 3 6:58
Priorities so much, I think.
I would say.
Personally, like I'd want to be able to get pronouns right and address them as they want to be addressed.
Umm.
And I'll just say just yeah, having that conversation with them about how they want to be spoken about.

Interviewer7:20
Umm, so sounds like a person centred approach, yeah.

Participant 3 7:24
Yeah, the same way.
Yeah, the same way a cisgendered person.
You would make a point.
You would say he or she, wouldn't you?
And I feel like everybody, everybody should feel safe in that way.

Interviewer7:41
Absolutely.
So stepping back a bit from looking specifically at this patient group and can you describe a bit about your care priorities in general or if you'd have a self-described philosophy of care at all?

Participant 3 7:48
OK.
Good question.
Umm, it's interesting because obviously I'm moving between professions.
I'm relatively new as a health visitor, so it's very different way of working.
I would have said as a midwife it's all these classic things, you know, you want to be women centred.
You want to be compassionate and you want to feel like you can work collaboratively with your patient.
And I still think that...
That one definitely carries over.
I definitely still feel like you want to work collaboratively with families.
Umm, but I say I get to work more holistically with families as a health visitor than I did as a midwife because I have continuity and I work in the community and I see their home and you're assessing the family unit more rather than just the person in midwifery...
It can just be a bit.
Tick-listy.
I guess. Umm yeah.

Interviewer8:51
That's absolutely fine.

Participant 3 8:54
So I'd say yeah, being able to work with families more collaboratively is definitely something.
I enjoy doing.

Interviewer9:03
So it sounds like the continuity is important there as well for you.

Participant 3 9:07
Yeah, I think that's massively important, especially when you've got families that have not had a good time.
With things of late, not having to rehash over stories and things and say that was someone you know with.
May be an issue with their gender identity or they are transgender then.
Having that continuity in that same person, you're gonna actually build a relationship with them, aren't you?
I guess that is that is what it is about with the relationship building.

Interviewer9:42
Umm and kind of building on this and how would you define safe care?

Participant 3 9:55
Umm.
Umm.
Obviously.
I think primarily it's keeping people alive, isn't it?
And clinically well, and I felt that more so midwifery than now because now I do less clinical work. Umm.
But I think that.
Patients should always feel.
Feel cared for as well, in that they should never feel unsafe, they should always feel like... if you've got no relationship and you know patient can't talk to you as well to raise any worries, then that's not very safe.

Interviewer10:45
Absolutely and so in an ideal world to say we didn't have the constrictions of that.
Actually, we need to focus on keeping that person alive.
What would you like to see
Safe care encompassing?

Participant 3 11:01
I think the NHS at the moment, and well, obviously I work for the Council now, the biggest constraint is time, I think being able to give people your time matters immensely.

Interviewer11:16
Umm.
And thank you.
Sorry, I know that they can be quite existential questions. It's just to frame us before we start talking about some of the recommendations for the care provision.
Um and when you were working in in a hospital setting, could you describe the organizations’ philosophy of care or their care priorities?

Participant 3 11:45
I know we had, like we had the trust values, obviously at both trusts I worked at Umm and it was always.
Yeah.
Like safety, compassion, very much like the 6Cs of midwifery.
Personable, those kinds of things. Umm.

Interviewer12:04
Hm.

Participant 3 12:06
Yeah.

Interviewer12:08
Yeah.
Thank you.
Umm.
And before we go into the care recommendations.
We did speak a bit previously about times that they've been a transgender person on the wards.
Do you feel that the healthcare professionals working with and for them have the right tools and resources to provide that that's safe care that we've spoken about?

Participant 3 12:34
At the time.
No, I don't think so.
I think because it was something that was probably quite newly emerging.
I don't think so.
At the time. Safe, in terms of clinically, but in terms of their or emotional wellbeing, perhaps not.

Interviewer12:52
Or.
Umm.
And what sort of tools or resources do you think could have helped those midwives or other health workers?

Participant 3 13:05
I think a lot of people want to know how to get it right as such.
Umm.
And I think that has become more of a thing now and.
What was the question types of things?

Interviewer13:19
The yeah, it could be something physical.
It could be it any sort of like tool, resource, education, anything that could have been helpful for care provision.

Participant 3 13:32
I guess like, yeah, training, I think they just need training and.
I don't know.
I think it's hard because it's not something you can just like, pull an info sheet out a drawer and it's got all the answers on it.
So that's tricky one. I don't know what else.
I mean, I guess there's always like eLearning.
You can do and if it's not like an in person thing like an eLearning module to add that on to all the other things you have to do, but.
Umm.
Yeah, I guess, yeah.
It's just.
I'm not sure what format of education but.
I think in health visiting, we did.
I think it might be part of uni.
We had like a module on it, so obviously we had like a lecturer and I thought that was really helpful.
Umm something like that.

Interviewer14:35
Umm OK, so an in-person education session.

Participant 3 14:41
I think so.
I think that's better than doing an eLearning module.
People actually take stuff away from it.

Interviewer14:44
Yeah.
Yeah.
And who do you feel like would be in the most appropriate or the most helpful to deliver this sort of training?

Participant 3 15:01
You need.
I can't remember who... what kind of person we had do something like that for us at uni.
Umm, but she had a lot of experience and that's the main thing.
I think, is someone with experience, possibly even someone that had, like, knows the healthcare setting as well.
But I don't think that's necessarily like an absolute necessity.
Umm, I think just someone experience with working with that community.

Interviewer15:33
So it would be someone who holds a professional role as well.

Participant 3 15:38
Yeah.

Interviewer15:38
Yeah.
OK.
And just stepping back a bit, you said that you had some education, a bit for your health visiting training, do you remember any education for your midwifery degree?

Participant 3 15:53
No, I don't remember doing anything on at uni or afterwards really.
We did a lot on like women's emotional health, and it was very much.
For cis women on say then you know, as a more inclusive kind of community.

Interviewer16:17
Umm OK.
It is really interesting that that you've brought up training because this is actually one of the recommendations that we wanted to talk about from the literature of you as one of being the most one of the most important things to help to have safe, high quality care provision.
Um, so going on from...
From what we've spoken about and from that and how feasible do you think it would be for there to be a training for health workers on caring for transgender people?

Participant 3 16:50
Definitely feasible 100%.
I do think it should be...
I think it is coming into workplaces umm as a professional thing, hopefully in healthcare as well.

Interviewer17:03
Umm, OK.
And would having additional training change how safe the care provision was, in your opinion?

Participant 3 17:15
I'd like to think so.
I'd like to think that.
I'd like to think it would open people's eyes and.
Make it a more even.
Make care more even across the board, if that makes any sense, so that people are on the same page with what they're providing, same as other training that you would have.

Interviewer17:40
Yeah.
And so.
So you feel that it?
It would increase safety.

Participant 3 17:45
Yeah.

Interviewer17:46
Yeah.
OK. Amazing.
Um so any other comments or thoughts about experiences you've had or things you've seen in your professional roles?

Participant 3 18:01
Trying to think now.
There was, yeah, there was obviously.
The person on the ward, which I didn't have personal experience with but was kind of there.
Other than that, so it's interesting we did.
I did a piece of training as a health visitor.
Obviously it did like the training as in Umm, we had a workshop on.
I can't really remember what it was called, but it's something like "ethnicity and inclusivity" kind of thing, and she was amazing and she was saying about how, you know, there's no right or wrong in terms of the pronouns and this is how you can ask people these kind of questions like things that people maybe feel a bit uneasy about.
Umm.
And I found that really helpful and I felt like there were people going into that thinking "what a load of rubbish" and actually walking away like "that was really helpful".
I think that's great.
Umm, I had a not so good experience within my.
I was actually not that long ago.
I think I was still in my training to be health visitor and we there's something we complete which are called "review health assessments".
So, umm children that are a "LAC" are "looked after child" have assessments every few months to basically see how they're doing with their health and development.
And it was basically going through the questions that you ask. how you write up the report, those kind of things.
Umm.
And the person that was doing the training was.
Basically, saying about how you would ask two or three year olds what their pronouns were and documenting the clothing they are wearing.
If they were identifying as a boy or girl that day, umm and these kind of things which a lot of us agreed wasn't particularly appropriate.
Umm.
Obviously it's, I think personally it's absolutely fine to document.
What they were wearing and what toys they were playing with that day, but I don't think at two or three years old you can necessarily ask them their pronouns because they're still very, very young.
And I also don't think that they maybe when they're older, they will say, oh, I I knew at that age.
But I think you almost pushing it down their throat a bit to say "ohh you were a boy today" or "you were a girl today".
I don't think that's fair.
So that was the kind of negative, not necessarily a care experience, but educational experience for us.

Interviewer20:59
Umm.
Umm.
OK, so kind of a more and more focused on gender identity than you would feel was appropriate at that sort of age?

Participant 3 21:16
Yeah.
Obviously, working with under fives.
It's, you know, that they're under 5.
They're so, so young.
Still, umm.
And yeah, I didn't think it was appropriate to be saying "ohh Ella was playing with dinosaurs today.
So she's a boy."
That's that's just not really appropriate.
Umm, as you've got obviously right.
A teacher.
Who's working with teenagers?
I think maybe that's different story.

Interviewer21:45
OK.
Um no thank you for that as well.
So I guess, I guess thinking back to the safety, how do you feel that that sort of care provision would impact safety for safe care provision?

Participant 3 22:03
As in, with like the woman talking about the that?

Interviewer22:07
Yeah.

Participant 3 22:09
I think that's.
I'm not sure what necessarily impacts their safety.
I'd say it's.
It's just gonna possibly impact their development.
Let's say.

Interviewer22:26
OK.
No, thank you for that.
Thank you for sharing your experiences as well.
So if it's OK, I'll move on to some of the recommendations that have come up.
So you've already spoken a little bit about training.
Any other thoughts or comments about training to support healthcare workers to care for transgender and gender diverse pregnant people?

Participant 3 22:51
Umm.
I don't think so.
Umm, I would think.
I just think it would be great to do that, to be honest.
I think it would be good to kind of get the ball rolling with that.
I guess it would be like how?
How would it be delivered in terms of who are you...
Who is going to deliver it?

Interviewer23:18
No, that makes a lot of sense. I'm.
Ah OK.
So if we look at some of the other recommendations and so these are in no particular order of a frequency, it's just things that have come up the most.
One of them is actually something you spoke about in in what you would see as your care priorities, which is asking service users their pronouns.
Um so, for...
For yourself, what do you think of this as a recommendation?
Do you see this as important or not so important?

Participant 3 23:54
I guess it depends.
If, as in, would that be a recommendation for you then asking everybody?
Because I think it would, I'd say yes, like definitely ask people what their pronouns.
But equally,
I'm not sure it would be entirely helpful if you're only asking certain people who make sense.

Interviewer24:21
So you're thinking about not discriminating based on how someone appears?

Participant 3 24:27
I'd say, yeah, I'd say if you're gonna.
If you're going to recommend that it would be across the board, you'd be... like that's just a part of care.
Same way when you walk into a room as a labour ward midwife, umm and their names written down as "Rebecca".
You would say, "are you a Rebecca, Becca, Becky?" and they say "ohh
I usually go by this". Like that's, I think we could make that quite normal with asking pronouns.

Interviewer24:59
What do you think would contribute towards making that more normal?

Participant 3 25:04
I think just doing it.
People...

Interviewer25:08
Practicing.

Participant 3 25:08
Just doing it, just exercising it like you would... as you do when you go and put on a CTG or it's just, it would just become a part of the process.

Interviewer25:22
Umm.

Participant 3 25:22
And I think you just have to.
You just have to start doing it, really. Make it...

Interviewer25:28
Yeah.

Participant 3 25:29
Making it a conscious effort to do that when you introduce yourself.

Interviewer25:35
And do you see... How feasible do you see this?

Participant 3 25:40
I mean, I personally don't think it's a big ask and say it.
It doesn't take very long, does it?
But that could make a really big impact to someone that's maybe unsure, or, you know, might really value
Actually just being asked that.

Interviewer25:55
Umm.
So if we think back to our what we said about safe care provision.
How does this impact the safety of care provision?
Do you feel?

Participant 3 26:11
Say that again, sorry.

Interviewer26:12
Sorry that I phrased that a bit confusingly.
So if we think back to the things we spoke about with safety, which primarily is keeping someone alive, but secondarily building that relationship and making sure they always feel safe, how do you think that impacts the safety of the care that you're providing?

Participant 3 26:32
As in the I put the priorities that way round?

Interviewer26:36
Or, if asking pronouns does that at all contribute towards safety?

Participant 3 26:39
Yeah.
Yes, I would say so.
Umm, more so for the fact that that will help them feel safe in that environment, that they're in an inclusive environment.

Interviewer26:55
Hmm.
OK and.
Is this something that's currently being done in in care environments?
Do you feel?

Participant 3 27:06
Not, not particularly.
I don't think, not as like a day-to-day occurrence.
I mean now doing less clinical work, more office based work.
Lots of people will put umm pronouns and things like on their email signature, but not necessarily asking of other people.

Interviewer27:31
So potentially that that could help with the normalization in like a digital sphere.

Participant 3 27:37
Yeah, yeah, definitely.
And I say that that's definitely taken off a bit.
But I think people hold back from just asking because they're probably a bit scared and like, I mean, I'm definitely guilty of that sometimes.

Interviewer27:52
Umm.

Participant 3 27:55
I wouldn't go straight in.
Uh.
Within that small piece of training that we had, the lady was saying about, just asking people, "how do you identify?"
Just raising the question and saying that, you know, generally the response that you get at is quite positive that people actually like being asked that.
Umm.

Interviewer28:17
Umm.

Participant 3 28:18
Which I just found pretty interesting.
Sorry, bit off piece.

Interviewer28:21
No, no, not at all.
And.
What sort of tools or resources or information do you think that healthcare workers would need to start asking service users their pronouns?

Participant 3 28:44
I guess your if you're going to do training. Training on like how to ask that question in just saying like "how do you identify?"
Tools...
It's really tricky to come up with something on the spot.

Interviewer29:06
No, it.
And that's absolutely fine.
It's OK if you if you don't...

Participant 3 29:10
I'm sure there's really good ideas out there, I'm just not thinking of them.

Interviewer29:15
No, that's fine.

Participant 3 29:16
I think well yeah, either like adding it into training.
Maybe it is, I don't know.
Like if you did have...
Something to refer to, either physically or electronically, that had like ideas of how to ask.

Interviewer29:39
Umm.

Participant 3 29:39
Like, have you tried this?
Have you tried that?
Umm, like a like a pocketbook style thing.

Interviewer29:46
Hmm.
So some literature for staff?

Participant 3 29:50
Yeah.

Interviewer29:52
Yeah. OK.
Any more thoughts about asking people their pronouns?

Participant 3 30:05
I don't think so.

Participant 3 30:07
I think I've said my piece.

Interviewer30:09
Yeah.
No, thank you so much.

Participant 3 30:12
I think it's just some.
I know it's something that.
That is not that difficult to do, but if it makes a difference to someone, then why shouldn't you try it?

Interviewer30:23
Absolutely.
So kind of building off that, another recommendation was asking service users their preferred terms for their anatomy and bodily functions.
So in a common example of this might be chest feeding instead of breast feeding, but some less common examples could include different terms for a vagina.
So someone might not want to refer to that anatomy as a vagina.
They might prefer term like pelvic outlet or front hole and so this recommendation was really so.
In summary, it was asking service users their preferred terms for anatomy and bodily function.

Interviewer31:04
What do you think of this in in your care provision?

Participant 3 31:09
I think.
I mean, I think that's absolutely fine.
The only difficulty of it is obviously in a medical profession, I think it's not as easy.
Umm.
With those type of things.
But then I think.
It would probably only be in certain circumstances, so I'd say definitely ask, but then maybe also add in that you know in in certain situations we might need to refer to it as.
"This,
Like would that be OK?" Because
Otherwise, I think ... I don't know.
There's potential for wires to get crossed. Possibly.
But then in like your day-to-day practice, like if you're working on a postnatal ward like saying chest feeding would be fine, I think everyone would know what that is.
It gets more difficult when you're referring to a vagina.
I'd say yeah, that's that...
That one is more tricky, I'd say.

Interviewer32:23
And that miscommunication that you're speaking about is that between healthcare workers and service users or healthcare workers and other healthcare workers?

Participant 3 32:35
Possibly more so between service users and staff.
But then that it could be between staff as well.
If you're a midwife looking after somebody, and you were asking the doctor for stitches and then you are explaining where, then did you see what I mean?
That might just get a bit confusing with that.

Interviewer33:01
Umm, so it seems like that there's a time and a place where medical terminology, it seems more appropriate for you?

Participant 3 33:08
Umm I also yeah.

Participant 3 33:12
On a bit of, not personal experience, but I know that there was a situation in the hospital where parents didn't want the baby, basically to be gendered.
Umm, so it was really difficult when I think there was an issue with the genitalia.
Then, them talking about that and obviously it's important. In terms of like, opening up a bit more, I guess you've got like the legal aspect in terms of you have to legally register them and that bit would be a bit difficult if they don't want to refer it, refer to them as.
A boy or a girl, or a by saying they have a vagina or penis.

Interviewer34:01
No, that makes complete sense.
So we we've kind of touched on this with your initial thoughts, but how feasible do you feel that this would be in health care and practice?

Participant 3 34:18
At the moment, not very feasible.
I'd say starting with pronouns definitely would be a good start.
I think going into the root of names for certain body parts would be more difficult to implement at the moment.
And because I think that ... I think there would be more conflict in terms of staff members being on board or not on board with it.

Interviewer34:50
Hmm.

Participant 3 34:51
And yeah, I think just kind of dilemmas we talked about in terms of like miscommunication and these kinds of things.
I was thinking in, but in discussion with like as a staff member in discussion with the service user, they wanted to call it chest feeding.
That, yeah, then that that's definitely feasible.

Interviewer35:20
Hmm.
Do you?
Do you feel that there's any degree of feasibility?
So for example, you said at the beginning that chest feeding might be more feasible than a preferred term for genitalia.
Would you be able to expand on that at all?

Participant 3 35:39
That's yeah, I think.
I think because... but then,
Pelvic...
Yeah, cause pelvic outlet could mean different things.
If, like interpreting that personally, I could interpret that differently.
Whereas chest feeding it's yeah, chest and breast, I guess a bit more interchangeable.
So I'd say chest feeding is is more feasible than pelvic outlet or front hole.

Interviewer36:12
Yeah, that's absolutely fine.
And do you think that this preferred language is something that's currently taking place in healthcare settings?

Participant 3 36:25
No.
Short answer, no I don't.
I really don't think it is. Chest feeding definitely been heard about.

Participant 3 36:34
Umm.
And then I think people would really...
Yeah, I think the general response I've heard about chest feeding, is caused uproar.
So I think, yeah, other things as well, which.
I don't think so.

Interviewer36:56
Yeah, that's fine.
Can you expand a bit about the uproar with chest feeding?

Participant 3 37:03
I think.
In midwifery,
It's difficult because even in your training it is "women centred care" and all about women.
You know you don't go in.
You're told you don't go into it for babies.
You go into it for women and.
You know it's you want to be proud of your body as a woman to be able to do those things, to child bear.
And I definitely get that.
So I think then.
Comments, like "men can give birth", chest feeding to be more inclusive.
A lot of people, and I guess I have my own view on it as well that it's it, I guess what people touching on is that it's taking away from women to be inclusive of those other than cis women.

Interviewer38:04
Hm.

Participant 3 38:05
I think I said that right, but I think.
I don't think that's necessarily right because you're not taking away anything.
You're just adding to it.

Interviewer38:21
Umm, so it sounds like you're leaning towards.
There's a feeling of erasure.
Among cis women, potentially?

Participant 3 38:29
Ohh.

Interviewer38:31
Umm.

Participant 3 38:31
I think... what's the word, sorry?

Interviewer38:35
Erasure.

Participant 3 38:38
What does that mean?

Interviewer38:39
Oh, sorry.
As in cis women are being removed or rubbed out from the conversation.

Participant 3 38:50
Not necessarily.
I think that's how maybe they feel, some feel.

Interviewer38:58
Hmm.
No, that's completely fair enough.
And who do you think?
And it's OK if you don't know.
Who do you think the “they” are in this situation?

Participant 3 39:09
As in.
Some of the cis women that feel?

Interviewer39:12
Who?

Participant 3 39:17
Confused myself.

Interviewer39:18
No, no, sorry.
So you said that some people feel that instead of adding to the conversation, it's being taken away from them.
And who do you think feels this way?

Participant 3 39:30
I'll say cis.
Yeah, cis women.
Umm would be.
Would feel that.
Well, I've known some cis women expressed that they don't agree.

Interviewer39:49
Hmm.
And is this more healthcare professionals or people who have been pregnant or people outside of a medical sphere?

Participant 3 39:59
I say, yeah, more general, rather than particularly health professionals.
You'd like to think that health professionals are generally on the inclusive side.

Interviewer40:13
OK.
Umm.
And if we, if healthcare professionals were to ask service users their preferred terms for their anatomy, what tools, resources or information do you think that they would need to do that?

Participant 3 40:32
I think you definitely want some guidance with that.
So I guess again like the little pocketbook idea of like suggestions for.
Umm.
And just anything that's, I guess it's gonna give people confidence to ask.
And if you've got maybe that little sheet, then you're a little bit prepared for answers as well.

Interviewer41:00
And how do you feel that asking preferred terms for anatomy would impact the safety of care provision?

Participant 3 41:10
I think because of like what we talked about with the miscommunication, let's say it's.
There's potential that it wouldn't add to their safety to ask.
I think they would again like feel safer in an inclusive environment, but.
And well, medically speaking, umm, you need the communication with obviously within sort of staff members to be on the same page.
So I guess if you've got, if it I guess if you if you did ask and they said "OK, we're referring to it as the pelvic outlet" and then everyone was you know on board with that, that would be fine.
But I think the issue would be if maybe people weren't and obviously like we've midwifery and health visiting, I guess you've got different professionals going in that aren't necessarily all joined up together because that's just the way it kind of works I guess.

Interviewer42:17
Hmm.

Participant 3 42:17
So that would be more difficult.

Interviewer42:20
OK.
Thank you and.
Any other thoughts on that one?
Or otherwise, we'll move on to the next one.

Participant 3 42:29
I don't think so at the moment.

Interviewer42:30
OK, so kind of building on something we spoke about.
Just before about asking about pronouns, one of the other recommendations was the documentation of correct pronouns. What do you think of this?

Participant 3 42:46
As in, is it happening, or do I think it should happen?

Interviewer42:48
Uh.
Any thoughts at all?

Participant 3 42:52
Ohh.
Umm, I would say if it's not being asked, it's probably not being documented.
I'd like to think that people do document it correctly.
But also, making it really clear and.
Yeah.
Because then that would maybe stop, because there's asking, and then there's every single person you meet within your care asking.
So it was probably be nicer to service user if someone asks and then it's documented and that can be kind of clearly kept throughout.

Interviewer43:26
Hmm, so how feasible do you think it would be for pronouns to be documented?

Participant 3 43:34
How easy?

Interviewer43:36
Yeah.
Easy or feasible?

Participant 3 43:38
Well, very feasible.

Interviewer43:42
Hmm.

Participant 3 43:42
I mean, we're on.
We've got electronic records now.
It cannot be that difficult to have a section for that.
I'm sure we've all the things that you can do like plotting on graphs and stuff online.

Interviewer43:49
Umm.

Participant 3 43:53
I'm sure it would be feasible to be able to put pronouns on there.

Interviewer43:58
I'm really glad you brought up the electronic medical record systems because that is actually the next one we're going to talk about as well.
So ahead of the game. That's fantastic.

Um so where do you see?
So as obviously you said, electronic medical record systems, so is this where you would see the documentation happening or where else do you feel would be a helpful place for them to be documented?

Participant 3 44:25
I would say.
I guess a helpful place would be documented would be you've got the NHS spine obviously which like carries all the people's details.
I mean putting it on there would be quite handy because then that's gets translated through to like your GP, child health.
So then that kind of goes across the board.
So that would work quite well, umm.
And then yeah, I think you don't really have paper anymore.
So it would just be then having it in a nice little big space or something, or on just like the front.
I guess with demographics is what I'm going with.
Yeah, just make it clear, like you know, name, address, pronouns. Yeah.

Interviewer45:14
Perfect and.
Do you think is there any documentation of pronouns currently?

Participant 3 45:29
No.
Not that I've seen, I don't think when whenever I've seen it documented, it's on someone's email signature that I can think of, that's all.
And that's obviously professionals, not I've not seen it on services users I don't think.

Interviewer45:49
Umm.
And so in that example, we spoke about where there was a transgender pregnant person on the ward.
It sounded like it was more of a verbal handover between staff rather than anything documented for that patient?

Participant 3 46:02
For umm yeah, I mean it might wanna have been on like we used to do hand over sheets where you would have like a little, umm, but that was like, not really an official kind of document.
That was just like between staff members.
You would update the sheet.
Umm, so yeah, it was kind of more passed through word of mouth.

Interviewer46:26
Hmm.
Umm.
And what sort of tools, resources do you think would be required for us to document or for health workers to document pronouns?

Participant 3 46:41
I guess you're going to need.
You're going to need IT to implement it like to add it in on to systems, and then obviously you've got all the different types of.
Umm electronic records cause.
Obviously you've got like the spine, badgernet, epic and we've got [electronic medical record system name].
So you're gonna.
You're gonna then have to.
I guess...
Those are the tools, I guess.
And you're gonna have to kind of approach them all to, do same thing.

Interviewer47:16
Umm.
And I think I asked already on all those systems.
Do you?
Do you feel there's a?
Across the board level of feasibility, or is there anywhere that would be easier or harder?

Participant 3 47:34
I don't know because I don't...
I don't know how easy it would be to go to implement it.
Obviously...
You know from within.
Like what I use at the moment?
It probably be a case of like I would approach the person who's the head of all of that stuff and getting them to put a little box in.
But another idea would be when people register with the GP that it goes on that document form and then gets put in there because then that would get translated out I guess.

Interviewer48:08
So on intake forms?

Participant 3 48:10
Yeah.

Interviewer48:11
Umm yeah, and
How much does this contribute towards safe care provisions you feel to document someone’s pronouns?

Participant 3 48:27
I mean, in a way, I guess I don't think it really impacts their care ...
Well, that's not true.
And that they'd still have the same, you know, the same processes would still happen, but they would be able to have been referred to what they identify as.

Interviewer48:51
Hmm.
And so thinking about that emotional safety that we spoke about, and do you think it would impact that at all?

Participant 3 49:00
Yeah.
More than, yeah, more than anything, I would like to think that medically everything would happen as it does.
And then, yeah, in terms of more their emotional wellbeing and how they perceive their care would be impacted.

Interviewer49:19
Umm, I think you and any other thoughts about the documentation of pronouns.

Participant 3 49:27
I think that's covered it.

Interviewer49:30
Yeah.
Amazing.
And I think we've combined really nicely the electronic medical record systems recommendations with the documentation of pronouns recommendations, but just a little bit more context for that.
One issue which came up in in the literature quite frequently was the electronic medical record systems not allowing sex to differ from gender and not allowing male patients to be admitted to labour wards or women's only wards and.
How?
How much do you feel this?
These sorts of problems could impact care provision?

Participant 3 50:20
I'm gonna go to the second part first, because that comes to my mind.

Interviewer50:24
OK.

Participant 3 50:24
But with I guess with.
Men, I guess the men being on a female only ward.
I mean.
I as a tricky one more because.
I don't know, but then you've got things like side rooms.
I think that would be feasible.
It would be more.
You've got to think of patient safety in terms of even don't necessarily know the other of the people’s opinions on the ward.
And I guess as a care provider, you don't want to put your patient on a ward that they're unsafe on.
Possibly from some other people, not from a care point of view.

Interviewer51:23
Hmm.

Participant 3 51:24
Umm.
And if you were looking at someone that was maybe committed, got a criminal record, that would also be difficult.
As in.
Someone that was born a man who's got a criminal record, possibly against women, cis women.
Yeah.
That's really hard.

Interviewer52:02
Absolutely, that's fine. So

Participant 3 52:06
Struggling to articulate it.

Interviewer52:10
You said a about like utilising side rooms and private rooms.

Participant 3 52:14
Yeah.

Interviewer52:15
Private beds.

Participant 3 52:20
I think, but I'd like to think that that isn't ... that would be a temporary measure.
And not as people are...

Participant 3 52:30
Maybe of more educated ... it would just become normal.

Interviewer52:38
Hmm.
And in a pregnancy care setting, so do you...
Do you feel like that this the side rooms would be the way forward in in shared bay and ward environments?

Participant 3 52:56
Possibly.
But it's difficult because you have to treat them as you would kind of.
You're not treating them as any other patient, but also you know, you've got other women that are on the ward and plus, you know, part of their care.
I think it would really depend on the individual perhaps.

Interviewer53:28
Umm, so going back to that person centred.
Care that we're speaking of.

Participant 3 53:33
Yeah.
And obviously if that's kind of why you've got, you know, with complex care midwife or we've got a certain team in [location] that you know people with any kind of that's additional needs or support can kind of have a bit of a plan made.
So it might be that they just need that further kind of input to make that plan for OK, when you are an impatient, this is what we'll do.

Interviewer54:01
Umm.

Participant 3 54:06
This is what's gonna happen.

Interviewer54:08
Umm, you brought up to two really important things, the communication between professionals and service users and also the forward planning for birth and the postnatal period.

Participant 3 54:24
Yeah, I'd say that's that.
That part is going to be really important in terms of pregnancy and I guess that is bringing to light the safety is.
Because it could be if.
If this person's identifying as a man, it could be quite confronting to go into labour and birth and have to, you know, talk about body parts or maybe didn't want to talk about and be asked a lot of personal questions.
It's probably quite a big thing, so I'd definitely say that planning aspect would be quite important.

Interviewer55:04
Umm.
And what do you think could contribute towards a...

Interviewer55:11
A helpful plan or like a... sorry I can't phrase my words now,
Something that could...
A plan that would support a person for when they go into labour.

Participant 3 55:27
I guess it's the same as when you do just...
I guess your bog-standard birth plan, you're asking those questions like pain relief and kind of you know, "do you want to cut the cord?"
All those bits and bobs. It is that forward thinking and.
That bit of planning of like what to expect.
Ohh.
I guess for pregnant person it's going to be important to, I guess not...
Bury your head in the sand and being able to.
Sort out the kind of.
You know "what are we gonna call the body parts?" and those kind of things beforehand will be better than being in labour and having to have difficult conversations.
Umm, because it is, you know, painful, stressful at times.
Not necessarily, but.
And obviously there's different modes of birth as well, so that's kind of important to talk about on that.

Interviewer56:43
Hmm.

Participant 3 56:45
You might want to repeat the question I feel like I've gone way off.

Interviewer56:47
No, no, it's really good.
I'm and so.
So what you're really like referring to it, if I'm interpreting correctly, is appropriate antenatal education?

Participant 3 56:59
Yeah, I guess...
Yeah, I guess I'm quite.
I've always believed quite strongly in the antenatal education and I think...
It's like with some of my clients now that have got additional needs and need like extra support and that kind of complex planning is all about, you know, preparation, managing expectations.
Umm I think that's really important.
Like to sort of mentally get your head in the game of what is gonna happen.

Interviewer57:34
Umm.
And in the hospitals you've worked at have health workers or midwives provided antenatal education classes?
No.

Participant 3 57:47
Not since COVID. I did them as a student, umm...
And then it pretty much stopped from there.

Interviewer57:55
Umm.

Participant 3 57:55
And it is actually a big, I found it's quite a big thing.
I know in [location] as well because there were different classes and now there's just nothing.

Participant 3 58:08
And now when I do my antenatal appointments,
Hardly anybody, unless generally are more middle class and have the money to pay for.
A private hypnobirthing course or NCT.
Umm, people aren't doing any antenatal education. But I do think that really helps to manage your expectations in general.

Interviewer58:33
And was antenatal education.
Something you were able to provide at these appointments?

Participant 3 58:40
I mean, I tried to include little bits as much as I can.
I guess a lot with I did one last week that was... we did a lot of feeding.
Umm, just the expectations of newborn, normal newborn behaviour.
Umm.
Or just kind of the labour and birth bit, I think there's a lot.
There's a lot to think about.
Umm.
And it's not necessarily carried through generationally like it used to. Maybe.

Interviewer59:19
And so in the role you're in now, is there...
Is there a difference really in in the time you can spend having these sorts of discussions?

Participant 3 59:28
I would.
I would just love to have so much more time.

Interviewer59:32
Hm.

Participant 3 59:33
Umm, I mean, I know it's probably a more personal passion, the antenatal side of things.
Yeah, I would love to have more time to do all that education.
I mean, I did nearly set up my own hypnobirthing course, but I didn't.

Interviewer59:51
And if you were, say one of your caseload was a transgender person, do you feel that you'd have the tools to provide that, that same education for them?

Participant 3 1:00:05
I'll probably feel more unsure of myself because it's not something I've done.
I've never done it before, but I feel like a lot of it is going to be the same.
I think as long as you.
Can treat people, you know, just I think just being honest, you know, "I'm not really sure on this, but I'll ask" or also just... you get a long way in asking them their pronouns and then say you're going to address feeding you'd say, "umm, have you thought about feeding?"
"Do you have a preferred term?
You would like me to call it or anything to avoid?"

Participant 3 1:00:48
I think just, I don't know, just working with them. Umm.

Interviewer1:00:57
Is there anything that you think would help you or other health workers to feel more confident in this care provision?

Participant 3 1:01:11
There's nothing really coming to mind.
Again, I think it's something that will probably just develop as we are starting to do it more. Umm.
I don't know about anything in particular.
That's... because it's not something that people have done before, so it's not like someone can, you know, you've got midwives that was delivered, hundreds of babies they can come in and tell you how to do this, that
And the other, I'm not sure there's the script for this just yet.

Participant 3 1:01:39
So I think it's hard.

Interviewer1:01:41
Yeah.
No, absolutely.
And.
How much does antenatal education in general do you feel contribute towards the safety of care provision?

Participant 3 1:02:00
I think.
Obviously, the research around antenatal education is that it's basically a great time to educate women and families and.
Umm.
I think.
You might have to repeat the question.

Interviewer1:02:30
No, it's OK.
Do you feel that there's any and benefits in terms of safety from antenatal education?

Participant 3 1:02:37
But.
I am recovering my train of thought now.
Yes, well obviously it's known as being a good time to educate families, but I think it helps both.
Professionals and clients in terms of their safety because they're just more prepared for.
Outcomes.
And like I said it kind of helps manage those expectations, so I think... but then it's not necessarily going to change certain aspects of their care so....
Yeah...

Interviewer1:03:25
Umm, so falls more in that emotional safety again rather than physical?

Participant 3 1:03:34
Yeah, but I think that you can really empower people too.
You know, to take kind of control with their own care, which but also benefit there medical, physical safety as well.

Interviewer1:03:51
Umm.

Participant 3 1:03:51
I guess if you're talking to someone about signs of labour, then they're gonna perhaps more accurately judge when to go into hospital or when to get their midwife to their home birth.
So yeah, I would say both.

Interviewer1:04:09
Yeah, no.
That's really interesting.
So going back to the medical records a little bit, umm do you feel that, and we have spoken a little bit about this in terms of ward environments, do you feel that having a more inclusive system, so this is one that allows sex to differ from gender and allow...
The computer allowing the admission of someone who identified as male to the labour ward.
Would this impact the safety of care provision at all?

Participant 3 1:04:48
Possibly.
I mean.
I feel like there's got to be like, say, you accidentally admitted.
A man to labour ward that was meant to go to a different ward, for example, because they had changed their system.
I mean, there's got to be a way around that, I feel.
Because if, say on an online system you've got them registered and then surely you would just register them.
Say it's labour ward.
You'd basically have like a giant list. I guess.
Almost like a caseload of, like people that can be admitted.

Interviewer1:05:37
Umm.

Participant 3 1:05:38
And then you would just be able to admit it from there.
Then maybe that wouldn't happen, and it might not be that simple.
But just thinking out loud, really.

Interviewer1:05:48
Yes.
So other criteria to allow admission rather than just focused on sex?

Participant 3 1:05:55
Umm yeah, I think because yeah, it could impact safety if you could...
Admit any one to any ward, wouldn't it?
But I feel like if it was well thought through, you could definitely do that.

Interviewer1:06:10
Hmm.
OK.
Any other thoughts on electronic medical record systems?

Participant 3 1:06:21
I don't think so.

Interviewer1:06:23
OK.
So I'll move on to our last recommendation and this one was gender inclusive posters and decorations.
What do you think of this in a pregnancy care setting?

Participant 3 1:06:40
Oh, I don't see why not.

Interviewer1:06:43
Umm.

Participant 3 1:06:44
You know, we've got, there's, there's all sorts out, though.
Think we've got all sorts put on...
On the notice boards and that. I know on antenatal ward.
In [location].
I had to.
Well, I didn't have to.
There were.
There was like induction of labour and I'm sure we have one at [location] as well. Umm.
I guess.
Is it just adding?
Do you say gender inclusive?

So I guess you maybe want to alter what is there as well.
As adding.
Or maybe... Yeah.

Interviewer1:07:41
So what would it look like in your opinion?

Participant 3 1:07:51
The definitely loads that you could do.
There would be slightly more different...
You know, when they you've got like those posters where it says about the size of your cervix, but obviously you wouldn't really be able to do that if you don't wanna refer to it as a cervix.

Interviewer1:08:13
Hmm.

Participant 3 1:08:13
So whether you have a mixture.
Of things up.
I guess that is being gender inclusive, isn't it?
Is, you know, you've got things for trans and cis women?
That would be OK.
I guess it depends.
If, as a trans woman, you're offended umm, if someone showed you a poster of cervix.

Interviewer1:08:48
Umm.
And I guess also keeping that informative side of those of those documents as well.

Participant 3 1:08:58
Like induction of labour like is fine, because that's just you've got like, you know, positions for labour and, you know, literally anybody could do that.
Couldn't they? But it could be that you maybe change the image that's included with the information.

Interviewer1:09:07
Umm.
Hmm.
And how feasible do you think it would be for these images or posters to be changed or added to?

Participant 3 1:09:25
I feel like that's definitely feasible.

Interviewer1:09:28
Umm.

Participant 3 1:09:29
I think, yeah, definitely.

Interviewer1:09:35
And is there any specific tools, resources or information that would be needed to make those changes?

Participant 3 1:09:49
I guess to use as a resource would be asking like service users what they'd wanna see because half the time the there's not great stuff, like not decorated with great stuff anyway.
So using that as a tool to, you know, develop it, I guess.
Yeah.
Don't know what else you would.
I guess having it in different formats as well, not just like a printed poster, but again, it could be umm, I know that at [hospital trust].
Umm.
Basically, anyone pregnant gets there at the next few and app, and I think there's certain things on there would like information on.
So I I guess you'd have to look at doing it like again electronically, physically physical copies of stuff.

Interviewer1:10:56
It's very interesting.
So thinking more about the umm, the digital environment as well as the inpatient environment.

Participant 3 1:11:04
I guess that's good.
That's they.
There is so much digitally now, including notes that it's everything's moving that way.

Interviewer1:11:15
Umm.

Participant 3 1:11:15
So they're like, yeah, changing posters and stuff would be great.
You probably want to address the other stuff as well, I guess.

Interviewer1:11:23
Umm.
And do you think that this is something that's currently being done?
So are posters and decorations being changed to be more inclusive?

Participant 3 1:11:37
I'd like to think yes, but about walking onto a ward and actually seeing it.
I'm not entirely sure. Umm.

Interviewer1:11:45
Umm.

Participant 3 1:11:47
I'd say from like from stuff you see.
From just the stuff you see in like day to day life.
I'm not sure if I've seen a poster. Uh.
Definitely seeing.
Seeing more inclusive images and Instagram has got loads of stuff which is really good.
But yeah, not sure in terms of like an actual clinical environment.

Interviewer1:12:16
Yeah, so lots of information outside of a health setting as well.
It sounds really fantastic. And how much would gender inclusive posters and decorations impact the safety of care provision?

Participant 3 1:12:22
Umm.
I guess a pregnant person might feel that they can... That it's just more accessible and that they can actually get the information that they want about...
Anything within their pregnancy, I guess the same as you know.
Any cis woman would want to as well.
Umm, you know, that just makes it fair, really, doesn't it?

Interviewer1:13:09
So you feel it would help people to be more informed?

Participant 3 1:13:14
Yeah, which I think helps with their safety.

Interviewer1:13:18
Umm, thank you.
Umm.
Any other thoughts about gender inclusive posters or decoration?

Participant 3 1:13:29
Not at the moment, I think.

Interviewer1:13:31
That's fine.
So thank you so much for all your thoughts.
So those were the six recommendations from the literature.
Are there any of those that surprised you?
I can run through them again.
If you need as well, yeah.

Participant 3 1:13:50
Yes, please.

Interviewer1:13:52
So it was training, asking pronouns, asking preferred terms for anatomy, electronic medical record systems, documenting pronouns, and the gender inclusive posters and decoration.

Participant 3 1:13:55
Umm.
I guess I hadn't really thought about the actual like documenting part.
It's not necessarily a surprise because we had like, when we talk about electronic records, I wouldn't say I was surprised, but yeah, that's just something I hadn't really thought about before.

Interviewer1:14:25
Hmm.
And is there anything that hasn't been included in those recommendations that you would have seen as a priority for care provision?

Participant 3 1:14:45
I'm thinking.

Interviewer1:14:47
That's OK.

Participant 3 1:14:55
Don't think so at the moment.
Might come up with something tomorrow.

Interviewer1:15:02
That's fine.

Participant 3 1:15:05
Priorities...

Participant 3 1:15:14
I don't you think so.
So I guess.
Thinking more as well about like.
I don't know why but.
With anatomy and things like that obviously on.
Labour ward and things.
Would people want to talk about oxytocin, like, is that something that?

Participant 3 1:15:48
Being you know that.
Female love hormone.

Participant 3 1:15:56
I don't know, that's something else to consider as well?

Interviewer1:15:59
No, that's a really interesting thought.
Do you think in terms of how healthcare professionals discuss the promotion of oxytocin or just as a concept?

Participant 3 1:16:16
Just a random thought, just as a concept I guess.

Interviewer1:16:19
Umm yeah.

Participant 3 1:16:27
Overall, I don’t think so.
I think you know in terms of, yeah, the documenting, asking about programs and anatomy.
Yeah, the main things.

Interviewer1:16:41
Umm.
Is there any of those that you see as being more important than the others?

Participant 3 1:16:55
I say the anatomy part would be I feel like would be.
I guess it's difficult.
Item and important as in.
For the for the safety of their care.
I'd say that would be the one I'd wanna hash out with them.

Interviewer1:17:22
Hmm.
And.

Participant 3 1:17:25
Uh, if I was looking after them, I guess.

Interviewer1:17:28
So that would be something that would be more important to discuss?

Participant 3 1:17:35
Yeah, if that was something.
They were wanting to do then.
Yeah, I feel like I would want to do that.

Interviewer1:17:40
And which of these do you feel is being done?
Or is something you have seen in practice?

Participant 3 1:17:57
I've not really seen a lot in practice.
I'd say umm yeah.

Interviewer1:18:00
I guess you've spoken about training being done, so that's really, really positive.

Participant 3 1:18:08
Umm yeah.
Yeah, there's definitely training coming out.
That's good.
Not sort of, but yeah, that was more through my uni training than within like my employer setting.

Interviewer1:18:29
So no continued professional development training that you've seen?

Participant 3 1:18:35
No, it's like not as like your statutory mandatory stuff.

Interviewer1:18:41
Umm.

Participant 3 1:18:42
Haven't seen it like as part of that.

Interviewer1:18:45
Hmm.
Thank you so much, [participant’s name].
Any other thoughts or comments or anything?
Anything else you wanted to add?

Participant 3 1:18:57
Don't think so.

Interviewer1:18:59
So, so much for your time.
And if you're ready, I'll end the recording now.

Interviewer stopped transcription

# Participant 4 Transcript

24m 4s

Interviewer0:04
OK.
So that started now so.
So this study is exploring healthcare professionals experience, knowledge and attitudes to care provision for transgender and gender nonconforming pregnant people.
Did you have a chance to read the information sheet?

Participant 4 0:20
Yep.

Interviewer0:21
Amazing.
Do you have any questions?

Participant 4 0:23
No.

Interviewer0:24
OK.
And are you happy for me to record?

Participant 4 0:26
Yep.

Interviewer0:27
OK, amazing.
So what is your professional role?

Participant 4 0:33
I'm midwife, rotational midwife.

Interviewer0:35
Great and in which region or hospital or whatever you're comfortable with.
Do you work in?

Participant 4 0:41
In [location].
So it's [hospital trust name].

Interviewer0:44
Amazing.
And how long have you been in your current role?

Participant 4 0:48
Um.
About nine months.

Interviewer0:53
Amazing.
And what would you describe your gender identity as?

Participant 4 0:58
Female.

Interviewer1:00
So, just looking at some background experience.
Have you had any transgender or gender non conforming pregnant people that you've cared for, or at your hospital?

Participant 4 1:11
And not the pregnant person, but the partner.
I've cared for family as a whole that's had a transgender person in the family.

Interviewer1:22
OK, amazing.
And in general, what do you feel are the priorities for midwives and other healthcare professionals when providing care for transgender people?

Participant 4 1:35
What are the priorities?

Interviewer1:37
Hmm.

Participant 4 1:38
Umm.
Yeah, I didn't really know what you mean by that.

Interviewer1:47
So what do you think's important when providing care for them?
And it can be that there's nothing different from regular care.

Participant 4 1:55
I'm yeah.
Suppose you just.
You would just treat them as a normal person or normal family, but.
Probably for them and for health care professionals is knowing how to address them and how they want to be addressed and getting that correct and not offending people.

Interviewer2:16
Yeah.
Absolutely.
Umm.
And do you feel that healthcare professionals have support in knowing how to provide care for trans and gender nonconforming people?
Or do you feel,
Do you feel that more support is needed or there's currently enough support?

Participant 4 2:35
I would say probably more support is needed and there's not really spoken about.
I don't think in our training it was covered and training days and things.
It's not really.
Don't think it's really addressed. Ever.

Interviewer2:49
OK.
And and can you describe any times where you feel that there hasn't been adequate care for this patient group?
It's OK if not.

Participant 4 3:02
And no, I don't think I've seen it or anything.

Interviewer3:07
OK, that's fine.
OK, so from the literature review, there's been six recommendations and that that we've chosen to include based on the frequency of their appearance.
So it's in no particular order, but I'm just gonna present the recommendation to you and then just tell me what you think about it.
And we can talk through it in a bit more detail.
Is that alright?

Participant 4 3:29
OK. Yeah.

Interviewer3:30
OK, so the first recommendation is to have gender inclusive posters and decorations in hospitals and ward environments.

Participant 4 3:41
OK, So what things and that decorations like, obviously, maternity units would normally kind of pink or purple.
Is that the kind of thing? Yeah.

Interviewer3:49
Umm yeah.

Participant 4 3:53
And again, I don't think there is really any posters or anything about it.
I've I've not.
I can't think of a time I've seen really anything about caring for transgender families or people.
I think uh, the only thing I can think of actually is in our like monthly statistic posters, they'll put the amount of women breastfeeding or chest feeding.
So that is kind of inclusive, which is probably the only thing that I've noticed.

Interviewer4:25
Yeah, that's really that's notable.
That's fantastic.

Participant 4 4:27
Yeah.

Interviewer4:29
And so yes, posters and decorations.
How feasible do you think it would be for your hospital to have inclusive posters and decorations?

Participant 4 4:41
I mean.
I think they've got to pick in terms of like the colours of the walls and things on and like decorations and posters, they're they are aimed women and female, I think probably what you'd say like and the gender at birth being female, that's who that aimed at because obviously we care for people that have a uterus and breasts, that lactate and things.
So I think.
When you see posters about, for example, breastfeeding, it does normally say breastfeeding rather than chest feeding and colours on the walls.
I think normally they try and pick something kind of relaxing, comforting.
I think I reckon they probably could change things, but then it's difficult because I suppose you can't please everybody.
And we do, I mean, the majority of people we care for identify as female and are born female.
And I think that's that's what they're normally cater for.
But I think obviously the world is changing and they will have to change these things eventually.

Interviewer6:10
Umm, how much does having inclusive posters and decorations align with your current practice?

Participant 4 6:20
Umm.
As in, what effect does it have on like what?

Interviewer6:26
Yeah.
And how much does it align with?
Maybe your philosophy of care or your organisations philosophy of care.

Participant 4 6:33
Umm, I suppose everyone wants to be inclusive so.
Can you say a question again?

Interviewer6:42
OK, so how much does having gender inclusive posters and decorations align with the with your current practice or your organisations philosophy of practice?

Participant 4 6:53
OK.
I say it is it.
It is important for everybody and it should be important.
And that's of that.
The aim of the care we give is to be an ordinary, non judgmental and to be kind and caring to everybody and to meet everybody's needs.
So it it would align with the philosophy of care and the hospital like trust, values and things it would, it would, I think it would be important to include it.

Interviewer7:25
OK.
And and so I think we've spoken through some of the constraints to that as well.
So that's fantastic.
And so one of the other recommendations is asking service users, their pronouns, what do you think of that?

Participant 4 7:38
Umm.
And I think we should so that we're not offending anyone.
It can be something at the booking appointment.
I mean, normally we take partners details and name and phone number and things.
So we should just say what are your pronouns and I have noticed some at some healthcare professionals when they do like a send all email, they will have pronouns on like on the.
Is it like the email stamp?
Forget what it's called, but the bottom bit, that's just generic that they have that on there, they'll say like she/her or he/him for example.

Interviewer8:18
Amazing.
And do you think that asking service users their pronoun is feasible for healthcare providers?

Participant 4 8:26
Yeah, I think it's very simple thing that we could do.

Interviewer8:28
OK, amazing.
Do you think that any tools, resources or information would be needed for for healthcare professionals to start asking pronouns?

Participant 4 8:40
Yeah, I think like a guide on how to address it politely and and respectfully, would be good.
I think maybe I don't categorize like old midwives, but maybe they're a bit more traditional in their thoughts and they might not really understand the sort of like.
Kind of
What the pronouns mean if there if maybe have like old fashioned thoughts.

Interviewer9:09
Umm.
And would asking service users that pronoun?
Does that contribute to safe care?

Participant 4 9:22
Yeah, I would say so.
I think if you.
If you think of it as you were treating them with dignity and respect, they're more likely to want to integrate with their care, which does lead to safety and care.

Interviewer9:38
Thank you, umm.
And then one of the adjacent recommendations was in addition to asking service users about their pronouns, was also to ask them about their preferred terms for anatomy and their bodily functions.
So this is something you've mentioned already with chest feeding, but other examples could include instead of saying vagina or vulva, pelvic opening or front hole, depending on the person's preference.
And there's obviously other examples, certain times of bodily functions.
Some people wouldn't talk about their period, but they might use a more medical term such as menstruation.
And what do you think of asking service users their preferred terms for anatomy and bodily function?

Participant 4 10:23
I think it's something that you can do, but I think it would take a lot of time to integrate that because...
Pelvic opening might not necessarily be seen as a medical term for it.
It is more of a preferred term, same as breast and you have breast tissue, not chest tissue, but obviously so I think probably like in terms of medical things to clarify what you're talking about, it would be hard to integrate that.

Interviewer10:54
Absolutely.
Umm, so how do you how feasible do you feel that would be?

Participant 4 11:02
I think it would be.
Feasible for...
Like when you're doing one to one care and you know the patient well, it's a bit like I don't want to sort of like make it seem like a smaller subject.
But people, when people want contractions to be called surges, I think medical professionals really struggle with that because we medically we call it a contraction.
And I think it'd be the same as saying like pelvic opening.
Your natural instinct is to say vagina or vulva or something bit more.
Sort of medical.
So I think it would be quite tricky to integrate that in overall care, but probably one to one care less tricky.

Interviewer11:51
Thank you.
And what tools, resources or information do you think would be required for healthcare professionals to be asking service users their preferred terms for their anatomy?

Participant 4 12:08
I think it could be integrated into like a study day.
So you could have like a whole session.
Umm, discussing it and possibly like reminders.
I know we have a we have, I don't know about where you work, but we have quite a lot of like ... door notices.
So things like "barrier nursing" and you could have like a little something that goes on the door.
I know some hospitals as well.
I think on boards that have something above the bed that says the patients name or preferred name.
Umm, normally used for like a nickname rather than saying their full name if that's what they like to be called.
So you could have that and then the pronouns like above the bed.
That's just like you're looking at the patient and so above their head is a physical reminder.

Interviewer12:59
Amazing.
That actually leads on really nicely to one of the other documentations.
The other recommendations, which is about the documentation of the correct pronouns for for a person and what would you think of this recommendation and how would that look to you?

Participant 4 13:12
Umm.
Um, something like?
On wristbands or any like identification label, you could have you have the name and the date of birth and everything.
You could have the pronouns on there and then it's just that is repeated in so many areas and we we get people to confirm those things.
You could confirm the pronouns as well.
Possibly just say your then it's a visual reminder, isn't it?
Cause you're not gonna remember everybody's that you come into contact with, but if if someone is specific about them, that is very clear reminder.

Interviewer14:00
Umm do you think that documenting correct pronouns would be a feasible care provision?

Participant 4 14:07
Yeah, I think so.
Like on like I say on, along anything with the name can be next to it or.
Yeah, I think I don't think that's a tricky thing to do.

Interviewer14:20
OK.
And I I guess similar to asking service users their pronouns in the 1st place.
Do you feel that?
What?
What tools, resources, information do you think healthcare professionals would need to start documenting pronouns?

Participant 4 14:38
Umm.
Like a, a highlighted area or box that you have to fill in when you come into contact like the first point of contact like a booking form, it would be on that which would you know like translate into the rest of the and the notes or.
Yeah.
Yeah, I think that would be it really recently.

Interviewer15:08
Yeah. OK.
Yeah.
Again, that leads really nicely onto the last recommendation that that came up in the literature, which was gender inclusive and electronic medical record systems.
So for example, on most electronic medical systems, the sex is recorded, but you don't necessarily have an option to record gender.
Umm.
And if someone has legally transitioned and and they are on the electronic medical system as a male patient, then they cannot be admitted to the labour ward, which can cause complications.

Participant 4 15:50
Ohh.

Interviewer15:53
This is a bit of a bigger question because it's more on the IT side of things rather than on one to one.
Healthcare professional care.
Umm, but what do you think of making our electronic medical systems more gender inclusive, so allowing sex to differ from gender and allowing male patients to be admitted to the labour ward?

Participant 4 16:16
Yeah, I think it's important.
It's also important to know.
If somebody has transitioned again so that you, you're addressing that and that could be something and like the adds to their medical record, but also it's important to see that, that gender that they identify as.
So I think it shouldn't be completely like ohh, you've transitioned, so there's no record of your your sex at birth because it's still important to know that I think.
But I'm surprised.
Actually, I didn't realize that you couldn't be admitted onto, so that would be like the same in the main side, like a female ward.
They probably wouldn't be able to be admitted into like a female only award may be.

Interviewer17:06
To be honest, I'm not sure.
I haven't looked at that, but that would be something interesting to OK.

Participant 4 17:09
Yeah.
Yeah, because.
Yeah, I mean they need to.
They need to have the right care, and if that is on a labour ward, that's what, that's where they need to go, isn't it?
They can't for an IT fault.
That seems crazy to but change their care essentially.

Interviewer17:25
Yeah.
Absolutely, and a bit of a bigger question as well.
Um, kind of outside the scope of just our care provision.
How feasible do you think improving the electronic medical record systems would be?

Participant 4 17:44
Umm.
Yeah.
I mean I they they make adjustments all the time, don't they? To update?
Different things on there.
There's digital midwives.
There's big IT departments and hospitals.
I think that that's something they may be able to do.

Interviewer18:01
Okay, and we use spoke about this a little bit already with allocating the correct care provision, but how much does the gender inclusive electronic medical record systems align with safe care provision?

Participant 4 18:21
To say that again, sorry.

Interviewer18:22
So how, how?
How much do you feel that gender inclusive electronic medical record systems would ensure or provide safe care?

Participant 4 18:34
UM, well, it's their medical record.
Everything needs to be accurate on there, and so it whatever's on there should reflect the person as a whole, really.
If, if if it doesn't reflect their medical record accurately, then you're not gonna be able to provide safe care for them.

Interviewer19:01
OK, amazing.
Thank you.
So the last recommendation from the literature review is training as a part of continued professional development, specifically on cultural competency.
So this would be like about what it means to be transgender.
Umm.
And then also training on sexuality and gender diversity. What do you think of of this as a part of continued professional development?

Participant 4 19:38
Yeah, I think it is important.
And like I say it's you know, the the world is changing and and people's views are changing.
Umm and I think.
Everybody should be on the same level, umm.
Of knowledge, of knowing how to care for ... is the same as you would, like a diabetic study day, like you should all be able to give the same standard of care for somebody with diabetes.
So you should be able to understand anybodys care needs and if that is slightly different because they are transgender, we should be able to understand their needs and be able to provide for them properly.
And so I think a study day or like training day, would be important I think so that you know everyone's on the same level of knowledge.

Interviewer20:37
Umm.
And how feasible do you think it would be to have this training?

Participant 4 20:44
I mean, we do loads of training, so don't see why like one more thing wouldn't be a problem.

Interviewer20:51
Hmm.
And what tools, resources and information do you think people would need in order to provide the training?

Participant 4 21:01
I think an like a personal account of somebody that has somebody that's transgender that has access maternity care.
Their point of view would be really amazing to hear because it's all well and good, like reading something or watching.
I don't know, watching some like a video resource, but if you can actually hear their point of view and actually understand their needs in an open way, and also probably have a, like an open and like an open non judgment question thing because people will have questions about this subject and they will maybe be a bit confused at the beginning as to what... Because really you're providing the same care aren't you shouldn't really be changing it that much, but there might be a few adjustments that people need.
So it's important to understand that from someone that's access to care.

Interviewer22:02
Thank you.
And do you?
How would you?
How much do you feel that training would contribute towards safe care provision?

Participant 4 22:14
I think it would contribute a lot to safe care because like I say, you should.
You should understand people's care needs properly to be able to care for them well and safely.

Interviewer22:24
OK.
Thank you.
And dear, how much does it align with the current at the philosophy of care within within your hospital or in your practice?

Participant 4 22:37
And.
But I'm pretty sure our values and like safe, kind and excellent.
So really it does fit into all of those, doesn't it?
Because you want to provide safe care, you want to excel in caring for people with additional or like, different, possibly different needs.
And you wanna be kind to everyone.

Interviewer23:01
Amazing.
Thank you.
And, from.
So those were the recommendations that came up most frequently in the literature review.
Did any of those surprise you?

Participant 4 23:12
No, I wouldn't say I think that things that we should be doing already, but perhaps we're probably not.

Interviewer23:19
That's fair enough.
And is there anything that I didn't mention in those recommendations that you would have expected to hear or think would be important for midwives or other healthcare professionals?

Participant 4 23:30
Um no, I don't think so.

Interviewer23:33
OK.
Amazing.
That's really good to hear.
Umm, so those are all my questions.
Do you have any other comments or things you think would be important and in relation to the experience, knowledge or attitudes to care provision that you've seen?

Participant 4 23:54
No, I didn't think so.

Interviewer23:55
OK, amazing.
Thank you so much [participant’s name].

Interviewer stopped transcription

# Participant 5 Transcript

1h 15m 10s

Interviewer started transcription

Participant 5 0:06
Thank you.

Interviewer 0:06
OK, so the recordings on now, so this study is aiming to explore healthcare professionals experience, knowledge and attitudes towards the care provision for transgender and gender nonconforming people.

Participant 5 0:10
Yeah.

Interviewer 0:20
Have you had the opportunity to read the information sheet?

Participant 5 0:23
So I'm sorry. I haven't.

Interviewer 0:27
That's OK.
Are you still happy to go forward with the information you have, or do you want me to go through?

Participant 5 0:31
Yeah, there are more than happy to go with the information that I have.
Think you've explained everything really well.

Interviewer 0:38
OK.
Thank you.
And are you happy for me to record the audio and visual?

Participant 5 0:42
Yeah, absolutely.

Interviewer 0:44
Amazing.
And just to reiterate that the recording will be transcribed and then the originals will be deleted.
So your face won't be in the research and your name won't be ascribed anything that you said.

Participant 5 0:55
Yeah.

Interviewer 0:57
And so just some demographic questions before we get started.
Any questions from you?
Are you happy to go ahead?

Participant 5 1:02
No, no, no.
All good.

Interviewer 1:04
Anything.
And so just in demographic questions, what is your professional role?

Participant 5 1:09
So, midwife.
So [specialty role] midwife plus labour ward sister.

Interviewer 1:14
OK.
And in what region do you work in?

Participant 5 1:17
[Location].

Interviewer 1:19
And how many years have you been a midwife?

Participant 5 1:23
UH-14.

Interviewer 1:25
And how long in in the current role?
So [specialty role]?

Participant 5 1:29
Ohh, eight months.

Interviewer 1:32
I'll amazing.
And how would you describe your gender identity?

Participant 5 1:37
And I would be.
Just female, she/her.

Interviewer 1:43
Female.
OK.
Thank you.
So just a bit of background before we get into the literature.
Have you had any experience working with transgender pregnant people?

Participant 5 1:53
Yes.

Interviewer 1:54
Can you tell me a bit about that experience?

Participant 5 1:58
Yeah.
So overall was a really good experience and over arching, I think the most striking things were that I literally had no idea when you read some maternity notes, how… I don't know what the word is, but how, like at one point they even talk about husband.
The fact that my service user, what I was calling my client was a pregnant but identified as male.
There was so much that talked about “Mummy”, about “bonding with mum” about breastfeeding and given that he'd had a bilateral mastectomy. It was just a language, was incredible and things that I hadn't really noticed before all of a sudden looking at them with a different set of eyes.
Yes, it's amazing.
You know, every day what we write, what we read, leaflets that we give are all very much led to “Mummy”.
But there's even one part in our [trust name] medical notes that talks about the husband. It's go back to the 1960s a little bit.

And the computer system, my client lives as a male and is registered on the NHS spine as a male. However, we couldn't add a pregnancy to a male and it's just not part of the system. Doesn't let you do it.
So you know, we obviously had to add him a second record as a female link it to his male client, which, you know, I sort of saw that as a little bit of a risk.
You can imagine if we'd needed to get urgent blood in a MOH [maternal obstetric haemorrhage] situation.
I think that would have been difficult.
It would be easy to get blood with a wrong MRN [medical record number] and the same name.
Kind of scenario, so that was something we had to look at.
But I don't think it really got resolved, I think it was just a computer says no, we can't change this.
It's and that is a national problem.
And yeah, and also sort of attitudes, I think that you know, I thought the staff would brilliant and actually my client had a mainly really good experience and you know, was quite that grateful that we sort of really took on board and were quite honest and open and we did have a few slip ups on along the way, but the staff were mainly, you know just, they were… I think my client really quite accepted that people were gonna make slip ups and you know call him by calling by the gender that he didn't identify with.
But there were a few blinding ones that were quite embarrassing, like, but mostly it was people calling him “Mummy”, not calling him “she”. If you know what I mean?
It was sort of his role as a pregnant person.
He was gonna become a “mummy” and not a “daddy”, but was…
I think that was the one that people found the most difficult thing to take on board and also a massive lack of sort of resources.
*[The authors have made the decision to redact this information from the transcript due to the risk of identifying the person described].*

I sort of reached out and found a really great inclusivity midwife in [location].
You may meet.
Meet him on your studies.
Better he was. He was really great.
Sent me lots of, you know, like LGT… L… LGBTQ+ information that I could pass on *[The authors have made the decision to redact this information from the transcript due to the risk of identifying the person described].*
I mean, this isn't our usual client group, so that was good to find sort of links and you know I will be better prepared if this is… If I have another client like this which is you know, uh, yeah.
So that that's it really, I don't know, it was a bit… On the negative side, I think it was a little bit of the curiosity, but almost a bit… Everybody was asking about him, whereas actually he’s just another person in our care. He's a human. It's not different, but everyone was very fascinated. And you know, in a way that's interested, but also, you know, when you came in for his [caesarean] section, it was almost a bit like, please keep this on a “need to know basis” because I don't want people, you know, who were just… “want to see, want to see” you know… what the situation looks like.
I think you know, just to sort of keep things quiet was quite difficult.

Interviewer 6:33
It sounded like potentially a little bit of a novelty?

Participant 5 6:35
So that was.
Yeah.
Yeah, exactly.
And it wasn't meant in a bad way at all, but yeah, essentially it was some, you know, like, you know, we were all interested in the more unusual cases sometimes because we're curious people.
We're healthcare workers, but you know, sort of to keep interest.
The interest was quite a lot in this this case.

Interviewer 6:59
Umm.
And you said a little bit about the MDT [multidisciplinary team] working, how was that and were there any issues with the multidisciplinary team?

Participant 5 7:10
Actually I thought the multidisciplinary, I thought everyone was fantastic and I think people were really open and did their best, even if that wasn't their natural… natural you know, “way”.
But you know, I thought I thought people were very interested and you know, and I.
Yeah, I didn't meet anybody who was negative or didn't do their best.
Or… People were worried I think that they'd say the wrong thing.

Interviewer 7:43
And what was this your first experience caring for a transgender pregnant person?

Participant 5 7:50
Yes a pregnant person.
But I was nursing before I was with, with.
With what I have looked after transgender clients before, but yeah, there's my first transgender pregnant person I've looked after.

Interviewer 8:03
And it was in a case loading capacity?

Participant 5 8:08
Even if he was booked under [specialty team] and but to be honest, that wasn't his “headline” by the end of his pregnancy. His headline… so, but I think as he was complex. [*The authors have made the decision to redact this information from the transcript due to the risk of identifying the person described]*
I think I did his booking and had the difficult conversations about the computer system and the second [medical record number], and you know, we had choices.

He could have kept his paper notes and not been on the spine at all.
But you know the responsibility of never losing your paper notes and having no other records is also quite a thing.
So you know, and that you know, just personally we had a nice relationship.
So I sort of, saw him for his, you know, for a lot of his antenatal care.
*[The authors have made the decision to redact this information from the transcript due to the risk of identifying the person described]*

But yeah, I sort of kept in touch with him.
And saw him postnatally as well and did postnatal follow up phone calls so.

Interviewer 9:31
It sounds like so… good communication between the multidisciplinary team, but also good communication with the client and giving the options of how information is stored.

Participant 5 9:43
Yeah.
So I mean it's a lot with all our all our clients, it’s transparency, isn't it?
It does make difference, but I mean he, you know he, [client’s name].
Sorry, I shouldn't mention his name.
But, and his partner, they were both so um… open.
And you know, and they understood that it wasn't gonna be easy for everybody.
So they actually made it very, very easy for us.

Interviewer 10:14
OK.
*[The authors have made the decision to redact this information from the transcript due to the risk of identifying the person described]*
But in general, do you feel there's any specific priorities for healthcare workers when working with transgender pregnant people?

Participant 5 10:39
Well, I don't know.
I never… after my first meeting, I never really thought of him as transgender he was just one of my, you know, people I looked after and we did what we needed to do and he never asked for anything extra than what we added.
*[The authors have made the decision to redact this information from the transcript due to the risk of identifying the person described]*

So you know, but it was a consideration and for none of us to have that sort of resource or that also understanding. That's not a situation that I may ever come across again in my career, but knowing where to find your resources, I think that was would probably be a good thing.

Interviewer 12:41
So you've touched on loads of things there, sometimes where there's been really good care and actually sometimes where some more information is needed and what's really interesting is you actually touched on loads of the recommendations from the literature as well.
Any other thoughts or comments about the experiences you've had?

Participant 5 13:05
And I had a… actually had a really good experience.
I really enjoyed looking after him and you know, it was nice.
It was.
It's just different challenges, but yeah, they were an absolute pleasure.
And you say if challenges I did have we was people were very, very interested.

It's hard not to not.
I mean, not that you'd break confidentiality, but that kind of almost.
You know, bordering on gossip sometimes, isn't it?

Interviewer 13:41
Umm, so it sounds like once you knew this person, there was a degree of like normalizations, you said after the first meeting it became a lot easier for you to think of this person as a male and potentially do you think the midwives who had lots of questions hadn't had the same experiences perhaps?

Participant 5 13:55
Yeah, maybe.
They're just interested and you know it's not a bad thing to be interested.
*[The authors have made the decision to redact this information from the transcript due to the risk of identifying the person described]*

It's like when.. when is… And you know to have sort of more knowledge and information as a trust would… But you know at the same time, you got to see where we work.
We work in [trust name].
We don't have this case load like very often.

I think this is the second time I've seen… looked after a transgender male at [trust name].
The first one didn't stay with us very long.

Interviewer 14:47
OK.
Do you think that was just their personal circumstances, or related to the hospital?

Participant 5 14:50
Personal circumstances. It was in that 2020, it was lockdown. I was on labour ward then. I just met them in [triage] once, and then they transferred their care.
So I don't know what happened or any outcomes or… it was brief.

Interviewer 15:06
Yeah.
OK, so going on, just some questions about you and about the care you provide.
And again, this is as much as little detail you're comfortable giving and as just like a bit of a primer of a before we go into the recommendations.
Could you like describe your philosophy of care or your priorities when you're providing care?

Participant 5 15:32
Is this just for anybody to look after?

Interviewer 15:35
Yeah, just in general.

Participant 5 15:38
Well, I mean, when it… for [specialty role], it's always good to have them at their.
I know it's a bit cliche, but they need to be at the centre so that it doesn't need to be about their medical centre, their medical problem.
So you know, so obviously with [named consultant], who's the consultant. And she does the doctor, she's the doctor. I'm the midwife.
So although I understand a lot of these medical conditions, I look at it with a bit more of a holistic. You know, sort of looking how their medical condition affects their pregnancy in more than what their medical condition is and it's working a way through recommendations that they may have.
They may not… You know they… vaginal birth may not be suitable, so it's sort of helping them come to terms and you know, and I sort of like to do their... See them on labour ward and to see them postnatally.
So you know, a bit continuity and you know, just putting them at the centre rather than making it about their medical condition or about their safeguarding.
And quite often our [specialty role] ladies have multiple teams.
So you know, with somebody complex, it's just sort of being there as sort of the glue, the glue of the… of the many professionals that they see.

Interviewer 16:55
Umm, that's a really nice way to describe it, and it sounds like you touched on like the holistic care as well seeing them as a whole person.

Participant 5 17:04
Yeah. Yeah.
Uh, actually, lots of more people you see in [specialty role] are often complex and you know, and a lot of them have had a lot of medical input through their whole lives, so they'll have different sort of outlooks to you know the, you know, typical low risk case load.

Interviewer 17:24
Umm.
And also touched on like that individualized aspect of it in terms of like mode of birth and things.

Participant 5 17:31
Yeah.

Interviewer 17:32
Yeah.
Thank you.
And then?
In an ideal world, so if there weren't any constraints on your practice or work environment, do you think your philosophy of care would be any different?

Participant 5 17:50
Yeah, umm...
I don’t know if this is philosophy. I mean, I'd love to caseload some more and do more labour care.

Interviewer 17:57
Hmm.

Participant 5 17:58
That's it.

Interviewer 18:00
So full continuity?

Participant 5 18:02
Yeah, yeah.
Is that what does that answer your question or not?
I guess that.

Interviewer 18:05
No, no, no.
That's fine.
And these are kind of a bit existential, so it's OK if it's not a solid answer and then sort of last two parter, bit wider question is how would you define safe care?

Participant 5 18:24
How do I define safe care?
Well, I mean you could say “healthy mum, healthy baby”.
That's the end result, but you know again it's sort of, it's holistic, isn't it?
It's making sure people come out the other side with mental health and with hopefully, even though it may not be a, you know, a waterbirth coming in at 8 centimetres, but you know an experience that they feel prepared for and.

Interviewer 18:50
Umm.
So touched on education, emotional safety and the physical safety?

Participant 5 18:57
Umm yeah.

Interviewer 19:00
Great.
Thank you.
So we we've touched on some of these with, with the experience you've had actually which is so fantastic.
Umm, but there were six recommendations that have come up in the literature review, so we'll just talk through those if that's OK and just any thoughts or comments you have about them.
So the first one is something that you discussed quite a bit.
So the training, so this recommendation is training as part of continued professional development on cultural competency and sexuality and gender diversity.
And what do you think of this as a recommendation for healthcare providers?

Participant 5 19:34
I'm I mean that that's definitely a lot of curiosity, definitely.
I think it would be, it would be good, but I say yes.
As far as I said [location], but it's not like what we see a lot of.
There's lots of other things that we probably see a little bit, a lot more commonly that we need more training on.
So it's a bit of a tricky one.
Actually, this is an unusual case for us, but I think there’s a sort of need for training, but and the staff are so open to it like they were fantastic.
Like everybody I spoke to was so like, I don't know, you know… I emailed people, say you know that the antenatal team, like things like the toilets, like just having a bit of consideration that he lived as a male, so to use a female toilets was not how he lives his life normally, and it's a bit like asking me to walk into a men's toilet.
And, you know, he was uncomfortable with that because he sort of was waiting until people went to sleep before he could go to the toilet.
And you know, they were great. They went, they went the extra mile without making him feel different. So I just thought the staff are fantastic.
So you know other training wise it's not, it's not you know that they need to… They see what needs to be done and get on with it.
But yeah, there's definitely a lot of curiosity.

Interviewer 20:56
Umm.
And it sounds like in the case as well, it was potentially rather than a lack of openness, a lack of resources?

Participant 5 21:03
Umm, yeah.
The toilets stuff.
The maternity unit is not the easiest but I mean we found him a side room which we are able to do that at [trust name] and the staff were really good at doing that.
But then it was a bit… because he wasn't the kind of person who wanted to be made to be different or wanted any special… He didn't want the red carpet to be rolled out.
He just wanted to come and have a baby like everyone else.
For that part, almost doing it without letting him know that we make a… we sort of had to plan and spend 3 days making sure that [a side room] is free, but I could do that without him knowing that I did that.
If you know what I mean, you probably thinks he comes into [trust name] and get a single room and but you know, we we've made that happen.

Interviewer 21:53
Amazing.
I want… So we talked about the physical resources but also the, maybe educational resources or the guidelines for care provision saying about how health professionals weren't sure about how a caesarean would affect a phalloplasty later in life.

Participant 5 22:13
Yes.
I mean, yeah, that's absolutely.
That sort of was obviously not my remit.
My remit, but it was.
Yeah, that was people just didn't know.
So you know to… it's sort of speaking to a surgeon who does this surgery and I guess there's not that much data. It's not like, you know, a gallbladder is it? It’s something that like for a woman who's had a caesarean. Women, men or client, I mean that goes for a phalloplasty like that must be rare occurrence.

Interviewer 22:53
Umm.
And so maybe if there was a need for additional education, you'd see it more as like the care recommendations rather than the concepts?

Participant 5 23:06
It would be a surgical recommendation. From surgeon to surgeon.
But you know, as a midwife, you kind of… if people are asking these questions, you need to know where to find the answer.

Interviewer 23:21
Umm so signposting for health workers?

Participant 5 23:25
Yeah.
Which you know, we've did find somebody in the end, but no, it's the signposting.
And the information because it's say, there's not… It's unusual, unusual case, I guess from the phalloplasty side and from the obstetric side.

Interviewer 23:42
So it sounds like training on the go was very much a part of this this example?

Participant 5 23:54
Yeah, we learned as we went.

Interviewer 23:56
Yes.
And if you were to care for someone again, do you feel like there would be any additional tools, resources, or information that could help it be more streamlined?

Participant 5 24:11
I think I sort of… now I have a contact.
I think I would probably immediately contact rather than you know, we found that the contact… you know there are midwives doing this but they aren't local but also very, very open to helping and offering advice.
And so that would be something I'd touch base with a lot earlier. Like this all happened right at the end when we knew we were going to have a planned [caesarean] section.
And so yeah, that… I forgot what you asked me.

Interviewer 24:46

About what resources would help with the care provision, really.

Participant 5 24:56
Yes.
Uh, so we… do you know what, I haven't read them, but he sent me… Various groups he could get involved with, because that was the other thing that was sorry, I keep thinking of things as we're talking but sort of offering antenatal classes.
But yeah, I mean what he didn’t do antenatal classes.
But you know it's really… They are a really good thing for pregnant people to do, like for sort of sharing.
I mean, he did have a lot of friends who had babies, so he wasn't socially isolated at all.
They actually weren’t difficult to care for because I knew he'd be fine. But you know if he had been socially isolated. How would I have sort of helped him in meeting other people… Like there's not… There's not anyway.
Then the person I spoke to had sort of, they weren’t local, but you know not that far away, things they could tap into.
And so that is all good information to have. Sort of more specialised.

Interviewer 26:08
So education for the clients?

Participant 5 26:10
Yeah.

Interviewer 26:16
And do you feel that the that training for health workers would make any difference to the safety of the care provision?

Participant 5 26:29
Maybe. I mean not physical safety, you know....
Yeah, you know, he was… He was going to get a good outcome because see was, you know, seeking care, had good consultant care.
Had you know… Mentally could have made things easier, I guess, but it would have been.
It would have been tricky.
I don't know. Actually, I don't.
I don't know the answer.
I don't know.
I think because we did have a good outcome and I had a lovely couple. They appreciated it and had a really good experience.
I don't know, we will see how things go down the line.
Won't we? See how he does postnatally…

Interviewer 27:18
Yeah. No, that makes complete sense.

Participant 5 27:25
I’ve only got him to base my… sort of… experience on.

Interviewer 27:30
No, that's OK. And it can also just be opinions, it doesn't have to be of things you've seen just in your experience of the NHS as well.

Participant 5 27:38
I used to feel a bit, but again, it's that difficult thing of making somebody different, isn't it?
But I used to feel a bit, like almost a bit sad when I used to see him sitting waiting for his scans in antenatal clinic.
*[The authors have made the decision to redact this information from the transcript due to the risk of identifying the person described]*
But then what do you do?
Do you, you know, put your points at the beginning of scan lists? But then also is that just start taking things a bit too far?
Like or like you know, I do… I do that for somebody you say for, you know, a wheelchair user who needs it, you know?
But they didn't need that.
I’d think, “oh wait, could we do anything different or could I improve this for him?”
But actually it's the it's NHS, isn't it?
Scan lists are busy and we've got to bend somewhere, or somebody does.

Interviewer 28:33
And that, that discomfort in waiting would do you feel that was more to do with, *[The authors have made the decision to redact this information from the transcript due to the risk of identifying the person described]*
Or was it to do with the like the fact he was transgender in that environment?

Participant 5 28:45
It’s that he was by himself.
It wasn't anything to do with that he was transgender. It was that his partner couldn't come with him, but then I just thought you're very vulnerable… I used to feel like he looked vulnerable.

He looked different is obviously not, you know, and I just think, “gosh, is everyone, like are the other women looking at him?”

Interviewer 29:07

Hmm.
And any other thoughts about training or otherwise we can move on?

Participant 5 29:14
Not really, I don't. I don't know.
I guess you could… We do our cultural [competency training] with [training leader], don't we?
You know, we do it now. It's a recommendation of Ockenden and embracing that we've now do cultural competency.
Yeah, it could, I mean… Include that in.
You know that almost with cultural competency, it's similar lines.

Interviewer 29:47
Yeah, if that training was done, who do you feel would be the most effective at delivering it?

Participant 5 29:57
Oh my gosh, I don't know. I don't know. I honestly don't know.
I mean, well, I'd love to hear, you know, like the person I was talking to in [trust location].
I'd love to hear him talk, because he does this all the time. He's, you know he caseloads transgender clients having babies.
I'd love to hear, and also as a transgender male himself, like, I'd be fascinated to hear him talk, but I don't know.
I don't know. I would have… I wouldn't feel qualified to do it.

Interviewer 30:35
So someone with a lot of experience?

Participant 5 30:39
Yeah, I'd say. It's something that is, I guess it's difficult to gain a lot experience in isn't it?

Interviewer 30:40
Yeah, absolutely.

And on that you said that they're a transgender male themselves.
Do you think that it would be important for a transgender person to be delivering the education or more important for the experience?

Participant 5 31:02
Are you a better midwife if you’ve had a baby? The answer’s no isn't it?
I don't think so.

I think you know, to have an empathy and an understanding is really important.
And you don’t have to be a transgender male at all. But you know to have some experience and some… Definitely empathy and.
They have to give the whole, but like, you know, like you, you could. You give it a lot. You've given this a lot of thought and exploring.

I don't think you could just stand up and deliver it.

Interviewer 31:40
Any other thoughts about training tools?

Participant 5 31:49
Not really, I’d say, having caseloaded, I don't know what would have helped.
To be honest, I don't know. Apart from knowing where to signpost.

Interviewer 32:04
Hmm, OK.
So one of the other things that came up in the literature was about asking service users their pronouns and what do you think of this as a care recommendation?

Participant 5 32:17
Well, I think it's quite good. I like it. I think it opens a conversation, doesn't it?

Interviewer 32:23
And is this something that in your experience also?

Participant 5 32:24
Most of our client, some people would like it, some people wouldn't, but I think most people would accept. But also you know like, you know, I've got my daughters, one’s 21 and one's 19 and that's normal for them.

You know as we get, you know we’re moving forward in maternity this is going to be completely normal, but it's not always normal for you know some of our older mummies. It's not what they'd be used to, but you know, I think moving forward, we'll do it for everybody anyway.

Interviewer 32:58
So in this example, you're thinking that the role of like, potentially a midwife would be like prompting and asking?

Participant 5 33:07
Yeah, at booking, like you know, “What pronouns would you prefer?”
We ask so many things at booking you know that would be easy just to add on.

Interviewer 33:18
And in the example, in your experience, was it something highlighted at referral or something that you found at that booking appointment?

Participant 5 33:28
No, we knew about it because it was… we had [electronic medical record system]. Because of the [electronic medical record system], so we knew about it because we couldn't add the pregnancy.
So we knew all about it before the booking and the admin staff had had to contact and.

Interviewer 33:44
And do you feel that it made it easier to have that prior information?

Participant 5 33:49
Yeah, definitely. It would have been a tricky booking, I think. Had I not been able to add a pregnancy, and had to do it on paper, and then there so much, I think I would have cried! Not because he was a transgender male, just because of the whole everything else that was, you know, the admin, the noise surrounding it.

Interviewer 34:09
How feasible do you think it would be to ask service users their pronouns?

Participant 5 34:18
Yeah, I think it would be so easy. We ask them so much worse, don't we?
You know, some of the things we ask. You know, and we...
Everything we ask you may not be relevant to you, but it's relevant to somebody.
So we ask everybody as a blanket and now, I don't know if you find, but I've never found anybody who thinks it's weird if you have asked if they've had genital cutting or… people just like appreciate that it's important for other people.

And I think that's probably with most of the things we ask at booking.

Interviewer 34:57
Yeah. And do you think that asking pronouns is something that's currently being done?

Participant 5 35:03
Not really.

Interviewer 35:06
And is there anything that you think would help healthcare workers to ask for people's pronouns?

Participant 5 35:14
Well, it would help if it, you know if you had to add it to a booking then everyone would get asked. But I don't think that, from my experience I wouldn't think staff would find that uncomfortable at all. We ask about domestic violence, we ask about so much worse.

Interviewer 35:34
And do you think that asking service users pronouns would impact the safety of care provision at all?

Participant 5 35:42

I think if they… if you know, it would either make people more comfortable to talk to about whatever they want to talk us about or it would, they wouldn't really care about it.
You always get one person who's like “why are you asking me this?’
Always, we have those people you know, and that's fine.
Yeah, I think most people, the vast majority of people, would not have a problem.

Interviewer 36:16
Yeah.
And so for some people say, it might create a bit of a disconnect, but for others could enhance that?

Participant 5 36:23
It's a very, very few.

Interviewer 36:27
Yeah. OK. And any other thoughts about asking for pronouns or any other situations you think it could be helpful?

Participant 5 36:36
How to mean?
I think with, I think with um… my recent client’s case, I think it would have been helpful.

*[The authors have made the decision to redact this information from the transcript due to the risk of identifying the person described]*
But you know, I think just having a bit… pronouns on the front of, I know we're going digital.
Well, you know, she may have not done that.

I think, well, I think it's more of… the midwife was more affected by it than [the client].

[She was] the more, the one who came off worst for the two of them.

Interviewer 37:40
So it sounds like that was a situation where the service user could advocate for themselves quite well, and it wasn't done maliciously by the midwife?

Participant 5 37:51
No, I think it was at 4:00 in the morning, half asleep, what they’d usually say, their normal patter, which obviously usually works for most people we have. But, yeah, that said, they apologised and the client actually found it hysterical because of the amount of apologizing and we all moved on.

Interviewer 38:15
So there's a degree of understanding that mistakes will be made, but it's more about the attitude of the work health workers potentially?

Participant 5 38:25
Yeah.

Interviewer 38:26
Yeah. OK.
And then kind of building on from asking about pronouns, something else that came up was asking service users their preferred terms for anatomy.
So you spoke about this at the beginning in terms of like a double mastectomy.
Umm an example would be someone preferring the term chest feeding rather than breast feeding or having preferred terms for their genitals.

What would you think of this as a recommendation, to ask people their preferred terms for anatomy?

Participant 5 39:01
Yeah, I guess so.
I mean, I did have the conversation about how he felt about vaginal examinations and I never even thought about to ask.
We talked about, vagina and penis.
I didn't ask if he preferred terms, um we talked about cabergoline because although he'd had his breasts, he didn't have breasts.
You know, I mean again, I had to ask, I didn't know the answer, but you know in case there's any remnants, could he still get… Could he actually still in fact produce milk and get mastitis?
I didn't know the answer and you know he was gonna bottle feed because he couldn't breastfeed.

But we never talked about chest feeding.
We talked about artificial feeding and we talked about cabergoline, to the fact that in case there was any tissue there. He could have had some ducts leftover which could have made him feel poorly, which you were obviously you didn't, definitely didn't want to feel poorly, so we gave you some cabergoline.
So we did have the conversation, but I used the terms I would always use with anybody I was looking after.
I don't know what I think… I don't know.
It's keeping things professional sometimes, isn't it? That’s your job, you're a health professional.
I don't know, I’m just thinking when my children were young and some of the terms people used to describe their children's [genitals], I don’t know if I’d feel very comfortable…

Interviewer 40:38
So potentially there’s a time and a place for medical language?

Participant 5 40:42
Exactly.
I think you know that's what we do, isn't it?
We are, even though we're trying to be the holistic people we are…. Medical, unless somebody actually gave me a… told me that this is what they wanted to.
I don't think I would generally ask.

Interviewer 40:58
So in terms of feasibility, do you see this one potentially a slightly less feasible than asking about pronouns?

Participant 5 41:06
Oh, yes.

Interviewer 41:08
Yeah.
And have you heard people using terms like chest feeding in ward environments or any discussion around it?

Participant 5 41:19
No, no.

Interviewer 41:24
So potentially not as prominent as the conversation around pronouns at the moment.

Participant 5 41:29
No, no.
And I mean that you get… I say, I've only had this one experience but again it was… we didn't really have to go there, you know there… it wasn't an option that he would chest feed.

Interviewer 41:49
But that was more his choice rather than anything that was?

Participant 5 41:54
It was medically you wouldn't have been able to.

Interviewer 41:57
Ah, OK.

Participant 5 41:58
He didn't have a… you know, his nipples weren't attached to anything.
So and sort of body image wise I'm pretty sure that wouldn't have been his choice anyway, but yeah, with him it was always artificial feeding.

But I like say I don't know, in a different situation I probably would have used the term chest feeding.
Because I've done my infant feeding study day. I know that's a term that he would have felt more comfortable with.

Interviewer 42:34
And so it sounds like potentially it's something that's being discussed a small amount? So you said in in the infant study day it's discussed?

Participant 5 42:45
Yeah, yeah.
In my last infant feeding study day. But it's not something I would talk about a lot.
And if you sign post then you, well, you know, if I'm in antenatal clinic, it's all breastfeeding, isn't it?
Any resources you signposting, so that's a natural term I would use, but again you know, I think things do and are changing aren’t they, but yeah definitely he would have been a lot more comfortable with that term but it wasn't ever anything we did talk about.

Interviewer 43:23
OK. And if health workers were to start asking about preferred terms for anatomy, what tools, resources or information do you think they would need to do this?

Participant 5 43:36
Oh my gosh, I can't imagine. I don't know. What? I don't know.

Interviewer 43:45
That's OK.

Participant 5 43:46
Honestly don't know.
I’d say I guess, at some antenatal, you'd have to just added some… You know the sort of checklist you do at each antenatal appointment.
At one point, maybe doing a birth plan.
It may be something you could ask? but you'd still be like, “what would you like me to call your vagina?” I don’t know, It just feels a bit… Feels a bit something you do to a small child.

Interviewer 44:21
Yeah.
Well, I mean, some of the examples would be some, some people might instead of the term vagina prefer front hole or pelvic opening.

Participant 5 44:30
OK, right.
I understand that, my mind went somewhere else.
Yeah, I suppose that could be… Could be something that, but it would be another of those things that you know if you're trying to avoid to say a word.
It's like when you're in, well for me, I've never been really been a birth centre midwife, but when we have people who don't want me to say the word “contraction”, that's all I ever feel like I say.

Interviewer 45:02
That makes you trip up a bit more?

Participant 5 45:04
And then you find you spend your life saying “really sorry” or somebody said “please don't tell me what you are on vaginal examination” and then you just somehow can't help it pops out, but I don't know. I don't know.
I’d say I've never heard that or seen it in clinical practice, but yeah… Yeah, sounds. I'm sure women would find that more comfortable.

Interviewer 45:35
And so you mentioned a bit about other language that can be changed to make people more comfortable than labour and you said you find that difficult as well?

Participant 5 45:46
It's not like I've got a problem with it. It's just, it's just sort of again, it's your patter, isn't it? What you do every day.
Sometimes difficult to not… to be different than what you're normally doing, which is something we all have to adapt and do our best, don't we?
But you know it's that kind of. And yeah, I think most midwives have that story.
Where they weren't supposed to say something, and that's all they could like say, like the words “surge” or “wave” and or you feel like you're like “contraction, wait, surge”
and all the words come out.

Interviewer 46:27
Umm and do you think that asking preferred terms for anatomy would affect the safety of care?

Participant 5 46:38
I don't think so. I don't think it would make a massive difference.
That said I think some women would probably really like it.

I don't think it would hugely change their experience.
I don't, I don't think.
But that that would be me. I don't know. Maybe it's cause I've never asked.

Interviewer 46:59
That's fine.
Any other thoughts about that one?

Participant 5 47:13
No, not really. I'm processing. I would have to have a think about it.

Interviewer 47:19
That's fine, and we can always come back to it as well.
And so again, building on something we spoke about earlier with the pronouns, one of the other recommendations was documenting the correct pronouns and what would you think of that?

Participant 5 47:34
No, I’m happy, more than happy to do that, I don't think… I think this is becoming a really common thing at the moment. We probably should be doing it more than we do already.

Interviewer 47:43

And so how feasible do you see it to document pronouns?

Participant 5 47:49
I think it would be very… I don't think it'd be difficult to do at all, to be honest.

Interviewer 47:54
Hmm.
What sort of places do you think would be helpful to have it documented?

Participant 5 47:59
Well, we're losing on notes. I would have said on the notes, but I can't say that anymore. And I'm not sure what the [electronic medical record system] is gonna look like, but maybe on the [electronic medical record system] report.

Interviewer 48:14
OK, and I think you kind of already mentioned this saying it's not done very commonly and have you seen documentation of pronouns in practice?

Participant 5 48:26
Not really, not really. Not at [trust name]
I think that's in notes from other hospitals.
With pronouns, I think.

Interviewer 48:40
And if it was to be done, what sort of tools or resources or information do you think that health workers would need?

Participant 5 48:50
And I don't think they'd need a need a lot.
I don't think so at all. We already ask gender questions at booking.
I don't think it will be any more difficult, asking what pronouns people, their preferred pronouns, and asking “are they gender they were assigned to at birth?”

Interviewer 49:11
And do you think this would impact the safety of care provision at all?

Participant 5 49:20
For some people, possibly like some people, possibly. Like sort of, I'm talking mental health, but I think it, you know, it may.
As I say, I’m not massively experienced, but I did mention one case and I think it would have, possibly would have changed an embarrassing situation, umm, but you know the client I was looking after, that didn't, you know that didn’t get to them when people made mistakes, but I have looked after other couples who have been upset when people, like a same-sex couple, who, somebody asked if their partner was there sister or such you know, and they got very upset by that.
I mean, I guess pronouns wouldn't really make a difference there, but you know, it's just documenting sexuality may prevent slip ups by staff that affect some people more than others, but I don’t know.
I'm not sure pronouns would massively help that to be fair, but.

Or though it may increase awareness and us not just to assume things.

Interviewer 50:29
So reducing assumptions and potentially handovers between staff?

Participant 5 50:39
How do you mean handovers like?

Interviewer 50:41
Appropriate information sharing that that that person present is their partner?

Participant 5 50:46
Yeah, yeah, yeah, I think so.
I think, yeah, I mean to be fair, in 14 years I've seen lots of people mistake, you know, I mean, I've heard a dad been a grandad. They were embarrassing.
It's always good to not have that kind of, you know, if we can raise awareness of not even just sexuality. But you know assumptions are a bad thing for all parties.

Interviewer 51:14
So reducing assumptions in general.

And how feasible do you think it is to document pronouns?

Participant 5 51:30
Yes, I say I don't.
I think it wouldn't be a problem.

I don't as expect. I think with new midwives coming in, this is completely normal for them. Again, I think people tend to get onboard really well with things, to be honest.
I think midwives have been open and accepting and taking on new things.

Interviewer 52:00
OK. Thank you. That's really good to hear and any other thoughts on that one?

Participant 5 52:09
What, documenting? I mean I don't know how the new [electronic medical record] system is going to work, so I can't quite see how it would look if you know what I mean.

But I think as far as the staff are concerned, they would, you know, it would… It would be fine.
It's not a big thing to document it.
You don’t have a write a big old… It's not gonna take hours like some forms, like the swab count that you have to document in six places, it would just be an add on to their name.

Interviewer 52:45
OK. And we'll move on now to one that knew you spoke about quite a bit actually the electronic medical record system.
So something that came up in the literature a lot was making electronic medical record systems more inclusive.
So exactly what you've said, allowing male patients to be admitted to the labour ward or have a pregnancy added.

Participant 5 53:00
Yes. It's not even just embarrassing. It's a clinical risk.

Interviewer 53:11
Umm, so you see this one potentially as impacting safety a bit more?

Participant 5 53:16
Yes, you're right.
Yeah, that is it.
It’s safety.

Interviewer 53:23
Umm and.
How feasible do you feel it would be to have this changed?

Participant 5 53:31
I mean, I only know what I was told and it's not.
It's not [trust name]’s system, it's the national spine.
So I'd say very unfeasible.

Interviewer 53:41
Umm.
And in the situation you gave the alternative was, to change that, create a new record for the person?

Participant 5 53:51
So that's what we did.
We created the same name with a different MRN number, connected to the male record was a female record with the pregnancy added to it. So we added any documentation for the pregnancy on female record, but he actually had a different MRN which was his male record that was used, you know, if he had to go into hospital for anything else, that would be his record.

So I can just see that this is gonna cause problems in the future as well.
I don't know how easy it is to remove that second record now that he's had the baby, so he's going to get this… This is going to be stuck with him for years, isn't it?
And having two hospital numbers to one person, as you can well imagine, like, just imagine going to blood bank having to get platelets that's at three in the morning.
I can't imagine that ever going smoothly or easily.
I did speak to them and explain that, you know, you speak to one person and somebody's… that person isn't working when you when you need it.

Interviewer 54:51
How did this this two records system does this affect?
How the records are viewed?
So for example, with looking back on the medical records from the pregnancy?

Participant 5 55:04
Ohh not at all.
There is that like the same, it's just that he has… So on the spine, he has his name and his MRN number as a male, but almost imagine a little arrow going off from his MRN number, with the different MRN number and a pregnancy record with him as a female.

Interviewer 55:25
OK, so it shouldn't affect future care interactions?
Say he does go for surgery later on?

Participant 5 55:32
Technically not, unless somebody accidentally prints out his wristband with his female MRN number, which then would be the same name in the same date of birth, but not the same MRN number. Do you know what I mean? It’s that blood bank situation would spring to mind the most. That's so yeah, it say it could be safety.
I mean, luckily he never needed any blood in this pregnancy, but…
I don't know what how to change that.

Interviewer 56:04
So we, we've spoken about safety, the feasibility and again this might be a question more for the, the IT experts, are there any tools or resources that you think could help make electronic medical record systems more gender inclusive?

Participant 5 56:29
Our [trust name] notes were absolutely awful, to be honest.
I actually did email [staff member] to say, can somebody just have a look at them?

To like… just with gender inclusivity in mind.
Because I think I even found the word husband.

Interviewer 56:49
Umm any other examples of that?

Participant 5 56:53
Well, like it's again.
You talked about it, but “breastfeeding”.
Umm. Yeah, “mums”, a lot of talk of “mum”.

And it, you know, without thinking, dad is pregnant, like it was, it was slightly embarrassing to be fair, we could have done a lot better, but we're changing the notes anyway, so.

Interviewer 57:17
So it sounds like things are in progress, which is fantastic.

Participant 5 57:19
But I think it will be slow. It will be slow, but I, you know, I think projects like you it's raises awareness and things slowly change but you some of the resources we've had are not new they've been around a few years and things move forward quickly.

But I think I did do a fair bit of feeling, slightly embarrassed about how non-inclusive things where, but I'd literally never even seen it before.

Interviewer 57:53
Umm, a bit of an eye opening experience as well?

Participant 5 57:56
Yeah, yeah.

Interviewer 58:00
Any other thoughts about the electronic medical record systems?

Participant 5 58:07
Not really, not really.
I mean that the, you know, the overarching ones were the two, two records, but.
No.

Interviewer 58:15
Umm, OK, thank you.
So I'm going to our… the last recommendation, which is something we've kind of covered in in terms of the notes, but also in terms of environment and it's to do with having gender inclusive posters, decorations and signage.
What would you think of this in in the maternity setting?

Participant 5 58:40
I love it. I think we should! We should celebrate diversity. It's good.

Interviewer 58:48
And what sort of things do you think would be important for these sorts of decorations or posters?

Participant 5 58:56
What would be important for posters?
Depends what we're trying to say. What are we trying to say? Just you know, what we're trying to raise… like we're awareness raising.

Basing it on my sort of experience. I don't know. I don't.
I'm trying to think what would have been important when we were looking after our transgender clients.
I honestly don't know what would have been more important to him.
I think you know, he's quite lucky that he had a very holistic service and, you know, he was sort of case loaded and we… But I just think in if we had lots of, you know, if we had lots of clients like him.
What? What would it, as a group, what would be important? I don't. I honestly don't know.

Interviewer 59:56
One thing you mentioned was perhaps the toilets?

Participant 5 1:00:00
Toilets. Yeah, that was a problem. But again, that's sort of establishment, isn't it?
Like what? What can we change with the toilet in [antenatal ward]?

It's… It's how… What could we have done better?
Like what we said, like we had a… Again, we just said, “hey, look, I know this is not… I understand why this will be really awkward” because he sort of mentioned it in a laughing way.
And [antenatal lead midwife] said, “well, why doesn't he just use the toilet next to the…” and that was fine. That's how we dealt with it.
But I don’t know, I don't know how you change it.
If you made it into a… I don't know, a toilet that was genderfluid and everyone could use, obviously there are people who would find that very uncomfortable.

Interviewer 1:00:53
So perhaps you only want the pregnant person?

Participant 5 1:00:57
To use the bathroom? Well, we do that anyway, don't we?
But also I don't know.
I don't know. I don't know… quite know if we're ready for…. I don't know.

Interviewer 1:01:09
That's OK.

Participant 5 1:01:11
Yeah, I don't know what the best answer to… would be to that.

Interviewer 1:01:14
So moving a bit wider, so not the focus on toilets or bathroom signage, thinking more about gender inclusive educational posters.
Do you think it would be feasible to include these?

Participant 5 1:01:32
Yeah, I do.
Umm, I don't know what they say though.
You know, if it was like advertising… I don’t know like an antenatal class or a… you know, but I don't know what they'd say unless we had a celebration of something’ And “we’re celebrating this” or… Again, that individualized care, isn't it?
It's individualized, holistic care.
You can't group….
I don't. I don't know. It's just difficult, isn't it?

Interviewer 1:02:09
Umm. So potentially raising awareness in the posters.

Participant 5 1:02:16
Yeah, yeah.

Interviewer 1:02:18
Yeah. I mean, I guess you spoke about this in the in the notes. In the inpatient and antenatal clinic environment.
Do you see it as being gendered, or do you feel it's quite a neutral environment?

Participant 5 1:02:35
I think it's definitely gendered. Definitely. But I think this is quite new to us and you know we try, but I don't, as I said, I already said to you, the notes… it's just our sort of I don't know… client group at [trust name].

It reflects… It reflects. Antenatal clinic does reflect who comes to our clinics.
And have a client like [client’s name] wasn't…
My client was a more unusual.

Interviewer 1:03:13
Can you think of any examples of things that are quite gendered in that space?

Participant 5 1:03:19
Well again, the toilets. We've got female toilets.
The men have to go somewhere in the corner. But ohh I don't know.
Just, I just… the notes, posters, if you look at all the posters, they've all got like a smiling mum with an adoring looking dad, perfect little baby.
I don't know. I just think it's… That's how it's been for many years.
I did feel it a bit when I was caring for my transgender client.

Interviewer 1:03:48
Umm, so a lack of representation?

Participant 5 1:03:51
It's subtle little things, but you can… when you, when you're aware, you see them more.
They're always like a lovely, you know, a beautiful looking mum with a gorgeous bump and an adoring little baby wearing a pink baby grow and a bow.
It's all. It's still, it is. I think that's that. Yeah, you're right, it is.

Interviewer 1:04:17
Yeah, so a lack of representation for people who are pregnant, who don't identify as women?

Participant 5 1:04:24
Yeah. Ohh yeah absolutely.

Interviewer 1:04:27
OK. And how feasible do you think it would be to create gender inclusive posters or change decorations?

Participant 5 1:04:42
I mean, I think you could probably…
Ohh I I don't know. I really don't know.
I mean that… I think it is glaringly obvious that most, I'm just sitting in the staff room and I'm just looking at one, two, three, four. Four posters. In every single one has got a mum holding a baby.

And no representative… Yeah, I I don't know how. I don't know how you can make it look differently. If you had a transgender male who was sat postnatally holding a baby. I don't think people would know… get the meaning. I think they’d just think he was a dad.

Interviewer 1:05:32
What she would be as well.

Participant 5 1:05:33
I don't think we're quite ready.
And I don't know. I think if you had an antenatal poster of a transgender male with a bump in antenatal clinic, I think people would look at it.
Whether it would normalize it, I don't know.

Interviewer 1:05:52
Yeah. OK. Are there any tools or resources or information you think would be needed to improve the posters and decorations in terms of making them more gender inclusive?

Participant 5 1:06:12
Given that I don't know how I do it, I don't know.
I'd probably like to speak to umm, somebody a… specialist inclusivity midwife and ask from their experience. I'm not… haven't got that much experience.

Interviewer 1:06:31
That's OK.

Participant 5 1:06:32
And also, you know there is this age thing whereas you know, if you ask my 20 year old daughter, she'd be much better at talking about this than I am because her normal was very different than my normal as a young person.

So, I think it depends sometimes on the age of people. Is that bad to say?

Interviewer 1:06:54
No, not at all this.

Participant 5 1:07:02

I think it, I think with sort of and… I don't know how I’d do that.
I definitely phone a friend and speak to somebody who had experience and what they what they had done.
But again, if you if you go to [trust location], their client group is different than ours and somebody, like the contact I met who had a lot of experience in this field because that… that you know his normal. He always has a caseload.

You should speak to him.
It would be interesting. I can pass you on his details.

Interviewer 1:07:31
That would be fantastic. That would be great.
And do you think that it would impact the safety of care provision at all to have more gender inclusive posters and decoration?

Participant 5 1:07:45
Not really.
I think when you when you look after when you do 6000 births a year, maybe 1% is gonna be very offended by this and the rest of the people… You know, maybe 10% are gonna be hugely interested and the rest of the people are going to be like oh that's nice and move on to what interests them.

Interviewer 1:08:05
Yeah. OK.

Participant 5 1:08:06
I don’t know.
I think you know for that 1%, it may, maybe they find it… things offensive that they don't want to come and look at this poster or… it's very difficult.
I just think it's such a breadth people aren't they like, you're never gonna please everybody.

Interviewer 1:08:22
Yeah. OK. Any other thoughts or comments about that last recommendation?

Participant 5 1:08:29
The posters?

Interviewer 1:08:31
Yeah.

Participant 5 1:08:33
Ohh yeah, I mean, that's… I think it's great.
I think you know it depends on what the posters were trying to say.
If it's just to celebrate inclusivity and diversity, brilliant.
Is it to give you information? Like you know, if for example if we… you know, if we could offer a support group and to give some… you know, that kind of information brilliant.
I think the poster for the sake of a poster or just to add, maybe not, but it depends on what the message of the poster was.

Interviewer 1:09:04
Hmm.
So it sounds like you wouldn't want it to decrease to just a tick box, “we've been inclusive”?

Participant 5 1:09:10
Exactly. Exactly.
You don't.
You don't wanna just… Yeah, exactly.

Interviewer 1:09:16
Yeah. OK.

Participant 5 1:09:16
If it was useful and it was to give a message and you know, I would be all for it.

Interviewer 1:09:22
OK, great.

Participant 5 1:09:24
We have a lot of posters as well, don't forget to like you know, a lot of posters just get diluted in there.

Interviewer 1:09:30
That's true.
And so those were the recommendations from the literature. Do any of those surprise you?

Participant 5 1:09:40
No, not at all. I'm surprised that people who talk about their computer system have never run into the same problems, given this is national, but no not at all.

Interviewer 1:09:51
Yeah. And any of them… do you see any of them as being more important than the others? And I can run through them again if you need.

Participant 5 1:10:00
So the pronouns, I think is a great idea.
The only one that I think was slightly controversial is asking what people prefer to call their genitals by… I'm not… I think people will either find that hilarious…
I think it's quite an ask for the midwives and doctors, which we do step up to again, maybe I'm showing my age.
I think people do step up to what we are asked to do and if this became something that women felt strongly about, we would do it.
I don't know. I think we get…. I don't know.
I just can’t imagine it, but I can see certain groups would find that comforting.

And I don't know, that's one… Maybe I'll sit on the fence for a little while and see what happens there.
I'm not sure.

Interviewer 1:10:50
That's fine.

Participant 5 1:10:52
I think education, always so important, normalizes things, makes people think twice.
Umm, I think that's great.
I think having information posters is great, just depending on what message they’re giving.
Umm yeah.

Interviewer 1:11:13
OK. And is there anything that hasn't been included in those recommendations that you would see as important?

Participant 5 1:11:20
Uh, I think having sort of having a, having people like [specialist midwife] more available, I don't know, like sort of having a network maybe you know, like we have a [specialist role] where I'll be in touch with [specialist role] midwives, having somebody like a person in a trust who could be like the point of… because this isn't, this may not have… It may happen in a month, it may never happen again for two years, you don't know, but you know to have all that information as soon as you book somebody who's transgender will be so much easier.

Instead of having to have to work it out and wing it and go by… but it's really, I think any complex pregnancy is important to be holistic. And just look at their needs as they arise. And I think like for… he was quite lucky that he came in to maternal medicine because it allowed us a bit more time, even though [specialist consultant] barely saw him.

Because his [specialist role] problem was… not headline, but it was… in a way lucky to have a team who could take the helicopter view and make things, you know, like almost predict the next thing that was gonna happen and make sure it was very comfortable.

Interviewer 1:12:37
Yeah. So potentially more focus on the multidisciplinary working aspect?

Participant 5 1:12:53
Oh, I mean because it's rare, it's almost has to be like a wider multidisciplinary like South of England multidisciplinary that you know that there are people who do have a lot of experience in this kind of thing. You almost have to tap into their experience, but from [trust name] that there's a lot we can't change.
It's the same, you know, with what I'm trying to look at, high dependency unit the minute and how we look after the more sick women we have upstairs, some things you just can't change.
We can't change the bathroom. We can't change, you know, we don't have two toilets, one for men.
You know we… I think to suit all sort of cultural… cultures, I think to have a sort of gender fluid toilet wouldn't work.
I think you just have to almost… you know what same thing where we have a wheelchair using, you almost have to sort of pre-empt what's coming and make that journey of them coming in to have their baby as smooth as possible without them having to work things out.

Interviewer 1:13:54
Umm so pre planning and communication within the team?

Participant 5 1:14:00
Yeah, but as it as it any complex woman or man or birthing person.

Interviewer 1:14:06
Hmm. OK, great. Thank you.
Any other thoughts or comments or anything that we haven't covered that you'd see as important?

Participant 5 1:14:15
No, not really. Not really, I think it I think we're learning.

I think it's quite new.
I mean, as I said I thought the staff were absolutely fantastic. They were brilliant.
Umm, so the only thing I would say was that was the… Almost gossip level, but again it again, like you said, it's education and people are interested and we are at, you know, we wouldn't be very good midwives if we were… weren't interested and we wanted everybody to be the same. But the same time, you almost have to keep the noise down the little bit.

Interviewer 1:14:53
Yeah. OK. Thank you so much and thank you so much for your time, [Participant 5] .
If, if that's everything, I can end the recording now.

Participant 5 1:15:01
Yeah, yeah, I hope it was only useful.
I think you're doing a great thing, it's a great thing you're doing.

Interviewer 1:15:04
Umm.
Thank you.

Interviewer stopped transcription

# Participant 6 Transcript

1h 13m 44s

Interviewer started transcription

Interviewer 0:06
So thank you so much for agreeing to talk to me. This study is exploring healthcare professionals experience, knowledge and attitudes towards care provision for transgender pregnant people.
Are you happy to participate?

Participant 6 0:19
Yes.

Interviewer 0:20
Amazing. And do you have any questions about study at the moment?
Amazing. And are you happy for me to record audio and visual?

Participant 6 0:28
Yes, that's fine.

Interviewer 0:30
OK. Thank you. So what's your professional role?

Participant 6 0:34
Consultant midwife.

Interviewer 0:36
And in which region of the country do you work?

Participant 6 0:39
[Location].

Interviewer 0:40
[Location], and how many years have you been working as a consultant midwife?

Participant 6 0:44
Less than one.

Interviewer 0:46
Oh, congratulations.
And what's your gender identity?

Participant 6 0:52
Female.

Interviewer 0:53
Female, and sorry, just before you're a consultant midwife. How long were you working as a midwife for?

Participant 6 1:03
18 years, 19 years this year.

Interviewer 1:06
Wow. Amazing.

Participant 6 1:07
Mm hmm.

Interviewer 1:09
Amazing. So just a bit of background information before we go into the recommendations. Have you had any experience working with transgender or gender non-conforming pregnant people?

Participant 6 1:20
I must have done, but I genuinely don't think in a closer caseload and look after predominantly people in the Community and not to my explicit knowledge on my caseloads. I have, yeah, not myself basically I've known of and been aware of within our service, but I have not provided the care.

Interviewer 1:34
Hmm.
Hmm.
No, fair enough. What encounters have you been aware of in your hospital or place of work?

Participant 6 1:53
I've been aware of transgender women coming through the service and having their babies.

Interviewer 2:03
OK. And what sort of support for providing care did the midwives have for those people?

Participant 6 2:13
Sadly, we're still haven't got… we don't have a guideline to my knowledge.

No, I know other places that do that have created guidance and that is something that is available to support their staff.
That's in [location name], by the way, and I'm aware of that.
There's very little training, I'd say, if any, and certainly on the national agenda for training at the moment, there's nothing that talks about this. We haven't even technically got neurodivergence on our training agendas from a national perspective at the moment, which I really hope all these things will change.
So, yes, very, very little.

Interviewer 3:10
The midwives who were providing the care, did you have any, like, discussions with them about how they felt about the care provision or it was very removed from?

Participant 6 3:20
No, I haven't had any discussions with them. I wasn't within my role as I am now. I think if it had been within my role now, I'd be a lot more involved or at least one of us would be just touching in maybe just because of the fact that we're aware we've got so little support in place.
So no, I haven't.

Interviewer 3:40
Yeah. No, that's fine.
And speaking a bit more generally, so within in your role as a consultant midwife, can you describe a bit about your philosophy of care and your care priorities?

Participant 6 3:55
My philosophy of care. I'd like to I'd like to think that it was, is just very holistic individualised and I can't quite think of the right word I want to say now, inclusive I guess is the right word, but you know, aware of everybody as a complete individual.
I would say that I specifically am very trauma informed as a background kind of perspective and framework almost.
So I think the way I approach people who come through our services very much based on the high likelihood that we have to assume everyone's got their own specific things, issues, experiences, whatever it might be that they want to describe it as that's gonna curate how they experience us as a care provider as well.
So I'm very aware of that and I say that's the base, that's the foundation for my care.

Interviewer 5:12
Hmm. Thank you and what tools, resources and information do you have available to you to support the care provision and the care you provide?

Participant 6 5:25
In regards to specifically transgender?

Interviewer 5:26
Just for generally for anyone, caring for any member of the population.
So like what informs your practise? So you're saying that the trauma informed care provision so, So what? What supports you to make your care trauma informed?

Participant 6 5:36

OK, well, there's a... I don't think it's been well utilised within maternity units guidelines and organisational cultures, but there is a… I don't know if it's called a white paper. There is a paper anyway. Basically there's… I'm trying to think who it's been provided by now. It was done by some of Blackpool. It was a few years ago. It was like 2019, 20, a Blackpool… Young something or other?
This is not very helpful, is it?

Interviewer 6:22
It's OK.

Participant 6 6:22
I was involved in it as well and I can't remember. I was lucky enough to be involved in it. I didn't, I wasn't involved in the whole thing in terms of writing it, but I was involved in the reviewing of it before it became out of a publication. So almost like a sense checking and editing.
So there is basically a trauma informed care for maternity services paper that details, I suppose a lot of common experiences from people who come through our service the way they experience us, what they've had going on potentially in terms of the bigger issues in their lives, for example, a big one would be sexual abuse.
And then what kind of things are needed and required to be supportive for that individual, but also then for a larger organisational perspective and there's lots of work on trauma informed organisations, for example, that is not, I would say, implemented as a standardised structure within the NHS though. So the ways that I've tried to do that in our own specific area and unit is to talk about that in mandatory training. To highlight that basically and then to put it through all of the work that I provide with personalised care planning, it runs through that. The language I use and to update all the language so things like the rebirth, the RCM rebirth paper is another useful way of utilising service users’ experience and words and pulling it into what and how we provide services.
Which we're trying, which we're trying to do, for example.
So yes, there are things out there I say on this kind of topic though, that it's on any on anything that comes from evidence based. It's a long slog to get it into a place where it's actually norm, the norm in care provision. Does that answer your question?

Interviewer 8:25
Hmm, I think you know that's really interesting. Thank you. And then I think you touched on it a little bit with the care you provide, but in general, so within your care, how do you define safe care?

Participant 6 8:38
How do I define safe care?
Well, gosh, we could go on so many tangents here.
I'm not sure I can articulate that succinctly at the moment.
Safe care for everyone, so safe care for the people and for the practitioners?
Is that what you're talking about or any? Are you literally going as big as you want?

Interviewer 9:02
Yeah.
Just a big just a primer before we start talking about the recommendations. So wherever it brings your mind really.

Participant 6 9:11
OK, well, when I talk about safety in maternity services, my mind goes off into over like 100 different directions.
Because I think that there's a lot that's conflicting within what we consider to be safety, the safety agenda.
And there's obviously a safety agenda, for example, for a reason.
And at times, I think it also, it supports personalised choice and care planning and also at times it is like the complete opposite of the personalized care planning agenda.
So I think safe care in a nutshell would be care that is physically and emotionally appropriate, or psychologically maybe, appropriate for the person, the individual that you are dealing with. Bearing in mind that we deal with thousands of people in any given year, and we have a safety agenda. That directs us to standardised care that we have to provide as a baseline.

Interviewer 10:29
Hmm. Yeah. No, that makes a lot of sense. Thank you.
OK, fab. So if it's OK with you, I'll go on to some of the recommendations from the literature and then it's just anything that springs to mind really, and I'll ask some prompt questions throughout as well. So, they’re are no particular order, but it's just the things that came up most in the literature review.
One of the recommendations was training as a part of continued professional development on cultural competency, including sexual and gender diversity. What do you think of this?

Participant 6 11:08
I think it makes sense.
I think there's…
I think the general trend towards dictating what training is provided as a minimum standard in our maternity units is very much here.
My general thing is that training is an easy thing to say that we need to do, and I don't think that there's, I don't know what the evidence I've heard conflicting things and I don't know it. I haven't read it. I don't know what the evidence is, so I think training alone doesn't tick the box. And like it ticks a box, but it doesn't... It doesn't achieve everything you want it to achieve. So I think it's a good part of a like a plan.
And I think it is important and sensible that that is put within like our personalised, for example in the Core Competency Framework, version two, that we've got now this year's one, we have EEPC, equality, equity, personalised care so that, and at the moment there's like a whole list of topics and as a minimum you have to rotate around with them on a three yearly basis so your worry is, is that at the moment on a three yearly basis you'll be you'll get some training on certain topics and then you have to wait another three years. Everyone believes that their topic is important enough to be yearly and actually they are.
The problem is that in maternity services, the Core Competency Framework basically suggests or lays out that you should probably have about five study days a year based on what they're saying and a lot of these are MDT, which is good.
But that has a massive, massive impact to our service provision and the cost. So as a simple like suggestion, recommendation from a piece of evidence, yeah, it totally is like your goal, number one that you just write down.
We'll provide training, lovely. Everyone says it is a tick box thing, done.
Like the realities are really hard in terms of who delivers the training, what does the training, what does the training mean? What's going to be backed up behind the training? What do we need to support that to make it a real part of our organisational culture and knowledge base?
And that's what we are massively going to be struggling with over the next couple of years within maternity because of Ockenden and Kirkup and not because of in a bad way, but because of though where we are with like the training as it is now and what we've got to put in there and what that means for trusts and it's a lot of money. It means a lot of money and who's going to deliver it, like that's really important. Like if you haven't got the expertise in house, who are you going to have to deliver it? Will you get the same thing out of an e-learning package?
You know it’s the almost nitty gritty of how do we deliver something meaningful?

Interviewer 14:34
Absolutely. And some of the questions you posed are actually the questions that I would also ask to you. So in an ideal world, what sort of person or what sort of expertise do you think would be needed for the training provider?

Participant 6 14:51
People with lived experience or with an in depth knowledge of working with people and who can speak knowingly with expertise for the knowledge base that they are updating you with, like anything, you know, whatever we provide, I wouldn't want to be talking particularly about preeclampsia at this point. And I absolutely can talk about preeclampsia in a basic term or, you know, in a normal way, no problem. But do you want me to speak about preeclampsia?
You know, no, you definitely don't because you want someone who's got, like, a real passion for it and a real knowledge base that's specific to preeclampsia.
And I think yes, that would, that's the important bit is the knowledge, the expertise and the passion to be doing it even if it's not their own personal experience.

Interviewer 15:51
Mm hmm, no, absolutely. And it's OK if you're not sure, but based on the knowledge you have at the moment, what sort of things do you feel would be important to include in training?

Participant 6 16:09
I think really, it's probably going back to basics because a lot of things you just… you really worry, you're going to get wrong for them.
And I know we put pronouns on our emails, but I think for a lot of people, I don't know that it means anything, you know, because it's not… It doesn't mean something to us in the same way that it's meaningful and it needs to be listened to.
And then I think, and I think genuinely just getting used to saying and not being worried about tripping up, if you say the wrong pronoun is I think it's just a general worry about you know, you almost overthink and over worry about how you speak.
Because you don't want to cause offence, so I think it's, it's just it almost is like really basic things to a degree around making sure everyone feels heard and comfortable and feels able to have conversations as we would completely, but in a in a way that is gonna make sure that the person feels like they are being listened to and their wishes are being listened to.
Yeah, I think that's just probably a personal fear that I would worry about slipping up and not doing it right.

Interviewer 17:45
Hmm, no, that makes complete sense.

Participant 6 17:48
And I think also, you know in terms of other training and things and that would be like your basics and just making sure that everyone felt that they could be human and that you know that there is that element of kind of normalising it all, but also I think really importantly, to talk about people's experiences so far, because I don't think as we all know that we're very good at listening to women in terms of what comes out of the papers around birth.
And so, you know, I think if we don't listen to women, then I bet your bottom dollar we are even worse at listening to people who don't identify as women.
Or people who,? you know, it says that it's just the historical thing of being different on some level, like we're not. We're not. We're not even listening really to the that we… We don't listen full stop to the people that we ought to be, that we're serving and that we ought to be listening to whether…
Whatever gender they identify as.
But I suspect strongly that it would be like that… It would be worse, like it is, you know, for black women, because I think again, we can't hide from that fact that we know that that's there.
The unconscious bias or the way that these women and these babies have worse outcomes, is because of that.

Interviewer 19:22
Yeah. No, that's really interesting. Thank you. So training informed by the lived experience of transgender people who have been pregnant and accessed care services. Yes. OK, thank you and.

Participant 6 19:31
Services.

Interviewer 19:36
This sort of training where we would have informed by people with experience and informed by professionals with expertise, how feasible does that seem within NHS services at the moment?

Participant 6 19:51
Providing the training?

Interviewer 19:53
Yes.

Participant 6 19:56
So again, I think if this was to be something that had to happen then it would come through the MTP [maternity transformation programme] and it would come with money, for the first year, and then what they do is they then put it into like the core competency framework. So that's going to be the only way that you get this as a standard, a national standard, or a standardised topic in everyone's maternity unit, so at least there is a clear way for that to happen now, because that wouldn't have been possible a few years ago.
So I say it is. It's entirely possible, but it has to come with the right provision and support because different areas are going to have different abilities to find their own training, and I think you know, you're kind of left with the maternity service to put on all this training for specific topics yourself and you've got to find people with appropriate knowledge base ultimately for some of these things or you'd like to because then it's more meaningful. And if you don't have access to that within funds, which we don't generally. Then then you're not going to have a meaningful training, so it's pointless.
And that's the problem really. You know it will… The quality would still vary so much depending on what area you were and how much money was attached to it. And you know, again in [location], I'm sure they have amazing training and because they've got very specifically, I know they've got a midwife that works for them that has pushed this agenda, for example and created guidelines and then does training and things so that has really helped shape what they do as a service and what they can provide as training and I think in other areas that would be a lot harder to provide without funding. A bit more hit and miss.

Interviewer 22:06
Hmm.
Fair enough.
And thinking about the multitude of definitions of safe care. How do you feel training impacts the safety of the care provision, or specifically this, If we had training on caring for people who are transgender during pregnancy?

Participant 6 22:25
I think training is an important asset. It's like your kind of cornerstone, isn't it? In a way. That's why I say it's like your first recommendation for everything that comes out.
Because it's also, it's also the easiest thing to say to that we can do and should do, but it is all. It's like your cornerstone. It's like your baseline way of getting information and having conversations, I think as well having meaningful conversations during training is far better than being told things.
So definitely it's an important part of any safety agenda because it helps to standardise and raise things. Raise issues generally that people can have conversations over.

Interviewer 23:12
And any other thoughts about this recommendation, so training on cultural competency and sexuality and gender diversity?

Participant 6 23:22
I mean I have been on, so I have been on a training session like this before as part of the my AHSN [health innovation network] role and I found it really really really useful. Really interesting.
And very yeah, very helpful. So I think being able to do that and get it to all clinicians is really important because it's like a lot of things, things exist, but only if you know about them or you sit outside of that clinical bubble.
So I think having been on something like that, it would be wonderful to be able to provide that to our staff and to make it multidisciplinary like a lot, you know, like that's a really important topic to make sure it's not just midwives and the core competency framework does talk very much about and you know all the reports, talk very much about multidisciplinary training. So that we can share experiences and listen to each others, because midwives will have very different experiences working one-on-one in the labour rooms. For example, for long shifts.
You know, you get to know people. You have very different experiences and care compared to the doctors who come in and out and who the anaesthetists who have, like a head end experience for, you know, an hour maximum, or a back experience for an epidural for half an hour. You know, I think so. I think being able to listen to each other as well and talk in that way together is really important because otherwise the other professions kind of just feel a bit like, well, this is just my one little bit.

Interviewer 25:09
Yeah, no, absolutely. So making training and care provision consistent?

Participant 6 25:18
Well, in terms of in terms of like represent, making training probably more representative of your care provision in like you know you work as part of a MDT [multidisciplinary team] team. And I think historically the midwives had all this training kind of aimed at them, whereas now it's shifting to be MDT.
And that's really important when you've got such important topics.
That impact everybody's way of working.

Interviewer 25:48
Absolutely.
OK, so one of the other recommendations was something you touched on briefly in terms of e-mail signatures, but this one was asking service users their pronouns. What do you think of this as a recommendation?

Participant 6 26:03
Fine. I think we do that. I think we might do that. I'm not sure 100%.
That’s fine, I think it all just, you know, it's the way we have. I think we've gone in terms of like professionals, we've accepted this change and you know very quite quickly I think. But I don't know, I could be very wrong on that front.
And relatively easy that it's become the norm.
And I don't see why we shouldn't be extending that very specifically. And again, I think some places will be doing that already and we might be already doing that.

Interviewer 26:45
And if it is being done where is this taking place? Like what appointment?

Participant 6 26:49
Booking.

Interviewer 26:54
And typically in, if you've had any conversations do people typically find this quite feasible or quite a difficult conversation to have?

Participant 6 27:03
I think it's… I think if I'm right in thinking it is a booking or maybe it's on the self, it could even be like on the self referral which is just like an information sheet that you fill out and put in.
I'm not sure, but I will have a look on our one. But again this is all on [medical record system] right now for us for example. And it's like when you started having to ask about smoking or when you started having to ask about domestic violence when these things have been over years, haven't they? But I think it's like any change or anything that's a little bit kind of you know uncomfortable initially it always is maybe a little bit like… it's just new.
We just have to get on with it. I don't think it is a… It's not a big enough… It's not a big problem. I think we just address… as long as you address the why with the people who are doing it so that they accept it quicker then that's fine. That tends to be the way to go.

Interviewer 27:55
So needing an understanding of why you're asking?

Participant 6 27:58
Simon Sinek “lead with the why”. It's like the greatest piece of advice I have ever come across as a leader.

Interviewer 28:06
Interesting. Thank you. And do you think that asking service users pronouns would impact the safety of the care provision?

Participant 6 28:17
I would hope to think that it would.
You'd like to think that it would be a positive impact.
But like I said, I think I'd need to see more. I'd like to see more in evidence or understanding of what people's experiences are.
Because it would be interesting to know whether their experiences, how they experience some maternity services. And whether they felt safe because I think safety from an individual, from a person going through anything, a person going through a service, it's your personal perception of safety.
Ultimately, because you're the person at the end of it who's gonna have felt safe and not come out psychologically damaged and have trauma so in a way, my perception of safety doesn't matter. It's their perception of safety. So, do they receive safe, appropriate clinical care? Yes, most likely. Was that done in a way that was conducive to having psychological safety? Not sure. That's a personal thing. And I know we clearly get that wrong quite a lot.

Interviewer 29:36
That's a really interesting point. Thank you.
And kind of going on from that, one of the recommendations was asking service users preferred terms for their anatomy and bodily function. So one that people might have heard is preferring the term chest feeding instead of breastfeeding. But this can also extend to other parts of anatomy. So for example, a transgender person might not like to use the term vagina to describe their anatomy and might prefer frontal hole or pelvic opening as just two examples.
What do you think of this recommendation? So asking for someone's preferred terms for anatomy?

Participant 6 30:17
I think if you don't ask, you don't know and I don't know how many people would offer that as a request outwardly. So if it is important to them, then clearly we should know. And if we're not asking them, we're reducing the likelihood that we will do the right thing for them.
I also think and again it's very difficult. It's like the knowing and the doing is still two different things. So we can ask the question and we can say we will endeavour to use these terms for you. If you notice that we do not or haven't, you know please if appropriate and you know I don't know because again, when you're in labour, it's completely different, isn't it? You will notice, but you won't have the energy to say anything, but it's like, please highlight that to us, you know, and that's something that we will do our best to do and I don't know whether you can say that.
And that be enough for someone or that'd be appropriate or not?
I think from a personal perspective, that feels like the most honest thing to say. I will absolutely endeavour to do this the right way for you. There might at times be, you know, it might be at times if I don't get it right, I apologise and please highlight it to me because I might not notice because I think these things are really, you know, these are really new things for and they won't happen very often either.
So I think listening to someone is very important and having that acknowledgement of our humanness and not being able to get it right all the time is still being likely is also really important to have to have that understanding, almost like a contract between you and your individual care provider. So it's on your birth plan. And I just think it's really important to have that honest, open communication and say I absolutely have read your birth plan and I will absolutely will do my utmost to uphold it. If I don't please let me know.
I just think that's honest and open and it's giving the right message, because I fear that otherwise we will ask a question and we won't achieve it because it's so different for us.
And it's not commonplace, so it will take even longer for that to become commonplace because we don't have to deal with it all the time, if that makes sense. So it's almost like a thought process of how yes, absolutely fine, but how do we make that a reality that feels right? Or how do we make it happen?
And I think that's the bit that's harder as always.

Interviewer 33:15
Absolutely. And so in terms of feasibility, it sounds like you're saying asking is feasible, but implementing might be a bit trickier?

Participant 6 33:25
I think that's just human nature. Yeah, I think, we've got a lot of well-trodden snow tracks in our brain of neural pathways.
And especially when it comes to the kind of job that we do in maternity, and it's not me making excuses at all, this is just me kind of like I think explaining like our habits and especially when you become, you know, like proficient in something.
You're on a well-trodden neural pathway of what you do, when and how. And you're quick at it as well because you need to be. And that's your job. And I'm not anymore.
But I think that's the idea. When you're in a clinical practise environment anyway. So I think then in those moments where you've naturally got a higher stress level.
You're again, you are in much more of a heightened… Yeah, you've got more adrenaline going around. You think, you know, I'm just thinking about your labour ward always like your… I was gonna say something really mean then.
But as you're like point of decision making unhappiness and like the most difficult places of having to work psychologically, like in terms of like you know just your normal biological responses to what's going on to stress, and we all know what our stress responses do to us. So actually, no, I think that's a lie. I don't think lots of people know actually. And this is part of the problem around trauma informed care. So I think having that understanding of people and of the people who provide the care is really important. 'cause you're just acknowledging everyone's needs and everyone's limitations.
And the ski track… You're gonna fall off if you start to make a new pathway. You're gonna just, you're gonna tumble a few times before you make that new pathway, and before it's solidified in your brain. And I think that's reality.
So I just think it takes time and patience and encouragement on all fronts to get that kind of thing happening with consistency and reliability.

Interviewer 35:51
And, I'm kind of following on from that. Do you think that any specific tools, resources or information would be needed to ask and then implement specific terminology?

Participant 6 36:12
I'm thinking the implementing, but it's really difficult. I think you need… I think it's like… I don't know what it would be. I would say that it needs to be informed by like a, you know, informed by an understanding of our reactions under stress, informed by change management processes so that it is feasible. I don't know what that looks. I can't tell you what I think that would look like right now.
But I think it's a bit like making every contact count and everything as well, isn't it?
It's having that understanding, I think it's the understanding takes longer in all honesty. So maybe like something more specific for community midwives would be really helpful because they are your people who deal with generally all of your all of your service users the most.
And so starting with having, you know, like a really good understanding for that cohort of your staff would be really important, because if you can get it right there, you're going to really massively at least improve how they feel in pregnancy.
But also then hopefully you know, it will trickle out, but in terms of I find the implementation a bit really difficult. I think that's genuinely the hardest thing on a realistic level, you can completely come up with something that is… that sounds doable and it sounds fine. I wouldn't have a problem with it at all, but like we know people and we know the issues of… like I'm a coach now like are 100% see all the time, how difficult it is for someone to action what they say they're going, they want to do or create new habits. You know these things are psychologically really deep.
And we have to go to that place with them if we're going to try and shift.
And I don't know if the NHS has got the time for that.

Interviewer 38:44
No, that's fine. Thank you. And thinking back to that like perceived safety, do you think that asking about preferred terms for anatomy would impact the perception of safety for the service user?

Participant 6 39:02
I mean one can only hope that it would be yes, but they've got to feel listened to because so we can't just ask and then not do. So only if we do it.

Interviewer 39:17
No, that makes a lot of sense.

Participant 6 39:17
Or we make an attempt, you know, or we make an honest attempt to do that.
And then and but again, I think it comes down to their psychological safety.
Their physical safety, I think they will… They will feel either physically safe or not safe based on the care that we're providing.
And the way that we're providing it.
And like anyone, like what… How much you are already aware of or expecting or not expecting so again it comes down to expectations a lot of the time and having an understanding of what your body is going to do and those things as well. But yes, if we can action it appropriately, then I would hope that would make them feel safer.

Interviewer 40:23
Hmm. And following up from that actioning, one of the other recommendations was about documentation of correct pronouns.
What do you think of this recommendation?

Participant 6 40:37
Along the lines of asking for the pronouns and then documenting it on the notes?

Interviewer 40:43
Yes.

Participant 6 40:44
Right. And then documenting it consistently in the notes, you mean?

Interviewer 40:48
Documenting it consistently in the notes and having it available for other members of staff to refer to.

Participant 6 40:58
So we're all electronic it would it, I think probably the best thing…
Because you're right, that definitely isn't a pronoun like on the top banner like we have all our demographics on the top banner, for example.
You see everything, what would be really helpful is if you have a pronouns there.
Documenting it in the notes consistently right, I think again, it's much like saying it consistently right. That takes time and effort and will happen with time and effort, I think.
As long as it's documented in the appropriate place in the front page or on the front banner, then you should be alerted to it enough that you can see that you need to be using a different pronoun when you are writing anything in the notes or their name, and you know very specifically that. I think, can I be really candid here and just say what's on my mind on that front in terms of times and the thing that came to mind when I was thinking that was that again takes mental energy and effort to remember to write they, rather than she, for example.
And my worry would be that we have to write contemporaneous notes as best we can. And how do we make sure that, it's not really an issue, but do you know what I mean? I'm just thinking you would slow… You would slow down and not be as contemporaneous if you couldn't just write. So I think it's almost like, do we just take out? Do we just try and take out any gender specific.
So you, you know, it's so that your notes can be this is what we did and I think probably most of the time in those situations it's not going to be that kind of documentation that you need to be more careful over or worded. But how, I don't know what is my question, how careful would we do we have to be?
Would we, would it be appropriate to write your notes in the moment as quickly as you possibly could, as contemporaneous as you could and if you didn't quite get if you did put something in just out of absolute like, just have it or have it, but just like norm, you know that kind of thing of like doing it, you're on, you'll do it, you're doing what you have to do clinically and we know what that's like and you, we know that we have to document contemporaneously and if we don't document properly, then we are we're going to have a bigger problems. So my first thought there on like a what barriers could there be rather than this is an issue, what barriers could there be are that that slows people down and makes people worry about what they're writing. And then we don't have appropriate level of documentation. So do we just almost in those situations just go absolutely, just write to your… Make sure you've got contemporaneous documentation, and then we will have a look and we check back over it afterwards just to make sure that if there are any inconsistencies or if we have written a wrong pronoun that we can go back and edit that pronoun and that that's OK.
Because I'm pretty sure on [medical record system], you can do that or we could at least put in a note saying written contemporaneously in an emergency situation, aware we got pronouns wrong and again is how important is that to that person? Is it something like we don't ever want to be…
But do you know what I'm trying to say? In terms of like, you're just thinking about the clinical environment, what you need to do, how and when and making sure we're trying to do the right thing for everyone, but not tying ourselves up in so many knots that we're not providing safe care because of a word.

Interviewer 44:58
Absolutely.

Participant 6 44:59
And I think that sounds really harsh, but that would be your… that would be the nitty gritty of fear around getting it wrong for people. And that way that we don't want to get it wrong, but what's the appropriate level of getting it wrong here, and what's the damage it causes? I think that's the kind of thing that's really… that would be the really important like little really, really minutiae detail of how we care for people and what's important from a service provision safety level and from a service user experience level.

Interviewer 45:35
Mm hmm. Amazing. And I think you've touched really well on the feasibility of it in that. If you think about it and take time, then it can be more feasible than in a rush and an emergency. And then also in that situation, the safety of having contemporaneous documentation being very important.

Participant 6 45:56
Yeah, we've just, we've never been in a position in maternity services as we are right now where we're so under pressure, we've got so much admin to do for anyone given person.
And we are so under the spotlight.
So it's getting it right, but it's getting it right at the appropriate level for safety and on a clinical and a psychological level as best we can.
And yeah, and thinking about like what the reality is of saying things.
Kind of in a “we can just do this way” and I'm guilty of that, “we can absolutely, yeah, absolutely”. We can just do “this sounds very sensible and very reasonable”.
And the reality is, is that sometimes that will have more of an impact than we realise.
But it's on the really small little details, probably that don't happen very often, but we don't want to compromise safety there. So just it’s the guidance, how do we support midwives and professionals to do their job and how do we also support them to make sure that they the service users feel safe.

Interviewer 47:19
And.
Any specific tools, resources or information? Do you think that would be needed to document pronouns either on [medical record system] in general and like a or?

Participant 6 47:32
It just needs to be in on all the normal documentation stuff. Like we are all digital now or we're going all digital. It needs to be a normal, expected part if that's what, if that's what we need to do. And that's what is expected of us as professionals then why shouldn't we do be doing it for our service users? And then it therefore it needs to be just a part of all maternity digital notes and I would say on the initial assessment because at the moment I have a surrogate couple who were trying to register the fact that they're going to be living somewhere else with their baby in London rather than down here where the surrogate is and they couldn't register for, you know, kind of care because the format was just like you say, very female focused. And then were a gay couple having a baby by surrogate. It doesn't really apply. And I'm like, no, it sucks. I'm sorry.
Yes, I think it should be on the initial contact form, literally name, pronouns, title. You know it's all there, isn't it? We ask for a title, but title, pronouns, name, relationship to baby.
'Cause then you're also asking, do you want to be called a mother or not?
Are you the father actually? Because we're two surrogate fathers.
So again, I think relationship to baby is probably an important one because we're not… We haven't really talked, we've talked about pronouns. We haven't really talked about like their names. For what you, what you identify as.
Do you identify as a father?

Interviewer 49:15
Absolutely.

Participant 6 49:17
Or a parent? Do you just go, I'm not even identifying as a father. I'm just identifying as a parent. So maybe you know, that needs to be on there as well. My pronouns are. And the way I identify to my child is.

Interviewer 49:31
Yeah. No. Amazing. That's a really good point.
Any other thoughts about that documentation?

Participant 6 49:39
No, it's a tricky one.

Interviewer 49:41
Yes, yes. And you kind of touched on this next one as well, which is so fantastic in terms of moving to digital systems.
One recommendation was to improve the gender inclusivity of electronic medical record systems. So, for example, the majority of electronic medical record systems don't allow someone sex to differ from their gender.

Participant 6 50:04
Yes.

Interviewer 50:07
And some have constrictions where a male patient cannot have a pregnancy added or a male patient can't be admitted to an inpatient female area, such as the labour ward. I'm not sure how that affects [medical record system] because that is specifically for maternity.

Participant 6 50:13
Again, because I haven't ever done this specifically, I'd have to ask someone what we did or if we had to do anything at all. One would hope now that [medical record system] being one of the largest providers is on this because, you know, we do have probably at least one person every year who's going through the service as transgender so you would hope that we have figured this one out, but I actually don't know.

Interviewer 50:52
And have you encountered any difficulties with electronic medical record systems or know of anyone who has?

Participant 6 51:06
I mean, I know it happens, what you just said, but I don't know. I don't know about the examples we've had in our unit.

Interviewer 51:08
OK. Fine, no worries.

Participant 6 51:15
I didn't ask. I didn't think to ask.

Interviewer 51:18
That's absolutely fine. Don't worry. And then this is kind of more of an IT side of things question. But from the knowledge you have of the systems available, how feasible would it be to increase the gender inclusivity of them, so allowing sex to differ from gender, or allowing a pinned pronoun with the demographics.

Participant 6 51:41
I think the pinned pronoun should be pretty easy and asking what they identify as was like in terms of parent, parental role in terms, in terms of the other it side of things, I've got absolutely no idea you're much better going straight to [medical record system] for that conversation and saying this is what we need, make it happen and then they do that on a national basis.

Interviewer 52:02
Hmm. So it needs to be coordinated.

Participant 6 52:05
Yeah, 100%. That's part of their system that would have nothing to do with us.

Interviewer 52:10
Yeah, absolutely.
And do you think that changing electronic medical records systems to allow sex to differ from gender pin pronouns or allowing male patients to have pregnancies added, would that impact the safety of care provision at all?

Participant 6 52:32
One would hope, yes.
I think it's… I think anything that we can do to make it more individualised and specific is important because that's what we talk about doing.
And we've got more chance of getting it right if we've got more information about the person that is correct and applicable.
So that's a safety, that's part of the safety you know, agenda ultimately.

Interviewer 53:04
Amazing. And I think we've kind of talked about it all together instead, so that's fantastic. But any thoughts specifically on the computer systems that we're using?

Participant 6 53:17
No, everyone's got different kind of experiences and opinions on which one's better. I think the one we've got at the moment is better than what we had before, for example. So, and I think [electronic medical record system] is a fairly large provider.
So, not massively, I haven't got any other experience of other systems per say.
To say anything else is better, or does it better already. I wouldn't know.

Interviewer 53:49
That's fine. And have you ever encountered any problems with gender inclusion on [electronic medical record system]?

Participant 6 54:00
Haven't looked specifically to figure that one out, because I've mostly dealt with surrogacy. So I mean, I mean ultimately in terms of the surrogacy things, yes, because you can't… You've got that issue around. You know, registering for care and not being able to as a man. Yeah. So yes, but not in the context of specifically transgender.
I would have to go and have a look at the notes of the people that we've cared for as a unit to know what we did or how we got round that one.

Interviewer 54:37
Still a very good point in terms of inclusion with surrogacy as well, absolutely.

Participant 6 54:39
Oh, it's massive. Yeah.

Interviewer 54:42
OK, amazing. So if it's, I'll move on to the last recommendation and this one was to have inpatient and outpatient maternity environments that were gender inclusive, so this could include gender inclusive educational posters or decorations in the ward environment to make them less gendered, including signage. What are your thoughts on this?

Participant 6 55:10
This is an interesting one because this made me think immediately to Milli Hill’s stuff, and I'm sure you're very aware of that.
I saw something the other the other week. I just thought, oh, OK.
Umm.
This is really hard, isn't it? I don't know that I have given this one enough thought to be able to say I've got a definitive opinion on it.
I think it's… I think it's… Should I explain why I think it's hard?

Interviewer 55:45
Absolutely. If you feel comfortable.

Participant 6 55:47
And why I don't feel like I know what the right thing is, or the right thing I think here is or isn't, because in my head again the easiest thing to say is absolutely we should be making people, all people and any people feel absolutely valued and part of, you know, the makeup of our society and what is I think for want of a better word, normal, you know to not have norms and not like to go away from, but I think but I suppose the difficulty is we do have norms.
And I think on a societal level, that's probably like a… there's probably a lot of argument around why we have norms and how that's important, for example. But anyway, that's a completely different topic really.
So it feels like a really difficult push pull for me on this one, because then the other part of me goes I do understand the whole thing around not getting to the point where we have… like we've… we're afraid or we don't… We don't, we don't talk about women. And I have that. I do... I understand that and I relate to that as a woman, and where I don't even think we have… And this again is the same thing though, because we don't even have what we need and want as women in society or in our lives.
And then so I'm acknowledging that as true. And then I'm also acknowledging the fact that then that therefore means that people who identify as male but can have babies and do want to have babies and things and have to do that process to become parents, they're going to be feeling that on a whole other level.
So it's like I can hold all of these truths as reality and it gets really muddied as to what the best way forward is then.
But I'm very aware of… I'm very aware of the need to listen and to value and to respect everyone's experience as an individual and for that on both sides, not to diminish the other or the others specific issues or terminology or wording, so I would like to get to a place where it can all be held as OK.
I think. Does that make sense?

Interviewer 58:52
It does. No thank you. It's really interesting. And you kind of alluded to it a little bit, but would it be OK to ask you to explain like the pushback or the discourse that you've seen online about potential changes?

Participant 6 59:11
I think and again I haven't looked into this and it's not been something I've kind of got all activist in my kind of in myself about. So I haven't kind of gone into it in detail reading it, but I've just been aware, in lots of different probably, you know, arenas as well of like… of this idea or whether or not it's that's an idea or just you know a belief maybe that everyone…
That we’re...
Yeah, I think the words that I maybe I've picked out on are that we're diminishing to some degree maybe, which sounds wrong. It's almost like, I don't know, it's almost like I can listen to. I could hold them both and I could be like absolutely, I agree with both. OK. So how do we come to the point where we crossover and we make that. what we like we were saying before. meaningful for everyone and the truth for everyone.
And I think there's a fear. Maybe that's what it is. It's like there's a fear that by saying different... I guess it's different words ultimately because I don't think there's an issue, I mean, I certainly wouldn't have an issue with pictures at all, but I think I'd probably would have more of, my gut instinct or my gut feeling would be more of don't take the word women away from women, 100%, which I don't think this is trying to do or we are trying to do, but that would be my gut fear and instinct was we need to keep women's experiences as women's experiences because they also are. Does that make sense?

Interviewer 1:00:51
Absolutely respecting the gender identity of cis women as well.

Participant 6 1:00:56
Yeah. And the fact that we haven't, we haven't even done a good enough job on that front. So we need to up level both.
Ultimately.

Interviewer 1:01:05
Absolutely.
Yeah. And potentially you spoke about holding those ideas together.
It sounds like you aren't holding... It sounds like you aren't holding them in opposition to each other, but instead...

Participant 6 1:01:18
I'm not, but I know that other people do.
So I know that by putting different wording on, you’re one hand being inclusive and you're on another hand being, you know, like it's going to for a period of time as like with anything, that is going to be divisive.
And it is difficult because the majority of our population are female, identifying as women.
You know we are… I don't know what the tell me how the percentage, but I'm guessing it's probably about 1% of the population that come through?

Interviewer 1:02:03
Yeah, the literature I’ve seen says 1% of the population identifies as transgender. So of those people who are choosing to have babies, it's going to be.

Participant 6 1:02:13
Even less. And I so I think that's that for me is probably more of the waiting if that makes sense behind it. In terms of like signage and postage, I would probably, at this point in my time, rightly or wrongly, I would probably say that we want to try and make everything as inclusive as we can.
But I don't feel like changing the words on a sign. For example, in a ward area I don't know.
Oh, it still feels uncomfortable saying it, but I think I think what we're asking otherwise to do is there are so many things that we need to do or that we need to do better in that affects more than 0.2% of the population.
That sounds so harsh, doesn't it?

Interviewer 1:03:10
No. So talking about appropriate prioritisation.

Participant 6 1:03:15
I think so. If you're going to put it in a much more coherent manner.
It's probably more like, yeah. What are the priorities? What's the need and where does that come on the massive list of priorities that we've got and is that the most appropriate way to spend our money and time and effort or whatever it is.
And does it also does it cause people to have a difficult reaction based on 0.2% of the population who might come through your service.

Interviewer 1:03:58
That's interesting to think about.

Participant 6 1:03:58
It's hard, isn't it?

Interviewer 1:04:01
Absolutely.
And I think you've touched on this with the kind of mixed responses. How would you see changing posters, signage, decorations to be more gender inclusive. How would you see that as impacting the safety of care provision?

Participant 6 1:04:22
I don't know. I guess it's like all things, you know, if people feel included then that's going to be better for them. I think like I think going away from pink and blue, great please, let's lose that. No problem whatsoever. I think there are there are lots of ways that we can do this. But again, also respecting the fact that lots of people want pink and blue, so have pink and blue and green and yellow.
Like have all kinds of options available, but you choose what you want and your baby's hat or your baby's name card, or you know, we make it right for you.
Maybe we don't try and make it right for everyone on a external basis. Does that make sense?

Interviewer 1:05:09
Absolutely.

Participant 6 1:05:10
That might be... Maybe that's a better way of doing and thinking about it because it's not that we don't want to, and again, pictures and images are, you know, important thing to have up and to raise awareness of like this is what changing our culture looks like. It's absolutely fine, having that around, not a problem at all, and having different colours around completely fine. I think my only sticking point is the signage.

Interviewer 1:05:37
No, fair enough. And so I mean like, that sounds like you're going back to like that philosophy of care that we spoke about, like the individualisation of the care making sure it's appropriate for that person.

Participant 6 1:05:46
Yeah, which I think is that I think that's the key philosophy of what we've been trying… What we try to do in maternity services anyway and but ultimately, we don't do enough of, like you say for people who don't fit our stereotype of normal.

Interviewer 1:06:03
Hmm. Any other thoughts on gender inclusive posters and decorations?

Participant 6 1:06:13
No, that was a difficult topic to think about.

Interviewer 1:06:16
No that’s absolutely fine.

Participant 6 1:06:18
I think I went through it all externally with you.

Interviewer 1:06:21
No, absolutely.
I'll just recap what the six recommendations were and then just ask you some final questions about them, if that's OK. So that was training, asking about pronouns, asking about referred terms for anatomy, documentation of the pronouns, gender inclusive EMR systems and gender inclusive posters and decorations.
Of those, did any surprise you or any make you think about things?

Participant 6 1:06:50
The posters and decorations totally.

Interviewer 1:06:52
Yeah.

Participant 6 1:06:53
Yes, because I think everything else felt like I, you know, is very much within the realms of, like, your job and what you're expecting.
And then you know the, you know, like “the walking”, you know, the aesthetics in terms of like, you're walking around a maternity unit in 15 steps, you know, and all these things from a service user perspective are really important. So you know that. But I guess I don't, I often think about it when I come into unit. I just think, oh, there's a trolley. Oh, there's a chair. Oh, this looks really messy and just not inviting.
I kind of have always just kind of gone down those lines and maybe because you get so used to your own environment that you accept what's there and what isn't there rather than questioning what's there or isn't there.
And like, I know there's been changes in terms from central delivery suite and labour ward and having different names, and when we go to the new building, we're going to have names for wards. There's not going to be any obvious like antenatal ward, postnatal ward, we're moving away from that and they're going to be names of things. So you'll go to [ward name] ward and you'll go to [ward name] Ward and you'll go to whatever ward.
So again, there are moves and things that are happening in the new unit. We're all colour themed around whatever colour they've given us for our layout. You know again, that's completely out of our control. It's got nothing to do with maternity. It's, here's the colour scheme and palette. There's one for every floor. Pick your colour and this is what's left. It's that kind of thing. So I think really then it becomes about posters and images and I think they are really impactful and powerful. And I think that's important part of raising awareness on a subconscious level for people.
And then I guess I don't know what other signs we would have anyway around the unit. I don't know what you were thinking about it said signage, but yeah, certainly in terms of gender specific colours, I'm absolutely in favour of having a rainbow and you pick your colour that you like. I mean absolutely. Why do we even have pink and blue anymore anyway? It's ridiculous.

Interviewer 1:08:58
So some examples of signage could be toilets saying women's bathroom, so meaning the pregnant person So a toilet that's for a pregnant person like for example to provide a urine sample but not for their birth partner. May say women's toilet. It could be something like food is for women only, like meaning inpatients.

Participant 6 1:09:25
I would just say we could just get around that though and say service users.

Interviewer 1:09:28
Yeah.

Participant 6 1:09:30
And again, so I don't think those things are controversial. I think it's when you try and de gender in a really obvious way because I think that de gendering then is the potential is there to de gender the women that are coming through the service as women and again that might sound utterly, utterly ridiculous to you.

Interviewer 1:09:49
No, not at all.

Participant 6 1:09:51
But I wouldn't want to make a woman feel like she's not a woman anymore, and I wouldn't want to make someone feel like they're not a man if they want to be a man. So I think we just, you know, the safest thing is just to put toilets. Please do not use if you are a partner or a support person, do you know what I mean? Like I just if that's got to be the way we do it, then that's what we do and take out the gender but don't try and replace it with something that's trying to be like it. I think that's my issue.
Call it chest feeding for people who that's really important for and they need it to be chest feeding, 100%. But don't put chest feeding on a sign.
You know I think it's that individualisation and maybe de gendering in a way that still feels inclusive for everybody.

Interviewer 1:10:47
Absolutely, so respecting everyone’s gender identity.

Participant 6 1:10:49
Yes.

Interviewer 1:10:50
Absolutely.
And from those recommendations, is there any that you saw as being more important than any of the others?
I can run through them again if you need.

Participant 6 1:11:12

I mean, as a general, as a general thing, I think these things all come as a package and need to come as a package.
There's no point in having one. I think if you've done the work and these are evidence based guidance kind of things, then the work is there underpinning the why anyway and there's probably, you know you will have less impact if you do one without the other. So I think my general…
It'd be too easy just to pick one out but not acknowledge that it's part of a whole.

Interviewer 1:11:58
Absolutely. And was there anything that you felt was missing from those recommendations or anything you expected us to talk about that we didn't?

Participant 6 1:12:09
Umm, no, I don't think I had any expectations.
That's probably under prepared.

Interviewer 1:12:15
Not at all. That's absolutely fine. OK. Thank. Thanks so much. [Participant’s name], did you have any other thoughts or comments about anything we've spoken about?

Participant 6 1:12:26
No, this is very thought provoking. But for having not gone through this in the role that I'm doing at the moment and I will have to at some point.
Yeah, it's just very thought provoking and it will make me go back and double check what we do already and what we don't. And it’s certainly been on our radar that, you know, having a guideline which, you know and having these kind of recommendations underpin a guideline would be really helpful, because I wouldn't want to write a guideline or to be honest, we would just probably ask [trust name] for it, which I'm sure [senior midwife] did. But you know what I mean like.

Interviewer 1:13:10
Yeah, absolutely.

Participant 6 1:13:11
You'd wanna make sure the guideline was actually useful and feasible. There is no point in having another guideline that is not feasible or does not get implemented. So I think like everything these things take work and time.
And so having that expectation that's part of the process sadly, something you know, you do have to kind of come to terms with when you work in the NHS.

Interviewer 1:13:36
Absolutely. Thank you so much, [participant’s name]. And if you're happy, I'm going to end the recording now.

Interviewer stopped transcription

# Participant 7 Transcript

56m 57s

Interviewer 0:04
Thank you so much for joining me.

Interviewer started transcription

Interviewer 0:06
This study is to explore healthcare professionals, experience knowledge and attitudes towards care provision for transgender and gender nonconforming people.
Have you had an opportunity to read the information sheet?

Participant 7 0:18
Yeah.

Interviewer 0:20
And do you have any questions?

Participant 7 0:22
No.

Interviewer 0:23
And are you happy for me to record audio and visual?

Participant 7 0:27
Yeah, that's fine.

Interviewer 0:28
Thank you.
So just starting with some demographic questions.
What is your professional role?

Participant 7 0:34
I’m a midwife.

Interviewer 0:35
And which region do you work?

Participant 7 0:38
[Location].

Interviewer 0:41
And how many years have you been a midwife?

Participant 7 0:44
Uh, three years.

Interviewer 0:46
And where do you work within midwifery?
So Community? Hospital?

Participant 7 0:53
Umm, rotational within the hospital setting, so all the wards.

Interviewer 0:58
OK.
And for yourself, what's your gender identity?

Participant 7 1:03
Female.

Interviewer 1:04
Thank you.
So just before we go into the main questions, just some questions about your experience.
Have you ever cared for transgender or gender non-conforming pregnant person?

Participant 7 1:15
Yeah.

Interviewer 1:16
Yes.
Can I ask you a bit more about that?

Participant 7 1:20
Yes, I have been involved like in antenatal care of a couple, of two couples actually, a transgender pregnant person.
And in the last few months, actually.
So I've met them a few times through like our triage and Day Assessment unit.

Interviewer 1:44
Hmm.
And so do you feel like it's increasing in prominence, in the hospital or area where you work?

Participant 7 1:53
Uh.
Yeah, I think potentially, yeah, I thought or it's a coincidence that we have two people, two people at the same time that are both transgender.

Interviewer 2:03
Umm.
And how has it been caring for them?

Participant 7 2:08
Um I'd say…
Well mostly things are the same, but it's a language thing, I think, that trips people up. We're so used to saying women or like vaginal examination and those type of things.
That's more thing like made me think more about what I say before I say it.
That's just being extra careful with that sort of thing.
But I think that the actual care itself is it is that the same as it would be normally.

Interviewer 2:45
OK. Thank you.
And how do you feel it's been perceived by yourself and other members of staff, and having transgender pregnant people in the area?

Participant 7 2:52
Hmm.
Yeah, that that has been interesting.
I've definitely heard a few comments of not the nicest nature, which is not… is not nice.
Umm, but I think like, another person that I was working with said that they were worried they would go in the room and they wouldn't know which one of the people was the was the one that was pregnant.
And, but that's OK like you can you can say to them “Ohh, which one of you is having the baby?” That that was fine, I can't see that they'd have a problem with that, and I think it's because it's not common in our trust.
I think just people are nervous about saying the wrong thing or doing the wrong thing.
That's gonna offend someone.

Interviewer 3:43
Umm, so it may be a majority of the concern is coming from a place of caring and not wanting to offend all?

Participant 7 3:49
Yeah.
Yeah, I think so.
I don't think it's a… I haven't heard anyone like... That they don't agree with their circumstances, it’s that they don't understand it more than they have a sort of prejudice. Against it if that makes sense.

Interviewer 4:05
Umm.
And what do you think would help those healthcare professionals to feel more comfortable providing care?

Participant 7 4:15
I think it's just education of what sort of things… But I mean, that's really individual.
Like, what things that each individual person would find offensive, what they would like to be called, what's normal for them.
Umm, I think it's just not being afraid to ask people those things.
And that's the same if that was a woman that was pregnant, is that they some people like to be called certain things or have language that isn't used.
And I think that's just, I think we'll just get better at that as time goes on, with more exposure as well.

Interviewer 4:52
And you kind of touched it already, but in your experience of caring for transgender pregnant people and what do you feel are the key priorities for you within your work?

Participant 7 5:12
Uh.
I think it's just important to make it as…. just keep everything as sort of normal in terms of the care that that they would receive if they weren't transgender, because that shouldn't make any difference to the care that they're receiving.
Umm, making the same as anybody, making sure they're comfortable and that their needs are being met and expectations from the service.

Interviewer 5:39
Umm, so it sounds like not exoticizing that person?

Participant 7 5:43
Yeah.
And it's tricky because the one of the people we have at the moment is very complicated.
Aside of the gender situation, and I think the two are getting are getting confused, which is what makes it complicated as well.

Interviewer 6:00
Hmm.
So, seeing someone not just as that identity?

Participant 7 6:04
Yeah, it's not this the… I think the the couple see that as the problem is that we're anti the transgender couple whereas the issue is everything else that's going on in the pregnancy, not nothing to do with the gender.

Interviewer 6:05
Right.
OK.
So sounds like potentially a a mistrust from the service users as well?

Participant 7 6:21
Uh.
Yeah.

Interviewer 6:28
What do you think of anything that would like add to that sort of mistrust?

Participant 7 6:36
Uh.
Don’t know.
Can't think of anything off the top of my head.

Interviewer 6:45
That’s OK.
And then going back to, you said at the very beginning, sometimes there are a bit more negative comments.
Was there typically a type of person that was more likely to have more negative views?

Participant 7 7:00
And yeah, I’d say probably older people.

Interviewer 7:06
Umm.
And how does that usually present itself?

Participant 7 7:12
I think… I don't think it's intentional that I think it's just they're not…
The way that they would talk about the transgender pregnant person is not the way that they would speak about them in front of them, I think so they would be like misgendering. Just things like that.

Interviewer 7:36
How does it feel for you in in that sort of situation?

Participant 7 7:41
It’s difficult and I think they don't mean to be offensive.
I think it's just a… like education thing.
What you’ve been brought up around, what's normal for you, what you've had experience of in the past and things.
And so I think probably for quite a lot of the like older staff, they probably haven't come across many transgender pregnant people.

Interviewer 8:04
And can you describe any times that you've seen that the care has been particularly good for the transgender people you cared for?
It can be from yourself or from any other members of staff.

Participant 7 8:19
Uh.
I think like we put in place like really like robust like sort of birth planning and plans for like induction, postnatal stay, so that there's a... so things are kept as normal as possible.
But we're identifying sort of areas where there might be potential for any like offense to be caused or where things are…
It's even little things like the toilets on the ward have all got female signs on them, for example so it’s just making little, little changes, things like that before we expect them.

Interviewer 8:58
And who's been in charge of all?
Sorry, go ahead.

Participant 7 9:02
That's what I just gonna say before we sort of like expect them for admission or something.

Interviewer 9:03
I'm sorry.
Who's been in charge of leading this planning?

Participant 7 9:10
So I think it's been from one of our consultants.
And then, like a special, we've got like a specialist mental health and complications support team as well.

Interviewer 9:22
Ah, OK.
And do you know, and it's OK if you don't it, what's informing the preparations that they're doing?
Is it literature? Policy? The opinions and needs of the individual?

Participant 7 9:39
Yeah, I think it's been quite individual led.
I don't know whether we have like a policy and or anything in place.
I think if we don't, then we will be soon.

Interviewer 9:51
And can you describe any times that the care for these transgender pregnant people has been inadequate?

Participant 7 10:02
Umm.
I’m just thinking of a different a different person.
That, in a sort of an emergency situation, members of staff who are coming into their environment didn't know that they identified as a male.
So they were… They were calling them “she” or using like female words and obviously that's difficult because you're in a heightened situation anyway, but then that's only gonna make things more distressing for the person that's having the emergency care.

Interviewer 10:41
Umm, what do you think could have made a difference in that situation?

Participant 7 10:51
I suppose it could have just taken like one person in the room could have just said quietly to the person that was making the error that that was they were making a mistake and just said that sort of briefly.
But if you're having, if you're having a ward round or things you would say that “that the person likes to be using male pronouns and this is their name” and and yeah.

Interviewer 11:23
Umm.
So interdisciplinary communication being really important?

Participant 7 11:27
Yeah.

Interviewer 11:28
OK. Thank you.
And any other comments about anything you've witnessed or experiences you've had working as a midwife?

Participant 7 11:37
Umm, I think the main sort of problems that we've encountered have been sort of logistical thing, so for example, the patient that we've had recently, we couldn't admit them to a maternity board because the admission system said you couldn't admit male patients to maternity, so even something that should be very simple turns into a big palaver, which is just not necessary for adding additional stress to the patient.

Interviewer 12:10
Umm it's really fantastic, loads of the things you've raised are actually questions that I'm gonna ask later so this is so good. This is great.
So before I go on to those recommendations from the literature, I just wanna ask some questions about your care, if that's OK.
Could you describe in general so caring for any, any person, your philosophy of care?

Participant 7 12:43
So to provide safe, family centered care.
Making sure that women feel that they're, or pregnant people, feel like their voice has been heard and that they're involved in decisions around their pregnancy and their baby.
And yeah, promoting safe environment, but the facilitating choice and advocating for patients when they are making a choice that isn't necessarily the normal planned care.

Interviewer 13:21
Umm. And how would you define this safe care?

Participant 7 13:27
I say within the realms of, like evidence based and hospital policy.
And I think there's… obviously people go outside of guidance all the time.
And I think as long as it's an informed decision that they have made based on evidence or based on their like feeling and intuition towards things, then I would say it's promoting choice until other factors materialize and change that.

Interviewer 14:08
No, it does, and in an ideal world, if you didn't have any of the current constructions that you have on your practice or work environment, do you think your philosophy of care would be any different?

Participant 7 14:21
I don’t know. I feel like I am quite good at saying to patients if they don't agree with what we're recommending then they are well within their rights to choose otherwise.
So I feel like it would stay the same.

Interviewer 14:47
Amazing.
And how about that definition of safe care?
Would it change if there weren't any constraints on work and practice?

Participant 7 15:02
No, I think the same.
Again, I think it would probably stay the same.

Interviewer 15:06

OK. And what tools, resources and information are available to you to help you provide this care?

Participant 7 15:18
Umm so, like up-to-date evidence and research is available online, and literature, and hospital policies, training resources, and like conferences and umm yes, in trust training and updates.

Interviewer 15:43
Thank you. So it's OK with you I'll move on to the recommendations from the literature review, so these are things that have come up frequently in research regarding transgender pregnancy care, and they're in no particular order.
So the first one I wanted to discuss was training as a part of continued professional development on cultural competency and sexuality and gender diversity.
What do you think of this as a recommendation?

Participant 7 16:13
Yeah, I think that should be… It should be very easily achieved, for all sort of healthcare professionals.

Interviewer 16:24
And how feasible do you think it would be to incorporate this in training?

Participant 7 16:30
Yeah, I think that would be quite easy to incorporate in like an update day or the yearly mandatory training, as sort of a short session.

Interviewer 16:46
And what sort of person do you think would be best to lead this training?

Participant 7 16:56
Maybe somebody that is going to work closely with any anybody who does identify not as a pregnant women or anybody who has sort of additional…
I don’t know the right word to use…
Anybody that has like any sort of like, specialist needs.
I don’t know, it's not really a specialist need, but we had we had our training delivered by our consultant midwife.

Interviewer 17:26
So you've had training on caring for trans people?

Participant 7 17:41
So not for trans people specifically, but for like minorities.

Interviewer 17:48
OK. And so you're saying someone who has, like, a specialist knowledge in the area and also holds a professional qualification?

Participant 7 17:54
Yeah.
I think not necessarily. I think if there was a, I don't know a charity or something that provided that sort of training and that was like in depth and like specific to hospitals.
Then I think that that would be just as good.

Interviewer 18:20
OK and so as far as you know, is training on transgender pregnancy and care needs something that's done with in practice?

Participant 7 18:36
I think it's like… it's starting to be introduced.

Interviewer 18:41
Amazing.
And how has that been received?

Participant 7 18:47
Yeah, I think, positively. We can identify that there is a need for it.

Interviewer 18:58
Umm.
And how much do you feel that in increased training would contribute towards the safety of care provision?

Participant 7 19:11
I think that it would…
It's gonna help remove any anxieties around caring for a trans person, which means that you're just gonna focus on your normal care, which would help improve safety that way.

Interviewer 19:33
Umm, what do you feel the content of any training should include?

Participant 7 19:41
Umm, I think it's important just like her from, like perspective from trans people that have had a pregnancy and have gone through maternity care.
And because I think there'll be lots of things that we don't even think about that are like offensive or like not appropriate or could be made better to be more inclusive.

Interviewer 20:17
And any other thoughts about training on transgender pregnancy and care needs?

Participant 7 20:30
No, I don't think so.

Interviewer 20:32
OK, great.
So I'll move on to the next recommendation if that's OK?

Participant 7 20:37
OK.

Interviewer 20:37
And so another recommendation was that ask service users their pronouns.
What do you think of this?

Participant 7 20:46
Umm. I don't know.
I think it's not… I can see it both ways.
I can see that for people that don't use the “normal” pronouns for pregnancy, I could see that that would make it a lot easier than having to just bring it up themselves, but I could imagine that there will be some people that would take offense to being asked what their pronouns are.
So I can see I can see it from sort of both ways.

Interviewer 21:26

So with that in mind. How feasible do you think it would be to ask service users their pronouns?

Participant 7 21:37
I think it would, for the majority of people, I think that it would be easy, very easily done and that people would answer and that would be it, you can move on to the next question, but I think there will be some people that… Yeah, for some reason or another would take offense to being asked that question.

I think it would be a small, like a small number of people, but I just think there would be some.

Interviewer 22:06
No, I completely understand what you mean.
In your experience, how often is it that people are asked their pronouns, or in the case of the trans people, you've cared for, how did you know their pronouns?

Participant 7 22:28
Yeah, I don't…
I have never come across someone or like heard.
I've never asked someone and I've never heard someone ask what their pronouns are.
I think with the particular person that I'm thinking of, it was sort of known from booking that they identified as a male and that they, I'm assuming the midwife asked the pronouns then and then sort of since then there's been sort of an understanding in the unit that of this particular person because they're quite complicated.

Interviewer 23:06
OK, so if it was to be asked, is booking a more appropriate time?

Participant 7 23:15
Yeah, I think so.

Interviewer 23:16
Umm.
And in general, I know you said a few people might be offended by being asked their pronouns.
For people who are transgender, how do you think that would affect them? Being asked their pronouns?

Participant 7 23:36
I think it would be positive for them because it's sort of recognizing at that early stage that not everybody that's pregnant identifies as a woman.

They don't have to say that themselves, then, which might be like a source of anxiety for some people.

Interviewer 23:53
To make that disclosure?

Participant 7 23:55
Yeah.

Interviewer 23:57
And it's OK if you don't know, but could you see any way to balance that between people who would it find a positive experience to be asked, and people who be offended to be asked?

Participant 7 24:13
I think… I just think it's something that will get better over time because I think people like even 10 years ago, did people talk about pronouns at all? Probably not.
So I think if that over sort of time of the next few years, it would become sort the norm to be asked that.

Interviewer 24:36
OK. And I mean in general, do you feel that asking service users their pronouns would contribute towards safety of care provision?

Participant 7 24:53
Umm. Maybe a little bit, but I think it's more just a…
No, I suppose it's making everybody feel comfortable, so that is gonna help with safety in terms of people comfortable and like you trust your midwives and your health care professionals, they are more likely to go to them for any problems.

Interviewer 25:27
Umm, so improve that professional relationship?

Participant 7 25:29
Hmm yeah.

Interviewer 25:32
Yeah. And do you see that as being quite important within healthcare?

Participant 7 25:39
Yeah, definitely.

Interviewer 25:41
And if midwives or other healthcare professionals were to start asking users their pronouns, what tools and resources and information do you feel that they would need to do so?

Participant 7 25:58
I think that they would need like a… sort of a referral pathway in place.
So if somebody had said that they are transgender and that they have other pronouns that is there a sort of pathway to follow as to who needs to be informed of that for making like care provision within the hospital.

Interviewer 26:22
Hmm.
So asking the pronouns wouldn't be the end?

Participant 7 26:26
Yeah. So like what are you gonna say afterwards?
So if somebody turns around and says “no, my pronouns aren't, she/her”, what are you gonna say to them after that?
Is that gonna be the end of the conversation?

Interviewer 26:37
Umm, so it sounds like this pathway would include additional resources for transgender people?

Participant 7 26:44
Yeah.

Interviewer 26:45
Yeah, and.
And what sort of resources do you think they would be?

Participant 7 26:59
Maybe it's just about… I don't know, maybe like a passport or something.
That is the things that would be normal for them, things that they would consider like offensive, like language that they like to use, language they don't like to it, so things like that.

I don't know if that would be helpful or if that would make it more of a sort of thing than it needs to be.

Interviewer 27:33
I guess it's so individual, isn't it? That what it's comfortable for one person may not be for another, but what you're describing sounds quite similar to birth preferences as well.

Participant 7 27:42
Hmm. Yeah.
And I know we've had, like, I've looked after someone that had quite severe autism and they had like, an autism passport of things that like words and like, language that they understood or like that was not triggering for them.
And like a similar sort of concept of things like thinking of the person that we have at the moment like “vaginal examination” is really triggering for them, they don't want the word “vagina” to be used at all. Which could be difficult.

Interviewer 28:13
So having that conversation done?

Participant 7 28:15
Yeah. Like what is you prefer?

Interviewer 28:18
Umm.
And we spoke a bit about the comfort of service users being asked for their pronouns.
How do you think it would feel for yourself as the midwife or for other members of staff to be asking these questions?

Participant 7 28:33
Yeah, I think it would be… I think for most midwives that would be fine that they wouldn't have an issue with that or asking that question.

Interviewer 28:44
OK, that's good to hear.
Any other thoughts about that recommendation?

Participant 7 28:51
No I don't think so. I think that would be quite easily achieved.

Interviewer 28:58
Umm.
And so the next one we're gonna talk about actually links on really nicely to loads of things you said, which was asking service users their preferred terms for their anatomy and bodily function.
So the most.
Potentially the most known example, potentially, would be a trans person preferring the term chest feeding rather than breastfeeding, but other examples, as you said people might not like the term “vaginal” and might prefer things such as “pelvic opening” or “frontal hole” as just two examples. What do you think of asking service users their preferred terms for their anatomy?

Participant 7 29:36
I think that that's fine.
I think that should be like taken into consideration. What we have had recently is that we have asked all of these things and then explained that in an emergency situation that some, that those other terms may be used because they are the normal words that we would use.
And then the sort of heightened state we might like revert to that and forget that we are using the terms that they prefer.

Interviewer 30:09
And how are their preferred terms communicated between staff members?

Participant 7 30:17
Umm.
I don't know actually.
I'm just thinking that… whether it's like a birth preferences type that you would have in the notes, whether it's a similar type of thing.
But I think it has just more been like word of mouth between staff members, like sort of handed over to use this term rather than this term.

Interviewer 30:48
And how do you feel that is in terms of the safety of the care provision?

Participant 7 30:53
I think that's probably not the most ideal circumstance to rely on it being handed over, if you're busy and have got lots of other things to hand over then you may forget something like that. But I don't know whether having in the notes is appropriate either.

Interviewer 31:18
So potentially a space separate from the usual documentation. Would this space be accessible to just the healthcare providers or the service users as well?

Participant 7 31:27
Yeah.
So I think we… I think both could be achieved.
I think it would be like an individual, whether they were happy, if they were happy to have something in the notes, then I think that that would be probably the best option.
But if they're not happy, then it could be like only accessible by staff.

But it just might be missed

Interviewer 32:03
And you said about how in emergency situations we might revert to the most commonly used language, how or do you know how that that individual responded to you letting you know that?

Participant 7 32:17
I don't know.
Actually, I think I think that they were happy with that and understanding, but I'm not 100% sure.

Interviewer 32:27
Ah, no. Fair enough.
And if midwives and other healthcare professionals were to ask service users their preferred terms for anatomy, what tools, resources, or information do you think they would need to do so?

Participant 7 32:51
Again, I think it's just like understanding what you're going to say or whether you have anything available to sort of give to patients after. If they said they, they do want different terminology to be used, whether there's like a clear referral pathway in place or whether that's even necessary.

Interviewer 33:18
So some infrastructure to actually have standard guidelines for this?

Participant 7 33:26
Yeah.

Interviewer 33:27
Yeah. And it sounds like in in your experience, asking preferred terms for anatomy is something that's been done for the trans service users you've encountered?

Participant 7 33:39
Yeah.

Interviewer 33:41
Yeah. And do you know who has initiated this discussion?

Participant 7 33:48
But yeah, I'm not sure whether it's come from community midwives or from the consultant.

Interviewer 33:56
Umm, OK.
And this consultant, do they have a specialist knowledge or what's their what's their consulting role?

Participant 7 34:06
They're very involved in anyone who sort of requires any extra… they call it requires extra nurturing. So anyone that's got like sort of any complex sort of additional needs that they would be under the consultant for.

And so I don't know whether they have any sort of specialist training or insight into transgender people in particular or, not sure.

Interviewer 34:41
It sounds like their role is facilitating those individualized plans?

Participant 7 34:44
Yeah, yeah.

Interviewer 34:45
And yeah, OK.
Any other thoughts about asking service users preferred terms for anatomy?

Participant 7 34:54
Um no, I don't think so.

Interviewer 34:58
So if you're happy, we'll move on to the next one, which is again something we've touched on quite well, which is the documentation of the correct pronouns.
What do you think of documenting someone’s pronouns?

Participant 7 35:15
I think that if they're happy with that to be done, then I think that that's fine and something that could be done quite easily and like clearly to show anyone that sort of picked up or opened their records, that they want their plans are.

Interviewer 35:33
So where would you see this being documented?

Participant 7 35:39
Uh. Maybe the… at the moment what I think we have on our like electronic record, it says like “Miss, Mrs, or Mr” like in brackets after their name.
So maybe like after that or right like in the same sort of section.

Interviewer 35:57
So in the demographic, like the personal identifiers?

Participant 7 36:04
Yeah.

Interviewer 36:06
OK. And how feasible do you think that would be on the electronic systems?

Participant 7 36:13
I think that could be something that was done really easily.

Interviewer 36:19
And do you think any additional tools, resources or information would be needed to document pronouns?

Participant 7 36:28
Uh, no, I don't think so.

Interviewer 36:32
And for the people you've seen and cared for, are their pronouns documented?

Participant 7 36:44
I don't think so.

Interviewer 36:46
So it's word of mouth?

Participant 7 36:49
Yeah, I think so.

Interviewer 36:51
OK. And do you think if pronouns were documented, would that make any difference or contribute towards the safety of the care provision?

Participant 7 37:03
Umm. Yeah, I think the same, similar to before, like if it's a, like, encouraging that trusting relationship with the professionals. If you're meeting a new person and they already know that that's your pronouns and you don't have to like add it on, then I think that would… that gonna help promote that like trusting relationship.

Interviewer 37:35
So it sounds like psychological safety, but that psychological safety could also improve physical safety?

Participant 7 37:41
Yeah.

Interviewer 37:42
Yeah. OK. Any other thoughts about the documentation of pronouns?

Participant 7 37:54
I'm just thinking like handheld notes as well as umm, an online record.
So the patient keeps the handheld notes.
So I think if they… like, we have alerts stickers on the front page.
So if they if it became that we asked everybody that pronouns that we could… there could be something that was on the note, that identified that if and as long as the patients were happy with that.

Interviewer 38:24
Umm, OK, thank you.
So one of the other recommendations was again, something that you've touched on in your experience, the electronic medical record systems, so this recommendation would be to make them more gender inclusive.
So this would be examples such as allowing a male patient to have a pregnancy added, allowing a male patient to be admitted to the labour ward and allowing sex to differ from gender potentially.

What do you think about this in general?
What have you seen?

Participant 7 38:56
Yeah, I think that anything electronic seems to be the big issue with the like, not letting us admit male patients and lots of are questions that come up say “does the mother have this?”, “Does the women have this?” Like things that it's not necessary, like it could say patient, person, whatever. The same as I've noticed lots of documentation like post-delivery says like Mother, father, brother. You could have like parent one, parent two or something else that would be more inclusive not only to like trans people but to like same sex couples as well.

Interviewer 39:39
And have there been any issues in practice you've seen with with the electronic medical record systems and how have they overcome it?

Participant 7 39:52
Umm, I don't….
So I've seen that they couldn't admit a male patient and they have found a way around it, but I don't know what it was unfortunately.

Interviewer 40:06
OK. That's OK.
And so how feasible do you think it would be to improve the gender inclusivity of these electronic medical record systems?

Participant 7 40:17
I think that it must be something that's quite easily done, that it could just be a tweak, but it should be easy to admit a male patient to the maternity like it doesn't…
I don't understand why that was such a such an issue.

Interviewer 40:37
And so for the people that you've seen, has your system allowed a pregnancy to be added to these male patients?

Participant 7 40:56
Yes.
So I don't know how or whether that is a problem, because that's obviously done in community.
So I don't ever see that sort of side of things.
That. Yeah.
No, I'd imagine that it creates some sort of issue with that as well.

Interviewer 41:15
And how do you feel that the current lack of gender inclusivity in these electronic medical record systems affects the safety of care provision?

Participant 7 41:28
Uh. Well, if I think about the issues that we had with admitting the patient, that took however many members of staff away from giving actual clinical care to try and sort out how to admit someone.
So that's definitely sort of, if they could have been doing anything else, they could have been providing care then that would definitely have been improving safety rather than for them to be messing around with the computer.

Interviewer 42:01
Umm, so that wastes clinical time?

Participant 7 42:03
Yeah.

Interviewer 42:05
Umm, any other potential complications or concerns in terms of safety with these systems?

Participant 7 42:19
I don't know, not that I can think of.

Interviewer 42:21
It's OK. And if ,and this again is potentially more of question for IT teams, but worth mentioning because you said some of your record systems use terms like “mother” and “women” and assume that there's a father.

What tools, resources and information do you think would be needed to make these electronic medical record systems more inclusive?

Participant 7 42:49
Maybe it would be worth doing a sort of like a survey or something to ask people what they would prefer, like which language they would prefer to be used.
Or, yeah, just to change things because the electronic record side of things the patients don't see.
So it's just that… it’s just for us completing it, but I think it would be quite simple for them to change everything that says “women” or “mother” to say “person”, it could be quite easy.

Interviewer 43:28
If the patients aren't interacting with these record systems, do you still, would you see it as important to make those changes?

Participant 7 43:42
Yeah, because I think it's helping to…
Sort of like ingrain it in your head, that not everybody has the same like pronouns and identity and it's making it sort of more generic so that you can ask people what their sort of individual preferences are.

Interviewer 44:05
So it stops an assumption?

Participant 7 44:08
Yeah.

Interviewer 44:09
OK. Thank you. And any other thoughts about gender inclusive electronic medical record systems?

Participant 7 44:20
No, I don't think so.

Interviewer 44:21
OK. I'll move on to the last recommendation, which was to have gender inclusive posters and decorations in ward, in inpatient and hospital environments.
And obviously this is quite generic, so it could be interpreted in a lot of ways.
But when you hear this, what does it bring to mind for you?

Participant 7 44:46
Um.
I think it's just really depends sort of what, like what would be involved in that.
Again, I think that if…
For example, if like everything was, all the posters and things that showed breastfeeding, if they were changed to chest feeding, I could imagine that there would be people that would have a problem with that.
Umm.
So maybe if it's wasn't something that's so…
Or something.
What?
If it wasn't something like that that people are so passionate about.
If it was, I don't know. I'm trying to think of another example of what could be on the poster.

Interviewer 45:41
So it sounds like it's important that women aren't erased in this process?

Participant 7 45:44
Yeah.
Yeah, I mean lots of people have the sort of thinking that pregnancy is what women do and breastfeeding is the sort of like anatomical word taught for what is going on.
So you know, I think that people that I work with and like service users, I think I can think of like both colleagues and service users that would have an issue with that.

Interviewer 46:19
And how do these the, obviously this is a hypothetical example, because it's supposed to, as far as I don't don't exist in your trust?

Participant 7 46:24
Umm. No.

Interviewer 46:29
So these the colleagues and services to have these strong views, how did these present themselves?

Participant 7 46:38
Umm.
I think that people may think that we are changing… like changing the majority for the few if that makes sense.
So you be erasing anything to do with breastfeeding and everything being using the terms chest feeding would be just to suit a smaller minority of people, rather than the majority, that would still call it breastfeeding.

Participant 7 47:19
I thought, well, I'm not really making any sense.

Interviewer 47:21
No, you are completely.
So it sounds like if there were to be gender inclusive posters, it would definitely have to be in addition to women centered posters?

Participant 7 47:32
Yeah, I think so.
Rather than like replacing, but yeah, I think that would be better because that's offering sort of both perspectives for things.

Interviewer 47:42
Umm.
And what do you see any content of information or posters being if they were to be gender inclusive?

Participant 7 47:54
I'm not sure. I think that would be… It would be better to ask like a focus group or a group of transgender people that have been through pregnancy and maternity care to find what would have been informative for them and what they would have like associated with.

Interviewer 48:25
Umm OK, so make sure that it is focused on those individuals and the target populations?

Participant 7 48:32
Yeah.

Interviewer 48:33
It sounds like you're also leading to that they would contain information that would potentially link to other resources as well?

Participant 7 48:42
Yeah, maybe like this, if there was one for feeding, for example, there were like support groups or websites that they could have sort of information regarding those.

Interviewer 48:53
So going back to that point of not just asking questions and having these things, but having infrastructure to actually support it?

Participant 7 48:58
Yeah.

Interviewer 49:02
Yeah. OK. How feasible do you think it would be to in create gender inclusive posters?

Participant 7 49:13
I think if it was like a national thing, so they were available to trusts like all over the country, I think that would be quite achievable, because they're have the sort of cohort of people to ask and to gain information from.
I think locally that it would probably not be achievable just because I don't think there's a large enough sort of population behind it.
If that makes sense?

Interviewer 49:52
No, that does that.
And then focusing a bit more on that decoration, you said that part of the prebirth planning for the people that are transgender within your organization has been changing the ward environments slightly.
Can you tell me a bit more about what has been done?

Participant 7 50:11
So we had plan in place for a side room for admission so that they can have their partner stay with them.
Umm, in terms of like advocating for their needs and having that support network and yeah, we had just female toilets and the visitor's toilet so there's like the signs,
I think we've actually happened before this patient came through, but the signs were changed to female slash male. I’m trying to think if there was anything else.
This hasn't happened, but we were talking about it at work, but if there were, like postnatal complications for a transgender person, that they would prefer them to like mother and baby unit, and that in itself may be a problem because it's called a mother and baby unit. It’s not the right language.

Interviewer 51:14
No, that's really interesting.
Incidentally, that is something that came up, in terms of the names of services, came up as one of the… sorted into common, majority, and then few, and lots of studies that talk about the names of care provision and place. So like Women's Health centres, a bit more American than UK.

Participant 7 51:40
Hmm.
Like women's, women's and children's hospitals, which everyone seems to be doing.

Interviewer 51:45
Yeah, yeah, absolutely.

Participant 7 51:46
Yeah.

Interviewer 51:48
And so, no, that's definitely something that should be considered. Has the changing toilet signs, has that made or caused any complications?

Participant 7 51:58
I don’t think so.

Interviewer 52:00
Yeah, people are understanding that it's still for pregnant people and not for birth partners?

Participant 7 52:04
Yeah, I think so.
I think it's been fine.

Interviewer 52:18
OK, fine. That's good to hear.
And any other, so ensuring side rooms, ensuring that you can have your partner with you at all time, anything else that's being done that you know of?

Participant 7 52:34
Umm. No, I I don't… I can't think of anything here.

Interviewer 52:48
And anything else that is that you don't know if that's being done, but you think could be helpful to make the environment more gender inclusive?

Participant 7 52:57
Umm. There’s probably loads of things that I just can't think of the top of my head.

Interviewer 53:10
That's OK, don't worry.

Um so, they all grouped together within this recommendation, but if we talk about them separately.
So we said with gender inclusive posters that they would want to have actual services behind them and potentially come from like a national scheme rather than a local area.
And if this was to happen, do you think it would make any difference to the safety of care provision?

Participant 7 53:41
Umm.
Yes, I think if you're seeing something on the post that you identify with then it's making those services more accessible to you, you're not looking at and thinking “ohh like it's not, that's not for me.”
This will make like more services accessible to everyone.

Interviewer 54:06
And increase the visibility of those services?

Participant 7 54:09
Yeah.

Interviewer 54:11
And then in terms of the decoration or ward environment, do you feel this is affecting the safety of care provision within your trust?

Participant 7 54:24
What do you mean?
Like the ward layout?

Interviewer 54:37
Yeah.
Or the changes there’s been and the planning that has been put in place.

Participant 7 54:39
Ohh. So I think it's the same that you're more and more likely to remain in hospital for the like recommended time, recommended treatment, like you have your support network and not feeling like isolated within their environment.
Umm yeah, just that accessibility.

Interviewer 55:07
Umm OK, thank you.
And sounds like, again, psychological safety leading to physical?

Participant 7 55:13
Yeah.

Interviewer 55:14
Yeah. OK. Thank you.
Any other thoughts on from the gender increase of posters and decorations?

Participant 7 55:24
No, I don’t think so.

Interviewer 55:26
So those were the six recommendations. Do any of those surprise you? And I can go through them again if you want.

Participant 7 55:36
I don't think so.
I think they all sound feasible.

Interviewer 55:41
And it sounds like things that you've actually come across quite frequently in practice and which is really, not necessarily great cause some of them aren't on great things, but good to see that the literature is reflecting your experiences. Is there anything that you feel is missing from these recommendations or anything you see as being important that hasn't been brought up here?

Participant 7 55:45
Uh, no, I think it is it…
I think the main sort of problem in barrier is language and wording, and most of the recommendations are helping to improve accessibility of the service.

Interviewer 56:27
And I think you've kind of answered this already, but if you could see any of these principles as being the most important, would you say that would be language or?

Participant 7 56:40
Yeah, I think so.

Interviewer 56:43
Thank you and any other thoughts or comments about any of those?

Participant 7 56:49
Uh, no, I don't think so.

Interviewer 56:53
OK, lovely.
If you're happy, I will stop the recording.

Participant 7 56:57
OK.

Interviewer stopped transcription

# Participant 8 Transcript

54m 55s

Interviewer 0:03
That's on now. So thank you so much for joining me. This study is exploring healthcare professionals experience attitudes and knowledge towards care provision for transgender and gender non conforming pregnant people. Are you happy to go ahead with the information you've had so far?

Interviewer started transcription

Participant 8 0:18
Yep, happy, yeah.

Interviewer 0:20
Amazing. Do you have any questions about the study?

Participant 8 0:25
I guess when do you think you'll publish? Just out of interest, so I can look out for it?

Interviewer 0:29
No, it's absolutely fine. So the dissertation is due in August and then I'll be hoping to publish after that. Hopefully.

Participant 8 0:42
But probably you want longer right now, but that's actually not that long, is it?

Interviewer 0:45
No, it's not.
Any other questions about it?

Participant 8 0:51
No, no, all good.

Interviewer 0:53
And are you happy for me to record the audio and visual?

Participant 8 0:56
Yeah, I am. Yeah.

Interviewer 0:57
Amazing. So firstly, just some demographic questions. What's your professional role?

Participant 8 1:04
So I'm a band 6 midwife.
In like a low risk birth setting and also like a triage area.

Interviewer 1:12
Amazing. And how long have you been doing that for?

Participant 8 1:21
Six years in October.

Interviewer 1:24
Amazing.
And in what region do you work?

Participant 8 1:30
I work in [location].

Interviewer 1:31
That's it. Thank you. And what how would you describe your gender identity?

Participant 8 1:37
I would describe myself as a female.

Interviewer 1:42
Yeah. Thank you. Yeah, thank you. And so before we get into the recommendations from the literature review, have you had any experience working with transgender pregnant people?

Participant 8 1:50

I haven’t, no.

Interviewer 1:55
Yeah, that's absolutely fine. And do you know if there's anyone who has been transgender or gender non conforming in your trust?

Participant 8 2:07
Not to my knowledge, no.

Interviewer 2:10
Fantastic. And any sort of discussion about transgender pregnancy in ward environments or between colleagues?

Participant 8 2:18
Yeah, it's only I think, to my recollection, been one person that I know to be transgender on antenatal ward and I know it really blew people's minds about how they would go about caring for them and it was quite a challenge for everybody to get their head round.

Interviewer 2:41
Hmm. Do you remember any aspects that made it challenging for the care providers?

Participant 8 2:49
I think.
Probably it was just not wanting to get it wrong for them.
Not wanting to use the wrong pronouns, not necessarily understanding their relationship, and also they were quite young. So I think they were an 18 year old.
I think that posed another set of challenges of like, are they ready to make these decisions, both in terms of their gender and their… Like having a baby, basically, it's like quite a lot, isn't it? So I think it was just it kind of created more, more conversation and sort of like intrigue than possibly they would have wanted, I would say.

Interviewer 3:39
And that was between colleagues?

Participant 8 3:40
Between colleagues. Yeah, because I wasn't involved in that person's care.

Interviewer 3:42
OK.
Yeah, no worries.
And from the knowledge that you have or from the experiences that you've discussed with colleagues, what would you see as the priorities for care provision for transgender people at this time?

Participant 8 4:01
I think the priority is probably just to feel like everyone else in the sense that you get individualised care.
And that you're like your experience is a positive one and that you're respected in your views. I think that's probably like… That's actually across the board, but they just want it to be individualised.

Interviewer 4:25
Yeah, absolutely. And.
Do you feel that in the trust that you know if do you have any support in caring for transgender clients?

Participant 8 4:38
Not that I know of. I'm not sure if there is a policy. If there is, God knows I wouldn't be referring to it, but I just feel like there isn't anyone necessarily that I would think “Oh, perfect. You'll be my go to person to get guidance on this”, because I would expect them to know necessarily more than the average person. I don't think it's like an expert at our trust, that's for sure.

Interviewer 5:06
OK. Thank you. OK. Any other thoughts or comments about experiences you've had or things you've seen or heard up to this point?

Participant 8 5:21
I just think that when it comes to caring for transgender people there is that fear of the unknown, and it's another level, I think, within maternity services, we're always worried about getting it wrong, we’re always worried about making a complaint.
There is that other like level of unknown and uncertainty of “I'm going to get this wrong. I'm going to offend somebody” and I think that actually that almost
like demonises that group of people of like, they're gonna be like difficult clients.
They're like, yeah, I feel like there's a perception of “Oh, that would just be another level of difficulty.”
The only thing I can think of recently that was like causing a stir was the Dad Pad.
That we had in NICU.
And there was like comments from a number of staff that actually that wasn't particularly inclusive as a title.
And I would argue that it's OK not to be inclusive with certain things that actually that perhaps was aimed at dads, or it could have been easily changed to parent pad. And then perhaps that would have just been an easy switch. But the sort of how much that conversation kicked off was disproportionate, like it wasn't. It didn't make sense. If that makes sense. So it's like, yeah, it creates it's way more of a stir than it should of it should have just been, like, a simple conversation of is it a simple rename? And it became like, way bigger than it needed to be.
And it probably caused.. the person that brought up the conversation got more stick than they deserved.

Interviewer 7:15
Hmm, interesting. So you've brought up two things here like.
Inclusion and also like almost… Oh God, I forgot the word. Practising in a way to avoid litigation. Almost. Yeah. And with that, with that discussion around the dad pad. What was that pardon?

Participant 8 7:36
Defensive practise.

Interviewer 7:37
That's it, defensive practice.

Participant 8 7:38
That's the one.

Interviewer 7:41
With that discussion on, the dad pad, who were they worried weren't being included by?

Participant 8 7:49
I think it was from a perspective of a same sex couple.
Who had felt that the NHS site service was that clunky in terms of how often we referred, rather than saying like parent or like other, we would end up being like, oh, who is the father of the child, you know, like there's too many kind of slip ups where it caused offence and they found that the Dad Pad was another level of that.
Sort of not feeling like it was aimed at them.
Does that answer questions? Sorry.

Interviewer 8:24
Interesting. No, it does. No, it's really interesting. Thank you. You think you got some really interesting…

Participant 8 8:26
OK.

Interviewer 8:31
Interesting experience.
If it’s OK, I'll ask you some questions about the care you provide, and then we'll go on to the recommendations. So could you describe your philosophy of care at all?

Participant 8 8:47
I think I went into midwifery to… This is hard, to support women.
And that was like my perspective. I felt like as a woman that I think it's really important at a time of, like, real vulnerability that you are like, met by someone who inherently trusts you and you can trust them and that you feel respected and that you feel listened to and heard and seen, and that you can facilitate their choices even if you don't agree with them. And I think that comes up loads in midwifery like someone can have really different views or experience of life that I can't really comprehend or understand, but I just have to respect it.
And understand that it's not my shoes, but I'm gonna support you because it's your experience. But I feel like my philosophy of care is like that, I just wanna help support whoever I'm caring for in a way that feels right for them.

Interviewer 9:55
Amazing. That's lovely. Thank you and within your practise and within your organisation, how would you define safe care?

Participant 8 10:12
That's… it’s actually so tricky like how to define safe care, because I think probably our trust would define safe care as that we have met policies and procedures and that that had been documented.
And I… that differs from my definition of safe care because I think the evidence base that we are writing our policies and procedures on is largely flawed and sometimes non-existent.
So just by following that doesn't mean it necessarily will be safe care.
You can have safe care in terms of you've done enough checks and you've made sure that someone's observations are normal and that their baby's heart rate is staying within a normal range, and that they're, you know, the monitoring is all looking normal and that their labour is progressing within a reasonable time frame, not necessarily the one that we use, but like you can do all of those things, but you may have not met them psychologically. So I think there's another equally important layer of it being like psychologically safe care.
Where someone feels that the environment that they're in is safe and that they feel heard, listened to, seen, respected. Somewhere that they their body is allowed to labour in terms of like their brain will allow them to labour.
And I think that's just as important. We can like tick all the boxes and like appear to give gold standard care, but if they don't feel safe in our presence, they won't necessarily labour.
And therefore they can come into like unnecessary complications because of the environment that we've created.
So I think there's two parts, there's sort of safe care on paper and psychologically safe care.

Interviewer 12:04
Thank you. That's a really, really nice comprehensive answer. Thank you so much, [participant’s name]. And what tools, resources and information do you have within your trust or community to help you provide safe care? Thinking about that very expansive definition.

Participant 8 12:24
I well in terms of what the trust want to see, we've got [electronic medical record system], which is quite prescriptive. You just have to tick boxes to say that you have done the safe care.
You know, write in the free notes, perhaps that you've tried to meet their like… their care in terms of their psychologically, their psychological care would probably be what you'd write in the free notes. So that would kind of show that you've given it in terms of what the trust is looking at, but I think.
Can you repeat the question there again, sorry.

Interviewer 13:01
What tools, resources and information do you have available to support safe care provision?

Participant 8 13:07
OK. Yeah. So I think how we document it is sometimes a prompt.
Umm. However, I would argue that we have very limited resources beyond that, like we've got our shift lead, or our board holder, or our PMA [professional midwifery advocate], or consultant midwife.
Which I guess we could access their support.
But I don't think there's that much room for any conversation beyond the sort of immediate needs.
You know, have you done, a VE [vaginal examination]? How long was it since you emptied her bladder?
Yeah. Have you… have you spoken to the Reg [registrar doctor]? Like it's all the sort of like the very immediate have you followed policy? Have you covered yourself? Is it documented? Have you met these certain time frames? But if I was to have a conversation with a shift lead about, oh yeah, this person doesn't feel safe in this environment and they want to do things in an alternative way to what you've normally seen. I think there would be animosity towards that conversation because they're just ain’t the time.
So perhaps I could consultant midwife or a PMA might be more appropriate, but that would be on a shift to shift basis.

Interviewer 14:41
Thank you. And again, going back to that almost defensive practise with all that documentation.

Participant 8 14:46
Yeah. I honestly think that's like… It's become the lion's share of what people think the work is.
They think, oh, it's OK. I wrote it down and I'm like, but did they feel heard? Did you listen to them? Because just because you wrote it doesn't mean you did it.
Or that someone felt it? Yeah.

Interviewer 15:09
Thank you. So keeping those sort of premises in mind of like safe care and your care philosophy, if it's OK, I'm going to talk through some of the recommendations that came up in the literature review. So these are in no particular order of importance, but we'll just talk through each one and see any thoughts, comments, suggestions about them. Is that OK?

Participant 8 15:29
Cool. Yeah. Cool.

Interviewer 15:31
So one of the recommendations was training as a part of continued professional development on cultural competency and sexuality and gender diversity, what do you think of this as a recommendation?

Participant 8 15:44
Yeah, I think that it's essential and that it's really overlooked. I think we're quite prehistoric in maternity services, like when I talk to my friends that work in mental health, they've been using like., you know, just even like referencing their pronouns in their e-mail as standard. Whereas for us for like, well, why would we do that? You know, our service users are women. And I think that we're probably like a little bit behind the times in terms of being inclusive in our, in our sort of like practise and approach.
You're gonna have to remind me of the question honestly. I've had like 4 hours sleep. So you just have to keep repeating the question.

Interviewer 16:29
No. Well, thank you so much for being here, regardless, I really appreciate.

Participant 8 16:31
It's fine. It wouldn't be a good day. Don't worry.

Interviewer 16:35
So it was… It was about training so that no, that absolutely. Within that training. What do you think you would like to see? What do you think you'd like to be taught in regards to caring for transgender and gender non conforming people?

Participant 8 16:48
I think I probably just want a lot of case studies.
In terms of service users experience what they found helpful, what they found unhelpful, what perhaps put them off from like accessing care, what were the barriers to that?
And then like, I quite like situational like case studies where you can be like, OK, So what would you do? What would you say? And then to talk it through in a really like open way of like actually, you think you're doing the right thing, but perhaps someone could perceive it as that and just have like an open forum of like.
Yeah, people’s experience. How we can optimise it and the sort of pitfalls of where you can cause unintentional offence because I think that's a huge part of like what makes people nervous, is not wanting to offend.

Interviewer 17:47
Hmm. So it sounds like case studies. So it goes back to that person centred approach and also confidence building.

Participant 8 17:57
Well, yeah. I think for people who are open minded but perhaps inexperienced, which I think I'd consider myself, it's confidence. It's like I don't want to… I don't want to say the wrong thing. The last thing I'll ever want to do is cause offence.
And I think sometimes that can mean that you forget the basics because you're so caught up in your own head being like, yeah, use the right pronoun, like alter the what you would refer to as certain like kind of physiological processes, change the way that you would reference it.
Through fear of causing offence and then perhaps realise like, yeah, forgot to do that blood pressure.
I think sometimes you just forget the basics, so I think it would be good to have that confidence so that you could just be like, you know what, I'm confident in providing individualised care to all groups of people.

Interviewer 18:55
Thank you. And what sort of person do you feel would be helpful to deliver this training?

Participant 8 19:07
Either someone with firsthand experience of caring for a trans person or like a gender non conforming person, like or someone who was a service user that had that experience and probably both.
I think having both like kind of viewpoints, the kind of a bit more of a conversation would be helpful.

Interviewer 19:35
Thank you. And is this sort of training anything that you're receiving at your trust?

Participant 8 19:37

No!

Interviewer 19:38
No, that's fine.
No, that's OK. And then last question on training.
Do you feel training impacts the safety of care provision in regards to care for transgender people?

Participant 8 19:56
Yeah, absolutely. Because I think it's like, first impressions count and if we, you know, like, if we mess it up at the beginning and perhaps like.
Yeah, just make a faux pas on the offset, they might just think you know what, this service, they can't meet my needs. They don't understand me. I felt embarrassed, ashamed, misunderstood, offended. And therefore I will not access it again and like it could have been that we just mess up their booking.
And then we don't see them again. So I think, yeah, I think it's huge.

Interviewer 20:37
Yeah. So highlighting that importance of relationship building for, for safety as well in terms of the facilitating access.

Participant 8 20:44
Yeah.

Interviewer 21:00
No, you're fine. It's good. OK.
Yes. Any other thoughts on training at all?

Participant 8 21:11
Umm.
I kind of think when I said, you know, like someone who's provided care and someone who's a service user, thinking about that, I think that people have some gross misconceptions that would probably need unpicking before they were put in a situation where they could offend. So perhaps like a morning session of like just exploring views and what people's understandings are so that they can speak a little bit more freely and have those views unpicked before they perhaps have any training with the service user.

Interviewer 21:54
So still protecting that that service user?

Participant 8 21:57
Yeah, because I really don't…
Thinking about conversations I've heard, I think, if I'm being really honest, I think trans people are one of the few groups of people that people still think is socially acceptable to ridicule.
Alongside travellers.

Interviewer 22:20
Interesting, can I ask a bit more about the conversations that you've heard? Is it in, in like clinical environments or in like outside of work?

Participant 8 22:31
Both.

Interviewer 22:33
In a hospital environment, what have you heard? If you don't mind sharing?

Participant 8 22:41
I just think it it's probably more the conversation about like pronouns in terms of like I know that that's been sort of like tackled by.
Just one member of like staff who feels strongly that actually we're not particularly inclusive service and when they like argue that actually we're not really even considering pronouns.
People mock them. It's a bit like, yeah, a bit of a joke.
Been thinking about actual quotes.
Oh the chest feeding thing.
I think there is something that because we're a largely, largely a service of women that are midwives. There's something about the kind of conflicts with feminism that’s feeling protective of the journey of motherhood and protective over certain things like breastfeeding and not wanting to change terms to chest feeding for everybody, because that is like, perhaps maybe another experience of womanhood is like having to be altered to… Yeah, I think there there's something about it that actually kind of jars women. But they're like, well, actually this is something that that I do that I want to be referred to as that which… they don't want to change it for everybody. And then that causes a little bit of sort of animosity where it's it doesn't need to, it just needs to be individualised.

Interviewer 24:29
Hmm, so highlighted like making sure any changes are still inclusive of cis women who are being provided.

Participant 8 24:37
Yes. Yeah, yeah. Thank you. I think that's what I'm trying to get at, I think they don't want to... They're not wanting to lose their sort of rite of passage. They confuse it with it being sort of like changed or altered for them when actually it's more about including a group of people who just want to feel like seen, understood and safe, and actually that like as a group of people they're not really accessing maternity care.
Which is a massive fault of our own, that we're not making them feel included. So it is something that needs to be addressed, but it doesn't necessarily take away from their experience
That's gonna be.

Interviewer 25:18
Yeah. Thank you.

Participant 8 25:19
Probably I'm not really answering the question, but.

Interviewer 25:22
No, no, it's, it's really useful. Thank you.
Any other thoughts about training at all?

Participant 8 25:28
I think that's my thoughts.

Interviewer 25:31
That's really helpful. Thank you. So one of the other recommendations was to ask service users their pronouns. What do you think of this?

Participant 8 25:43
Yeah, I think that should be fine. Yeah, I don't… because you're just asking a question like, what would you prefer? In the same way that like it would be embarrassing.
To be, you know, like for example, referring to yourself as [interviewer’s birth name], when you're like, well, I've changed my name, it's [interviewer’s chosen name]. It's just like respecting how people want to be referenced. I don't see that that should be offensive, but also.

Interviewer 25:58
And how feasible do you see it to ask service user pronouns or for other midwives to be asking service users their pronouns?

Participant 8 26:16
Oh, will you repeat the question, sorry?

Interviewer 26:19
How feasible do you see it for yourself or other midwives to be asking service users their pronouns?

Participant 8 26:26
I think it's something that should be done at booking.
And then it should just be clearly like added to their notes.

Interviewer 26:38
Hmm.

Participant 8 26:38
I think possibly like asking every service user at every.
Because like realistically, it's quite unlikely that it's going to change during the path of the pregnancy.

Interviewer 26:50
Hmm.

Participant 8 26:51
Like what? The pronouns when they're booked, I would expect to stay the same, so I think like if we asked once at the beginning and perhaps rechecked when we first saw them for their first 16 week appointment that they we've got it all correct.
That would probably be appropriate.

Interviewer 27:11
Similar to other medical history not having to repeat yourself throughout.

Participant 8 27:17
Yeah. I think sometimes we make a mountain out of molehill. Like if you, if you if someone said like, oh, yeah, like just refer to me as they or he or she. And we're like, OK, fine. And then we double check it when we next see them, just to make sure I've got this right.
I think asking someone at every appointment makes it bigger and it makes it it kind amplifies the issue.
Issue is the wrong word but like yeah, makes more of a thing of it and makes someone feel more... But yeah, OK.
Just call me this and they might even… they might even change. Just be like, do you know what, just call me she if that’s easier for you.

Interviewer 27:55
Hmm.
Oh yeah.

Participant 8 28:04
You know, so I think it makes more of an issue. So just ask once, check once.

Interviewer 28:09
Hmm. And is, is this something that's being done in practise at all?

Participant 8 28:13
Not to my knowledge, no.

Interviewer 28:15
It's OK, and thinking back to that definition of safe care, do you think that asking service users pronouns would impact the safety of care provision at all?

Participant 8 28:29
Yeah, like I'm. I'm just relating it to name for example like if I had gone and had [participant’s child’s name], and everyone had been calling me [participant’s full name], I would have felt very annoyed and I'd have felt distracted and I'd have felt frustrated and I thought, why do I have to keep repeating myself? Why do you not know who I am?
And that would have been annoying in amongst trying to have a baby.
Yeah, I think it could… it could impact psychological safety, and therefore it could impact safety.

Interviewer 29:11
Mm hmm and in terms of, that's an important part of your identity.
Your name being listened to?

Participant 8 29:19
Yeah.
Yeah.

Interviewer 29:23
Just. OK, great.
And do you think that midwives or other health workers would need any additional tools, resources or information to ask a service user their pronouns?

Participant 8 29:41
I think perhaps an example of why it's important for somebody you know, just like perhaps even just like a one minute video of like, I would appreciate you use this pronoun. This is the reason for that.
Yeah, I understand that you may unintentionally make a mistake, but if you intentionally disregard my view, this is what this will feel like for me.
I think, that just a little clip to be like, just understand from like an actual person's perspective that could help, because otherwise I think it's like, you know, you hear phrases like, “oh, the world's gone mad. We're like, everything is so PC” and I'm like.
It's it's not that. It's just to make sure we capture everyone, isn't it? And make sure that everyone feels respected and I think making it like a bit more human by giving
human perspective would be helpful.

Interviewer 30:41
I think and then building on from that one of the other recommendations was to ask service users their preferred terms for their anatomy and bodily function. So you've already mentioned one example of this, which is some people might prefer the term chest feeding to breastfeeding, but some other examples could include a trans person not being comfortable with the word vagina being used for their anatomy. So for example, they may prefer a term like pelvic opening or pelvic outlet or front hole.
Just as two examples.
Any thoughts about this recommendation in general, so asking preferred terms for anatomy?

Participant 8 31:18
Yeah. So this I think is more challenging. Because.
I will give the alternative example like, I cared for women who referred to her vagina as her pussy, and that would be her preferred term of anatomy.
And I'm not saying the two of the same, but I can't start calling her vagina her pussy.
Just because that's how preferred term for anatomy because.
Our anatomy sometimes is really important in terms of like working in a multidisciplinary team that we're actually all talking the same language.
So you know, if I was to go out into the office and be like, I really don't know how to suture this pussy. That would cause issue.
And if I was to go out into the office, it'd be like I don't know how to suture to this front hole.
Like again, it would cause misunderstand and probably unnecessary like ridicule.
And draw attention that they that wouldn't necessarily want so I think.
It's hard to completely change your language of anatomy and not perhaps disproportionately affect their care. I think doing it on an individual basis of like…
I don't know. It's really. It's really difficult, isn't it?
Chest feeing I think is easier.
Because it's more something you're just gonna talk to them about, whereas other stuff might be like talked about.
In terms of like the care you need to provide and includes others.
I don't know.
But then again, like it's kind…. I’m trying to think of like things that I have had to change my language, like hypnobirthing, like it really doesn't bother me to be like I'm going to call it surges because surges feels like less fear inducing than contraction. If that works for you and that helps you to feel relaxed and I'm going to do that, so I think there's an element of when you're providing one to one care to that person, if it helps them to feel more relaxed, then you should try and adapt your language, but for them to expect you to adapt your language for the entire shift, for the entire team could cause confusion.
Yeah, that's what I would say.

Interviewer 33:49
Absolutely. So trying to facilitate things as much as possible, but sometimes being a multidisciplinary reason or medical reason to use a medical term.

Participant 8 33:59
Yeah. And I think I'd probably have to clarify that at the beginning that like it’s absolutely fine, we can call that what, you know what your understanding of it is or like not your understanding but like what you choose to call it because I appreciate you've worked very hard to have like have different like references to your anatomy.
So I'm fine doing that with you on a one to one basis, but when, perhaps, but I'm talking to the registrar, or if there's an emergency, please accept my language may default to like standard anatomy.

Interviewer 34:39
And I think we touched on this a little bit with those initial thoughts. How feasible do you see it for midwives to ask service users, preferred terms for clients’ anatomy and then also to use those preferred terms?

Participant 8 34:57
I think people would feel like uncomfortable asking initially.
Because… Yeah, like I've never asked someone. How would you like me to reference your vagina? I’ve never done it.
But it's not that we're squeamish or like easily embarrassed. It's just…
It would be a new question.
And it would probably again need a bit of training behind it and also a little bit of managing of expectations that I will do my best but there are reasons why I may slip up.

Interviewer 35:38
And just you mentioned training there, is there anything specific do you think that would help midwives to feel more comfortable asking these questions?

Participant 8 35:46
I think again, like you know why someone would want to call it pelvic opening, I have no idea.
I understand chest feeding. Yeah, like pelvic openings, front hole, I've not had any of those terms like used by anybody, so it would feel really clunky to try and change my language and I think I always find it easier to change something if I understand the background and why…
Yeah.

Interviewer 36:18
So a rationale understanding?

Participant 8 36:19
Rationale and a bit of context, yeah.

Interviewer 36:21
Amazing. Thank you and.
Thinking back again to that safe care provision.
With the knowledge you have at the moment, do you think that asking service users preferred terms for anatomy would impact the safety of the care provision?
And if so, in what way?

Participant 8 36:43
Yeah, it's the two sides to the coin, isn't there? So there's the psychological safety for the service user that you're providing the care for by using the correct terms of anatomy for them you made them feel included and respected, but by using the terms that they prefer you may cause misunderstanding and confusion with the multidisciplinary team, which could impact the safety on sort of a practical level.

Interviewer 37:16
Thank you. Any other thoughts about asking for preferred terms?

Participant 8 37:29
I don't think so. I think one thing I would say is.
It needs to be appreciated by sort of like the shift lead that if you're having these conversations, they do take more time.
And that person needs more time, and that providing individualised care, you know, you might not be able to do their ARM [artificial rupture of membranes] within an hour of them getting on to the delivery suite, and you might not be able to do examinations and you might have to kind of think of different ways of providing care and that you don't want to feel that that means that you're doing a substandard job. Or that you're being inefficient, it's that you're providing individualised care and that it's gonna take time, so I think just that sort of allowance for you to be like supported in that, would be helpful because I think what people feel scared of is A, getting things wrong, but B, it's just gonna take longer. To be able to get that understanding and to be able to provide care because there's gonna be more explanation that goes alongside it, you're probably gonna have to be, you know, like you should with everyone, but like it's gonna be trauma informed care and you need to take your time over it and not to feel like you're doing a bad job that day because you've taken longer things.

Interviewer 39:04
Thank you. And then one of the other recommendations again, all tying to these two is the documentation of correct pronouns. So, you mentioned this with recording pronouns at booking. But in general, any thoughts about the documentation of someone's pronouns?

Participant 8 39:26
I mean, if it could be automatically done when you add…
But you know that technology is so advanced, why can't we put someone's pronoun in at the beginning, when they say, you know this, these are my pronouns in their booking. We've double checked them. And then from that point onwards, if we get it wrong, when we write it, that it correct auto corrects because it would be a little prompt to be like, Oh yeah.
I think if technology could help us with that, that would be great.

Interviewer 39:57
Thank you. And I mean, in general, obviously we don't have those systems yet, but maybe one day. But in terms of at that initial booking appointment to document someone's pronouns, is that something that could be feasible within care provision at the moment?

Participant 8 40:05
Yeah. You've asked someone a question. They've given you an answer. You just need to remember to get it right.
Yeah.

Interviewer 40:24
Hmm.
And is this something that's being done at all?

Participant 8 40:30
I couldn't say it “at all” because I'm not in every person's practise or booking, but there's nothing I've heard of.

Interviewer 40:38
OK. And do you feel that any additional tools, resources or information would be needed to document someone's pronouns?

Participant 8 40:51
I think perhaps it's just confidence with the question.
And then that that's, that's all it is. I don't think it's necessarily like a tool as such, it's just, yeah, having it phrased like in a sensitive way, yeah.

Interviewer 41:10
Thank you. And thinking back again to that safety of care provision, how do you feel this would impact the safety of care provision?

Participant 8 41:19
I think it's just first impressions, relationship building, creating a trusting relationship where someone feels able to access care again. This is quite major when it comes to whether they're likely to receive antenatal care, if they attend hospital in labour,
or, you know, access support if they think something's not going right. So I think, yeah, first impressions count, so if we don't meet their needs at the beginning, then we could possibly… They're not going to access care at all.

Interviewer 42:05
Thank you. And then building on from that, the fifth out of six, the fifth recommendation was to make electronic medical record systems more gender inclusive. So for example, most electronic medical record systems don't allow someone's sex to differ from their gender, and some don't allow a pregnancy to be added to a male patient and also don't allow a male patient to be admitted to female areas such as the labour ward or antenatal ward, things like that.

Participant 8 42:38
Yeah, that makes sense, yeah.

Interviewer 42:40
Have you run into any or aware of any problems with your electronic medical record system?

Participant 8 42:48
I only know it from like a same sex relationship perspective where it's like the, you know, the actual question is like you know…
There's a lot of like “the father of the child”.
That I know do cause offence and I think you and then midwives like slip up and then they try and make a joke of it and they go, oh, you know what I mean, and I think it's like, no, it's not helpful like it should probably be the first question you ask: someone's name, their pronouns, their relationship to each other.
And then from that point onwards if the system could kind of like comprehend that information and adjust the questions, that doesn't seem like too technologically advanced.

Interviewer 43:40
Thank you. And does, does your system as you know, allow sex to differ from gender?

Participant 8 43:42

I can’t imagine it does, no.

Interviewer 43:44
And it's it's OK if you don't know, but has there been any limitation? So when there was a transgender person on the antenatal ward, were there any technical limitations to the admission of that person that you know of or not really involved in the care?

Participant 8 44:04
If I'm being honest, probably it would be a support worker that admitted to them. I don't think it would have even… But to be fair, I don't know when they had changed their gender in terms of like NHS systems, so I don't know if it would of caused an issue in terms of that.

Interviewer 44:26
Hmm.
OK.

Participant 8 44:31
Yeah.
Not sure because they were so young. I'm not sure. Like.
Actually that they had adjusted anything in terms of their like medical records.

Interviewer 44:43
OK. That's interesting.

Participant 8 44:44
Yeah.

Interviewer 44:49
Do you feel that there would be any impact on the safety of care provision if the electronic medical record systems did allow these changes?

Participant 8 45:03
No. I feel like they would just like help in terms of safety if they allow the changes because I don't know, just like simple things in terms of like… I don't know. I can imagine if it if it's difficult to admit a patient or you haven't perhaps printed their labels off.
It's like it might then become difficult to access blood products for them down the line. You know, like there's all… everything works in a certain way. So if you struggle to even admit them then you may have delays in their care. Potentially.
Yeah, especially if someone's, like, I can't work this out, it won't… There's something not going right with the system. Therefore, I'm just gonna write on paper.
But that could happen. And then, yeah, you could come into difficulties when you need to do certain things like, you know, even just discharging to the GP.

Interviewer 46:01
So appropriate handovers of care?

Participant 8 46:03
Yeah. Yeah, yeah, yeah. Thanks. That's what I'm trying to say.

Interviewer 46:07
No, thank you.
Any thoughts on I'm kind of going quickly over this one because so much of it is on the IT side rather than on something that we can concretely change. But any other thoughts or comments about electronic medical record systems?

Participant 8 46:21
No, I don't think I understand it enough to be able to have a comment.

Interviewer 46:31
That's absolutely fine. So if it's OK, I'll move on to the last recommendation and this was to increase the gender inclusivity of inpatient and outpatient environments through inclusive decoration and posters. So it's a very broad one.
Any initial thoughts about that in general?

Participant 8 47:00
Trying to think about like what the imaging would be like in terms of…
Even that's complicated because you don't want to make unisex toilets because you really don't want every dad using the same facilities as the birthing person because that's inappropriate.
So yeah, I think you would have to be done in a considerate way where you were like, you're still prioritising your birthing person, not just making it sort of like, yeah, unisex, like facilities. Does that makes sense?

Interviewer 47:52
Absolutely. Thank you. And the environments that you work in, do you see them as particularly gendered?

Participant 8 48:00
Yeah.
Think it's very like.
We... Yeah, it's definitely angled to be inclusive of women.
I think probably a disproportionate amount of midwives have some chip on their shoulder against men.
I actually just think to be in the service. There's something about you wanting to support women, and that perhaps you have some personal experience of being wronged by a man and I think that can sometimes get confused when you're caring for a trans person.

Interviewer 48:42
Hmm.
No, thank you. And then in terms of the posters and decorations.
Have you seen any which you would consider to be trans inclusive or gender neutral in their language?

Participant 8 49:04
Not that I can recall. No, I'm on the like on Instagram, it's like… I think it's called like Queer Birth Club. And then I know that I've seen things that like gender inclusive on there. But in terms of my workplace, I don't think that's been mirrored, no.

Interviewer 49:21
Hmm, so there are resources out there, but maybe not within an NHS realm.

Participant 8 49:26
No, I think the most sort of like advanced thing that we've done is perhaps put pronouns at the bottom of our e-mail.

Interviewer 49:34
OK. And is this something that's done by a lot of people?

Participant 8 49:39
I think it's pretty like scattered, and it'll probably be more like higher management that are doing it.
Because perhaps they've had training, but it wouldn't have trickled down to sort of band 6, band 5 midwives.

Interviewer 49:54
And if there were to be like gender inclusive posters and decorations, what would you envisage as being helpful for service users or members of staff?

Participant 8 50:14
I think it's probably more about sort of like accessing care. The last thing you want is to feel excluded from the even the offer of care. So you know like, I don't know, but maybe even, you know, call if you have vaginal bleeding. And then I think, well, the last thing I want is for someone, it's going to be really triggering for me if they refer to me as having vaginal bleeding. Therefore, I'm not going to call.
Or breastfeed, you know, like there's a breastfeeding support team and then they think, well, that automatically doesn't feel inclusive. And therefore I don't feel like it's for me. So I don't know. I don't know how you overcome it but making women included like, yeah, so like.
I don't know. I actually don't know. I think that's quite challenging.

Interviewer 51:08
That's OK. That's absolutely fine.
And.
I mean, we spoke a bit about inclusion and the naming of services as well with potentially they could be alienating for a person who doesn't identify with their body being that way or as being that gender.
Any other thoughts on posters or decoration?

Participant 8 51:41
I think perhaps we're getting more used to doing sort of like QR codes and things, so perhaps there could be more resources in that. So it's like actually that it's a broad offer of support on a poster that is inclusive of all. And then when you go on there actually that that could be a little bit more tailored to your needs.
I don't know, just a thought.

Interviewer 52:10
Thank you so much. And so that was all of the recommendations.
And I'll just run through the titles of them again. So it was training, asking pronouns, asking preferred terms for anatomy, documenting pronouns, electronic medical record systems, then gender inclusive posters and decorations. Did any of those surprise you at all?

Participant 8 52:40
I think I hadn't really thought through the practicalities of if you identify as like a male… if you're born as a female identifies a male, and then actually admitting you want to like a maternity ward. I hadn't considered that in terms of like technology.
The terms of anatomy that I'm just completely unfamiliar with.

Interviewer 53:10
Thank you. And do any of those seem to be more important to you than any of the others?

Participant 8 53:20
I think we get caught up on pronouns and perhaps overlook terms of anatomy.
Yeah, it's probably a little bit more to like think through.

Interviewer 53:38
And from those recommendations, do you feel like there was anything missing or anything you would have thought that had been included should have been included?

Participant 8 53:52
No, I don't think so. I think that was… Yeah, I think I learned a little bit more than I knew. Though I don't know what else is out there.

Interviewer 54:04
That's OK. So thanks so much for your time, [participant’s name]. Any other comments, thoughts about anything we've discussed?

Participant 8 54:14
No I think even like talking it through with his helpful because it's just a bit more like.
You know you don't know what you don't know, do you? And I think.
I would definitely be interested in organising that training.
For us as a trust, because like the more inclusive we can be as trust, the better it would feel to work there. But yeah, I'm really, really proud that you are doing what you wanted to do, really, like excited for you.

Interviewer 54:46
Thank you very much. I really appreciate that if you're OK.

Participant 8 54:49
I want to keep in touch with you.

Interviewer 54:51
Oh yeah. So if it's OK, I'll end the recording now.

Participant 8 54:54
Yeah.

Interviewer stopped transcription

# Participant 9 Transcript

58m 43s

Interviewer 0:03
So that should be working now.

Interviewer started transcription

Participant 9 0:05
It is working yeah.

Interviewer 0:07
Perfect. So thank you so much for agreeing to talk to me. This study is exploring health professionals experience, knowledge and attitudes towards care provision for transgender and gender non conforming people.
Are you happy to participate?

Participant 9 0:23
Yes.

Interviewer 0:24
Amazing. And are you happy for me to record?

Participant 9 0:27
Yes, I am.

Interviewer 0:28
Amazing. So just some background questions. What's your professional role?

Participant 9 0:34
I'm a midwife.

Interviewer 0:36
Amazing.

Participant 9 0:36
Do you need anything more detail than that?

Interviewer 0:39
Completely up to you.

Participant 9 0:42
I'm a midwife.

Interviewer 0:43
That's fine. And how many years have you been working as a midwife?

Participant 9 0:50
Umm. 15 years.

Interviewer 0:54
15 amazing and how would you describe your gender identity?

Participant 9 0:59
I'm a woman.

Interviewer 1:01
Thank you. And just before we go into the questions, have you had any experience working with transgender pregnant people?

Participant 9 1:12
No.

Interviewer 1:13
And so that's absolutely fine. If you were to care for someone transgender with the current knowledge you have, what would you see as the care priorities for that?

Participant 9 1:27
Hmm.
I would probably ask them what they think would be a priority for them.
And then I would have to do some additional reading because I am…
I have very restricted knowledge, close to none I would say, so I would say…
So we're thinking of me being their case loading midwife?

Interviewer 1:59
Sure. Or caring for them on the labour ward or seeing them postnatally.

Participant 9 2:06
I mean it's the same as anyone else. Where you give the general care to everyone, but obviously you have special consideration because they are transgender. So there may be, you know in the same way that people have their birth preferences. I think that's probably going back to what I said initially, is making sure that I am giving the care that they are expecting and also kind of making sure that as a team, we are providing the care that they are expecting because I'm very much aware that I am one member of a big team.
And there might be information they would like me to share. There may be information they would like me to keep. So I think that's things that we would have to establish together. That would probably be quite challenging in a birthing context, where potentially, I don't know… someone is at home, we've never met before. Hopefully I've had some information before that could help making everyone comfortable because me having no knowledge at the moment, I would hope that my colleagues who have seen that person before have discussed, you know how we can help them have the best experience and have a safe experience. That's always, you know what I'm aiming for, best and safe experience with individualised care plans. So yeah, I think that would be my priorities. I hope that answers your question.

Interviewer 3:32
That's amazing, touched on loads of things there, so like the planning together and also the planning as a team. And then you also mentioned safety of care provision. Can you talk to me a bit about what you define as safe care?

Participant 9 3:48
I guess there's the physical safety and there's the emotional safety, and we tend to think a lot about the physical safety, but the emotional safety is really important. And we're talking about again, asking the person how they're feeling. Maybe if we're in a birthing environment with having, you know, different people coming in the room, how their birthing partners are feeling. And that's probably, yeah. The two sides would be the physical safety and the emotional safety that we would have to explore.

Interviewer 4:30
Thank you so much. And at the moment when you're providing care in general, so not specifically for a transgender person, what tools and resources and information do you usually draw on?

Participant 9 4:52
What do I usually use? For the tool?

Interviewer 4:54
Yeah.

Participant 9 4:56
So you mean NICE guidelines. Our local guidance, the RCOG, there is a lot that I used because as I mean, you know me, but working as a birth options midwife I technically have a lot of resources available, but I have to admit that I do not have specific resources for transgender people.

I do have you know, resources to signpost people to if they have, you know, emotional issues, if they are experiencing Obstetric Cholestasis. You know, there are very specific topics that I know I can say, “OK, there's a good charity here that you can refer to”. But for transgender people, I have to say that I would not have any specific resource that I could use. You mentioned there were resources available. So I would have to check on NICE. You know now that you're raising awareness, I'm like, OK and obviously we have done that training together which has raised my awareness and opened my eyes to so many things. And I've realised there's so much for me to learn.
But yes, I would have to do some additional training. I guess it was one step to do that training that we're together last year, but is there actually NICE guidance for trans pregnancy?

Interviewer 6:36
Not that I'm aware of, but it's just good to know where people draw support on. So if there was to be guidance provided like what form would it be and how could we best help people to gain that information. But I think that would be definitely an important thing to have, some NICE guidance for it.

Participant 9 7:02
Even guidelines we don't have guidelines. We don't have specific guidelines.

Interviewer 7:07
So hospital guidance as well, absolutely. And so any thoughts in general about caring for transgender people in pregnancy? Otherwise, I'll move on to literature questions.

Participant 9 7:22
I guess for me…Yes, I said, there is the emotional side is, you know mainly, you know, focus to them and then in pregnancy and in labour, I guess I'm not familiar with maybe surgeries they would have, what would be the anatomy look like, if we go to a very specific medical terminology. I think where I would stand is what do I expect almost? Because I'm really not familiar with you know what type… Because we are, we are used male and a male body. And I would probably need some guidance or maybe also on medication that that person might be taking because, again, because I'm very new to that topic, I'm assuming it you know, to keep the hormones, you know, balanced. Maybe you have, you are taking medications only, maybe you had surgery.
I would need to explain to that person that I'm new to that. I'm new to, you know, transgender people. That would be my first person. The first person I'm looking after who is transgender and I will have to apologise for, you know, and explain that I am… I'm keen to learn and if they would please help me to learn I would be honoured because this is new to a lot of us and I want to give best care and I know I may not, you know, be… you're never the best. You can always progress basically. But I would love that person to help me in that journey so that I can again increase my knowledge in general but also individualised care for this person in particular. So I think that would be a matter of really establishing that we are a team. So here I guess we're talking in the context where, you know, I'm looking after this person during pregnancy and not just, we're meeting in labour. But if it's the first time I'm meeting someone who is transgender in labour and I have never met someone transgender before, I would definitely need to have some background knowledge.
Or to have had maybe some information from the team who looked after this person so that we limit the amount of time where it can be awkward. Let's put it that way. Because I'm assuming, you know, there might be awkward times, me being new to them and being new to a transgender person.
I hope that makes sense.

Interviewer 10:10
Absolutely. And I think touching on that like relationship building and continuity, that sounds really important to make sure that the midwife feels comfortable, but mainly also that the client feels safe as well.

Participant 9 10:23
And it's challenging because you know that we are aiming for continuity, but we know that in reality, unless you have a private midwife, you're not likely to have continuity, even if you have booked with the homebirth team, you're not likely to have continuity 100%.
So, and we know that there's bias, there's judgement. So we want this person to feel safe, but we also want our staff to feel like relaxed in that, let's say, in the context where we meet for the first time in this birthing environment.
Or in, you know, meeting each other for the first time, say in postnatal, maybe with breastfeeding or chest feeding. I know we're talking about, you know, different terminology as well sometimes, I've heard of that.
But yes, at the moment, personally I feel my knowledge is so restricted that I would not want this person to feel awkward because I am actually feeling like “oh, I am not doing… Am I doing the right thing? Are you know? Is this OK with you?”

Interviewer 11:29
So just that discussion with them as well and you also touched on like the handover with the multidisciplinary team.

Participant 9 11:37
Yeah we have a lot of learning to do and I'm glad that, you know our trust brought that you know that study, not study day but that you know workshop we attended because that was really one step forward. But in maternity we need to move on as well.

Interviewer 12:00
Hmm. OK, thank you.
So, you spoke a bit about language and training. So you had one study day. Have you ever had any other training?

Participant 9 12:16
No. Related to transgender people? No, not really.
No, I just know because of maybe reading or maybe you know videos I have watched but I have not had much information or I have not looked for a lot of information. I have to say about transgender people and pregnant transgender people.

Interviewer 12:54
Yeah. No, that's absolutely fine. And it also sounded like maybe.
The concerns you'd have with the care provision were more to do with, like the medical recommendations than, and please correct me if I'm wrong, than caring for them as a person?

Participant 9 13:16
I don't even know if… my main concern almost would be to make sure that they feel comfortable and they have a good experience. I'm not. I think medically speaking it's more about, you know, if there are medications, managing the medications, finding out how that may interfere with their pregnancy and physically and emotionally, how that may interfere with labour and birth. Again, because are those medications... Again, I am clueless. So take it from there.
Are they going to interfere with the birthing process? Because I want them to have a good birth, whatever birth they have, you know? So if we're thinking of a vaginal birth, is this going to interfere with, you know, establishing labour or maybe an induction of labour? If we're thinking of a caesarean, is this going to interfere with maybe increasing the risk of PPH [postpartum haemorrhage]?
You know those things that you want to be anticipating like, but I guess maternal medicine would probably help with that. But again, do you need to go and see maternal medicine? You know, do we need to medicalize people? If we had a pathway where we could actually understand and have some knowledge, providing that people are taking medications because again, I'm not sure that people are taking medications, once we know, is it that you need to see a doctor? Can we, you know, can we just have it as you know, a pregnancy that only needs midwife input because again having to medicalize people that do not need to be medicalized because of lack of knowledge.

Interviewer 14:56
Absolutely. That's a really good point.

Participant 9 14:58
And then you multiply people coming in and you know you're seeing a doctor at one stage, and then you're going to see another doctor because that doctor may not be there or…
And are those people familiar with transgender people? Are our doctors familiar with transgender people? Because are we generating again more awkwardness? Because that that's probably one of my main concern for people, not to be awkward and that person not to feel awkward in such a special moment in their life.
Yeah.

Interviewer 15:31
Hmm, absolutely. That was really interesting.
So I'll start talking about some of the recommendations that came up in the literature review, and then we'll just talk about each one, what you think of them. You don't necessarily have to agree with them or disagree with them. It's just any thoughts you have and we'll just talk through each of them. So one of the first, and these are in no order it's just things that came up in the literature. So one of the recommendations was training as a part of continued professional development on cultural competency and sexual and gender diversity. What do you think of this?

Participant 9 16:10
Yeah, that's really good. So we've started, I feel like we started cultural competencies with [midwife’s name], that was excellent.
I can't remember that we there was any input about gender identity. I think it was just based on cultural competencies, but that could definitely be something that needs to be added although I would argue that I would prefer the two sessions to be separated.
I think cultural competencies should be separated from the gender identity because there are two, although they can crossover, I think I would not want people to blend it together. I would prefer that I'm giving two different topics so they are separate from each other. I'd yeah, I don't want people to kind of mix and match in a way.
*[The authors have made the decision to redact this information from the transcript due to the risk of identifying the person described]* So that's, you know, that could be a project as well to work on in the future.

Interviewer 18:14
Thank you. And it sounds like you're touching on there the cultural competency in the case of people of colour, black and ethnic minority groups, improving safety.
How do you think cultural competency related to transgender pregnancy, would this impact safe care provision at all?

Participant 9 18:36
It will because cultural competency is necessary 'cause we know that we all have our bias, our unconscious bias, and that we may dismiss, you know, important information or important symptoms because of our bias and may we may compromise the safety of people, but also the experience. So I definitely think it would improve safety if we had some training.

Interviewer 19:07
And do you see, how feasible would you see it to have this training?

Participant 9 19:13
I think feasible. I see it in you. I see that, you know, if we had like a gender training you mean?

Interviewer 19:23
Yeah.

Participant 9 19:24
You know, [midwife’s name] training was excellent. I've had it less than a month ago and I thought, yes, breath of fresh air. Thank you for, you know, for helping me being a better healthcare professional. Thank you for helping me to brush off my, you know, my rusty brain and you know as health care professionals we are always seeking to improve and give best care. And if we feel like there is a gap and it looks like now there's a gap in provision and you are someone who is spotting that gap in provision. Let's try to… let's implement something, so maybe highlighting it to you know our head of midwifery and then once it's established just then maybe going to the RCM and saying okay, we've got that. Does the RCM have any training on gender identity? Because they have those bite size learning online and that's something that can easily be put together. You know if you have the information, they have the IT people or maybe super IT skills but you know.
That's something that once you have the evidence, can be put together very easily and then people can choose to access it, but also trust can choose to give that information as part of their mandatory package of information. Even we've got the e-learning with England EHL or I can't remember.
You know, there could be also some teaching being there. I mean nowadays with you know, look at us doing an interview. I'm sat in my car in front of Lidl, you’re at home. Anyone can take a training from anywhere so if the evidence is there and there's research coming, where there's a gap in provision and we actually have the information that can fill this gap, yes, let's you know, let's put training together, that's in your hands.

Interviewer 21:15
Thank you and what additional tools or resources or information do you think would be needed for an individual to provide training?

Participant 9 21:29
Tools or information? Well, I think the main thing is research, but I mean, you're asking me how I, say, if I was trying to put training together, what kind of resources and tools I would need? So I think I would probably need some, you know, research, but I would also want to speak to users. I would want to speak to transgender people, have experience. What was it? What was the wording? An expert by experience. So someone who's had that experience and someone who can tell me, okay, that's the research. Maybe that's my experience in a specific. So there could be some global training, but also if it's local, have someone who has used our services and you know in a way of feedback. We love a good feedback, but it's also good to point out what didn't go that well? What can we improve?
So maybe interviewing someone or few people who have used our services if it's a local training so that we can work on, how do we improve a provision locally? What are our good points and where can we do better? So yeah. research and experts by experience, I would say.

Interviewer 22:53
Hmm, thank you. I really like that term. That's a really a really nice way to phrase it expert by experience.
Perfect. Any other thoughts about training?

Participant 9 23:05
Meaning, any other thoughts?

Interviewer 23:08
In general, what it would look like if it's important to do in your opinion?

Participant 9 23:15
I think it's important. I think it's also important to, in in term of training, ideally you would like people to be able to discuss together. You would like to have a room where it's not… 'cause I was talking about the bite size learning from the RCM and it's great. But I think for me personally I would love to be able to talk with other people who maybe have had that experience as midwives of working with transgender people or because I may have ideas coming up. I would like to be able to bounce ideas with other midwives and also them to share their tips because they probably have some knowledge that I would like to, you know, learn from.

Interviewer 24:03
Hmm, thank you. So interactive education.

Participant 9 24:06
So yeah, it's good to receive the information, but the interaction is also, I think, crucial.

Interviewer 24:14
Thank you. OK, so one of the other recommendations was to ask service users their pronouns. What do you think of this?

Participant 9 24:21
Mm hmm mm.
Yeah, I think it's a really good idea because you'd like to… It's like your name, right? You want to be called by your name or the nickname that you prefer. So yes, the pronoun is really good and… Yes.
I know it might be also challenging for us to adapt to that. Let's face it, and probably I may you know, if again if I'm meeting someone who likes to be referred to by they or he, I may say, you know, it might be challenging for me, I may make the mistake. I'm really sorry. I'll do my best, I promise you. But I may warn that it might be challenging for me because it's new to me. My daughters and I have been watching…
What is it this thing? Where they’re making the makeup, the makeup artist of the BBC or something like that, I can't remember now.
Anyway, and there are people who refer as they and funny enough, it helped me feel like, okay, when we're talking about this person, we are saying they when we're talking about and that's that helped me in a general context because I know if I didn't have this experience, in maternity as a first hand, it would have been a bit like, okay, I need to make a huge effort. But now because there is some exposure, you know, in the media and it helps you feel more comfortable and it's not even more comfortable but easier to use, because also English is not my first language, so there's some little things that I need to tweak in my mind.

Interviewer 26:01
Hmm. Sounds like that practise there makes it a lot easier.

Participant 9 26:12
Exactly the practise and yes, having it in the media and seeing it on TV and talking about people that I see on TV and saying they and he and I'm like yeah, that's definitely something I can do.

Interviewer 26:25
Amazing, amazing and kind of leads on nicely. How feasible do you see it to be asking service users their pronouns?

Participant 9 26:35
I think it's very feasible. I don't see what…
But again, maybe it's because I've had some training. Maybe it's because I'm interested, you know, maybe there's some bias in me because I am very interested in that topic and I want to, you know, I want to improve. Maybe some people would find it challenging, but I don't see, you know, you ask people, do you want to be Miss, Mrs, or Mr anytime you're, you know, you fill a piece of paper, they ask you, do you want to be any of that? I don't really care if you call me Miss or Mrs or even
Mr. Why do people even ask? So if we ask people if they want to be he, they, I don't know that it would it… I think for the service users it would be absolutely fine and some people may say “I don't want to tell you, I don't want to think about it” and that's fine as well.

Because some people may say, I don't know how to explain. Some people may say why do you ask that? Do you really need to know? Do you need to? You know, in a sense where they might be feeling strong about, you know, I am a woman and I am she, so we don't have to put anything if they didn't want to.
That might be where awkwardness might be for users who are maybe reluctant 'cause we are healthcare professional working to, you know, work on that. But how is that question going to be received?

Interviewer 28:03
And do you? Do you feel that that there would be any additional training that midwives would need to be able to ask about pronouns?

Participant 9 28:12
I think you put… Would you add it in the gender identity training? I think you could combine them because it is related to gender. So we could have it together.
But yes, there's probably some training that is needed, or at least some awareness to be raised. At the moment as we stand, yes, we need to raise awareness because that's still a topic that a lot of people are not familiar with, I think.

Interviewer 28:47
Hmm. And is asking someone's pronoun something that you've ever seen done in practise?

Participant 9 29:08
No.

Interviewer 29:19
Hmm. Thank you.

And if we were to ask service users their pronouns, do you think this would impact the safety of the care provision?

Participant 9 29:19
I would not see why it would, however, thinking of unconscious bias.

It may.

I mean, it would not impact the safety, I would not think so, but you know, sometimes with the culture awareness, you think, “oh, I'm doing that and oh, wow, this is an unconscious bias”. So is it that people have unconscious bias so…
It could impact either way.
It could impact in a way where you're more careful.
And it could impact because of unconscious bias.

Interviewer 29:53
That's really interesting.

Participant 9 29:55
Although I would not initially I thought why would it?

Interviewer 30:01
Thank you. That's really interesting. Any other thoughts about asking for pronouns?

Participant 9 30:08
No.
But going back to the training, I think I'm always still a bit confused, but maybe that's me that you put, she/her or his or they. I think that's still a bit confusing to me, so I still need you know… So yeah, we definitely need some more information about that.

Interviewer 30:34
Yeah, fair enough.
And then the next recommendation actually builds or something you were saying earlier, and builds off pronouns, which is asking service users their preferred terms for their anatomy and bodily function. So what you mentioned already was instead of breastfeeding, some people might prefer the term chest feeding, but it could also apply to other anatomy. So for example, rather than referring to someone's vagina, they might prefer the term like frontal hole or pelvic opening. Something like that for example.
What do you think of this recommendation of asking preferred terms for anatomy?

Participant 9 31:18
I think it's good. I'm seeing it from 2 perspectives. I'm seeing it from the person's perspective and I think it is good to make them comfortable and use the terminology that they would prefer us using.
I think as a healthcare professional, we would probably need to be clear of what we are talking about in a way because… Again, I don't have enough knowledge about that, I guess to discuss like in depth, but in term of being safe and medically speaking and when we're doing procedure, you know, maybe a bit more technical because we, you know we have very strict terminology for anatomy at the moment and we're moving the lines. So I don't know if you would use the terminology that the person would feel comfortable with with them and then have a medical terminology in the background and how would that translate so we don't blur?
So we so we stay safe…
So I guess the terminology that they like, which keeps them emotionally safe, but we want to keep them physically safe and we don't want this terminology if we're not familiar with it, to become confusing and means that we are maybe compromising their safety because we're not sure about what we're talking about. I hope I'm making sense there, that if you say right now a vagina, we know exactly which part of their body we're talking about.
If you're talking, I don't know. Again, you know, front hole. It might be your urethra. It might not be your vagina. So you want to be and in a context of…
I don't know an assisted birth, for example, or you know.
We want we want to be careful. So I think we have to be careful, but I'm all into use terminology that people, you know, want us to use, but we need to be clear in the backgrounds with the staff, with the medical professionals.
Of what that means. How that translates in medical terms.

Interviewer 33:39
Hmm.
Yeah. And I think you've touched on this a bit with that answer, but how feasible do you see it to be asking about preferred terms for anatomy and implementing these?

Participant 9 34:01
I see that it should be… in a way should be simple because again, in a way should be that we have continuity of care and that you tell me that you'd like to be referred as your vagina or that part of your anatomy as front hole. And that's OK, that's what I'm going to be using. But in a way, should we not do that with everyone else? Some people do not talk. Some people who do not, who are not transgender or who identify as women do not know what is a vagina, you know.
There's the basics as well.
You know, when I hear at school, that's my daughters are told their vulva is a vagina. I'm like, oh, hang on so.
There's, there's that barrier as well of.
The knowledge of the person of their own anatomy.
And us being clear, as medical professional, I think I'm getting muddled here, but yeah, you, there's a lot of thinking in my mind with this question.
Because it should be. It should be quite easy to say. How would you like this part of your anatomy to be referred to.
And it's very, it's easier if it’s the same person looking after you, but if it's a new person coming in the room, you know someone who is not used to that.
It might be that it goes both ways as well that you want to warn the person that “yes, you have. Thank you for letting us know that you would like this part. You like this part of your anatomy to be referred as the specific terminology. Just be aware that some people may come in the room and not be aware.
Of your preferences and don't take it personally.” You know, I mean, giving awareness to both as well. Because we all trying to do our best and sometimes we will not get it right.
So let's you know. Let's try to make it right. As you know, to do as best as we can with that person. But it's also maybe informing that person that “yes, that we'll try and do our best, but sometimes there, there might be times where people may not be aware of their wishes or that we may need to use another terminology because of
medical conversations”. That's a challenging one. I didn’t think it would be so challenging.

Interviewer 36:33
Yeah. No, that's fine. So it's slightly less feasible.
Anything that you could see as making it easier for healthcare professionals to ask about preferred terms or to implement it.

Participant 9 36:54
I got the phone. I think anything that they would need.
No, I think once they are… You know it's it would be the same as you know, what's your name? Is it? Oh, you mean the terminology. Sorry.
I don't see…
How complex that could be? Because in a way, if really were thinking about that, we should ask everyone.
It's not. Why would we only ask transgender people? Is it that?
Women people refer to who identify as women say well, I prefer you call my vagina as my front whole. You know what I mean?
Are we going to start to create something?
I guess… If we ask transgender people, I think we should ask everyone. I don't see why that would be different.
And then there's also, there might be also, you know that might be a really good opportunity to do some teaching because some people, again, I'm digressing a bit while not transgender, who have no clue of what it looks like between their legs and they do not know how we call, you know, a vagina, a urethra. And it can sound a bit barbaric because it's medical terminology.
But it's education in a way, things that we should know from, you know, from being a child.
Things that we should have learnt in school, and we also know that being able to name our body parts can help to prevent from sexual abuse. So there might be an opportunity to do some education about asking to name body parts, but is this feasible in a way that do we have time to do that? Do we?
There's also time. Yeah, we have very short appointments. Again, if you have a private midwife, it changes everything. If you're coming in the NHS where we have, what, 15 or 20 minutes per family and then you need to move on. That might be a lot more challenging.

Interviewer 39:19
Thank you. And you also touched a bit about.
Appropriate handovers so not everyone knowing the correct information.
And emergency situations or quickly changing risk.

Participant 9 39:36
In a way, the terminology you could just add it as part of your birth preferences. If really you wants, you know we have this. So at [trust name] we have this you know,
set up template for birth preferences. Is it that we would add a question on “is there any terminology that you would prefer us to use or us to avoid?” Because there's that as well is you know and then we can move on to you know, people who have had some trauma and that being there as well. That question could be used.

Interviewer 40:14
So that really that leads on really nicely to the next recommendation, which was about documenting pronouns.
What do you think of this?

Participant 9 40:27
Yeah, I think that's a good idea, because by documenting your pronouns you also helping people who are seeing you register, you know by exposure.
That you might be they or you might be he, you might be she, might be them.
But by reading it, me as a healthcare professional, I'm like, okay, that helps me, you know, to start a conversation with you. If I've never met you before, someone has documented it, it might be a bit more difficult challenging that now that we're going digital. But it might also be easier because you can just put it on the on the front banner of the people's notes and that may help you also if you see that, I don't know “this person is called Louise and she identifies as they”.
Before you're seeing them, maybe you're going to find out in the documentation CRS, is there something else I need to know before I'm seeing that person that may actually help me to think, OK, is there something additional I need to know so I can give this person the best experience today and I can give best care so in term of safety as well.

Interviewer 41:42
And. So yes, so you kind of touched on that already, but do you think it would make any contribution towards the safety of care provision to do this documentation?

Participant 9 41:52
Sure. Yes, yes. If people are curious, I think it was. And also to help with the unconscious bias, absolutely.

Interviewer 42:05
Hmm. And then going back a little bit to asking about preferred terms for anatomy, I think we spoke a little bit about appropriateness in different situations and the importance of continuity.
How do you feel that asking about preferred terms for anatomy and implementing these would impact the safety of care provision?

Participant 9 42:27
So it may in a sense where you know we were talking about…
So we'd have to be really clear about what is the terminology you say. It could go either way. It could go one way where people feel like, oh, “I'm losing blood through my front hole” and we do know that they mean their vagina and not their urethra because that it could go either way. It could be that you improve safety because actually we know we are talking about the same thing, but it could be that we are deteriorating safety if people are not aware, so if you're coming to me and you're telling me I'm losing blood through my front hole. And actually I'm not aware that you're calling your vagina my front hole.
I'm thinking, OK, this lady's peeing blood. It's different to it's different to you having a, you know, losing blood through your vagina, potentially having an APH [antepartum haemorrhage] or a PPH [postpartum haemorrhage]. Obviously there's an issue here because you're losing blood.
But that changes completely the type of care we're going to give you. Or maybe that may change how we prioritise the care that we're giving you so.
Yeah, it is challenging. It could go either way.

Interviewer 43:53
Hmm, very interesting. Thank you.
So then you also spoke about this with the documentation as well. Moving to electronic systems.
One of the recommendations was to improve the gender inclusivity of electronic medical records. So at the moment, electronic medical record systems typically don't allow a person's sex to differ from their gender, which means that if someone is registered as male, you can't add a pregnancy to their record.
And you can't admit a male person to the labour ward.
So this would be about improving, improving that so that those changes could be made.
What do you think of this recommendation?

Participant 9 44:42
I have no… Hmm.
I guess if we're starting to, you know, that is a bit odd. I never knew. Actually. Didn't even think about it, but.
But.
Why that change that with the IT system? We can admit male people to the labour ward. I guess it's because we're not mixing people in the wards.
It's a very complex question I have to say and it's the same for mixing people who identify as….
Do two bays right and using the same bathroom.
It's… I don't really have an answer to that one. I don't really understand that it is an issue, but I would probably have to read more about it to read about why this is in place. Is this in place just because we said ‘Oh well, only women can have babies”, so when we're creating, the person who created the that software was like, “okay, easy, women only.”
Or is it actually we, I don't know that we have put that barrier on purpose.
I do not really have an opinion. I think you know.
It's small when it comes to actually having people identify as male in, you know, shared room on the postnatal ward that I think I need to think about that.

A lot more.

Interviewer 46:39
No, that's completely understandable.

Participant 9 46:41
About the digital system, I don't, I don't. I don't see why this is an issue that you identify as male and you can't be admitted on the labour ward.
Because then, what are we gonna do then? Do you know what I mean?
We need to admit you somewhere, right?
That probably would need.

Interviewer 47:02
And having systems that don't allow sex to differ from gender and don't allow a pregnancy to be added to someone registered as male.
Do you see this as impacting the safety of care provision at all?

Participant 9 47:50
Again, I will flag unconscious bias.
In term of safety.
On a similar stand as, you know, the cultural bias.
But I don't have any other thoughts about that so far.

Interviewer 48:09
Yeah, no. That's absolutely fine. Thank you. And then the last recommendation.
Through the literature review was to have gender inclusive posters, decoration and signage in outpatient and inpatient maternity areas. What do you think of that as a recommendation?

Participant 9 48:32
Yeah, I think it's good because it's inclusion, you know it's a step towards inclusion. We have. You know, we are now aware of our cultural bias. So now we can be a bit more aware of our transgender bias. So I think that would be a good idea and it also would mean that transgender people would maybe feel a bit less awkward, if they did.

Interviewer 48:59
Thank you. And how feasible do you see it as creating gender inclusive posters or changing decorations to be more gender inclusive?

Participant 9 49:11
I'm trying to think about in term of decoration.
I'm thinking are we very, it's do you mean are we very pink in the maternity units, which I can't really see? I think we're quite we're grey everywhere with flowers. We're quite where I work. But again, maybe it's an unconscious bias and someone would take me around and be like, oh, look at that, this is, you know, I did send you that that postage for the toilets that that might be a good one to explore, is that the toilets? Because I think so far downstairs it's only a female toilets, if I remember well.
And again, I don't really see why men or you know, transgender people could not use those toilets.
So decoration, I don't think that we're really biassed, but again, if someone takes me around, someone with fresh eyes and that's why we have, you know, you could involve our MVP [maternity voice partnership], for example in your study, because they come with fresh eyes and they tell us all, you know, this, that might be something you want to change.
Posters, yeah, I guess, you know, is this available now? Is this something that we have available now?
Is this also do we have users who would like to be photographed, maybe to change, for example, our maternity website is just that we would like to have some, you know, some pictures on our maternity website is aware or.
Yes, that's feasible. I don't see why not. I think, the thing is I'm not the one who is who is saying yes. You know, I don't own that website. I don't own the maternity unit. So you would have to probably discuss with someone who is a bit higher in the hierarchy.
But I don't see why that that would be a problem.

Interviewer 51:02
Hmm. No, that's absolutely fine. You touched a bit there on like representation of of different types of pregnant people and including transgender pregnant people.

Participant 9 51:15
It would, yeah, it would be… It would be good.
Just so that we are.
A bit… It's a bit more familiar, let's say, for all of us, staff, other pregnant people and people in general, non-pregnant people.

Interviewer 51:35
So adding to that normalisation.
Do you think that having gender inclusive environments so in terms of the posters, decoration, signage, would this impact the safety of care provision?

Participant 9 52:01
Again, it can go either way. That's gonna be the same answer again. It's about unconscious bias, so.
I think it needs to be associated with training and awareness amongst staff.
It can't be, because it's something new and we know that novelty can be worrying for people. Especially in our field of medicine, where everything are very much set and gendered.
Yeah.
Actually to have safety not compromised, I think it would be that we would definitely need some training associated with some change of the visuals that we have.

Interviewer 52:54
Hmm. And can you expand a bit more on that worrying that that people might have?

Participant 9 53:01
I don't know. I, you know, I hear people. I've had different conversations that people who are really… I don't know how to, I don't know which words to use for that.
Some people are very… Not against, but they’re just very uncomfortable with transgender people, and transgender pregnancies are almost kind of one step too far.
So, I would… Yeah. That's why I think having some training, some user… experts by experience, to talk and share the experience may help with the unconscious bias, or might not even be an unconscious bias. It might just be a bias full stop.
That some people.

Interviewer 54:07
And that bias is that something you see within some midwives and health professionals or more outside of a healthcare setting?

Participant 9 54:17
It's not outside of the healthcare setting but, I have to say I did not have enough chats with midwives about gender identity to be able to answer this question.

Interviewer 54:31
That's absolutely fine.
And at the moment, do you think gender inclusion, inclusion is something that's considered when posters, decoration website designs are being made in maternity?

Participant 9 54:51
Gender inclusion.
I don't really know.
I'm just even thinking of recent posters. I don't really recall any recent posters, I can recall old posters, but not recent posters so I don't really know if I can answer this question.

Interviewer 55:19
Any other thoughts about that last recommendation in general, so inclusive posters, decorations.

Participant 9 55:28
No, I think we've discussed it all.

Interviewer 55:31
Amazing. So thank you so much. Those were the six recommendations.
Out of those, do any of those seem more important than others to you? And I can run through them again, if that makes it easier.

Participant 9 55:44
Yes, go ahead. Run through them again.

Interviewer 55:45
Yeah. So it was training, asking about pronouns, asking about preferred terms for anatomy, documentation of pronoun, gender inclusive electronic medical record systems, and gender inclusive poster and decoration.

Participant 9 55:46
So… I think training is key. I think it's key. Training is key to raise awareness and then everything will unfold from the training when there's more awareness and less unconscious bias, if they are unconscious bias. But there's always unconscious bias anyway.
Because the pronouns, the naming your anatomy, we kind of touched on that a bit, but we could ask that to anyone. We could ask that if you identify as a woman, if we could ask it to all, all people coming to the maternity unit. But training is something that we need to start working on.
Yeah, to start improving care, basically. We need the knowledge before we we're starting to improve the care.

Interviewer 57:02
Thank you. And are there any of those recommendations that surprised you?

Participant 9 57:14
Not really, but that… It might have been because again, I'm quite new to the topic, I think I would need some more time to think about the recommendations, but I do. I think… Yeah. No, not really. Not really.

Interviewer 57:40
Thank you. And was there anything that wasn't included in them that you would have seen as being important?

Participant 9 57:58
Not at this stage. No. Again, probably because my knowledge is still quite limited and I would need to be with a stirring group to bounce ideas.
But not, to me right now, there's nothing that that comes to mind.

Interviewer 58:14
Oh, amazing. Thank you so much. Any other thoughts, comments, questions?

Participant 9 58:21
No, thank you so much. That was very thought provoking. I definitely need to find myself some more information on transgender pregnancy.

Interviewer 58:36
Fabulous.

Participant 9 58:38
Yeah.

Interviewer 58:39
Thank you. So if it's okay, I'll end the recording now.

Participant 9 58:42
Mm hmm.

Interviewer 58:43
Okay.

Interviewer stopped transcription

# Participant 10 Transcript

1 hr 1m 39s

Interviewer started transcription

0:0:0.0 --> 0:0:17.280
Interviewer
OK, so that should be working now. So this study is exploring healthcare professionals experiences, knowledge and attitudes towards care provision for transgender and gender non conforming pregnant people. Are you happy with all the information you've had so far about the study?

0:0:18.960 --> 0:0:22.400
Interviewer
Amazing. And do you have any questions about the study at the moment?

0:0:23.170 --> 0:0:24.410
Participant 10
No, no questions.

0:0:25.310 --> 0:0:28.830
Interviewer
OK. And are you happy for me to record the audio?

0:0:29.640 --> 0:0:30.640
Participant 10
Yes, very happy.

0:0:31.90 --> 0:0:37.690
Interviewer
Perfect. Thank you. So just to go through some demographic questions, what's your professional role?

0:0:38.500 --> 0:0:39.340
Participant 10
I'm a midwife.

0:0:39.750 --> 0:0:43.790
Interviewer
The midwife and in which region have you been? Have you worked?

0:0:45.160 --> 0:0:46.880
Participant 10
I've worked in [Location].

0:0:48.590 --> 0:0:52.470
Interviewer
And how many years have you worked as a midwife?

0:0:53.320 --> 0:0:56.840
Participant 10
And three and a half, 3 and a half years.

0:0:57.580 --> 0:0:58.380
Interviewer
Amazing.

0:1:0.120 --> 0:1:1.200
Interviewer
And what's your gender identity?

0:1:3.960 --> 0:1:4.120
Participant 10
Um I...

0:1:8.360 --> 0:1:8.840
Participant 10
Female.

0:1:9.490 --> 0:1:10.650
Interviewer
Female. Thank you.

0:1:11.930 --> 0:1:23.50
Interviewer
And so before we look at some of the recommendations from the literature review, just a bit of background questions. Have you had any experience working with transgender pregnant people?

0:1:24.460 --> 0:1:29.500
Participant 10
No. Very sadly, I would have loved to have done it, but I have no experience.
I've watched the seahorse documentary.
And have just talked to other people here and there about their experience, but not had personal experience.

0:1:42.860 --> 0:1:46.540
Interviewer
And what sort of experiences have other people had that you that you know of?

0:1:48.660 --> 0:1:54.580
Participant 10
Well, I have heard. I spoke to someone who had had.
Had supported someone at [hospital trust], and then someone at [second hospital trust], a midwife. When I was a student who had supported a transgender woman.
And they were talking about how they, well, they were talking about how they felt about it and how and the kind of language that they were using, but also.
This was a few years ago, so it was probably around the time that the Seahorse documentary came out. So they were talking about.
The bigger picture and what kind of responses this person had had to their pregnancy and also what it felt like as a midwife to actually a lot of people concerned that they were going to get it wrong in terms of saying the wrong thing or using the wrong pronouns or just having the documentation in a place that it wasn't appropriate really for that person.
So yeah, lots of, I suppose are kind of midwives sort of talking about it because it was a way of processing.
A you know difference and different experiences.

0:3:13.360 --> 0:3:32.480
Interviewer
So you spoke a bit about language pronouns and getting it wrong. Was there any differences in the in the care provision that they that they discussed with you or that you could identify compared to caring for someone who was pregnant and identified as a woman?

0:3:35.940 --> 0:4:0.820
Participant 10
No, I don't think so. I mean, it was antenatal care. We were talking about, not intrapartum care, but as far as I could tell, you know, they had. They were very happy to just be proceeding as they would normally in terms of their care with an awareness that the system itself wasn't really set up to, including the written information.
To feel inclusive.

0:4:6.650 --> 0:4:17.210
Interviewer
Hmm. And from the discussion you've had, what did you feel? What do you feel the priorities from the current knowledge when caring for transgender pregnant people?

0:4:20.980 --> 0:4:26.220
Participant 10
Priorities in terms of priorities from transgender people themselves.

0:4:28.770 --> 0:4:32.130
Participant 10
And you don't want to ask me that to me.

0:4:31.670 --> 0:4:32.390
Interviewer
Oh, no, I mean.

0:4:32.730 --> 0:4:34.10
Participant 10
Like who's?

0:4:34.30 --> 0:4:34.510
Participant 10
Is my question.

0:4:35.200 --> 0:4:36.840
Interviewer
That's a really good question.

0:4:40.550 --> 0:4:41.110
Participant 10
OK.

0:4:38.920 --> 0:4:43.640
Interviewer
As a care provider, what are your priorities for the care provision?

0:4:45.210 --> 0:4:46.770
Participant 10
My priorities are.

0:4:45.440 --> 0:4:49.160
Interviewer
And to be honest, sounds like that was your answer as well, that's.

0:4:49.480 --> 0:4:53.880
Participant 10
Yeah. Yeah, I think so. My priorities are safe, equal care, you know, being it but not just equal but equitable care.
So that, whatever is being offered or proposed is as fully understood and also appropriate for that person, as it would be for anyone.
But also that that person is aware that. Well, is able to, you know, be themselves in my care and therefore tell me, you know, share whatever they need to share, whether that's social or physical, whatever it is, emotional.
So I guess it's holistic care taking in the person.
And being able to. Really like not be afraid to ask them about their experience and ask them how I can.
How I can make them feel… I don't know what the word is now in that moment, but because it's not, yes, it's included and yes, it's… important.
But it's more than that. Make them feel more like themselves, which I know is complicated in pregnancy for a transgender person because there is often like body dysmorphia and stuff going on.
So I guess, you know, I would want them to have an awareness that I have a small awareness.
But and be very honest about that and say it's thoroughly inadequate. I can't, you know, I can't possibly understand what's going on for you in terms of your body and your mind right now, but I do want to be here for you.

0:7:15.360 --> 0:7:17.280
Interviewer
Sounds very individualised.

0:7:18.90 --> 0:7:18.770
Participant 10
Yeah. Yes, 'cause, it's gonna be that. That I suppose that experience of being pregnant is gonna be very different for each person, isn't it, 'cause? It's very dependent on.
How they feel about their body and what stage they're at, if they are transitioning, if they're not, you know all of that stuff.
Will it influence how they feel about being pregnant and what it means for the stages of pregnancy?
I think there's probably, and this is just an assumption, but I'm guessing changes in pregnancy or is always a big theme in antenatal care, but it's, you know, anticipating changes.
And dealing with the emotional side of changes might be even bigger for a transgender person.

0:8:11.180 --> 0:8:24.980
Interviewer
And if you were to provide care for a transgender person today, tomorrow, what sort of resources, tools do you have available to support you that you know of?

0:8:35.110 --> 0:8:47.310
Participant 10
Well, good question. I don't have any tools really. I'd love to know what resources are out there to, you know, signal to people and signpost.
And I know that our usually policies, but also just the documentation that like files or apps or whatever people get is usually all female orientated.
No, I don't know. I don't know. I couldn't say “Go to this website and this is where you'll find like people asking questions who might have some of these similar concerns or want to seek answers together.”
I hope there are websites for healthcare professionals that do exactly that, that like maybe have a section for healthcare professionals as well as people themselves, transgender people themselves.

0:9:52.10 --> 0:10:2.370
Interviewer
Some but it. But it's really interesting to hear that it's like no, no formalised pathway within the hospital that you can that you can identify.

0:10:3.520 --> 0:10:6.520
Participant 10
No, I can't think of a pathway that, I mean, I can't think of a place, whether that's website or part of local maternity website or person even you know, some trusts maybe have a specific person who… It's just thinking about diversity and equity.
And it's supporting other midwives with guiding and making sure that training, you know, education around.
What it means to be supporting a wide range of people with different identities.

0:10:56.30 --> 0:11:7.70
Interviewer
You've touched on loads of really useful things there that that would help care provision, which we're actually going to discuss later with the recommendations, which is so fantastic.
But thinking about yourself as a practitioner, so this is just before we start thinking about these recommendations.
I feel that you've covered this in terms of the care provision that you that you would have if you were to have a transgender client today. But could you talk a little bit about your care philosophy?

0:11:28.290 --> 0:11:28.410
Participant 10
Hmm.

0:11:29.530 --> 0:11:33.930
Participant 10
Yeah, I love this kind of question 'cause. It makes me think so hard.
My care philosophy.
I have a very.
Strong belief in.
The ability of.
And I'm gonna use the word woman here, woman's body to birth a baby.
And with trust I have, I have a high level of trust in that process.
Which means that my approach to care.
Generally.
Calm.
Observance.
And curious.
And positive.
And kind of watchfully.
Hopeful.
And I.
Also use you know physiological strategies to enhance.
Pregnancy, but also the birth itself.
And I I'm and I do absolutely, you know, care about.
People who would otherwise be.
Minimised within the system.
Being heard and listened to.
And you know, know that that's the only way that we can begin.
Make care more equitable.
So if I can work with people.
You know, have less voice.
Then I love to be able to do that.
And I've always I went even from the first placement of students, I drew this picture and I thought actually what I'm trying to do is bring home whether obviously home birth is one thing, but actually wherever I am, what does it mean to be bringing home to that person? Like a spirit of home.

0:14:3.40 --> 0:14:3.840
Interviewer
That’s lovely.
And within your care provision, if there weren't any constrictions on your practise environment or work environment, would your philosophy be any different?

0:14:19.230 --> 0:14:20.950
Participant 10
If there weren't any restrictions.

0:14:21.240 --> 0:14:29.680
Interviewer
So, for example, often people talk about being time restricted in the care they can provide.
Or perhaps for some tools which some people might find helpful might also be constricting for them.

0:14:38.230 --> 0:14:38.990
Participant 10
Right.
I don't think. I mean, I feel like my philosophy is my philosophy. That doesn't change. My practise could change.
And my practise on any given moment or in an hour could be forced to change, but my philosophy wouldn't change. So no.

0:14:57.690 --> 0:14:59.10
Interviewer
That's good and.
Again, just thinking a bit more broadly about care provision, how would you define safe care?

0:15:9.920 --> 0:15:18.160
Participant 10
The safest care I think, is care. That's really looked at the individual and really listened to what's needed.
Understands as much as possible about their background.
And.
What their priorities are.
And then.

0:15:35.720 --> 0:15:39.80
Participant 10
You know, talk openly about.
Whatever's there, clinically, dilemmas or whatever.
And again, like adapts in the moment, but prioritising the person at putting them at the centre.
I mean, obviously, clinical safe, all those things too, but.
All those things are necessary for safe care, but the safest care is I think, I guess, two things. Putting the person at the centre and whoever they are and what they need.
For emotional and psychological and physiological safety.
And then allowing the physiology to do itself, do it, do it's work.
Enabling.
That's that's. That's a picture of safety.

0:16:29.860 --> 0:16:31.780
Interviewer
That's great. Thank you so much.
So if it's OK, I'm going to present some recommendations from a literature review focusing on care provisions for transgender pregnancy.

0:16:51.380 --> 0:16:52.140
Participant 10
That's alright.

0:16:44.340 --> 0:17:2.500
Interviewer
They’re in no particular order, and it's just some discussion about what we think about them and their feasibility in an NHS setting. So one of the recommendations was training as a part of continued professional development on cultural competency, including sexuality and gender diversity.
What do you think of this?

0:17:6.160 --> 0:17:7.600
Participant 10
I think that's a really good idea.
I think there's there is sensitivity amongst midwife and of course the general population as well about.
When I've been talking about this, I think last month or two months ago about removing the word woman from documents and posters and other communication.
So I think this subject brings up a lot of feelings and I think that makes it even more important to be doing. And again, maybe it's not, just maybe it's not training as much as education.
Like enabling there to be a real conversation about what's needed but also, you know, hearing stories from transgender people.
For it to be like from the voice of the person as opposed to institution says we should be doing this.
Like what would it be like for midwives to have an experience even if they haven't cared for someone personally, through storytelling, through video, through other midwives experiences even.
Where we have an encounter with a person, not just another set of guidelines.

0:18:46.510 --> 0:18:49.910
Interviewer
So, so tied to a real life experience.

0:18:49.720 --> 0:19:2.80
Participant 10
Yeah. Yeah. And not just a study - Trans person walks in the room, “blah, blah, blah”, but more like who is this person, like give us more than just a scenario.
Give us a person and. And I'm not saying parade a person in front of a room. I'm saying like use stories, use videos, use real dilemmas as well, like.
Try and silence the voice of the person who says “I feel uncomfortable with this or I'm really nervous about the fact that we're taking the word woman away.”
Like that's a very valid perspective and it's so important I think that the kind of sustainability and longevity of and depth of equity not to silence that voice.

0:19:45.0 --> 0:19:53.880
Interviewer
Can you tell me a bit more about discussions you've had about like the changing or removal of certain language in maternity?

0:19:58.940 --> 0:20:3.140
Participant 10
Yeah. I mean, I've only heard amongst midwives, I've only really heard.
A few sort of overheard conversations. Almost.
And with some midwives feeling kind of disgruntled about.
Changing language generally, but particularly that the fear that the word woman's going to get taken away. And of course, my direct contact with this is more reportage on what is happening in the Women's Health world.
From different writers, but, particularly Milli Hill, who is writing about the word is woman and giving examples of where it's been taken away and just… I suppose.
Describe or I mean it's she's not even really trying to describe. She's just showing examples.
The fear being that the, you know, the actual message is lost because the language has become so neutralised. The health message is lost about endometriosis or whatever it is.
So I've overheard conversations like that amongst midwives. But I know that like in my trust, we haven't even attempted to adapt guidelines or even documentation that goes to families and parents. As far as I know, whereas I know some trusts have.
So it hasn't confronted midwives like really intensely yet.

0:21:48.650 --> 0:22:5.530
Interviewer
And so it sounds like if I'm understanding what you're alluding to, somewhere in the middle where there's a space for inclusion, but there's still space for cis women to have their identity validated and used correctly.

0:22:7.800 --> 0:22:12.680
Participant 10
Yeah, I mean I strongly feel that the word woman should be maintained in thing in in Women's health related information and documentation.
But I think, yeah, absolutely. There is a. There's a place where we can.
Make sure other people and transgender women feel comfortable and included because they feel their identity has been represented.

0:22:46.880 --> 0:22:50.920
Interviewer
And yeah, thank you. That's really interesting to hear.
In terms of training and specifically training, using storytelling and potentially people with lived experience, how feasible do you see this being within current NHS settings?

0:23:6.570 --> 0:23:9.810
Participant 10
I think it's perfectly possible, I mean.
It's what we would be aiming to do with.
You know, to talk about the experiences of.
Vulnerable women or families.
I mean, I think the feasibility.
Decreases with the problems of time and getting people together to do any kind of education work, but it could be part of monetary training, you know, that's the sort of thing that we should be talking about and giving space to.

0:23:49.720 --> 0:23:57.680
Interviewer
And what? What tools, resources or information do you think a trust might need to facilitate the training?

0:23:59.90 --> 0:24:1.610
Participant 10
Certainly some of those websites I don't know about.
And hopefully you know, they have some pieces on there which have been carefully crafted as lived experience stories that can be used with permission.
I was I thinking they need really skilled facilitators because it can be so clumsy and because you need to leave space for some disagreements, some discomfort.
And maybe just for like clearing up some assumptions as well. That can be that can be really uncomfortable for some people. I I think you need skilled facilitators and that it doesn't always mean midwives so actually.
And it also doesn't necessarily mean… Well, it might be organisations that focus on.
Supporting transgender people in in pregnancy and birth who can, you know, sort of you understand the lived experience, whether they've lived it themselves or not.
And can be advocates and... But from that place of exploration together rather than like
“It's what you should be doing”.

0:25:42.430 --> 0:25:51.230
Interviewer
Hmm. So open having conversations where it's open for discussion and multiple viewpoints can be heard.

0:25:51.990 --> 0:25:53.830
Participant 10
Yeah, yeah. And of course there may be transgender midwives, who've also had experience of pregnancy, who know that there could be a combination within identities within a person, including midwife, which would be interesting and helpful to hear. But in terms of volumes of people, you're more likely to be able to get support from organisations who regularly support people and who have a experience of transgender pregnancy.

0:26:34.630 --> 0:26:43.70
Interviewer
And in your experience, or to your knowledge, is there currently any training on caring for transgender people in pregnancy?

0:26:44.150 --> 0:26:57.790
Participant 10
No, not in, not as a standardised part of a training package, not in our trust anyway, but I'm sure there is another trust and I'm thinking [hospital trust].
I would be flabbergasted if there was no training there.

0:27:3.550 --> 0:27:6.270
Interviewer
Yeah, they have. They do have a policy, yes.

0:27:6.400 --> 0:27:7.720
Participant 10
Yeah, yeah.

0:27:15.840 --> 0:27:28.760
Interviewer
Hmm. And thinking back to that discussion on safe care, being focused on the individual, do you think that training would impact the safety of care provision?

0:27:32.840 --> 0:27:40.240
Participant 10
I do. Yeah, I think. Well, I think and again coming away from that word training and instead focusing on education, reflection, exploration even.
As soon as we start to recognise our own assumptions and prejudices, then our care improves. Because we can see how we haven't been equitable or we can see how the system's not serving the person.
So yes, definitely. And of course, out of that there could be very practical actions.
And simple things that can be implemented that increase safety because someone feels more heard or is more heard or they get what they need at the right moment. Whether that's you know medication or a conversation with the right person in terms of mental health or whatever.

0:28:43.140 --> 0:28:50.220
Interviewer
Thank you. Any other thoughts or comments about this education story sharing reflection?

0:28:54.760 --> 0:29:0.120
Participant 10
I mean, I guess it could, some of it could be done online, 'cause, if you've got the right resources and you're asking people to watch a video or a documentary or whatever.
You can do some of that online, leaving people a bit of space to kind of take it in before they're confronted with the group conversation, but then to pick up the threads as a group so that you can talk about what you what you're trying to do as an organisation.

0:29:28.920 --> 0:29:31.320
Interviewer
So coming back again to that facilitation?

0:29:31.990 --> 0:29:37.510
Participant 10
Yeah, I mean, I do think it, it's so important. It's so important.
And it's the things I mean, you know, my big theme around midwives and well-being, it's one of the things that is just, you know, for nurses, midwives and others, if the NHS was to invest in just a few people who really knew how to simulate groups.
There would be, I think, a feeling of. greater well-being because that can be that normalisation and increased comfort of talking about difficult things.
Being supported, but it you know, midwives have never had any training on how to handle groups and how to be in groups and how to support open conversation, so I think it's very unrealistic to do that with very hot topics.

0:30:35.50 --> 0:30:43.930
Interviewer
Hmm. Yeah, thank you. So another one of the recommendations was asking service users their pronouns. What do you think of this?

0:30:46.920 --> 0:30:48.680
Participant 10
I think that's fine. That's… Yeah, I think I mean, it's part of our booking process now as well.
And we're asking them demographic questions, and I know this is an identity question as well. But we're it's, you know, you're asking them “do you do you go by other titles?” You know there's a whole range of questions that are completely valid and. And yeah, I think that's fine.

0:31:23.10 --> 0:31:30.530
Interviewer
Hmm. And. And you mentioned it, it's part of the booking process already. How feasible do you see it to ask people their pronouns?

0:31:32.730 --> 0:31:33.890
Participant 10
It's definitely feasible.

0:31:37.10 --> 0:31:45.90
Interviewer
And do you feel that midwives or yourself would need any additional tools, resources or information to be asking these questions?

0:31:49.790 --> 0:31:55.310
Participant 10
But they might need to ask that person. Are there certain circumstances that, like when would you, Like where if your pronouns are, they/them.
Well, maybe they just. They just have to think about it. 'cause, I'm having to think about it right now.
Yeah, I don't think they need extra resources. I think it's just it's part of the education process and just like. And personal reflection. Just like. OK, I'll think about it. Like, what am I going to need to do to adapt, not just what I say, but also how to present the material to you which may not at all be inclusive. I think that's just about having a human conversation and saying that for them.

0:32:55.600 --> 0:33:2.600
Interviewer
Yeah. And is asking pronouns something that is currently being done in in practise that you've seen.

0:33:3.470 --> 0:33:6.990
Participant 10
I think it's being done at the booking appointment.
I hope it's, I hope it's been done. I don't know. Obviously no one knows.

0:33:21.580 --> 0:33:22.60
Interviewer
And.
Do you feel that asking pronouns would impact the safety of care provision at all?

0:33:31.240 --> 0:33:34.200
Participant 10
Yeah, I think if people feel able to, I mean if then if you haven't asked and you go on using the wrong, or, you know, an inappropriate pronoun or an uncomfortable pronoun through the pregnancy that only affects how someone feels about meeting you, what they feel, I guess. Comfortable about revealing in terms of safety at home or other kinds of safety, symptoms, that kind of thing, I think. Yeah, it can definitely affect safety of care. It's the consequences of not knowing.

0:34:17.190 --> 0:34:21.710
Interviewer
Yeah. And it sounds like you're talking about that relationship building as well.

0:34:21.650 --> 0:34:25.450
Participant 10
It's about relationship. Yeah. Yeah. And it's an opportunity to really establish trust, I think. And we all know how important that booking appointment is for setting the pregnancy off in a positive way for them, but also for the relationship itself.

0:34:48.130 --> 0:34:48.930
Interviewer
Thank you.
Any other thoughts about asking service users their pronouns?

0:35:5.540 --> 0:35:6.460
Participant 10
No, I don't think so.

0:35:8.260 --> 0:35:37.860
Interviewer
Great. Thank you. So kind of building up from that, another one of the recommendations was asking service users their preferred term for their anatomy and bodily function. So for example, one that people have often heard of is chest feeding that some people might find that more comfortable compared to breastfeeding, but some other examples could include someone having a preferred term for their anatomy. So instead of being comfortable with the word vagina. They may prefer something that is slightly less gendered. So for example, frontal hole or pelvic opening as just two examples. What do you think or what comes to mind when you think about asking service users preferred terms for anatomy?

0:35:59.520 --> 0:36:0.600
Participant 10
Again, I think that's fine. I mean, it's quite it's quite interesting to hear because it reveals something about that person. I mean it reveals something about how they feel about that anatomy.
What they want to be called I guess, but I mean you'd have to be curious about that 'cause you don't know but if you're gonna ask that you can't just go “so what would you what? What are your preferred terms for anatomy?”
I think you need to go a bit deeper as well as say, and, and therefore does that… Does it mean that there are certain things that I could say about the way a body changes in pregnancy, that might make you uncomfortable or, you know, how are you feeling about the pregnancy now? What are you anticipating and feeling about what it might feel like at 24 weeks or 32 weeks or whatever?
What do you know about how a body changes in pregnancy and how do the terms relate to that? I think again, it's getting away from that thing of like “what are your pronouns and what do you want the this this anatomy to be called?” Without actually knowing anything about the person feels like a tick box exercise.
And I think that's what people get. And then, what's the word… about is that feeling of like you're doing it, but you're still not getting to know the person, so you're trying to do it to be inclusive, but you actually have kind of missed the mark somehow?

0:37:56.410 --> 0:38:1.730
Interviewer
So not reducing it to a tick box exercise of asked then finished.

0:38:2.540 --> 0:38:5.780
Participant 10
Yeah. And then you're like, oh, I just got to remember not to call it a vagina, rather than like, let me get a bit of back story here and how might this person really be feeling? Because that's the reason that they don't want to be to be called a vagina.
Like I need to know more about that otherwise I've just got nothing to hang it on.

0:38:24.190 --> 0:38:35.950
Interviewer
Hmm, it's really interesting. You're seeing these as kind of conversation starters or diving boards into a much deeper, deeper conversation about that person's life.

0:38:36.500 --> 0:38:36.940
Participant 10
Yeah.
Yeah. I mean, in a way that's what we're doing with anyone, right? So if someone says to me, you know, I say “so what are your preferred pronouns?” And they she says she/her.
You know, I really want to have a conversation with her about what it means to be a woman. This woman, woman who is now pregnant and has a changing body.
And who may have had an eating disorder previously or may have had childhood sexual abuse or whatever. You know, we don't know any of those things. But actually what we're trying to do is establish a relationship that might enable those conversations to open up.
That would be optimal, right? For everyone to start with those conversations and to say I'm asking you about your pronoun partly because it's important that everyone feels included, but and that, you know, we're able to use a language that makes people feel they can be in their own identity. But also I'm asking because there may be certain things about being a woman now or a transgender woman now which… And in your changing body, that may feel uncomfortable. I mean that's that was applicable to everyone, right?
So I'm not minimising the transgender experience. I'm instead saying, what would it be like if we use these conversations to go deeper with everyone?

0:40:17.110 --> 0:40:17.830
Interviewer
Amazing.
Do you see this as something that's being done in practise currently? So using these initial questions to go deeper firstly in them?

0:40:32.710 --> 0:40:33.150
Participant 10
No. I mean the pressure on the timing for the booking appointment is a problem.
But I think there's room for it. I think there is room for it with an hour and a quarter or an hour and 20 or whatever.
It's it depends what's prioritised, I guess and obviously a skilled booking appointment can be… You know, there's certain things that you can kind of bounce through.
If you sent well, certainly with the transgender person like the yes, you've got to do all the essentials, but you wanna be focusing your energy on that conversation, or at least starting that conversation.

0:41:25.500 --> 0:41:27.220
Interviewer
And do you see preferred terms for anatomy being used in practise, ever?

0:41:34.550 --> 0:41:38.630
Participant 10
I've not been close enough to this situation to hear, to hear it now.

0:41:39.570 --> 0:41:40.770
Interviewer
Hmm, OK.
In terms of the safety of care provision, I think again we've kind of touched on this answer already. Do you feel that asking for terms for anatomy would make any impact on the safety of the care provision?

0:41:57.300 --> 0:41:59.460
Participant 10
Yeah, I think same thing, I think it would. Maybe. I don't know, maybe less so than pronouns, but maybe not. I would want to ask a transgender person that.

0:42:12.690 --> 0:42:14.650
Interviewer
Any other thoughts on that one?

0:42:18.380 --> 0:42:19.660
Participant 10
No, no more thoughts.

0:42:20.590 --> 0:42:21.270
Interviewer
Amazing.
So kind of building off those two, another one of the recommendations was to document the correct pronouns. So this could be in a variety of places, so it could be on handovers when you're writing about the individual and as a alert or somewhere prominent on the notes, what do you think of this as a recommendation?

0:42:50.20 --> 0:42:52.140
Participant 10
To document clearly?

0:42:52.560 --> 0:42:53.320
Interviewer
Yes.

0:42:54.320 --> 0:42:57.80
Participant 10
I think that's a good idea because it's so easy to miss, and if it's documented in small ways in time, any notes or in computer documentation, people aren't going to see it.
And you know that could be an opportunity to say to this person, “I'm gonna put a sticker on your notes that clearly shows what your preferred pronouns are. One of the reasons for that is that care, certainly if you ever have to come to hospital, care can feel a little fragmented, you might be passed… your notes might be passed from person to person and it's really important that it's visible up front to them.” So you can start to talk about what it might feel like to be in a hospital environment versus a home environment or, you know, choices of places of care and place for birth as well.

0:44:1.130 --> 0:44:6.530
Interviewer
Sounds like it's still involving that individual in the documentation that you're doing.

0:44:7.270 --> 0:44:16.310
Participant 10
Yeah, exactly. It's like there's a there's a story here. It's not just I'm gonna stick a sticker on your notes, but the story is you have choices.

0:44:18.110 --> 0:44:18.950
Interviewer
Thank you.
And so again, I think you've touched on that in terms of in the front of the notes, where would you see it being useful for pronouns to be documented?

0:44:38.760 --> 0:44:48.520
Participant 10
I think in places as that are as visible as possible, the go to places, wherever that is, and for some people that'll be an app. For some people it'll be a computer system.
But wherever people go most regularly, it might be paper notes, bits and pieces like for us, the kind of green notes the antenatal notes make it you know it needs to be really visible on there as well.

0:45:7.30 --> 0:45:11.390
Interviewer
Hmm. And is documenting pronouns something you've seen before in practise?

0:45:12.740 --> 0:45:14.140
Participant 10
Yes, I have seen that.

0:45:15.750 --> 0:45:20.550
Interviewer
And how's it been for you to see that? Has that been helpful? Unhelpful?

0:45:21.330 --> 0:45:22.50
Participant 10
Really helpful.

0:45:25.290 --> 0:45:27.690
Interviewer
OK, good and do you feel the documentation of correct pronouns would impact the safety of care provision at all?

0:45:41.660 --> 0:45:42.300
Participant 10
Yes. Because I think it probably builds people's confidence to speak up for themselves when they're feeling more able to represent themselves.
And again, we know how quickly, you know, hospital care can move. And how much you need to advocate for yourself so I can. I can only imagine what it feels like to not… Yeah, to feel like out of sorts with yourself out of, well, I had like half seen, half heard.
But that must it must affect how you feel about them speaking up for your care.
Yeah, I think it's important.

0:46:47.10 --> 0:46:47.810
Interviewer
Thank you.
Any other thoughts about that documentation of pronouns?

0:46:54.560 --> 0:46:55.640
Participant 10
No, I don't think so.

0:46:56.620 --> 0:47:2.780
Interviewer
Great. And so I'll move on to again, it's tied into something which we discussed briefly about commuter systems.
One of the recommendations was to have electronic medical record systems, which were more gender inclusive, so some examples of the limitations of some of the current systems is that they may not allow sex to differ from gender, and they may not allow a pregnancy to be added to a patient that is recorded as male and then due to this they also may not allow male patients to be admitted.
To maternity inpatient areas. So for example, the labour ward.
What? Any thoughts about those the current limitations?

0:48:12.520 --> 0:48:20.360
Participant 10
So I think it's really important to sort out this, whatever the structural things, the structural barriers might be to… Like simple things like enabling a person who's documented as male to be admitted to labour ward or have a pregnancy added.
Yes, that should be sorted out straight away because transgender people no doubt report hitting structural barriers all the time in life and then being recognised and we shouldn't be adding more to them.

0:48:57.760 --> 0:49:4.920
Interviewer
Thank you. And have you seen any limitations of current electronic medical record systems in practise?

0:49:7.90 --> 0:49:14.130
Participant 10
I haven't, but I can only imagine, you know, that knowing what we know about record systems. I can picture there being red flags coming up and you know, cannot proceed, “computer says no” stuff going on all the time.

0:49:31.860 --> 0:49:40.780
Interviewer
How do you feel? Do you feel that the limitations of these current systems would have any impact on the safety of the care provision?

0:49:42.210 --> 0:49:46.90
Participant 10
Yeah, I think it's horrendous when… I mean, that's the old, you know, in a way, a computer system saying, “oh, sorry, we can't do this because you're not… You're not the right gender” is a… It's a horrible way to, like, start a pregnancy. For example. Like. Oh, well, I can't. I can't document you. And you can imagine like clumsy words coming out with it, possibly a midwife or someone else saying, oh, yeah, we can't document you. We can't make the pregnancy official or whatever. Because it's not possible and it just being an absolutely horrible situation.
So yes, it should be. It would definitely affect the care provision because again, it denigrates or diminishes trust in the institution or provider as well as in the person.

0:50:44.160 --> 0:50:45.960
Interviewer
Thank you. And if electronic medical systems were to be made more gender inclusive, what, again, this is potentially outside of our remit, but is there any specific tools, resources or information that you think would be required to do this?

0:51:8.340 --> 0:51:11.500
Participant 10
I really don't know the answer to that. No idea.

0:51:11.240 --> 0:51:11.520
Interviewer
Yes, more of an IT question.
Any other thoughts or questions about the electronic medical record systems?

0:51:25.210 --> 0:51:25.530
Participant 10
No.

0:51:26.800 --> 0:51:50.40
Interviewer
Amazing. So then the last recommendation on the list was to create more gender inclusive outpatient and inpatient maternity areas. So I think we touched on this at the beginning with some of the discussions you'd heard or seen, but this would include gender inclusive posters and decoration and signage.
Obviously this is a very a very broad heading, but does anything spread to mind or any?
Any thoughts about that?

0:52:12.970 --> 0:52:17.890
Participant 10
Do I have any thoughts about that? I mean, I think it's it makes sense to.
You know, represent, just as you would want to represent the diversity of your population.
Which you know like race and other things are often not represented in maternity care.
And I think it again, it opens up conversations, it opens up discussions.
I don't know about… I don't know about like areas. What it would mean for areas.

0:53:1.140 --> 0:53:4.700
Interviewer
So some potential examples could be that some people may see a maternity environment as being quite gendered in terms of being quite feminine. So for example, a tendency to use like pink or maybe pink and blue for babies.
There may also be post posters or signage which elude only to cis women being pregnant. So for example toilet saying women's only bathroom when it's for the pregnant people to use.

0:53:43.190 --> 0:53:46.390
Participant 10
Yeah, I can see how that would be…
But I would, again, rather than changing it to pregnant people, I would say “Pregnant women slash pregnant people.”
Which might be clumsy, but I would still maintain that. But yes, I mean if.
That yes, that increases comfort then that's fine.
And yes, the gender thing is just ridiculous. I mean, like in terms of colour, like why the heck would we think of buying everything and…
But that doesn't make any sense at all, all the pink and blue stuff for newborn babies.
And why are those bands those CTG bands pink and blue for no reason whatsoever?
Because it automatically provokes the question in many midwives “are you having a boy or a girl?” which must be quite a provocative conversation for many people.
And again, that's a kind of you know, that's an interesting conversation to have as part of the education process, right? Like, what does it mean for people to hear that question?
But yeah, the environment. I can see like that we just do not need to gender-ify our environments as much as we do.
They can be way more neutral and yeah, take a lot of the edge off.

0:55:27.0 --> 0:55:35.680
Interviewer
It's really interesting, though. You're talking about the environment also being a diving board to like shape conversations and comfort.

0:55:36.380 --> 0:55:36.740
Participant 10
Yeah. Yeah, and how things can be a trigger like those bands have nothing to do with gender. The CTG bands, someone in some company somewhere has decided to make pink and blue bands, but it says something, doesn't it? And it provokes, it triggers a thought in people, which then leads often to a question when they're busy trying to buckle up the bands, which then leads to comfort or discomfort or whatever, we don't know, but depends on the person.

0:56:12.720 --> 0:56:12.840
Interviewer
Hmm.
And going back a bit to in terms of posters and educational material for service users, pregnant women, pregnant people.
Do you feel that that you've seen or that there is any representation of non cisgendered people who are pregnant, so trans men or non binary people who are pregnant?

0:56:38.50 --> 0:56:42.930
Participant 10
I haven't seen any representation in the hospital environments.

0:56:46.930 --> 0:57:2.650
Interviewer
And would see it as feasible to have posters such as these, would you see it as feasible to have that representation?

0:57:4.380 --> 0:57:8.380
Participant 10
Yeah, I would, but I would want it not to be.
I would not want. I wouldn't want it to be so front and centre that it's to the exclusion of the vast majority. So yes, absolutely representation, but not so that the first thing people cis women see is a transgender woman represented.
Yeah, because.
I think that there needs to be a balance there.

0:57:47.370 --> 0:57:49.290
Interviewer
Hmm, absolutely and.
If these posters were to be made, what sort of tools, resources or information do you think would be needed to create them?

0:58:3.750 --> 0:58:10.150
Participant 10
I think again, these kind of advocacy organisations and websites and things that can.
You know, produce high quality places or pictures or whatever with consent from families to enable, like high quality representation.
That's gonna make people feel good about themselves and feel that they're reflected in these pictures or whatever it is people choose to use.

0:58:43.450 --> 0:58:55.170
Interviewer
And then gender inclusive posters and decreasing the gendering of inpatient and outpatient environments, do you feel this impacts the safety of care provision at all?

0:58:56.730 --> 0:59:1.10
Participant 10
Yeah, I think again, because it's linked to identity and recognition and a feeling that the environment is open to you and to your wishes and priorities.
I think that it that increases safety, definitely.

0:59:22.470 --> 0:59:30.110
Interviewer
Any other thoughts about that one? Gender inclusive posters and gender inclusive environments?

0:59:32.850 --> 0:59:33.370
Participant 10
No.

0:59:34.360 --> 0:59:58.480
Interviewer
OK, so that thank you so much, [participant’s name]. That was all six of the recommendations. So I'll just run through the headings of them again. So it was training, asking about pronouns, asking preferred terms for anatomy, gender, inclusive, electronic medical record systems, documenting pronouns and the gender inclusive posts and decoration. Are there any of those that surprised you?

1:0:3.330 --> 1:0:6.50
Participant 10
Hadn't really thought about medical record systems.
But it makes total sense that they can be a barrier.
Other than that, no, none of those surprise me. I guess I haven't thought I hadn't thought to that level about gendered environments either.
But they are very gendered.

1:0:27.960 --> 1:0:32.760
Interviewer
Are there any of those that you see as being more important than the rest of them?

1:0:35.740 --> 1:0:37.100
Participant 10
What was the first one again?

1:0:37.500 --> 1:0:46.780
Interviewer
So it was training pronouns, preferred terms, documentation, record systems and posted some declaration.

1:0:47.540 --> 1:0:49.940
Participant 10
I think training is the most important.
'Cause only out of that… cause the rest of it really flows from there because the motivation to action on the record systems and the documentation and the environment comes from that. And then the, you know, the understanding and the depth of conversation that can come from the pronouns and the anatomy conversation.

1:1:8.280 --> 1:1:14.320
Interviewer
Great. And is there anything that wasn't included in those recommendations that you would have seen as important?

1:1:18.200 --> 1:1:19.160
Participant 10
I don't think of anything.

1:1:20.0 --> 1:1:26.240
Interviewer
OK. Thanks. Any other thoughts or feelings? Anything else you wanted to to raise?

1:1:28.100 --> 1:1:29.140
Participant 10
Don't think so.

1:1:29.900 --> 1:1:34.500
Interviewer
OK. Thank you so much for your time, [participant’s name]. And if you're happy, I'm going to end the record now.

1:1:35.210 --> 1:1:36.330
Participant 10
Of course, yeah.

1:1:36.810 --> 1:1:38.50
Interviewer
OK. Thank you.

Interviewer stopped transcription
